# Long-Chain Cyclic Arylguanidines as Multifunctional Serotonin Receptor Ligands with Antiproliferative Activity

**DOI:** 10.1021/acsomega.4c06456

**Published:** 2025-02-11

**Authors:** Przemysław Zaręba, Anna K. Drabczyk, Artur Wnorowski, Maciej Maj, Patryk Rurka, Katarzyna Malarz, Gniewomir Latacz, Krystyna Nędza, Krzesimir Ciura, Katarzyna Ewa Greber, Anna Boguszewska-Czubara, Paweł Śliwa, Julia Kuliś

**Affiliations:** † Faculty of Chemical Engineering and Technology, Department of Chemical Technology and Environmental Analytics, Cracow University of Technology, 24 Warszawska Street, 31-155 Cracow, Poland; ‡ Faculty of Chemical Engineering and Technology, Department of Organic Chemistry and Technology, Cracow University of Technology, 24 Warszawska Street, 31-155 Cracow, Poland; § Department of Biopharmacy, Medical University of Lublin, 4a Chodźki Street, 20-093 Lublin, Poland; ∥ Institute of Physics, University of Silesia in Katowice, 1A 75 Pułku Piechoty Street, 41-500 Chorzow, Poland; ⊥ Department of Systems Biology and Engineering, Silesian University of Technology, 11 Akademicka Street, 44-100 Gliwice, Poland; # Department of Technology and Biotechnology of Drugs, Jagiellonian University Medical College, 9 Medyczna Street, 30-688 Cracow, Poland; ∇ Department of Medicinal Chemistry, Maj Institute of Pharmacology − Polish Academy of Sciences, 12 Smętna Street, 31-343 Cracow, Poland; ○ Department of Physical Chemistry, Faculty of Pharmacy, Medical University of Gdansk, 80-416 Gdansk, Poland; ◆ Laboratory of Environmental Chemoinformatics, Faculty of Chemistry, University of Gdansk, 63 Wita Stwosza Street, 80-308 Gdansk, Poland; ¶ Department of Medical Chemistry, Medical University of Lublin, 4a Chodźki Street, 20-093 Lublin, Poland

## Abstract

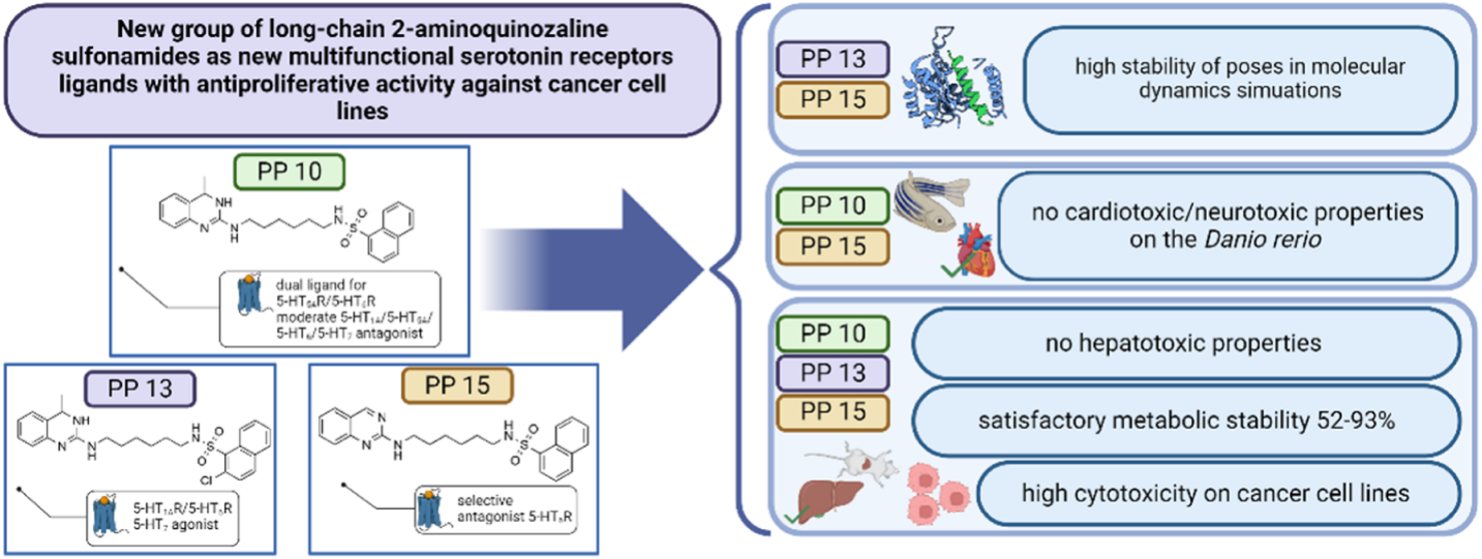

Recent investigations have shown serotonin’s stimulatory effect on several types of cancers and carcinoid tumors. Nowadays there has been a significant increase in interest in 5-HT_7_ and 5-HT_5A_ receptors in the context of cancer treatment. The possible role of 5-HT_6_R in the pathogenesis and progression of glioma remains an interesting and relatively unexplored issue. We developed a new group of long-chain 2-aminoquinazoline sulfonamides as new multifunctional serotonin receptor ligands, focused on 5-HT_6_R. The chosen group was further evaluated for antiproliferative effects on 1321N1 astrocytoma cells, along with U87MG, U-251, and LN-229 glioblastoma cell lines. Certain compounds were subjected to *in vitro* absorption, distribution, metabolism, excretion, and toxicity (ADMET) testing, for assessing factors such as lipophilicity, plasma protein binding, phospholipid affinity, potential for drug–drug interactions (DDI), membrane permeability (PAMPA), metabolic stability, and hepatotoxicity. Additionally, *in vivo* testing was performed using the *Danio rerio* model. The developed group includes the selective 5-HT_6_R antagonist **PP 15**, dual ligand for 5-HT_1A_R/5-HT_6_R **PP 13**, and dual ligand for 5-HT_5A_R/5-HT_6_R **PP 10**. The use of multifunctional ligands was associated with high anticancer activity both against selected glioma cell lines and other cancers (IC_50_ < 25 μM).

## 1 Introduction

Glioblastoma multiforme (GBM) belongs to the most aggressive forms of glioma, accounting for over 50% of all cases.^1^ There are four grades of gliomas, ending with grade fourth, corresponding to the most aggressive tumors.^2^ In the case of GBM, the prognosis remains unfavorable, with a median survival time of only 14.6 months.^3^ Treatment of glioblastoma, mainly based on bevacizumab and temozolomide (TMZ), improves the quality of life of patients and extends survival time. However, the effectiveness of available therapies is limited and causes various side effects.^4^ The pathogenesis of gliomas is associated with genetic abnormalities affecting cell signaling pathways or epigenetic processes. The most common changes concern tyrosine kinase (RTK) receptors^5^ as well as mutations in the epidermal growth factor (EGFR).^6^ Other aspects include changes in the phosphoinositide 3-kinase (PI3K) pathway, where key effectors such as AKT are often activated or enhanced. There is also often loss of phosphatase and tensin homologue (PTEN), known as the tumor suppressor gene negatively controlling the PI3K signaling pathway.^7^ Specific molecular mutation signatures in GBM also include the *TP53* gene encoding the p53 protein, which is crucial in determining cell fate in response to damages by arresting the cell cycle, triggering cell repair, or inducing apoptosis.^8^

Various studies have shown serotonin’s stimulatory effect on several types of cancers and carcinoid tumors.^9^ Psychotropic drugs that affect serotonin levels used in the treatment of mental disorders have shown therapeutic potential in the treatment of gliomas, maintaining good penetration of the blood–brain barrier (BBB).^10^ The studies conducted so far show that the effect of serotonin on cell growth occurs mainly through 5-HT_1_ and 5-HT_2_ receptors.^11^ However, in recent years there has been a significant increase in interest in 5-HT_7_ and 5-HT_5A_ receptors in the context of cancer treatment^12^ Both receptors have also been mentioned in the context of GBM.

Among the factors probably involved in the progression of CNS cancers is the 5-HT_7_R, which was confirmed in the *Drosophila* larval model of glioma. The expression of human 5-HT_7_R in this model reduced larval mortality and partially restored dysregulated biomarkers. Furthermore, some transcriptomic analyses of GBM patients have shown a significant decrease in 5-HT_7_R expression in GBM compared with normal tissue.^13^ Glioma tissues also express the 5-HT_5A_R subtype, but its expression is lower in high-grade glioma than in low-grade. Valerenic acid (5-HT_5A_R partial agonist) limits glioma cell proliferation *in vitro* and *in vivo*.^14^

The possible role of the 5-HT_6_R in the pathogenesis and progression of glioma remains an interesting and relatively unexplored issue. 5-HT_6_R has been shown to bind to cyclin-dependent kinase 5 (Cdk5) as confirmed by a coimmunoprecipitation (co-IP) experiment on neuroblastoma-glioma NG108-15 cells.^15^ It was observed that 5-HT_6_R activates Cdk5 independently of agonists in neuroblastoma and glioma cell lines, as well as in primary embryonic neurons, with native receptor expression.^16^ Cdk5 has been associated with the pathology of many types of cancer^17,18^ and is emerging as a potential therapeutic target for GBM.^16^ In GBM, Cdk5 is highly expressed in clinical tumors^18^ and is a critical regulator of GBM tumorigenesis.^19^ This kinase promotes tumor growth by activating the transcriptional activator STAT3 via Tripartite motif 59 (TRIM59), functioning as a ubiquitin ligase.^18^ Moreover, 5-HT_6_R interacts with other signaling pathways, such as the mTOR pathway. However, these aspects require deeper investigation, the efficacy of 5-HT_6_R antagonist in the gliomas treatment has not been demonstrated so far.

**Figure 1:**
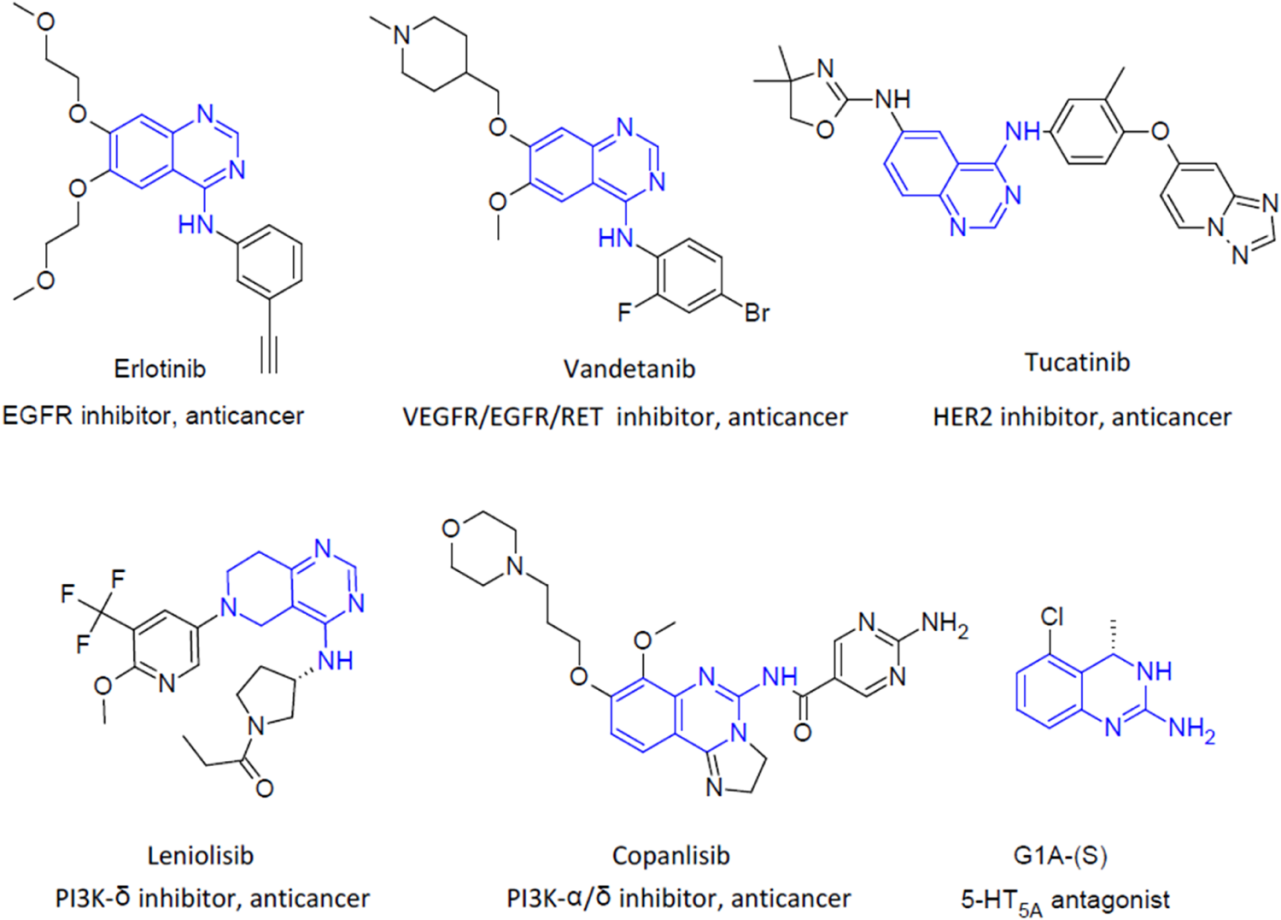
Structures of selected aminoquinazoline derivatives.

Derivatives of aminoquinazoline or partially hydrogenated aminoquinazoline constitute an interesting group of compounds with a wide spectrum of biological activity. Among them are compounds with anticancer activity directed at the tyrosine kinase receptor, such as EGFR (erlotinib),^20^ VEGFR)/EGFR/RET (vandetanib),^21^ and HER2 (tucatinib),^22^ as well as others such as PI3K-δ inhibitor (leniolisib)^23^ and PI3K-α and PI3K-δ inhibitor (copanlisib).^24^ Interestingly, in the group of 2-aminoquinazoline derivatives, a chemotype of serotonin receptor ligands for 5-HT_5A_/5-HT_7_ can be distinguished (G1A-(S)) (Figure 1).^25^ Importantly, selective 5-HT_5A_R antagonists reduce the frequency of tumor sphere-initiating cells in breast cancer cell lines.^26^

Taking into account the potential use of aminoquinazoline derivatives in the treatment of cancer, as well as the unexplained role of serotonin receptors, in particular 5-HT_6_R in the treatment of gliomas, we decided to design and obtain a library of nonselective 5-HT_6_R ligands (with a possible affinity for other serotonin receptors) based on the long-chain 2-aminoquinazoline sulfonamides core and evaluate their anticancer activity on selected cell lines. The mentioned chemotype was supposed to provide simultaneous affinity for 5-HT_6_R and other classes, such as 5-HT_5A_R, 5-HT_1A_R, or 5-HT_7_R. The molecules were designed using molecular modeling methods, starting with docking in the induced fit docking protocol, which accurately predicts ligand binding modes and accompanying structural changes in the receptor.^27,28^ The choice of the method was dictated by the flexible structure of the designed molecules. Further optimization of active conformations was performed using hybrid QM/MM methods to obtain the lowest energy complexes,^28^ and selected conformations optimized in this way were tested for stability by molecular dynamics and analyzed for the contribution of residue–ligand interactions to the total binding energy using the FMO approach.^27,29^ These methods were intended to enable the selection of compounds for synthesis and also to explain some of the structure–activity relationships (SARs), after *in vitro* tests. Observations were supported by a 3D-QSAR approach,^30,31^ which allowed for the assessment of the direction of activity profile changes under the influence of structural modifications. The obtained results were used to build a pharmacophore model, which will allow for further development of this group of ligands.^32^

Based on preliminary docking, two groups of compounds were selected for synthesis, containing an arylsulfonamide fragment or lacking an acyclic arylsulfone. Ligand affinity for 5-HT_6_R was evaluated, in the radioligand binding assay.^33^ The four most active compounds were checked for their selectivity (compared to 5-HT_1A_, 5-HT_5A_, 5-HT_7_, and D_2_) as well as their function was evaluated, by testing the ability to inhibit agonist-induced cAMP biosynthesis. The purpose was to select serotonin receptor antagonists for their potential antiproliferative applications. In the next step, ten most active compounds were tested for their anticancer effect, on 1321N1 astrocytoma as well as U87MG, U-251, U-251, and U-251 glioblastoma lines, MCF7 breast cancer, and AsPC-1 pancreatic cancer lines. Additionally, to test the influence of 5-HT_6_R affinity on antiproliferative activity, six low-affinity ligands were included in the study.

For the selected seven out of the nine most potent 5-HT_6_R ligands, a series of pharmacokinetic and toxicological tests were performed. The selection was made based on *in silico* absorption, distribution, metabolism, excretion, and toxicity (ADMET) parameter predictions.^34^ We started with lipophilicity screening and affinity to phospholipids, as parameters directly influencing absorption, using a biochromatographic approach developed by Valko.^35^ This stage additionally included three compounds with weaker affinity but characterized by good properties determined by *in silico* methods. For seven compounds, membrane permeability was also determined, in the PAMPA test, used to define the potential ability to the blood–brain barrier penetration.^36^ Distribution was assessed by determining the ability of selected compounds to bind to plasma proteins.^37^ Metabolism of ligands was described by their incubation with mouse liver microsomes.^48^ For three ligands with the highest affinity for the 5-HT_6_R receptor and additionally for the compound with the broadest spectrum of antiproliferative activity (**PP 24**), the ability to inhibit the activity of the two most important cytochrome P-450 isoforms was determined. They were also analyzed for *in vitro* hepatotoxicity. Two 5-HT_6_R antagonists with the highest affinity (**PP 10** and **PP 15**) were also evaluated for cardiotoxicity and neurotoxicity *in vivo*.^33^

## 2 Results and Discussion

The aim of this research was to create a new chemotype of long-chain 2-aminoquinazoline sulfonamides as new multifunctional serotonin receptor ligands, focusing on 5-HT_6_R and their antiproliferative activity was determined in glioma cells. The compounds were designed using the virtual screening (VS) method performed on 5-HT_6_ (PDB id: 7XTB) and 5-HT_5A_ (PDB id: 7UM4) receptors. Some of the obtained conformations are presented in the “Molecular Modeling” section.^49^ The molecules consisted of a 3,4-dihydroquinazolin-2-amine part, as a basic moiety, responsible for its strong affinity for serotonin receptors through interaction with D3.32^25^ as well as a sulfonamide fragment, associated with high affinity for the 5-HT_6_ receptor. In further studies, an attempt was made to remove the sulfonamide fragment from the structure. For compounds with the strongest affinity for 5-HT_6_R, an expanded receptor profile was evaluated, including related serotonin receptors like 5-HT_5A_, 5-HT_1A_R, and 5-HT_7_R, as well as the dopamine receptor (D_2_R), along with their respective functions. The selected ligands were also tested for their antiproliferative effect on 1321N1 astrocytoma cells, as well as U-251, U87MG, and LN-229 glioblastoma cell lines. Additionally, the antiproliferative effect was tested on AsPC-1 pancreatic cancer and MCF7 breast cancer cells, assessing the usefulness of the compounds toward glioma-targeted therapy. The selectivity studies for compounds with anticancer activity were conducted on normal human astrocytes (NHA). Several molecules were tested *in vitro* for ADMET, covering aspects like lipophilicity, plasma protein binding, phospholipid affinity, drug–drug interactions (DDI), membrane penetration in PAMPA test, hepatotoxicity and metabolic stability, as well as *in vivo* testing using the *Danio reri*o model. The obtained structure–activity relationship (SAR) was analyzed using molecular modeling techniques.

### 2.1 Chemistry

Based on the conducted VS, we obtained two groups of ligands, the main one consisting of naphthalenesulfonamide alkyl derivatives of 2-aminoquinazoline, and the second included their analogues without an open naphthalenesulfonamide group.

**Scheme 1:**
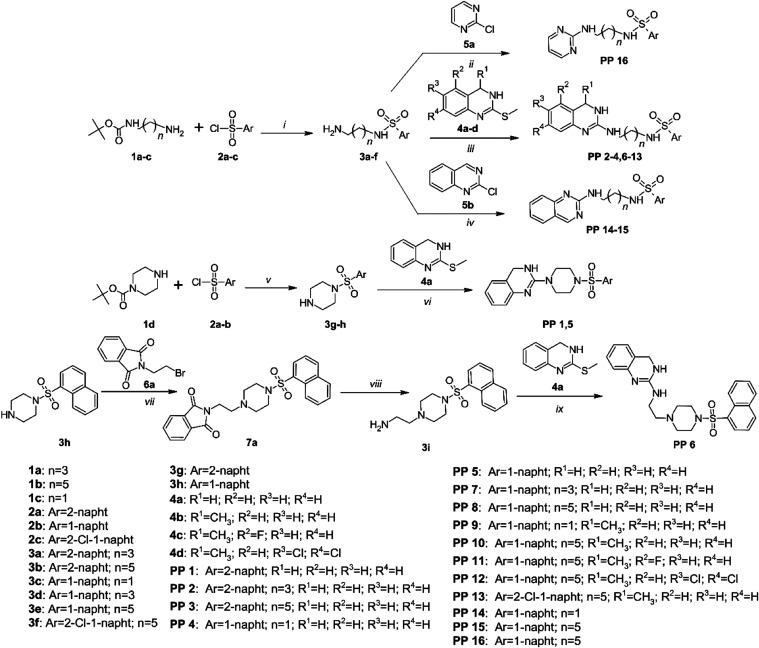
Synthesis of Ligands Belonging to the First Group Reaction and conditions: (i) acetone, TEA, room temperature (RT), 2 h; HCl in H_2_O; (ii) EtOH, K_2_CO_3_, reflux 4 h; (iii) TEA, 180 °C, 1 h; (iv) EtOH, K_2_CO_3_, reflux 4 h; (v) acetone, TEA, RT, 2 h; acetone, HCl in dioxane; (vi) TEA, 180 °C, 1 h; (vii) MeCN, K_2_CO_3_, reflux, 24 h; (viii) CH_3_NH_2_, H_2_O, RT, 8d; (ix) TEA, 180 °C, 1 h.

In the first group, two compounds with piperazine linker were obtained, belonging to 2-[4-(naphthalenesulfonyl)piperazin-1-yl]-3,4-dihydroquinazolines **PP 1,5**. Next, we synthesized molecules with alkyl chains, 11 *N*-{[(3,4-dihydroquinazolin-2-yl)amino]alkyl}naphthalenesulfonamides **PP 2–4,6–13**; two *N*-{[(quinazolin-2-yl)amino]alkyl}naphthalene-1-sulfonamides **PP 14–15** and *N*-{6-[(pyrimidin-2-yl)amino]hexyl}naphthalene-1-sulfonamide **PP 16**. The compounds were obtained by two-step synthesis.^38^ First, mono-BOC-protected diamine, *tert*-butyl(aminoalkyl)carbamates **1a**–**c** or *tert*-butyl piperazine-1-carboxylate **1d** were reacted with naphthalenesulfonyl chlorides **2a**–**c**. The reactions were carried out at room temperature for 2 h in CH_2_Cl_2_/triethylamine (TEA). The obtained products **3a**–**h** were deprotected by heating in 17% HCl. The resulting amines were reacted with 2-(methylsulfanyl)-3,4-dihydroquinazoline derivatives **4a**–**d** by heating at 180 °C in TEA for 1 h or with 2-chloropyrimidine **5a**/2-chloroquinazoline **5b**, in EtOH/K_2_CO_3_ for 4 h. **PP 6** with a hybrid alkyl-piperazine linker was obtained by a 3-step synthesis in which 1-(naphthalene-1-sulfonyl)piperazine **3h** was reacted with 2-(2-bromoethyl)-1*H*-isoindole-1,3(2*H*)-dione **6a** by heating in MeCN/K_2_CO_3_. Product **7a** was subjected to the Gabriel reaction with methylamine, obtaining amine **3i**, which in the next step was reacted with 2-(methylsulfanyl)-3,4-dihydroquinazoline **4a** by heating at 180 °C in TEA for 1 h (Scheme 1).

**Scheme 2:**
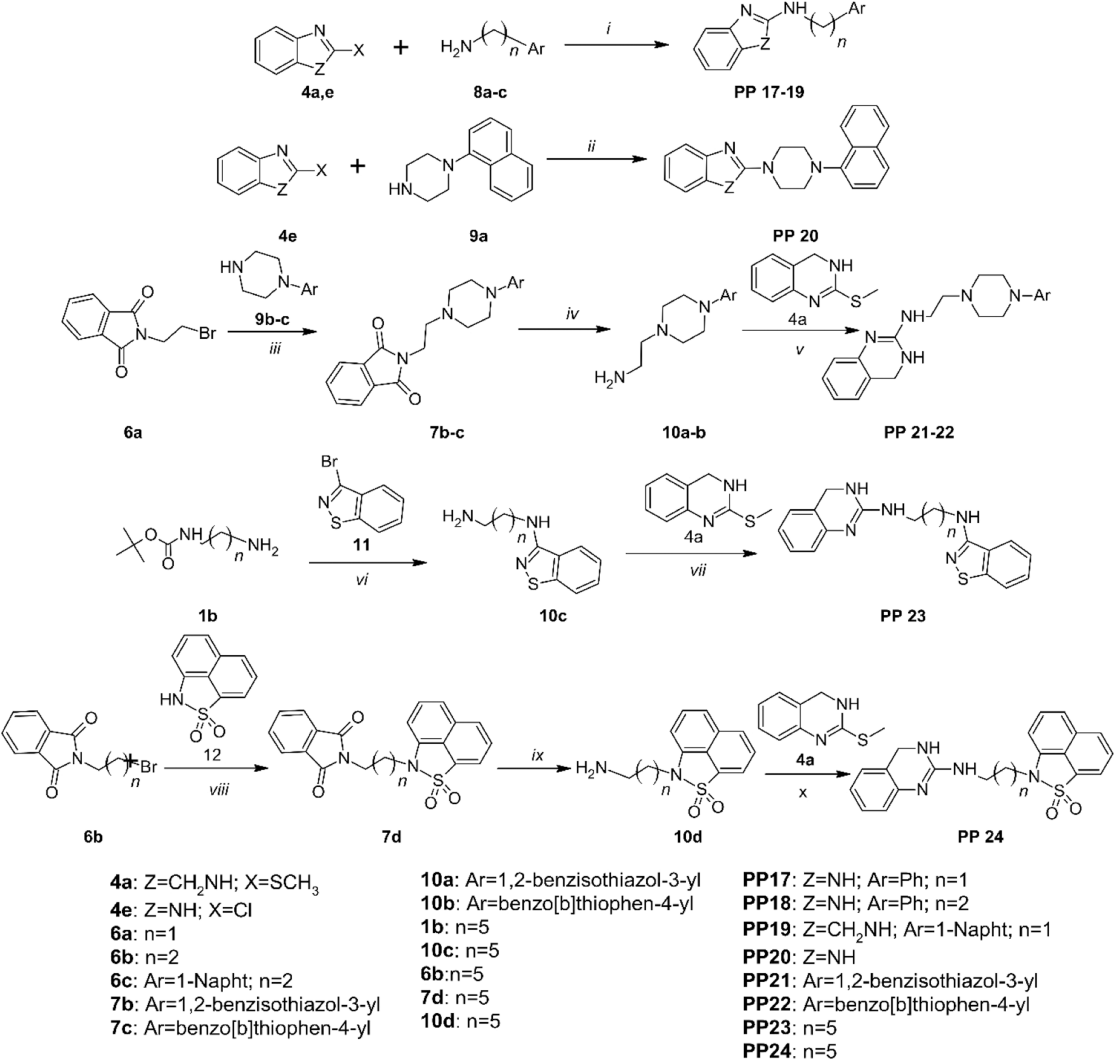
Synthesis of Ligands Belonging to the Second Group Reaction and conditions: (i) EtOH, K_2_CO_3_, reflux 4 h; (ii) EtOH, K_2_CO_3_, reflux 4 h; (iii) MeCN, K_2_CO_3_, reflux, 24 h; (iv) CH_3_NH_2_, H_2_O, RT, 8d; (v) TEA, 180 °C, 1 h; (vi) MeCN, K_2_CO_3_, reflux, 24 h; (vii) TEA, 180 °C, 1 h; (viii) MeCN, K_2_CO_3_, reflux, 24 h; (ix) CH_3_NH_2_, H_2_O, RT, 8d; (x) TEA, 180 °C.

In the second set, two *N*-(phenylalkyl)-1*H*-1,3-benzimidazol-2-amines **PP 17–18** and 2-[4-(naphthalen-1-yl)piperazin-1-yl]-1*H*-1,3-benzimidazole **PP 20** were synthesized. The compounds were obtained by reacting the appropriate amine **8a**–**b** or piperazine **9a** with 2-chloro-1*H*-1,3-benzimidazole **4e** in EtOH/K_2_CO_3_. **PP 19**, *N*-[2-(naphthalen-1-yl)ethyl]-3,4-dihydroquinazolin-2-amine, was synthesized by reacting amine **8c** with 2-(methylsulfanyl)-3,4-dihydroquinazoline **4a** by heating at 180 °C in TEA for 3 h. *N*-[2-(4-Arylpiperazin-1-yl)ethyl]-3,4-dihydroquinazolin-2-amines **PP 21–22** were obtained in a 3-steps, by reaction of arylpiperazines **9b**–**c** with 2-(2-bromoethyl)-1*H*-isoindole-1,3(2*H*)-dione **6a** in MeCN/K_2_CO_3_. The resulting products **7b**–**c** were subjected to the Gabriel reaction with methylamine, obtaining amines **10a**–**b**, which in the next step were reacted with 2-(methylsulfanyl)-3,4-dihydroquinazoline **4a**. *N*^6^-(1,2-Benzothiazol-3-yl)-*N*^1^-(3,4-dihydroquinazolin-2-yl)hexane-1,6-diamine **PP 23** was obtained in a 2-step synthesis in which *tert*-butyl(6-aminohexyl)carbamate **1b** was reacted with 3-bromo-1,2-benzothiazole **11** in MeCN/K_2_CO_3_. After deprotection in 17% HCl, the resulting amine **10c** was reacted with 2-(methylsulfanyl)-3,4-dihydroquinazoline **4a**. 3-{6-[(3,4-Dihydroquinazolin-2-yl)amino]hexyl}-2λ^6^-thia-3-azatricyclo[6.3.1.0^4^,^12^]dodeca-1(11),4(12),5,7,9-pentaene-2,2-dione **PP 24** was obtained analogously to **PP 21–22** by reacting 2-(6-bromohexyl)-1*H*-isoindole-1,3(2*H*)-dione **6b** with 1λ^6^-naphtho[1,8-cd][1,2]thiazole-1,1(2*H*)-dione **12**. The resulting intermediate **7d** was subjected to the Gabriel reaction, obtaining amine **10d**, which in the next step was reacted with 2-(methylsulfanyl)-3,4-dihydroquinazoline (Scheme 2).

### 2.2 Radioligand Binding and SAR Study

Ligand affinity for 5-HT_6_ receptors was subsequently tested with a radioligand binding assay, using the HEK293 cell line with human 5-HT_6_R, aiming to select compounds with the strongest affinity. Three compounds belonging to 2-naphthalenesulfonamide derivatives of 3,4-dihydroquinazolin-2-amines were obtained (Table 1).

**Table 1:**
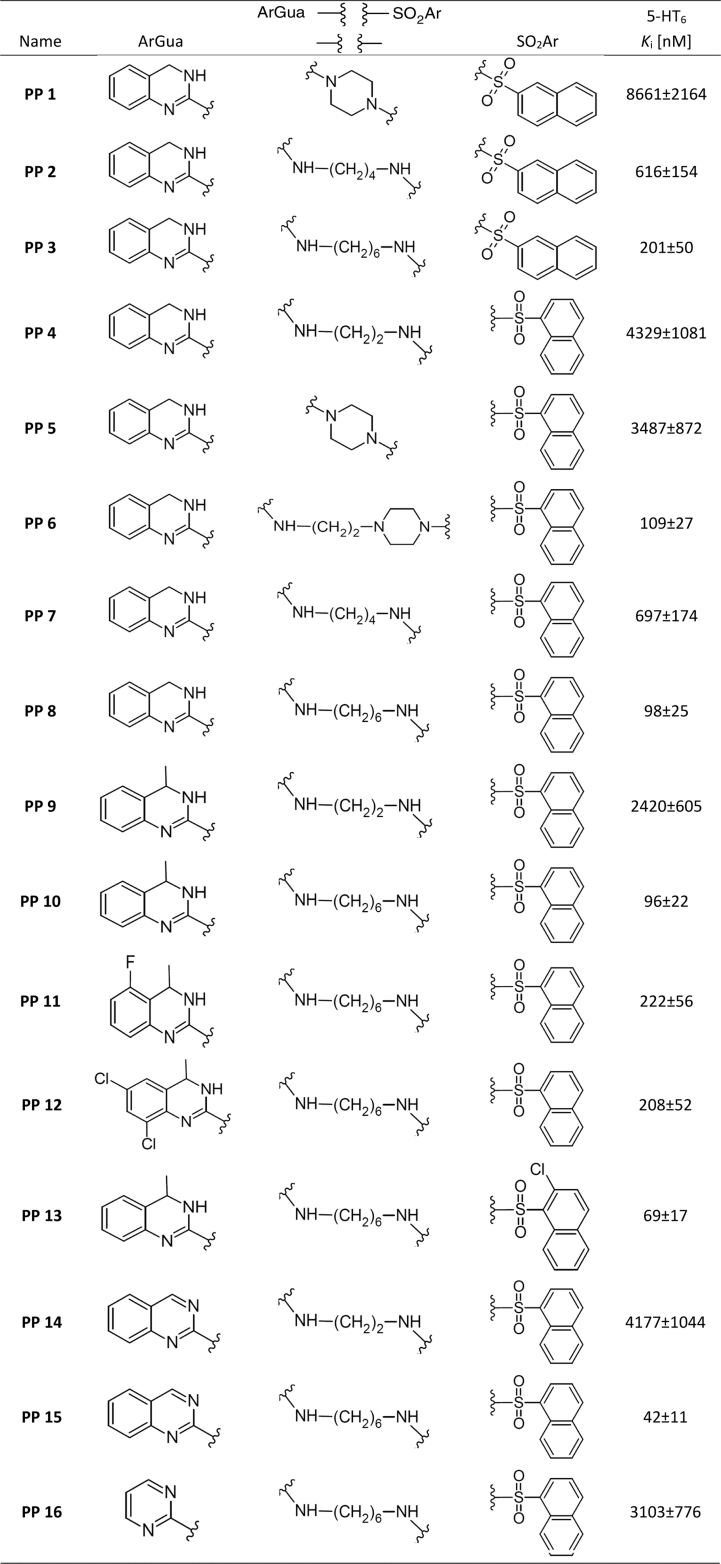
Radioligand Binding Data for 5-HT_6_ Receptors

Ligand **PP 1** with a piperazine linker was characterized by relatively low affinity (*K*_i_ = 8661 nM). Opening of the linker and its extension to butyl in **PP 2** or hexyl in **PP 3** resulted in a decrease of the *K*_i_ constant. A similar effect was observed with 1-naphthalenesulfonamide analogues **PP 4–8**. The most beneficial was the use of a hexyl linker in **PP 8** (*K*_i_ = 98 nM). Favorable results was also obtained for **PP 6**, in which a combined linker consisting of piperazine, and an ethyl chain was used (*K*_i_ = 109 nM). However, it should be noted that this compound has an additional, strongly basic nitrogen atom at piperazine. A group of 4-methylated analogues of **PP 8** were also obtained. Unsubstituted **PP 10** (*K*_i_ = 96 nM) had a very similar affinity compared to **PP 8**. Substitution of the quinazoline ring increased *K*_i_ constants. An opposite effect was observed for **PP 13**, which had chlorine as a substituent in the 1-naphthalenesulfonamide group. Even better results were obtained for the hexyl derivative of quinazoline-2-amine, for which the affinity constant was 42 nM.

In the second set, mainly derivatives of cyclic arylguanidines with an aryl or aminoaryl moiety in the terminal part, instead of an arylsulfonamide were obtained, to check the possibility of eliminating this core (Table 2).

**Table 2:**
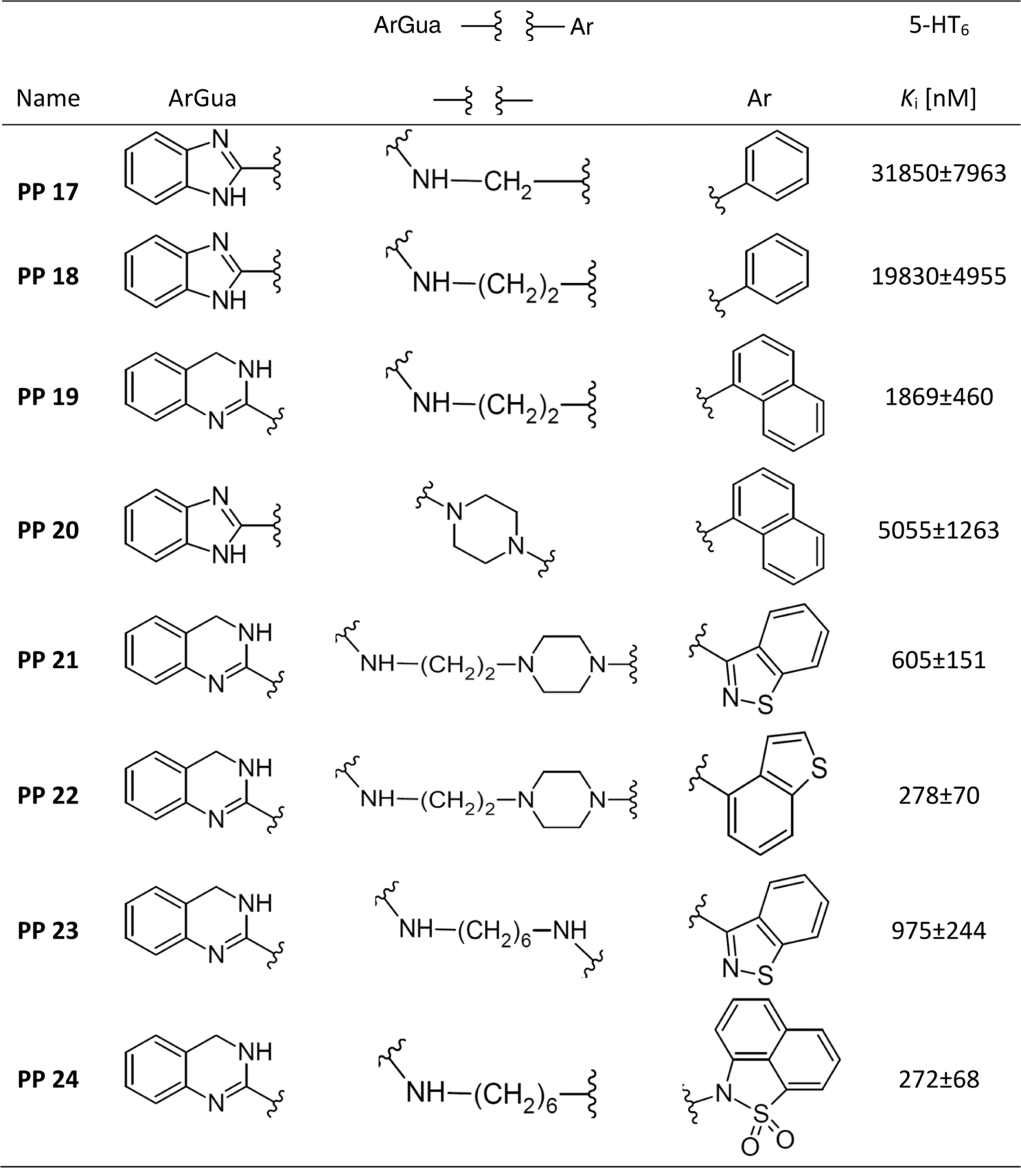
Radioligand Binding Data for 5-HT_6_ Receptors

In this group, three simple derivatives of 1,3-benzimidazol-2-amines **PP 17, 18**, and **20** were obtained, however, these compounds did not show any affinity toward the tested receptor. Replacing the 1,3-benzimidazol-2-amine part with 2-amino-3,4-dihydroquinazoline in **PP 19** resulted in a decrease in the *K*_i_ constant value. Next, two nonsulfonamide analogues of the **PP 6** compound were obtained, in which a hybrid alkyl-piperazine linker and a benzothiazole **PP 21** or benzothiophene **PP 22** terminal aromatic group were used. These compounds had moderate affinity for 5-HT_6_R, lower than the sulfonamide analogue. Importantly, opening the linker by using a hexyl chain also did not improve the affinity of **PP 23**. The exception in this group is **PP 24**. This ligand has a cyclic sulfonamide moiety (1,8-naphthosultam) in its terminal part. It can therefore be considered a direct analogue of **PP 8**, having a cyclic sulfonamide moiety in the terminal part. Interestingly, a decrease in affinity was observed for **PP 26** (*K*_i_ = 272) compared to **PP 8**, but not completely losing.

The selectivity of the four most active compounds was tested against 5-HT_1A_, 5-HT_5A_, 5-HT_7_ serotonin receptors, and dopamine D_2_R. Their functional activity was also evaluated by assessing their ability to inhibit agonist-induced cAMP production in HEK293 cells (Table 3). Extended radioisotope tests were aimed at detecting potential affinity for other aminergic receptors, whose role in cancer treatment may be important. Functions were tested for all receptors. The essence of functional tests results from the fact that serotonin stimulates tumor progression. Therefore, the ligand should have the potential to reduce its concentration, i.e., act as an antagonist.^39^

**Table 3:** Extended Receptor Affinity and Functional Assays^a^

	D_2_			5-HT_1A_			5-HT_5A_	5-HT_6_			5-HT_7_			HEK293
name	*K*_i_	EC_50_	*K*_B_	*K*_i_	EC_50_	*K*_B_	*K*_i_	*K*_i_	EC_50_	*K*_B_	*K*_i_	EC_50_	K_B_	IC_50_ [μM]
**PP 6**	7430	NA	NA	42	375	NA	328	109	367	NA	2399	NA	NA	51.0
**PP 10**	2932	NA	1814	165	NA	74	59	96	NA	36	189	NA	236	NT
**PP 13**	1903	NA	858	19	1535	NA	ND	69	488	NA	198	53	NA	NT
**PP 15**	71,770	NA	NA	7155	NA	NA	1093	42	NA	48	1563	NA	NA	NT

a Not active (NA). Not determined (ND). Nontoxic in 50 μM (NT).

None of the tested compounds showed high affinity for the D_2_R. **PP 6** and **PP 13** showed a similar receptor profile, having high affinity for the 5-HT_1A_R and 5-HT_6_R and the tests performed indicated their agonist function in the receptor. Moreover, **PP 13** was probably also a 5-HT_7_ agonist. **PP 10** was characterized by a moderate 5-HT_1A_/5-HT_5A_/5-HT_6_/5-HT_7_ antagonist profile. **PP 15**, a 5-HT_6_ receptor antagonist, turned out to be the most selective. Due to its potential function as an agonist, the viability of HEK293 cells treated with the test compounds was examined. No cytotoxic effects of the compounds were detected in the tested concentration range.

### 2.3 Antiproliferative Activity

The chosen group of ligands was further examined for their ability to inhibit proliferation across multiple cancer cell lines related to the CNS system (1321N1, U87MG, LN-229, and U-251). These cell lines were characterized by different mutation profiles, the main one being the variable status of the p53 protein. In detail, U87MG cells have a wild-type p53 protein, while astrocytoma and the rest of GBM cells harbor mutated p53 protein. Additional assays were performed on MCF7 breast cancer and AsPC-1 pancreatic cancer cells. The wide range of tested lines was aimed at screening the anticancer potential of the tested compounds. The first represents one of the most common and the second one of the most aggressive cancers in the world. Comparing the activity of compounds on GBM cells to other types of cancer may reveal their selectivity and usefulness in targeted therapies. The cytotoxicity of the compounds on normal human astrocytes (NHA) and *Feline catus* astrocyte (PG-4) was determined. Tests on normal lines serve to verify the potential toxicity of the tested compounds on healthy cells. Due to the targeting of CNS tumors, astrocytes were selected as normal lines. The IC_50_ values are presented in Table 4.

**Table 4:** Antiproliferative Activity of Selected Compounds against CNS Tumors, Breast Cancer, and Pancreatic Cancer, as well as Normal Astrocytes^a^

	IC_50_ [μM]							
name	1321N1	U87MG	LN-229	U-251	MCF7	AsPC-1	NHA	PG-4
**PP 3**	**13.3 ± 0.1**	**12.0 ± 0.9**	ND	ND	ND	ND	ND	ND
**PP 4**	>25	>25	>25	>25	>25	>25	ND	ND
**PP 6**	**20.9 ± 1.4**	>25	>25	>25	>25	>25	ND	ND
**PP 8**	**18.4 ± 2.9**	**24.5 ± 1.4**	>25	>25	>25	>25	ND	ND
**PP 10**	**13.4 ± 0.8**	**20.7 ± 1.2**	>25	>25	>25	>25	**12.6 ± 0.4**	>25
**PP 11**	**5.7 ± 0.1**	**8.5 ± 0.7**	>25	**12.2 ± 0.3**	**9.1 ± 0.8**	**13.8 ± 0.3**	**4.4 ± 1.3**	ND
**PP 12**	**5.7 ± 0.1**	**9.5 ± 0.7**	ND	ND	ND	ND	ND	ND
**PP 13**	**9.6 ± 1.8**	**13.6 ± 1.1**	>25	>25	**19.3 ± 0.3**	**14.6 ± 0.5**	**4.2 ± 0.4**	ND
**PP 14**	>25	>25	>25	>25	>25	>25	ND	ND
**PP 15**	>25	>25	>25	>25	>25	>25	ND	>25
**PP 16**	ND	ND	>25	>25	>25	>25	ND	ND
**PP 19**	ND	ND	>25	>25	>25	>25	ND	ND
**PP 20**	ND	ND	>25	>25	>25	>25	ND	ND
**PP 21**	ND	ND	**9.9 ± 0.7**	**13.0 ± 0.4**	>25	**7.4 ± 0.5**	**4.7 ± 0.2**	ND
**PP 22**	**10.4 ± 0.6**	**13.8 ± 0.9**	>25	**4.3 ± 0.6**	**14.5 ± 0.2**	**12.5 ± 0.4**	**8.1 ± 0.2**	ND
**PP 24**	**8.4 ± 1.9**	**13.6 ± 0.6**	**18.4 ± 1.1**	**16.5 ± 1.0**	**15.7 ± 1.6**	**12.1 ± 1.0**	**7.9 ± 0.4**	ND
**Doxorubicin**	1.1	ND	ND	ND	ND	ND	ND	ND
**Gefitinib**	ND	ND	>25	24.0	>25	ND	ND	ND
**Afatinib**	ND	ND	9.8	27.0	25.9	ND	7.5	ND

a Not determined (ND).

For some compounds, relatively low IC_50_ values (<10 μM) were obtained, indicating often stronger antiproliferative activity than commercially available drugs. In the case of the U-251 line, the IC_50_ value was 4.3 μM for **PP 22**, comparable to Gefitinib (IC_50_ = 24.0 μM, Afatinib IC_50_ = 27.0 μM). Similar values were obtained for other low-molecular-weight kinase inhibitors, e.g., p38 MAPK inhibitor IC_50_ = 24.61 μM;^40^ for MDM2-CDK4 dual inhibitor IC_50_ = 8.6 μM.^41^ In general, we did not observe a homogeneous pattern of antiproliferative activity of the tested compounds against CNS cancer cell lines. Nevertheless, it seems that 1321N1 and U87MG cells are the most susceptible to the first group of tested 2-naphthalenesulfonamide derivatives. Among them, **PP 11** and **PP 12** exhibited high antiproliferative potential against astrocytoma. For both derivatives, the calculated IC_50_ value was 5.7 μM. In the case of U87MG, the IC_50_ values were 8.5 and 9.5 μM, respectively. It is worth noting that the extended evaluation showed that **PP 11** also largely inhibited the proliferation of breast cancer cells (IC_50_ = 9.1 μM) and normal human astrocytes (IC_50_ = 4.5 μM). Thus, these results clearly indicated the lack of selectivity of this compound. **PP 3**, **PP 6**, **PP 8**, **PP 10**, and **PP 13** showed moderate (IC_50_ < 17 μM) or weak (IC_50_ = 17–25 μM) antiproliferative activity against 1321N1 and U87MG. Interestingly, most of them were nonactive (IC_50_ > 25 μM) in an expanded panel of cancer cells (LN-229, U-251, MCF7, AsPC-1). The one exception was **PP 13**, which was capable of inhibiting breast and pancreatic cells. Unfortunately, **PP 10** and **PP 13** were also cytotoxic on NHA cells. Moreover, our analysis indicated that **PP 4** and **PP 14** derivatives with an ethyl carbon linker did not show antiproliferative activity on any of the tested cell lines. It can relate to too weak affinity of compounds with short alkyl chains for serotonin receptors. This hypothesis is supported by the results of affinity for the 5-HT_6_ receptor. Moreover, the selective 5-HT_6_R ligand **PP 15**, belonging to quinazoline derivatives, showed no cytotoxic effect, which may indicate the insignificant role of the 5-HT_6_R receptor in cytotoxic activity.

Among the second analyzed group of arylguanidines with an aryl or an aminoaryl moiety in the terminal part, only three compounds exhibited high antiproliferative activity on cancer cell lines. Namely, **PP 21**, **PP 22**, and **PP 24** contained a benzothiazole, benzothiophene, and 1,8-naphthalenesultam group in the terminal part, respectively. It should be noted that **PP 22** was the most potent and highly selective derivative against one of the GBM cell lines harboring the p53 mutation. Indeed, it significantly inhibited the proliferating U-251 cells (IC_50_ = 4.3 μM). Comparatively, the antiproliferative potential of **PP 22** against other tested cancer cell lines was lower (IC_50_ ranging from 10.3 to 14.5 μM). This derivative was also nonactive on LN-229 cells. In the context of the usefulness of this compound in GBM-targeted therapy, it is crucial to determine its effect on normal astrocyte cells. Hence, a 2-fold weaker inhibitory effect on NHA proliferation after exposure to **PP 22** was noted. The calculated selectivity index as the ratio of the antiproliferative effect on NHA cells to U-251 cells was almost 2. Therefore, due to its quite good affinity to 5-HT_6_R (*K*_i_ = 278) as well as its high and selective antiproliferative potential, **PP 22** may be an interesting and promising candidate for the treatment of GBM with the common p53 R273H hot spot mutation. In the case of **PP 21** and **PP 24**, both compounds were nonselective against cancer cell lines, although they showed an interesting profile of antiproliferative activity (IC_50_ ranging from 7.4 to 18.3 μM). However, they also showed a high cytotoxic effect against normal astrocytes (the selectivity index was below 1). In summary, it can be concluded that within the tested group of compounds, there is no clear evidence of a connection between high affinity to serotonin receptors and high antiproliferative activity in the *in vitro* glioblastoma cells model.

### 2.4 ADMET Test

For compounds with the highest affinity for the 5-HT_6_R, we decided to examine the ADMET profile. Starting from absorption, we assessed the lipophilicity and affinity for phospholipids of selected compounds. We also examined the ability to potentially blood–brain barrier penetration in the PAMPA test, due to the targeting of CNS tumors.

Lipophilicity and affinity for phospholipids for each investigated molecule were assessed. Additionally, selected compounds with the highest biological activities have been characterized by their affinity plasma proteins. All experiments were performed using protocols developed by Valko.^35^ These methods facilitated the calculation of chromatographic hydrophobicity indices (CHI) and plasma protein binding percentages (%PPB) in a biochromatographic experiment, with results compared to the reference. A detailed description of the experiments is provided in the Supporting Information (SI), along with the raw data. The results revealed that the analyzed molecules possess relatively high lipophilicity, with some exceeding a value of 100. Although high lipophilicity may limit the medical use of the analyzed compounds since they may tend to accumulate excessively in the human body, it is remarkable that several clinically used drugs also have high lipophilicity. Analyzing CHI values across varying pH levels provides a rapid evaluation of acid–base characteristics, revealing that the studied substances predominantly exhibit basic properties. (Table 5). Overall, the target molecules showed a strong affinity for phospholipids, similar to their lipophilicity.

**Table 5:** Parameters Determined by the GlaxoSmithKline (Valko) Protocol, including Lipophilicity, Phospholipid Affinity, and Plasma Protein Binding

	CHI C18	CHI C18	CHI C18			
name	pH 2.6	pH 7.4	pH 10.5	CHI IAM	logKHSA	%HSA
**PP 4**	64.9	79.9	101.9	50.2	1.2	94.8
**PP 6**	92.9	101.5	138.2	50.9	1.4	97.2
**PP 10**	89.8	99.7	120.4	56.4	1.5	97.7
**PP 11**	103.4	103.1	93.1	60.1	1.8	99.2
**PP 12**	97.1	111.2	121.8	59.4	1.9	99.7
**PP 13**	115.8	105.7	128.8	59.9		>99.8
**PP 14**	78.3	86.3	87.1	37.2	1.2	95.0
**PP 15**	99	104.4	107.2	45.8	1.5	97.8
**PP 22**	56.4	115.8	168.7	56.4	1.6	98.4
**PP 24**	84.7	103.2	127	56.5	1.6	98.8

One of the most frequently used methods for checking the permeability of compounds is testing their passive penetration through artificial double-layer membranes. The permeability of four selected compounds was tested in PAMPA. Ligands have a poor permeability coefficient, lower than the breakpoint for permeable molecules (1.5 × 10^–6^).^36^ This fact may indicate the lack of penetration of the blood–brain barrier, which may be due to the high lipophilicity of the compounds resulting in high membrane retention. However, there are methods of delivering drugs that do not penetrate the blood–brain barrier, such as the use of nanocarriers,^42^ osmotic, ultrasonic, or magnetic opening of the barrier,^43^ the use of additional transporters,^44^ or intranasal administration.^45^

In the next step, we assessed the parameters related to the distribution of the drug. Plasma protein binding (PPB) should be investigated early in the drug discovery process because only the unbound fraction (fu) that can cross cell membranes exerts its pharmacological effect. Many drugs, particularly lipophilic compounds, bind to circulating PPB, including α1-acid glycoprotein (AGP) and human serum albumin (HSA).^37^ Columns modified with plasma proteins like HSA enable the efficient evaluation of plasma protein binding (PPB). Even though the target compounds are characterized by a high % binding of HSA, which predominates in PP (plasma protein), these results are still acceptable because several substances used in clinical practice can bind up to approximately 99%.^46^ The interaction with plasma proteins was additionally evaluated in the study using the commercial TRANSIL^XL^ PPB test. The test simulated physiological plasma conditions (HSA and AGP in a ratio 24:1). Warfarin, known for its high affinity for plasma proteins, was utilized as a positive control. (Table 6). The test confirmed the high ability to bind plasma proteins.

**Table 6:** Pharmacokinetic Properties for Selected Compounds^a^

	PAMPA	metabolic stability		PPB	
name	Pe* (10^–6^ cm/s)	% of remaining	main metabolic pathway	kD [μM]	fb [%] ± SD
**PP 6**	ND	72.76	hydroxylation	ND	ND
			double hydroxylation and double bond reduction		
**PP 10**	0.03	66.60	double hydroxylation and double bond reduction or double hydroxylation and ring opening	2.1	99.7 ± 0.16
**PP 11**	ND	74.22	hydroxylation	ND	ND
			double hydroxylation and double bond reduction		
**PP 12**	ND	76.87	decomposition/oxidation	ND	ND
			decomposition/ring opening		
**PP 13**	0.19	52.60	ring opening and triple hydroxylation	0.4	99.9 ± 0.03
**PP 15**	0.13	48.07	hydroxylation	0.2	100.0 ± 0.06
**PP 24**	0.73	93.13	hydroxylation	2.7	99.6 ± 0.13

a Dissociation constant (kD), fraction bound (fb). References: Verapamil (remaining 23.93%); Caffeine (Pe = 8.23 × 10^–6^ cm/s); Warfarin (kD = 9.5 [μM], fb = 98.5).

Moving on to the next parameter, which is metabolism, we determined metabolic stability, using mouse liver microsomes (MLMs). After 120 min of incubation with MLMs, all ligands were metabolized in less than 52%. In the case of **PP 10**, 14 potential metabolites were identified, defining double hydroxylation and double bond reduction or double hydroxylation and ring opening as the main pathway. A similar metabolic route was determined for **PP 13**, identifying 11 metabolites. Substituting the quinazoline ring with halogen atoms in the aromatic ring resulted in a significant increase in stability (**PP 11**, **PP 12**). For **PP 15**, the metabolic stability turned out to be the lowest, and hydroxylation was dominant, as in the case of **PP 24**. Interestingly, the last compound showed unexpectedly high metabolic stability, remaining in the unmetabolized form at 93.13%. The MetaSite 6.0.1^47^ analysis predicts that the quinazoline ring of **PP 24** is most prone to hydroxylation at positions *7-*. This fact may be due to the presence of a cyclic arylsulfonamide moiety in the structure of **PP 24**, which may complicate its metabolism.

**Figure 2:**
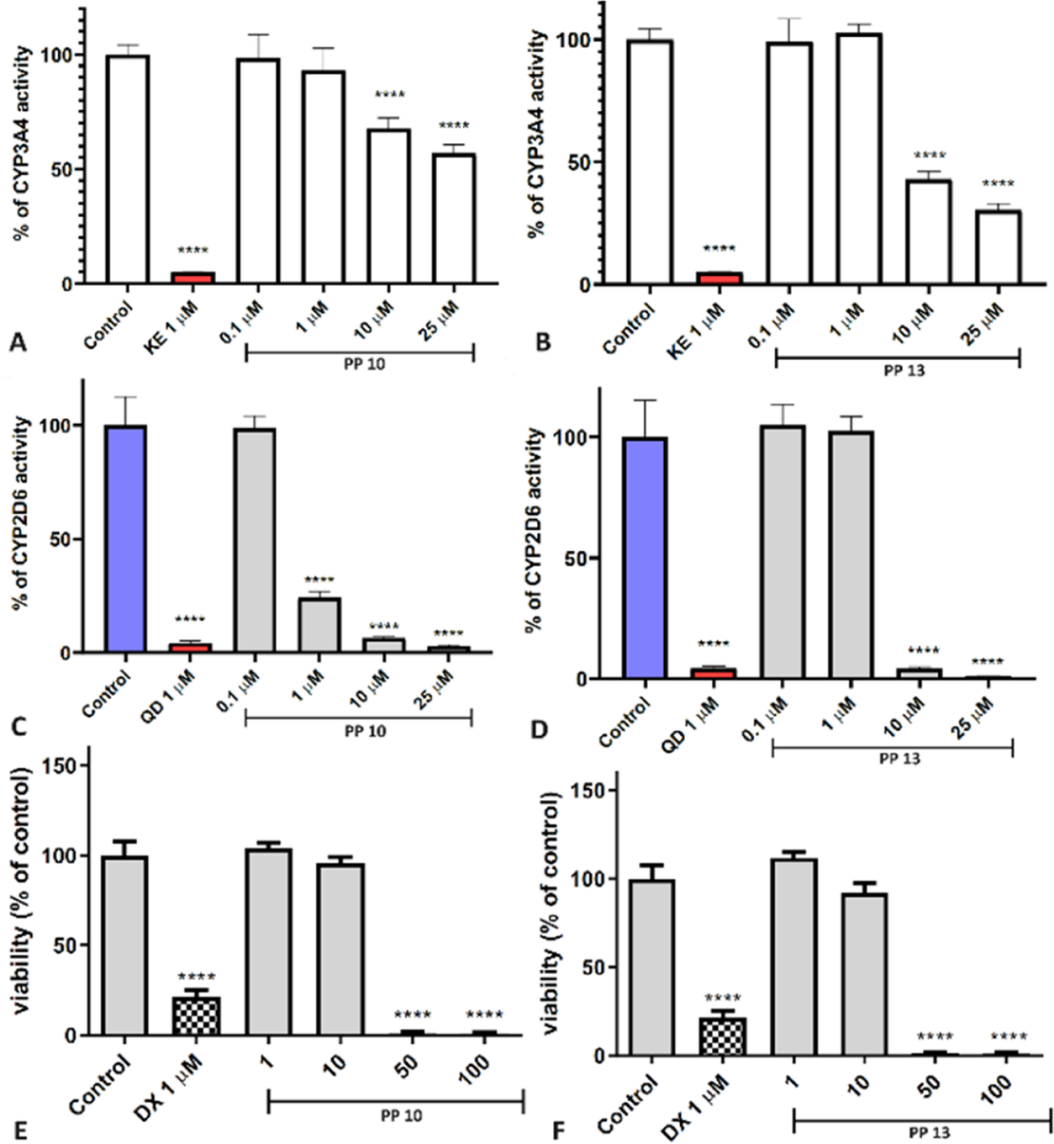
Impact of A, C: **PP 10** and B,D: **PP 13** on the activity of CYP3A4 (A, B) and CYP2D6 (C, D). Statistical significance (****p < 0.0001), with ketoconazole (KE) and quinidine (QD) serving as the reference inhibitors. The effect of doxorubicin (DX) and substances E: **PP 10** and F: **PP 13** on the viability of the HepG2 hepatoma cell line after 48 h of incubation. Statistical significance was assessed using GraphPad Prism 8.0.1, employing one-way analysis of variance (ANOVA) followed by Bonferroni’s post hoc test (****p < 0.0001, compared to the negative control treated with 1% dimethyl sulfoxide (DMSO) in growth media).

**Figure 3:**
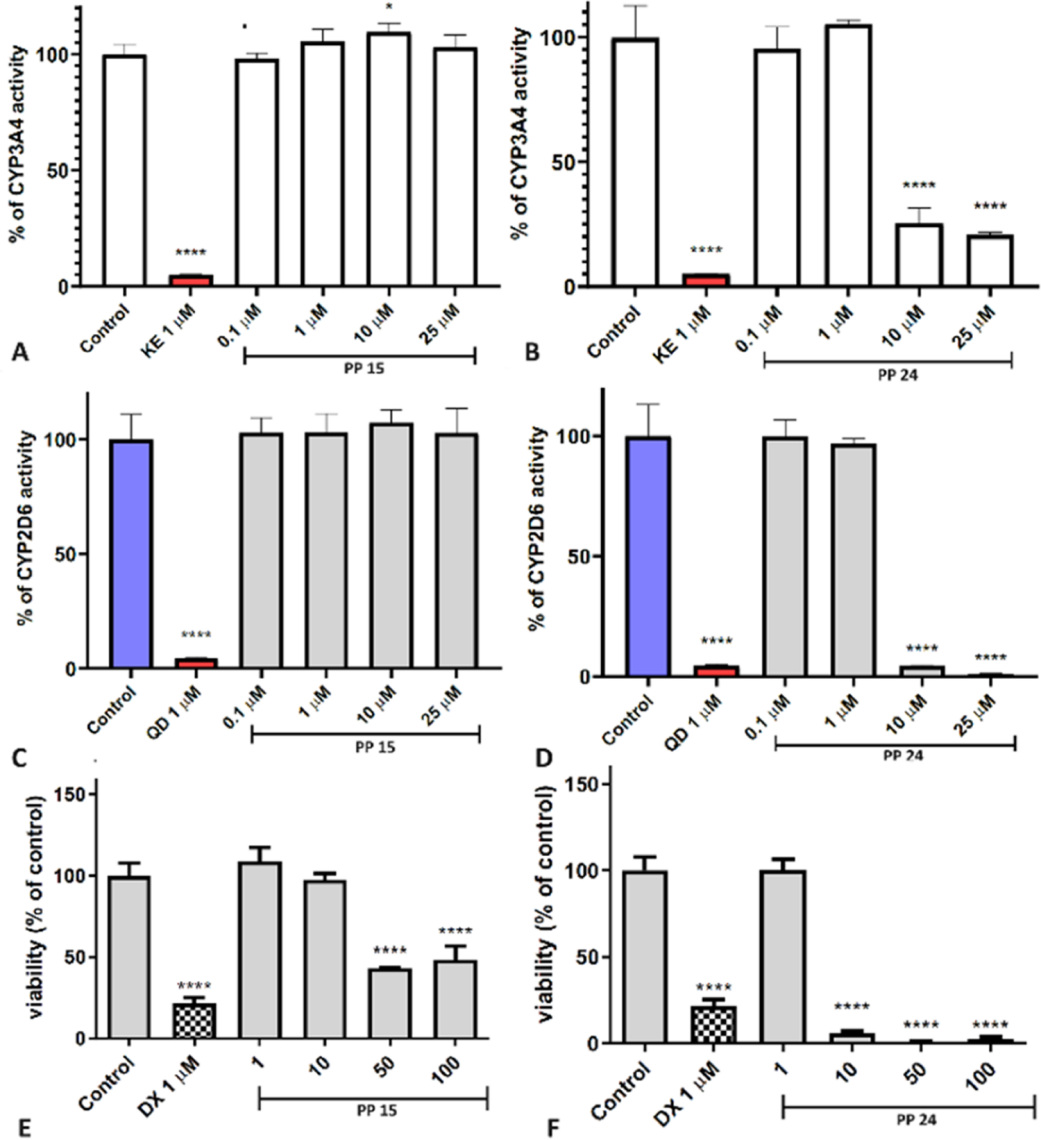
Impact of A,C: **PP 15** and B,D: **PP 24** on the activity of CYP3A4 (A, B) and CYP2D6 (C, D). Statistical significance (****p < 0.0001), with ketoconazole (KE) and quinidine (QD) serving as the reference inhibitors. The effect of doxorubicin (DX) and substances E: **PP 15** and F: **PP 24** on the viability of the HepG2 hepatoma cell line after 48 h of incubation. Statistical significance was assessed using GraphPad Prism 8.0.1, employing one-way ANOVA followed by Bonferroni’s post hoc test (****p < 0.0001, compared to the negative control treated with 1% DMSO in growth media).

In order to evaluate excretion, we examined the effect on the activity of two cytochrome P-450 isoforms. Inhibition of their activity may lead to the occurrence of drug–drug interactions. The influence of lead derivatives on two of the cytochrome P-450 isoforms CYP3A4 and CYP2D6, most involved in drug metabolism, was investigated in P450-Glo assays (Promega) (Figures 2A–D, 3A–D).

The conducted research allowed us to conclude that 3,4-dihydroquinazoline derivatives may exhibit drug–drug interactions, inhibiting the activity of both isoforms, particularly CYP2D6 at concentrations above 10 μM. **PP 10** exhibited a strong effect at a concentration of 1 μM. The impact of the tested compounds on CYP3A4 was slightly lower. Conversely, **PP 10** showed the weakest interaction with this isoform. Interestingly, **PP 15** turned out to be passive toward both isoforms tested.

As a next step, we decided to investigate the potential toxicity of our lead compounds by assessing their toxic effects on the liver (*in vitro*), heart, and brain (*in vivo*). The safety profile was assessed through a hepatotoxicity assay using HepG2 hepatoma cells. (Figures 2E,F, 3E,F). The compounds **PP 10**, **PP 13**, and **PP 15** showed no hepatotoxic effects at concentrations of 1–10 μM. A statistically significant decrease in cell viability was observed at the concentration of 50 or 100 μM. In the case of **PP 24**, a strong cytotoxic effect on HepG2 was noted at a concentration of 10 μM, which corresponds to the data presented in Table 4, emphasizing the nonselective cytotoxic activity of **PP 24**.

**Figure 4:**
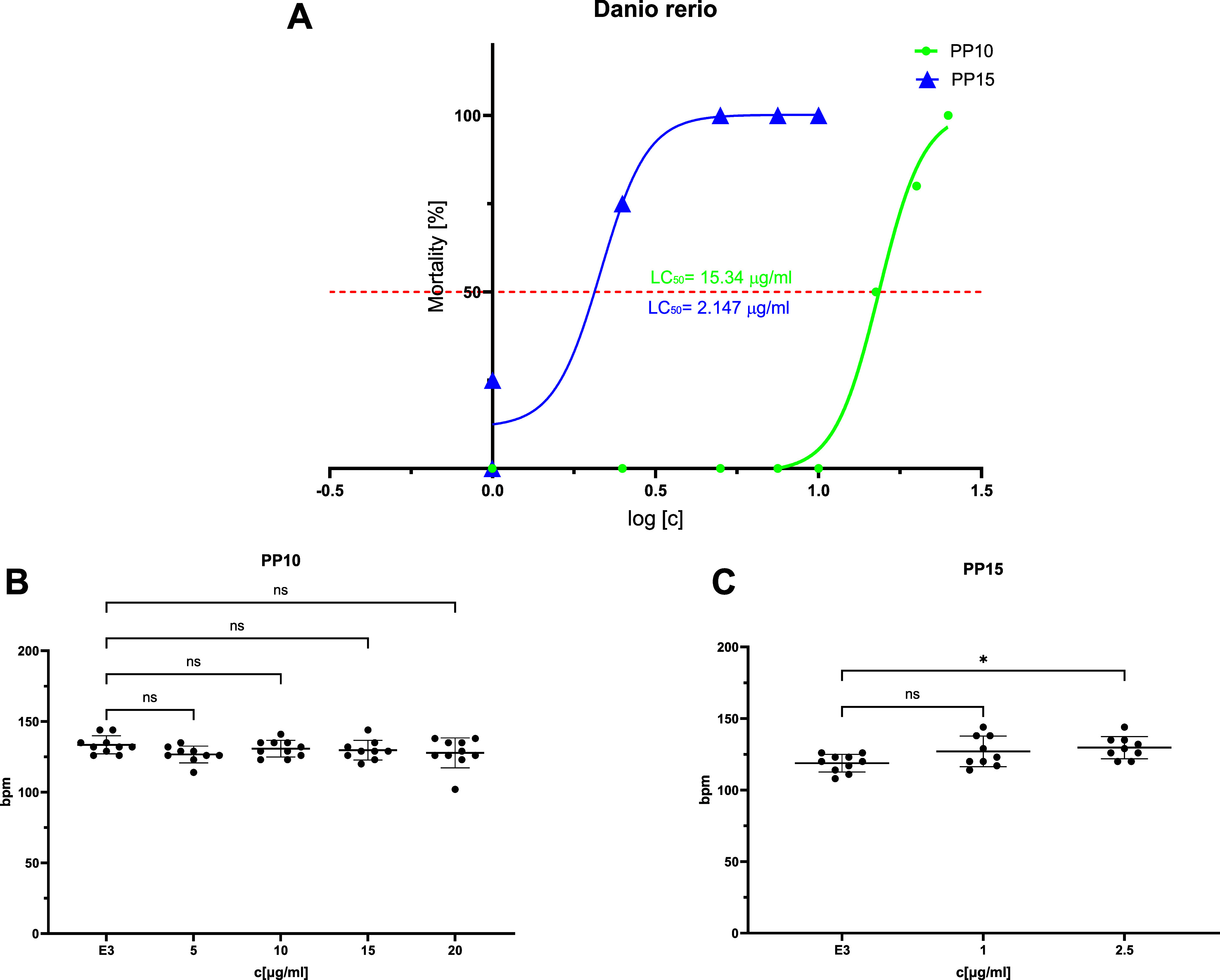
A: Toxicity of **PP 10** and **PP 15***in vivo* in the *Danio reiro* embryo toxicity test. The LC_50_ values were determined after 96 h of exposure. Cardiotoxicity of compounds B: **PP 10** and C: **PP 15** was evaluated by measuring the heart rate (beats per minute) in 5-dpf *Danio rerio* larvae, maintained in the E3 fish embryo growth medium. Statistical analysis was performed using GraphPad Prism 10.0.0, with one-way ANOVA followed by Dunnett’s comparison test (*p < 0.05, compared to the negative control E3).

**Figure 5:**
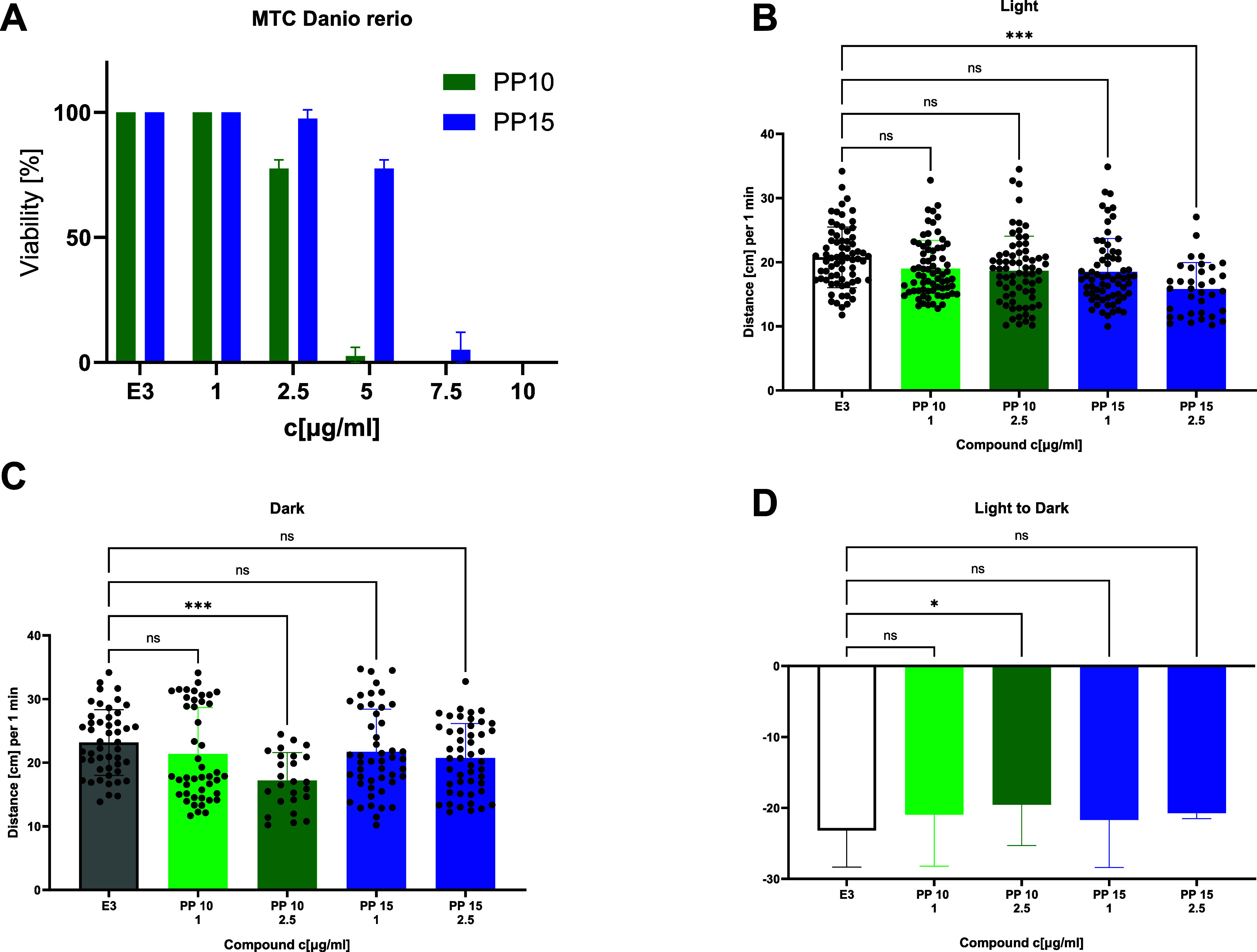
A: *In vivo* toxicity assessment of **PP 10** and **PP 15** in *Danio rerio* using the maximum tolerated concentration (MTC) toxicity test. B: Locomotor activity under light conditions, quantified as the distance (in cm) traveled per minute by 5-day postfertilization (dpf) *Danio rerio* larvae. C: Locomotor activity under dark conditions, with the distance (in cm) traveled per minute recorded for 5-dpf larvae. D: Comparison of activity during the transition from light to dark conditions. E3 refers to the fish embryo growth medium. Statistical analysis was performed using GraphPad Prism 10.0.0, with one-way ANOVA followed by Tukey’s post hoc test: *p < 0.05, ***p < 0.001, compared to the negative control (E3).

Two compounds, **PP 10** and **PP 15**, were tested for cardiotoxicity in the *Danio rerio* experimental model (OECD 236 test). Despite **PP 15** showing higher receptor selectivity and lower cytotoxicity, it exhibited significantly greater overall toxicity, with an LD_50_ value of 2.147 μg/mL compared to **PP 10**’s LD_50_ value of 15.34 μg/mL. Neither compound displayed cardiotoxic effects within the nontoxic range, as determined by heart rate measurements (Figure 4A–C). To assess potential neurotoxic effects, locomotor activity was examined in the *Danio rerio* model. Zebrafish larvae’s response to light and dark transitions serves as a useful method for detecting neurotoxicity. When exposed to sudden changes in light intensity, zebrafish larvae exhibit a startle response, typically involving quick, coordinated movements. In our experiment, we switched from light to dark conditions, as zebrafish larvae are known to avoid light and seek darker areas. This behavior helps them evade predators and maintain ideal environmental conditions. Our results demonstrated increased locomotor activity in the dark, suggesting minimal neurotoxic effects of the compounds (Figure 5A–C).

### 2.5 Molecular Modeling

**Figure 6:**
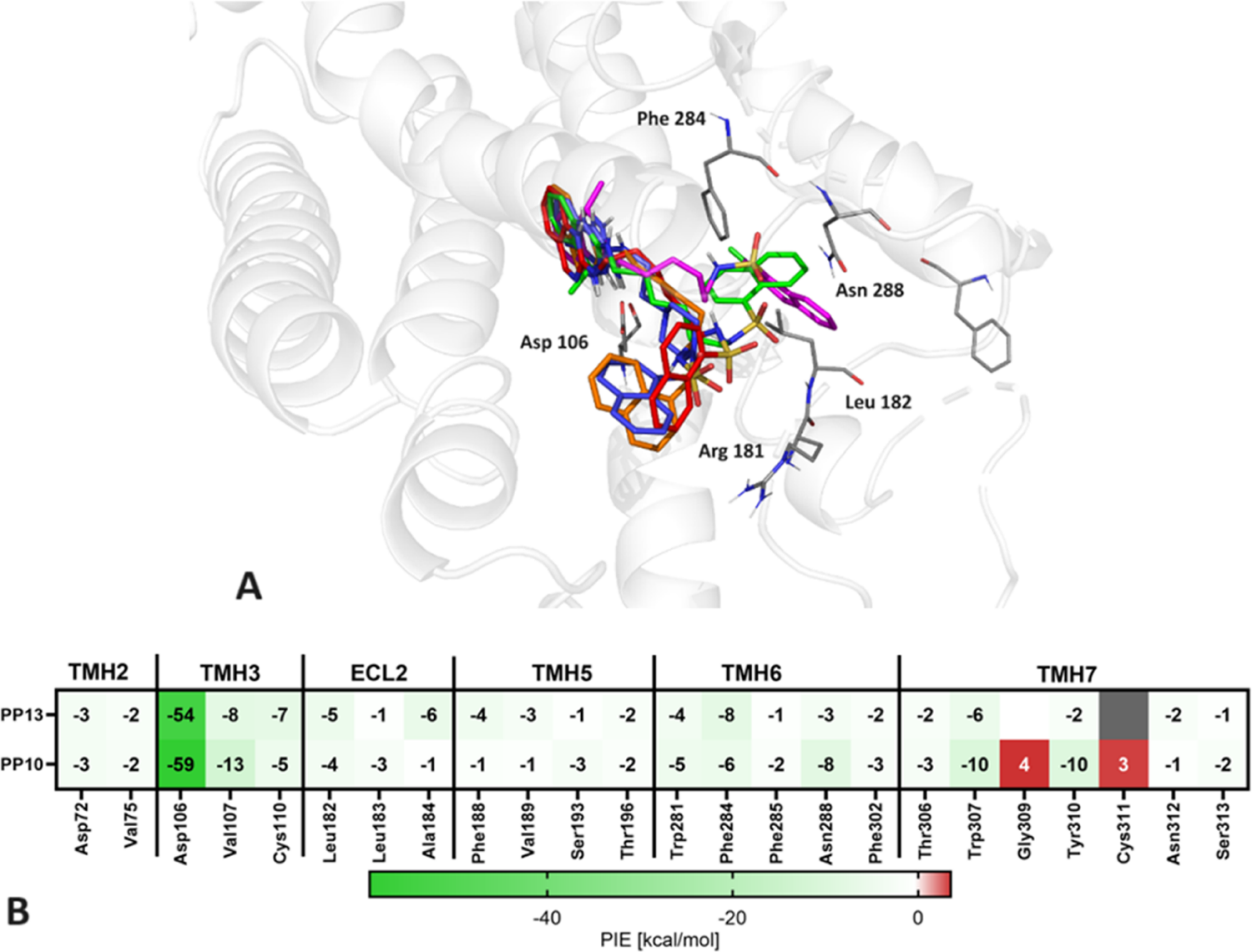
A: Poses of **PP 6** (blue), **PP 8** (orange), **PP 10** (green), **PP 13** (magenta), and **PP 15** (red), in 5-HT_6_R. B: Pair interaction energies (kcal/mol) between the ligand and residues of 5-HT_6_R are presented in the heat map. Attractive interactions are indicated in green, while strongly repulsive interactions are marked in red. Gray denotes the absence of any interaction.

The ligands were designed using molecular docking methods. After *in vitro* studies, the poses obtained in molecular modeling were reanalyzed to determine SAR. Molecular modeling was performed using the IFD protocol, which takes into account changes in amino acid arrangement under the influence of the docked ligand. We began with molecular docking, utilizing the crystal structure of 5-HT_6_R (PDB ID: 7XTB). The most promising complexes were then refined using the QM-polarized ligand docking (QPLD) methodology. Stabilization energies were computed using the FMO-EDA method to assess the contribution of each amino acid–ligand interaction to the total binding energy^50^ (Figure 6).

In the obtained poses, the 2-aminoquinazoline fragment facing the internal binding pocket was arranged similarly in all tested compounds. A strong salt bridge or hydrogen bond interactions with Asp 106 were observed in this region. Studying the relationship between active conformation and receptor affinity, we found that ligands with chains constrained by a piperazine ring (**PP 1** and **PP 5**) exhibit a different arrangement in the binding pocket compared to more flexible compounds. The quinazoline moiety is oriented perpendicularly compared to other ligands. In conformation of **PP 1** and **PP 5**, a hydrogen bond with Asp 106 was observed, but with an unfavorable geometry. In the case of compounds with a short, flexible alkyl linker (**PP 4**, **PP 9**, **PP 14**), the orientation of quinazoline was similar but bent toward helix 5, compared to ligands with longer linkers. In all ethyl derivatives, interaction with Asp 106 was observed, where 2 nitrogen atoms in the 2-aminoquinazoline fragment and NH in the sulfonamide acted as donors. Additional attraction of sulfonamide toward Asp 106 caused the bending of arylsulfone toward quinazoline and the appearance of repulsive interactions.

In the case of butyl and hexyl linkers, a similar arrangement of quinazoline was observed. For **PP 7** and **PP 2** in arylsulfone moiety stabilizing interaction with Arg 181 and no π–π contact in the quinazoline region was found. In hexyl analogues, π-stacking was present in the arylsulfone part with residues Phe 188 or Phe 284, as well as hydrogen bonding with Leu 182 and Asn 288. Additional stabilization explains the highest activity of compounds with the longest linker. **PP 15** with a nonhydrogenated quinazoline fragment, interacts with Asp 106 (hydrogen bond) with the most favorable geometry (distance 2.76 Å, angle 156°) compared to other ligands, which may explain its highest affinity. Replacing the quinazoline fragment with a pyrimidine resulted in a loss of affinity, due to the lack of a hydrogen bond with Asp 106. Among the derivatives from the second set, **PP 17**-**PP 20** showed low affinity for 5-HT_6_R, associated with poor filling of the binding pocket and the lack of additional attractive interactions. In arylpiperazine derivatives **PP 21** and **PP 22**, the affinity was higher, which was determined by both the interaction with Asp 106 in the quinazoline fragment and additional binding interactions in the arylpiperazine moiety, including a salt bridge with Asp 106.

In the case of **PP 13**, double stabilization was observed through interaction with this residue. **PP 15** formed an additional stabilizing π-cation bond with Phe 284. The resulting poses in the outer region exhibited two potential orientations: one bending toward helix 3, forming interactions with Trp 102 and Arg 181, and the other bending toward helix 5, interacting with Phe 188. To explore how the interactions between the ligand and individual residues affect the binding energies, the pair interaction energy (PIE) was analyzed using the FMO. (Figure 6B).^50^ The energy profile indicates that both compounds **PP 10** and **PP 13** form a very strong salt bridge with Asp 106 in the third helix and a series of weak but stabilizing interactions with residues in helices 2, 5, and 6. Interactions with helix 5, especially with Ser193, may suggest an agonistic nature of the interaction of these ligands with the 5-HT_6_ receptor.^27,51^ FMO analysis indicates that the main difference in the binding mode of **PP 10** and **PP 13** occurs in helix 7. The presence of a halogen substituent in the terminal aromatic ring of the **PP 13** structure caused the disappearance of two destabilizing interactions with Gly309 and Cys311.

**Figure 7:**
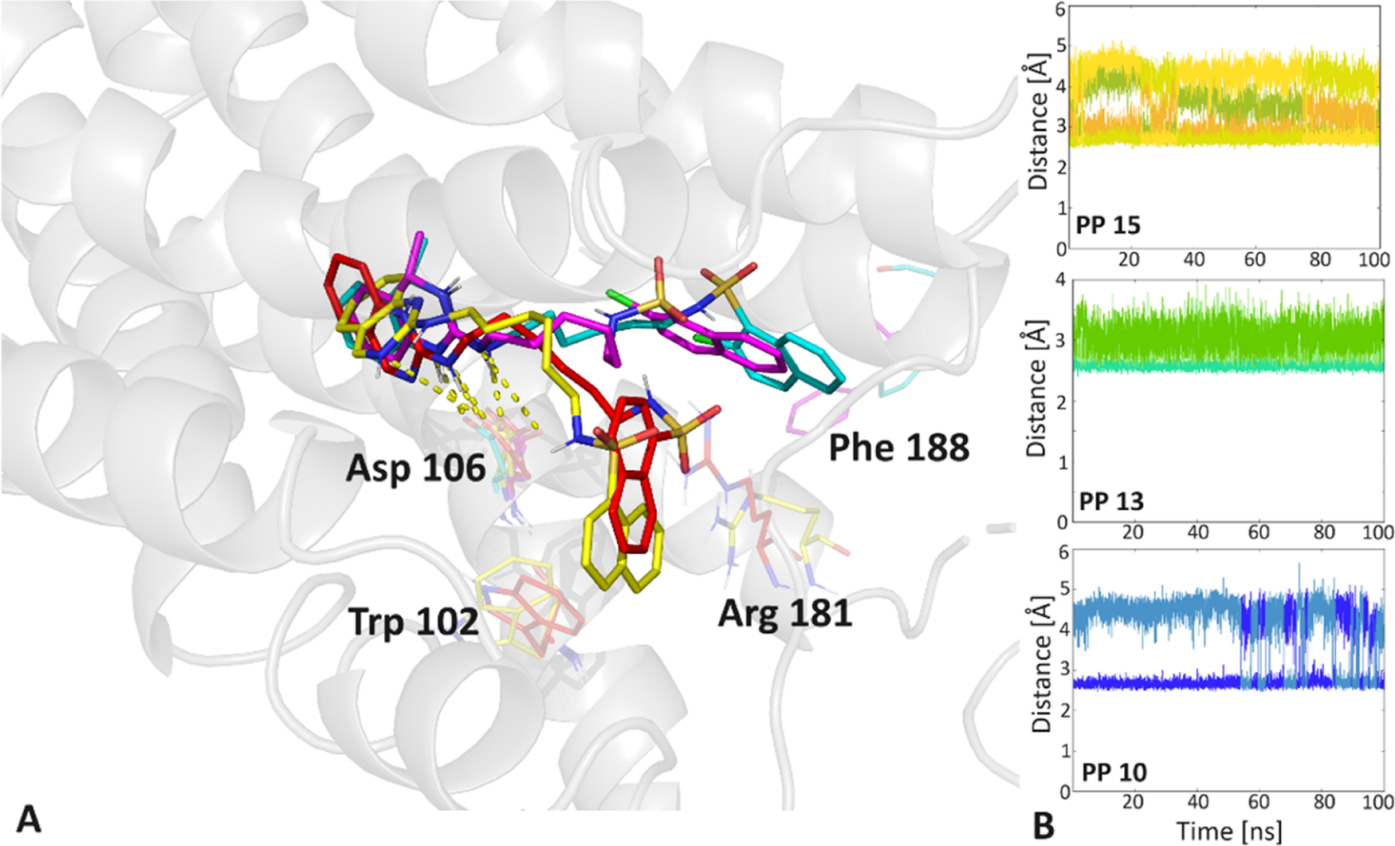
A: The binding modes of **PP 13** (magenta) and **PP 15** (red) were compared to the representative poses of **PP 13** (cyan) and **PP 15** (yellow) obtained from molecular dynamics clustering within the 5-HT_6_R. Hydrogen bonds are indicated by yellow dashed lines, with key residues highlighted as colored sticks. B: The ligand-Asp 106 distance is plotted as a function of time.

To determine the stability of the obtained complexes and the bonds observed in them, molecular dynamics simulations were performed for selected ligands **PP 6**, **PP 8**, **PP 10, PP 13**, and **PP 15** in NAMD.^52^ The results were presented in the example of the compounds with the highest affinity, **PP 13** and **PP 15** (Figure 7).

It is important to highlight that all poses exhibited excellent stability, with the root-mean-square deviation (RMSD) consistently remaining well below 2 Å. Observing the most representative dynamic poses (clustering) clearly shows their overlap with the initial conformations. Moreover, the high stability of interactions with Asp 106 should be emphasized, which may translate into the high affinity of the compounds. Building on the biological test and docking results, a 3D-QSAR analysis and pharmacophore model were created to optimize the design of future compounds with improved binding affinity. The data set of 24 compounds was randomly divided into a training set and a test set in a 1:1 ratio (50/50%). The training set was utilized to construct multiple 3D-QSAR models based on the Gaussian field (GFQSAR) methodology, with the model’s robustness assessed using partial least-squares (PLS) regression. Two PLS coefficients were employed to validate the model’s reliability and predictive capability. The most effective 3D-QSAR models were visualized by highlighting regions that are favorable or unfavorable for binding. The top GFQSAR model achieved *R*^2^ values of 0.97 for the training set and 0.84 for the test set.

**Figure 8:**
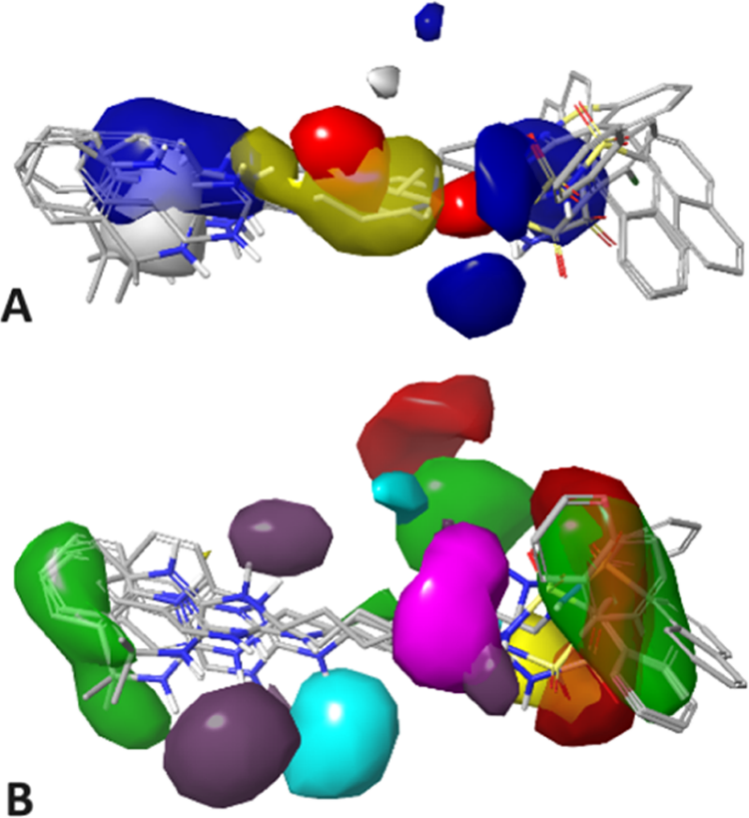
Contours obtained by GFQSAR. A: Electrostatic interactions are represented by positive (blue) and negative (red) charges and hydrophobic interactions by positive (yellow) and negative (white) markings. B: steric interactions are represented by positive (green) and negative (yellow) coloring, hydrogen bond donors (HBD) by positive (violet) and negative (cyan) colors, and hydrogen bond acceptors (HBA) by positive (red) and negative (magenta) hues.

The model highlighted that expanding the quinazoline ring at positions 8 and 7, along with substituting the arylsulfonamide at positions 1 and 2 with phenyl groups, enhances steric favorability. In contrast, steric hindrance in the carbon linker region negatively impacts affinity (Figure 8A). Furthermore, the hydrogen bond donor (HBD) in the quinazoline (Figure 8A) was identified as a critical factor for improving compound affinity.

**Figure 9:**
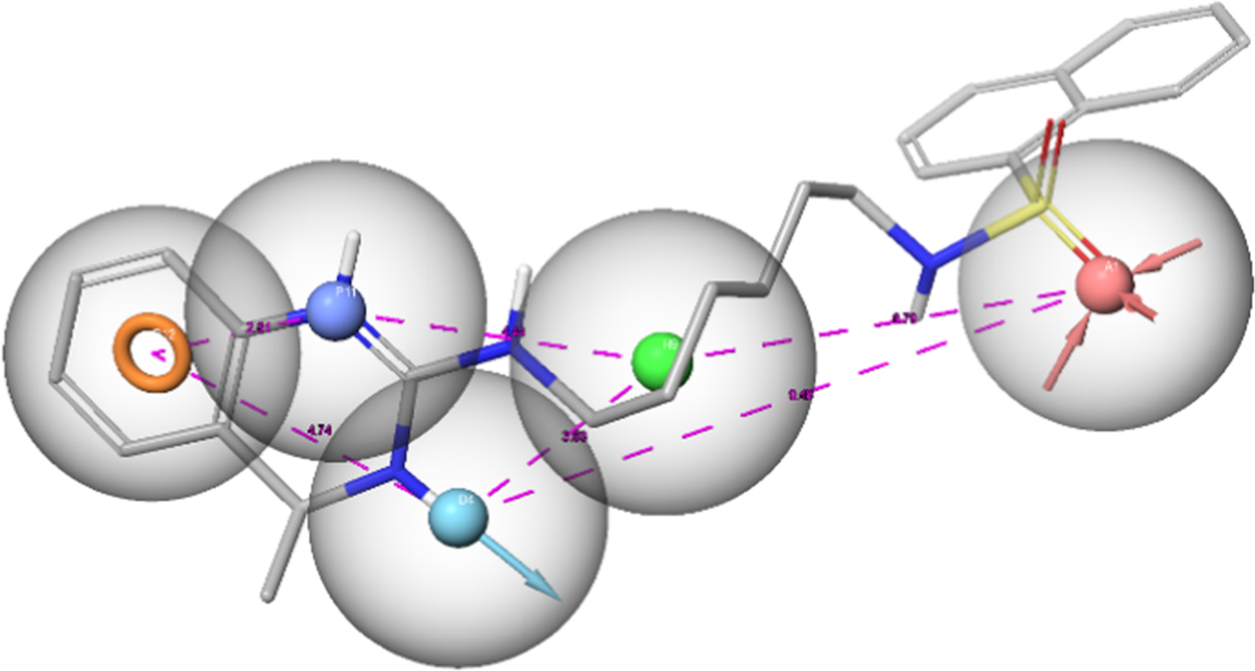
ADHPR pharmacophore model hypothesis includes a hydrogen bond donor HBD (D4), HBA (A1), positive ionization center (P11), aromatic ring (R12), and a hydrophobic fragment H5 shown in cyan, red, blue, orange, and green, respectively.

Employing a rigorous pKi threshold of ≥6.7 for active compounds and ≤6.3 for inactive ones, a pharmacophore model was constructed based on the ligands’ affinity for the 5-HT_6_ receptor. Ten pharmacophore models were generated, each with distinct combinations of pharmacophore variants, primarily focusing on ADHPR, AAHPR, or DHPRR. Among these, ADHPR exhibited the strongest correlation with the most active compounds, delivering the best survival site score and selectivity score (Figure 9).

## 3 Conclusions

Our research provided a group of new compounds belonging to long-chain cyclic arylguanidines. A chemotype of multifunctional serotonin receptor ligands was developed, focusing on the 5-HT_6_R receptor. Importantly, a correlation was noticed between the length of the carbon linker in ligand structure and the affinity for 5-HT_6_R. The developed group includes the selective 5-HT_6_R antagonist **PP 15**, dual ligand for 5-HT_1A_R/5-HT_6_R **PP 13**, and dual ligand for 5-HT_5A_R/5-HT_6_R **PP 10**. The use of a nonhydrogenated quinazoline ring was associated with high selectivity for 5-HT_6_R, while hydrogenation of the double bond in the 3,4-position led to a significant increase in affinity for other serotonin receptors.

Importantly, the selective 5-HT_6_R ligand **PP 15** showed low antiproliferative activity against the tested cancer lines. The use of multifunctional ligands was associated with high anticancer activity both against selected glioma cell lines and other cancers (IC_50_ < 25 μM). Affinity for the 5-HT_6_R does not impact antiproliferative efficacy, however, it may improve cognitive functions or induce an additional antidepressant effect during clinical anticancer therapy.

The obtained compounds showed satisfactory metabolic stability (52–93% of unmetabolized ligands in MLMs). At concentrations up to 1 μM, they did not affect the activity of cytochrome P-450 (CYP3A4 and CYP2D6). Above 10 μM, a partial or strong effect on the activity of these isoforms was observed, which may indicate the possibility of DDI at high concentrations.

The biggest limitation is poor passive diffusion through the membrane and high PPB capacity (fb > 98%). Both limitations may result from the strong lipophilicity of the obtained molecules, related to the long carbon linker. Importantly, there are known methods of delivering drugs that do not cross the blood–brain barrier, including carriers,^42^ opening the barrier,^43^ using additional transporters,^44^ or intranasal administration.^45^ Moreover, the compounds also showed high activity against breast cancer (MCF7) and pancreatic cancer (AsPC-1) cell lines, which may be an interesting direction for their further development. Due to the interesting anticancer properties, the structures should be optimized in terms of ADME parameters, lowering their lipophilicity by using a shorter carbon linker or incorporating heteroatoms. The high metabolic stability and cytotoxicity of **PP 24** indicate interesting properties of cyclic sulfonamide derivatives allowing the selection of a new hit for further research.

## 4 Materials and Methods

### 4.1 Chemical Synthesis

Microwave-induced reactions were performed using a CEM Discover microwave reactor. Ultrasonic synthesis was performed in an ultrasonic bath PS-08, with a power of 80 W and a frequency of 40 kHz. Thin-layer chromatography (TLC) was conducted using Sigma-Aldrich plates (silica gel on aluminum, with a fluorescent indicator at 254 nm, layer thickness of 200 μm, pore diameter of 60 Å, and particle size ranging from 8.0–12.0 μm). The analysis was carried out under UV light at 254 nm. Melting points were determined using a Boëtius apparatus. IR spectra were obtained with an FTS-165 spectrometer. NMR spectra were recorded on a Bruker Avance 400 MHz spectrometer, with trimethylsilyl (TMS) as the internal standard. For liquid chromatography–mass spectrometry (LC-MS) analysis, a Waters Acquity UPLC system was coupled with a Waters TQD mass spectrometer, operating in electrospray ionization (ESI) mode with tandem quadrupole detection. The analyses were conducted using an Acquity UPLC BEH C18 column (1.7 μm, 2.1 mm × 100 mm).

#### 4.1.1 Synthesis of *N*-(Aminoalkyl)naphthalenesulfonamides **3a**–**f** and 1-(Naphthalenesulfonyl)piperazines **3g**–**h**

In a round-bottom flask, 10 mmol of BOC-protected diamine *tert*-butyl(aminoalkyl)carbamates **1a**–**c** or *tert*-butyl piperazine-1-carboxylate **1d** with 10 mmol of naphthalenesulfonyl chlorides **2a**–**c** were mixed. The mixture was dissolved in 30 mL of CH_2_Cl_2_, and then while stirring at room temperature, 4.2 mL (30 mmol) of TEA was added dropwise. Stirring was continued for 1 h. The progress of the reaction was monitored by TLC. After this time, the solvent was evaporated, and 15 mL of 17% HCl was added to the residue. The resulting mixture was heated at 80 °C for 1 h. After this time, the liquid was neutralized with a saturated sodium bicarbonate solution and then extracted with CH_2_Cl_2_. After evaporation, the amine was used directly for the next step.

#### 4.1.2 Synthesis of 2-[4-(Naphthalenesulfonyl)piperazin-1-yl]-3,4-dihydroquinazolines **PP 1**,**5** and *N*-{[(3,4-Dihydroquinazolin-2-yl)amino]alkyl}naphthalenesulfonamides **PP 2–4**,**7**–**13**

1 mmol of *N*-(aminoalkyl)naphthalenesulfonamides **3a**–**f** or 1-(naphthalenesulfonyl)piperazines **3g**–**h** with 1 mmol of 2-(methylsulfanyl)-3,4-dihydroquinazoline derivatives **4a**–**d** were mixed in a round-bottom flask. Then, 0.42 mL (3 mmol) of TEA was added to the flask. The resulting mixture was heated without stirring at 180 °C for 1 h. The progress of the reaction was monitored by TLC. After cooling, 5 mL of H_2_O was added to the mixture, followed by extraction three times with CH_2_Cl_2_. The mixed organic phases were dried over anhydrous MgSO_4_ and then evaporated. The crude product was separated on a column (CHCl_3_/MeOH 9:1).

##### 4.1.2.1 2-[4-(Naphthalene-2-sulfonyl)piperazin-1-yl]-3,4-dihydroquinazoline **PP 1**

Formula weight for C_22_H_22_N_4_O_2_S: 406.5 g/mol, UPLC-MS: [M + H]^+^ = 407.4, purity = 100%, *R*_t_ = 4.84 min, *Y* = 49%, mp = >280 °C. ^1^H NMR (400 MHz, DMSO) δ 10.53 (bp, 1H, NH), 8.71 (bp, 1H, NH), 8.50 (bp, 1H, NH), 8.26–8.17 (m, 2H, ArH), 8.09 (d, *J* = 8.0 Hz, 1H, ArH), 7.82–7.69 (m, 3H, ArH), 7.25 (t, *J* = 7.8 Hz, 1H, ArH), 7.17 (d, *J* = 6.9 Hz, 1H, ArH), 7.08 (dd, *J* = 14.6, 7.4 Hz, 2H, ArH), 4.35 (s, 2H, AlH), 3.65 (s, 4H, AlH), 3.13 (s, 4H, AlH). ^13^C NMR (101 MHz, DMSO) δ 135.08 (C_Gua_), 134.00 (ArC), 132.36 (ArC), 132.32 (ArC), 132.27 (ArC), 129.99 (ArC), 129.81 (ArC), 129.45 (ArC), 128.76 (ArC), 128.36 (ArC), 128.26 (ArC), 126.20 (ArC), 125.09 (ArC), 124.84 (ArC), 123.53 (ArC), 123.45 (ArC), 123.29 (ArC), 46.04 (2AlC), 45.76 (2AlC), 41.50 (AlC). FT-IR: 3375 (N–H, Str), 2958, 2918, 2850 (C–H Aliph, Str) 1735 (C–H Ar, Bend), 1636 (C=N, Str), 1560 (N–H, Bend), 1495, 1453 (−CH_2_, Bend), 1381, 1346 (S=O, −SO_2_, Str), 1283, 1259 (C–N Ar, Str), 1189, 1159, 1131 (S=O, sulfonamide, Str), 1075, 1051, 1033 (C–N, Str), 952, 939 (C=C Ar, Bend).

##### 4.1.2.2 *N*-{4-[(1,4-Dihydroquinazolin-2-yl)amino]butyl}naphthalene-2-sulfonamide **PP 2**

Described in ref (38).

##### 4.1.2.3 *N*-{6-[(1,4-Dihydroquinazolin-2-yl)amino]hexyl}naphthalene-2-sulfonamide **PP 3**

Described in ref (38).

##### 4.1.2.4 *N*-{2-[(3,4-Dihydroquinazolin-2-yl)amino]ethyl}naphthalene-1-sulfonamide **PP 4**

Formula weight for C_20_H_20_N_4_O_2_S: 380.5 g/mol, UPLC-MS: [M + H]^+^ = 381.0, purity = 99%, *R*_t_ = 4.32 min, *Y* = 45%, mp = 76–82 °C. ^1^H NMR (400 MHz, DMSO) δ 10.25 (bp, 1H. NH), 8.63 (d, *J* = 8.6 Hz, 1H, ArH), 8.22 (d, *J* = 8.3 Hz, 1H, ArH), 8.15 (bp, 2H, NH), 8.06 (d, *J* = 7.8 Hz, 1H, ArH), 7.77–7.62 (m, 4H, ArH), 7.28 (t, *J* = 7.7 Hz, 1H, ArH), 7.21 (d, *J* = 6.9 Hz, 1H, ArH), 7.11 (td, *J* = 7.5, 0.9 Hz, 1H, ArH), 7.03 (d, *J* = 7.9 Hz, 1H, ArH), 4.42 (s, 2H, AlH), 3.39–3.34 (m, 2H, AlH), 3.04–2.95 (m, 2H, AlH). ^13^C NMR (101 MHz, DMSO) δ 151.86 (C_Gua_), 135.49 (ArC), 134.35 (2ArC), 133.66 (ArC), 129.44 (ArC), 128.92 (ArC), 128.88 (ArC), 128.44 (ArC), 127.89 (ArC), 127.35 (ArC), 126.61 (ArC), 124.99 (ArC), 124.94 (ArC), 124.65 (ArC), 118.83 (ArC), 115.95 (ArC), 79.66 (ArC), 41.93 (AlC), 41.38 (AlC), 41.31 (AlC). FT-IR: 3176 (N–H, Str), 3030 (C–H Ar, Str), 2970 (C–H Aliph, Str), 1738 (C–H Ar, Bend), 1663 (C=N, Str), 1589 (N–H, Bend), 1496 (−CH_2_, Bend) 1365, 1346, (S=O, −SO_2_, Str), 1319 (C–N Ar, Str), 1158 (S=O, sulfonamide, Str), 1260, 1231, 1216, 1130 (C–N, Str), 979, 928 (C=C Ar, Bend).

##### 4.1.2.5 2-[4-(Naphthalene-1-sulfonyl)piperazin-1-yl]-3,4-dihydroquinazoline **PP 5**

Formula weight for C_22_H_22_ClN_4_O_2_S: 406.5 g/mol, UPLC-MS: [M + H]^+^ = 407.4, purity = 100%, *R*_t_ = 5.29 min, *Y* = 79%, ^1^H NMR (400 MHz, DMSO) δ 9.33 (bp, *J* = 126.7 Hz, 2H, NH), 8.71 (dd, *J* = 20.1, 8.3 Hz, 1H, ArH), 8.34 (d, *J* = 6.8 Hz, 2H, ArH), 8.15–8.12 (m, 1H, NH), 7.82–7.62 (m, 4H, ArH), 7.24 (dd, *J* = 10.9, 4.5 Hz, 1H, ArH), 7.18 (d, *J* = 6.7 Hz, 1H, ArH), 7.12–7.04 (m, 2H, ArH), 4.37 (s, 2H, AlH), 3.67–3.54 (m, 4H, AlH), 3.27–3.19 (m, 4H, AlH). ^13^C NMR (101 MHz, DMSO) δ 151.76 (C_Gua_), 135.39 (ArC), 134.75 (ArC), 134.51 (ArC), 131.92 (ArC), 131.00 (ArC), 129.67 (ArC), 128.92 (ArC), 128.87 (ArC), 128.73 (ArC), 127.53 (ArC), 126.17 (ArC), 125.20 (ArC), 124.99 (ArC), 124.94 (ArC), 120.78 (ArC), 117.05 (ArC), 79.68 (AlC), 46.27 (AlC), 45.39 (AlC), 43.97 (AlC), 41.61 (AlC).

##### 4.1.2.6 *N*-{4-[(3,4-Dihydroquinazolin-2-yl)amino]butyl}naphthalene-1-sulfonamide **PP 7**

Formula weight for C_22_H_24_N_4_O_2_S: 408.5 g/mol, UPLC-MS: [M + H]^+^ = 408.9, purity = 97%, *R*_t_ = 5.61 min, *Y* = 61%, ^1^H NMR (400 MHz, DMSO) δ 10.23 (bp, 1H, NH), 8.66 (d, *J* = 8.6 Hz, 1H, ArH), 8.21 (d, *J* = 8.3 Hz, 1H, ArH), 8.12 (dd, *J* = 7.3, 1.1 Hz, 1H, ArH), 8.08 (d, *J* = 7.6 Hz, 1H, ArH), 8.01 (bp, 1H, NH), 7.85 (bp, 1H, NH), 7.71 (ddd, *J* = 8.5, 6.9, 1.4 Hz, 1H, ArH), 7.68–7.62 (m, 2H, ArH), 7.27 (t, *J* = 7.7 Hz, 1H, ArH), 7.22 (d, *J* = 6.9 Hz, 1H, ArH), 7.14–7.07 (m, 1H, ArH), 7.05 (d, *J* = 7.5 Hz, 1H, ArH), 4.46 (s, 2H, AlH), 3.14 (s, 2H, AlH), 2.82 (d, *J* = 5.0 Hz, 2H, AlH), 1.40 (dd, *J* = 24.2, 21.6 Hz, 4H, AlH). ^13^C NMR (101 MHz, DMSO) δ 151.78 (C_Gua_), 136.10 (ArC), 134.32 (ArC), 134.11 (ArC), 133.80 (ArC), 129.39 (ArC), 128.88 (ArC), 128.77 (ArC), 128.27 (ArC), 127.99 (ArC), 127.30 (ArC), 126.63 (ArC), 125.12 (ArC), 124.99 (ArC), 124.55 (ArC), 118.94, (ArC) 115.89 (ArC), 79.68 (AlC), 42.43 (AlC), 41.34 (AlC), 26.88 (AlC), 26.10 (AlC).

##### 4.1.2.7 *N*-{6-[(3,4-Dihydroquinazolin-2-yl)amino]hexyl}naphthalene-1-sulfonamide **PP 8**

Formula weight for C_24_H_28_N_4_O_2_S: 436.6 g/mol, UPLC-MS: [M + H]^+^ = 437.0, purity = 100%, *R*_t_ = 5.49 min, *Y* = 61%, mp = 69–73 °C. Composition: C(66.03%) H(6.46%) N(12.83%) O(7.33%) S(7.34%), found: C(66.24%) H(6.41%) N(12.893%) S(7.30%). ^1^H NMR (400 MHz, DMSO) δ 10.27 (bp, 1H, NH), 8.66 (d, *J* = 8.6 Hz, 1H, ArH), 8.22 (d, *J* = 8.2 Hz, 1H, ArH), 8.09 (ddd, *J* = 16.0, 7.9, 4.3 Hz, 2H, ArH), 7.92 (bp, 2H, NH), 7.73–7.61 (m, 3H, ArH), 7.27 (t, *J* = 7.7 Hz, 1H, ArH), 7.22 (d, *J* = 6.9 Hz, 1H, ArH), 7.13–6.99 (m, 2H, ArH), 4.48 (s, 2H, AlH), 3.15 (s, 2H, AlH), 2.78 (dd, *J* = 12.5, 6.6 Hz, 2H, AlH), 1.31 (dd, *J* = 15.3, 8.4 Hz, 4H, AlH), 1.18–1.06 (m, 4H, AlH).^13^C NMR (101 MHz, DMSO) δ 151.86 (C_Gua_), 136.23 (ArC), 134.32 (ArC), 134.06 (ArC), 133.83 (ArC), 129.36 (ArC), 128.88 (ArC), 128.79 (ArC), 128.22 (ArC), 128.03 (ArC), 127.27 (ArC), 126.63 (ArC), 125.19 (ArC), 124.97 (ArC), 124.53 (ArC), 118.97 (ArC), 115.86 (ArC), 79.68 (AlC), 42.71 (AlC), 41.35 (AlC), 29.41 (AlC), 28.71 (AlC), 25.94 (AlC), 25.88 (AlC). FT-IR: 3164 (N–H, Str), 2969, 2931 (C–H Aliph, Str) 1738 (C–H Ar, Bend), 1665 (C=N, Str), 1589 (N–H, Bend), 1495 (−CH_2_, Bend), 1365 (S=O, −SO_2_, Str), 1315 (C–N Ar, Str), 1157 (S=O, sulfonamide, Str), 1129, 1073, 1027 (C–N, Str), 980 (C=C Ar, Bend).

##### 4.1.2.8 *N*-{2-[(4-Methyl-3,4-dihydroquinazolin-2-yl)amino]ethyl}naphthalene-1-sulfonamide **PP 9**

Formula weight for C_21_H_22_N_4_O_2_S: 394.5 g/mol, UPLC-MS: [M + H]^+^ = 395.2, *R*_t_ = 7.30 min, purity = 99%, *Y* = 77% mp = 68–72 °C. ^1^H NMR (400 MHz, DMSO) δ 10.42 (bp, 1H, NH), 8.63 (d, *J* = 8.5 Hz, 1H, NH), 8.52 (bp, 1H, NH), 8.22 (d, *J* = 8.3 Hz, 2H, ArH), 8.13 (dd, *J* = 7.3, 1.0 Hz, 1H, ArH), 8.07 (d, *J* = 7.7 Hz, 1H, ArH), 7.76–7.60 (m, 4H, ArH, NH), 7.33–7.22 (m, 2H, ArH), 7.13 (t, *J* = 7.5 Hz, 1H, ArH), 7.06 (d, *J* = 8.0 Hz, 1H, ArH), 4.72 (q, *J* = 6.3 Hz, 1H, AlH), 3.39 (s, 2H, AlH), 2.99 (s, 2H, AlH), 1.35 (d, *J* = 6.6 Hz, 3H, AlH). ^13^C NMR (101 MHz, DMSO) δ 150.96 (C_Gua_), 135.49 (ArC), 134.35 (2ArC), 129.44 (ArC), 128.90 (ArC), 128.86 (ArC), 128.44 (2ArC), 127.90 (ArC), 127.36 (ArC), 126.32 (ArC), 124.98 (ArC), 124.95 (ArC), 124.75 (ArC), 123.83 (ArC), 116.06 (ArC), 79.65 (AlC), 47.97 (AlC), 41.32 (AlC), 24.49 (AlC). FT-IR: 3191 (N–H, Str), 3068 (C–H Ar, Str), 2971, 2927 (C–H Aliph, Str) 1732 (C–H Ar, Bend), 1659, 1629 (C=N, Str), 1587 (N–H, Bend), 1496, 1443 (−CH_2_, Bend), 1371, 1346 (S=O, −SO_2_, Str), 1318 (C–F, Str), 1256 (C–N Ar, Str), 1201 (C–N amine, Str) 1158, 1130 (S=O, sulfonamide, Str), 1090, 1042, 1026 (C–N, Str), 979, 927 (C=C Ar, Bend).

##### 4.1.2.9 *N*-{6-[(4-Methyl-3,4-dihydroquinazolin-2-yl)amino]hexyl}naphthalene-1-sulfonamide **PP 10**

Formula weight for C_25_H_30_N_4_O_2_S: 450.6 g/mol, UPLC-MS: [M + H]^+^ = 451.3, purity = 99%, *R*_t_ = 6.09 min, *Y* = 86% mp = 68–74 °C. Composition: C(66.64%) H(6.71%) N(12.43%) O(7.10%) S(7.11%), found: C(66.56%) H(6.75%) N(12.41%) S(7.19%). ^1^H NMR (400 MHz, DMSO) δ 10.37 (bp, 1H, NH), 8.66 (d, *J* = 8.6 Hz, 1H, ArH), 8.48 (bp, 1H, NH), 8.22 (d, *J* = 8.2 Hz, 1H, ArH), 8.14–8.06 (m, 2H, ArH), 7.94 (t, *J* = 5.7 Hz, 1H, ArH), 7.82 (bp, 1H, NH), 7.74–7.60 (m, 3H, ArH), 7.32–7.22 (m, 2H, ArH), 7.16–7.02 (m, 2H, ArH), 4.77 (d, *J* = 5.7 Hz, 1H, AlH), 3.25–3.05 (m, 2H, AlH), 2.78 (dd, *J* = 12.8, 6.7 Hz, 2H, AlH), 1.37 (d, *J* = 6.6 Hz, 3H, AlH), 1.35–1.23 (m, 4H, AlH), 1.13 (d, *J* = 3.3 Hz, 4H, AlH). ^13^C NMR (101 MHz, DMSO) δ 150.89 (C_Gua_), 136.24 (ArC), 134.32 (ArC), 134.06 (ArC), 132.69 (ArC), 129.37 (ArC), 128.90 (ArC), 128.79 (ArC), 128.21 (ArC), 128.03 (ArC), 127.28 (ArC), 126.36 (ArC), 125.19 (ArC), 124.97 (ArC), 124.72 (ArC), 123.89 (ArC), 115.96 (ArC), 79.65 (AlC), 47.95 (AlC), 42.71 (AlC), 41.29 (AlC), 29.42 (AlC), 28.78 (AlC), 25.87 (AlC), 24.53 (AlC). FT-IR: 3165 (N–H, Str), 3065 (C–H Ar, Str), 2926, 2855 (C–H Aliph, Str) 1738 (C–H Ar, Bend), 1661, 1626 (C=N, Str), 1587 (N–H, Bend), 1495, 1444 (−CH_2_, Bend), 1364, 1346 (S=O, −SO_2_, Str), 1314 (C–F, Str), 1256 (C–N Ar, Str), 1200 (C–N amine, Str) 1157, 1130 (S=O, sulfonamide, Str), 1081, 1027 (C–N, Str), 978, 938 (C=C Ar, Bend).

##### 4.1.2.10 *N*-{6-[(5-Fluoro-4-methyl-3,4-dihydroquinazolin-2-yl)amino]hexyl}naphthalene-1-sulfonamide **PP 11**

Formula weight for C_25_H_29_FN_4_O_2_S: 468.6 g/mol, UPLC-MS: [M + H]^+^ = 469.3, purity = 98%, *R*_t_ = 6.37 min, *Y* = 71%, mp = 76–80 °C. ^1^H NMR (400 MHz, DMSO) δ 10.77 (bp, 1H, NH), 8.66 (d, *J* = 8.6 Hz, 1H, ArH), 8.22 (d, *J* = 8.3 Hz, 1H, ArH), 8.14–8.08 (m, 2H, ArH), 8.04 (bp, 1H, NH), 7.94 (bp, 1H, NH), 7.73–7.62 (m, 3H, ArH), 7.34 (td, *J* = 8.2, 6.3 Hz, 1H, ArH), 7.04–6.88 (m, 2H, ArH), 4.89 (q, *J* = 6.5 Hz, 1H, AlH), 3.18 (d, *J* = 8.4 Hz, 2H, AlH), 2.78 (dd, *J* = 12.4, 6.5 Hz, 2H, AlH), 1.38–1.27 (m, 7H, AlH), 1.13 (d, *J* = 3.2 Hz, 4H, AlH).^13^C NMR (101 MHz, DMSO) δ 159.51 (C_Gua_), 157.08 (ArC), 150.64 (ArC), 136.23 (ArC), 134.32 (ArC), 134.06 (ArC), 130.56 (ArC), 130.46 (ArC), 129.36 (ArC), 128.79 (ArC), 128.2 (ArC)1, 128.03 (ArC), 127.27 (ArC), 125.20 (ArC), 124.97 (ArC), 111.12 (ArC), 110.92 (ArC), 79.66 (AlC), 70.24 (AlC), 42.71 (AlC), 41.36 (AlC), 29.41 (AlC), 28.72 (AlC), 25.94 (AlC), 23.93 (AlC). FT-IR: 3227 (N–H, Str), 3077 (C–H Ar, Str), 2928, 2858 (C–H Aliph, Str) 1738 (C–H Ar, Bend), 1689, 1651, 1633, 1614 (C=N, Str), 1545 (N–H, Bend), 1507, 1428 (−CH_2_, Bend), 1367 (S=O, −SO_2_, Str), 1318 (C–F, Str), 1294 (C–N Ar, Str), 1235, 1214 (C–N amine, Str) 1159, 1132 (S=O, sulfonamide, Str), 1078, 1053 (C–N, Str), 982, 955, 937 (C=C Ar, Bend).

##### 4.1.2.11 *N*-{6-[(6,8-Dichloro-4-methyl-3,4-dihydroquinazolin-2-yl)amino]hexyl}naphthalene-1-sulfonamide **PP 12**

Formula weight for C_25_H_28_Cl_2_N_4_O_2_S: 519.5 g/mol, UPLC-MS: [M + H]^+^ = 519.2, purity = 100%, *R*_t_ = 6.85 min, *Y* = 41%, mp = 96–100 °C. ^1^H NMR (400 MHz, DMSO) δ 9.93 (bp, 1H, NH), 9.03 (bp, 1H, NH), 8.66 (d, *J* = 8.5 Hz, 1H, ArH), 8.41 (bp, 1H, NH), 8.22 (d, *J* = 8.2 Hz, 1H, ArH), 8.13–8.07 (m, 2H, ArH), 7.95 (t, *J* = 5.7 Hz, 1H, ArH), 7.74–7.61 (m, 4H, ArH), 7.45 (d, *J* = 2.0 Hz, 1H, ArH), 4.85 (d, *J* = 5.7 Hz, 1H, AlH), 3.28–3.06 (m, 2H, AlH), 2.79 (dd, *J* = 12.9, 6.7 Hz, 2H, AlH), 1.43–1.24 (m, 7H, AlH), 1.14 (s, 4H, AlH). ^13^C NMR (101 MHz, DMSO) δ 150.90 (C_Gua_), 136.22 (ArC), 134.32 (ArC), 134.06 (ArC), 129.36 (ArC), 129.21 (ArC), 128.79 (ArC), 128.54 (ArC), 128.22 (ArC), 128.03 (ArC), 127.28 (ArC), 126.93 (ArC), 126.90 (ArC), 125.57 (ArC), 125.19 (ArC), 124.97 (ArC), 120.13 (ArC), 55.38 (AlC), 48.00 (AlC), 42.70 (AlC), 41.45 (AlC), 29.40 (AlC), 28.41 (AlC), 25.88 (AlC), 24.48 (AlC). FT-IR: 3135 (N–H, Str), 3057 (C–H Ar, Str), 2970, 2937, 2861 (C–H Aliph, Str), 1738 (C–H Ar, Bend), 1666, 1615 (C=N, Str), 1579 (N–H, Bend), 1506, 1435 (−CH_2_, Bend), 1366 (S=O, −SO_2_, Str), 1317 (C–N Ar, Str), 1263, 1216 (C–N amine, Str) 1158, 1130 (S=O, sulfonamide, Str), 1082 (C–N, Str), 981, 951 (C=C Ar, Bend), 832, 803, 771, 730, 669 (C–Cl, Str).

##### 4.1.2.12 2-Chloro-*N*-{6-[(4-methyl-3,4-dihydroquinazolin-2-yl)amino]hexyl}naphthalene-1-sulfonamide **PP 13**

Formula weight for C_25_H_29_ClN_4_O_2_S: 485.0 g/mol, UPLC-MS: [M + H]^+^ = 485.2, purity = 97%, *R*_t_ = 6.64 min, *Y* = 76%, mp = 69–74 °C. Composition: C(61.91%) H(6.03%) Cl(7.31%) N(11.55%) O(6.60%) S(6.61%), found: (61.88%) H(6.06%) N(11.59%) S(6.54%). ^1^H NMR (400 MHz, DMSO) δ 10.32 (bp, 1H, NH), 9.05 (d, *J* = 9.1 Hz, 1H, ArH), 8.45 (bp, 1H, NH), 8.20–8.10 (m, 2H, ArH), 8.06 (d, *J* = 7.2 Hz, 1H, ArH), 7.77 (bp, 1H, ArH), 7.71 (ddd, *J* = 8.8, 6.9, 1.5 Hz, 1H, ArH), 7.65 (ddd, *J* = 7.9, 6.7, 3.2 Hz, 2H, ArH), 7.32–7.24 (m, 2H, ArH, NH), 7.17–7.04 (m, 2H, ArH), 4.77 (dd, *J* = 6.5, 2.7 Hz, 1H, AlH), 3.24–3.07 (m, 2H, AlH), 2.87 (dd, *J* = 12.9, 6.7 Hz, 2H, AlH), 1.40–1.27 (m, 7H, AlH), 1.15 (d, *J* = 3.5 Hz, 4H, AlH). ^13^C NMR (101 MHz, DMSO) δ 150.84 (C_Gua_), 134.75 (ArC), 133.90 (ArC), 133.03 (ArC), 132.87 (ArC), 132.67 (ArC), 130.97 (ArC), 129.48 (ArC), 129.35 (ArC), 128.98 (ArC), 128.90 (ArC), 127.25 (ArC), 126.36 (ArC), 125.73 (ArC), 124.73 (ArC), 123.90 (ArC), 115.98 (ArC), 79.65 (AlC), 47.96 (AlC), 42.51 (AlC), 41.29 (AlC), 29.30 (AlC), 28.79 (AlC), 25.99 (AlC), 24.53 (AlC). FT-IR: 3189 (N–H, Str), 3065 (C–H Ar, Str), 2931, 2859 (C–H Aliph, Str) 1739 (C–H Ar, Bend), 1660, 1626 (C=N, Str), 1586, 1555 (N–H, Bend), 1496, 1446, 1423 (−CH_2_, Bend), 1326 (S=O, −SO_2_, Str), 1299 (C–N Ar, Str), 1257, 1209 (C–N amine, Str) 1162, 1150, 1137, 1116 (S=O, sulfonamide, Str), 1082 (C–N, Str), 979 (C=C Ar, Bend), 853, 817, 749 (C–Cl, Str).

#### 4.1.3 Synthesis of *N*-{[(Quinazolin-2-yl)amino]alkyl}naphthalene-1-sulfonamides **PP 14**–**15** and *N*-{6-[(Pyrimidin-2-yl)amino]hexyl}naphthalene-1-sulfonamide **PP 16**

In a round-bottom flask, 1 mmol of *N*-(aminoalkyl)naphthalene-1-sulfonamides **3d**–**e**, 1 mmol of 2-chloropyrimidine **5a**/2-chloroquinazoline **5b**, 0.414 g of K_2_CO_3_ (3 mmol), and 5 mL EtOH were mixed. The contents were heated under reflux for 4 h. The progress of the reaction was monitored by TLC. After the reaction was completed, the solvent was evaporated. Next, 5 mL of H_2_O was added to the residue and extracted three times with CH_2_Cl_2_. The mixed organic phases were dried over anhydrous MgSO_4_ and then evaporated. The crude product was separated on a column (CHCl_3_/MeOH 9:1).

##### 4.1.3.1 *N*-{2-[(Quinazolin-2-yl)amino]ethyl}naphthalene-1-sulfonamide **PP 14**

Formula weight for C_20_H_18_N_4_O_2_S: 378.4 g/mol, UPLC-MS: [M + H]^+^ = 379.2, purity = 99%, *R*_t_ = 5.05 min, *Y* = 84%, mp = 177–184 °C. ^1^H NMR (400 MHz, DMSO) δ 9.04 (bp, 1H, NH), 8.66–8.58 (m, 1H, ArH), 8.21–8.11 (m, 3H, ArH, NH), 8.08–8.01 (m, 1H, ArH), 7.78 (dd, *J* = 8.0, 1.0 Hz, 1H, ArH), 7.71–7.60 (m, 4H, ArH, NH), 7.40 (d, *J* = 8.4 Hz, 1H, ArH), 7.24 (t, *J* = 7.0 Hz, 2H, ArH), 3.37 (dd, *J* = 12.2, 5.6 Hz, 2H, AlH), 3.05 (d, *J* = 6.1 Hz, 2H, ArH). ^13^C NMR (101 MHz, DMSO) δ 162.54 (C_Gua_), 159.72 (ArC), 135.82 (ArC), 134.57 (ArC), 134.30 (ArC), 134.14 (ArC), 129.37 (ArC), 129.00 (ArC), 128.35 (ArC), 128.21 (ArC), 127.94 (ArC), 127.23 (ArC), 125.23 (ArC), 125.06 (ArC), 124.92 (ArC), 122.58 (ArC), 120.13 (ArC), 41.12 (ArC). FT-IR: 3248 (N–H, Str), 3034 (C–H Ar, Str), 2970, 2850 (C–H Aliph, Str) 1738 (C–H Ar, Bend), 1621, 1600 (C=N, Str), 1557 (N–H, Bend), 1507, 1472, 1434, 1411 (−CH_2_, Bend), 1385, 1366, 1343 (S=O, −SO_2_, Str), 1319 (C–N Ar, Str), 1248, 1233, 1216, 1197 (C–N amine, Str) 1135, 1117 (S=O, sulfonamide, Str), 1096, 1025 (C–N, Str), 991, 950, 897 (C=C Ar, Bend).

##### 4.1.3.2 *N*-{6-[(Quinazolin-2-yl)amino]hexyl}naphthalene-1-sulfonamide **PP 15**

Formula weight for C_24_H_26_N_4_O_2_S: 434.6 g/mol, UPLC-MS: [M + H]^+^ = 435.3, purity = 99%, *R*_t_ = 6.57 min, *Y* = 89%, mp = 87–89 °C. Composition: C(66.33%) H(6.03%) N(12.89%) O(7.36%) S(7.38%), found: C(66.29%) H(6.09%) N(12.78%) S(7.35%). ^1^H NMR (400 MHz, DMSO) δ 9.07 (bp, 1H, NH), 8.68 (d, *J* = 8.7 Hz, 1H), 8.19 (d, *J* = 8.3 Hz, 1H, ArH), 8.12 (dt, *J* = 8.4, 4.2 Hz, 1H, ArH), 8.06 (t, *J* = 6.1 Hz, 1H, ArH), 7.92 (t, *J* = 5.7 Hz, 1H, ArH), 7.76 (dd, *J* = 8.0, 0.9 Hz, 1H, ArH), 7.72–7.68 (m, 1H, ArH), 7.67–7.62 (m, 2H, ArH), 7.44 (d, *J* = 8.4 Hz, 1H, ArH), 7.29 (bp, *J* = 5.3 Hz, 1H, NH), 7.22–7.15 (m, 1H, ArH), 3.37 (s, 1H,, AlH), 3.24 (dd, *J* = 13.2, 6.6 Hz, 2H, AlH), 2.78 (dd, *J* = 12.8, 6.7 Hz, 2H, AlH), 1.38 (bp, 2H, AlH), 1.32–1.22 (m, 2H, AlH), 1.17–1.05 (m, 4H, AlH). ^13^C NMR (101 MHz, DMSO) δ 162.36 (C_Gua_), 136.28 (ArC), 134.41 (ArC), 134.31 (ArC), 134.02 (ArC), 129.33 (ArC), 128.82 (ArC), 128.30 (ArC), 128.17 (ArC), 128.06 (ArC), 127.22 (2ArC), 125.22 (2ArC), 124.93 (ArC), 122.12 (ArC), 119.98 (ArC), 79.63 (AlC), 55.36 (AlC), 42.76 (AlC), 41.06 (AlC), 29.48 (AlC), 26.41 (AlC), 26.17 (AlC). FT-IR: 3271 (N–H, Str), 3061 (C–H Ar, Str), 2927, 2853 (C–H Aliph, Str) 1738 (C–H Ar, Bend), 1620 (C=N, Str), 1595 (N–H, Bend), 1552 (C=C Ar, Str), 1508, 1462 (−CH_2_, Bend), 1407, 1374, 1347 (S=O, −SO_2_, Str), 1310 (C–N Ar, Str), 1242, 1202 (C–N amine, Str) 1160, 1128 (S=O, sulfonamide, Str), 1079, 1026 (C–N, Str), 976, 932 (C=C Ar, Bend).

##### 4.1.3.3 *N*-{6-[(Pyrimidin-2-yl)amino]hexyl}naphthalene-1-sulfonamide **PP 16**

Formula weight for C_20_H_24_N_4_O_2_S: 384.5 g/mol, UPLC-MS: [M + H]^+^ = 385.2, purity = 95%, *R*_t_ = 6.50 min, Y 41= %, mp = 55–60 °C. ^1^H NMR (400 MHz, DMSO) δ 8.66 (d, *J* = 8.5 Hz, 1H, ArH), 8.25–8.20 (m, 2H, ArH, NH), 8.14–8.05 (m, 2H, ArH), 7.91 (t, *J* = 5.7 Hz, 1H, ArH), 7.74–7.60 (m, 3H, ArH), 7.06–6.95 (m, 1H, ArH), 6.51 (t, *J* = 4.7 Hz, 1H, ArH), 3.10 (dt, *J* = 14.4, 7.2 Hz, 2H, AlH), 2.77 (dd, *J* = 12.8, 6.7 Hz, 2H, AlH), 1.32–1.21 (m, 4H, AlH), 1.06 (dd, *J* = 9.7, 6.1 Hz, 4H, AlH). ^13^C NMR (101 MHz, DMSO) δ 162.73 (C_Gua_), 158.27 (ArC), 136.25 (ArC), 134.31 (ArC), 134.05 (ArC), 129.35 (ArC), 128.82 (ArC), 128.19 (ArC), 128.03 (ArC), 127.25 (ArC), 125.19 (ArC), 124.95 (2ArC), 110.06 (ArC), 42.74 (AlH), 40.85 (AlH), 29.47 (AlH), 29.16 (AlH), 26.34 (AlH), 26.15 (AlH). FT-IR: 3276 (N–H, Str), 3083 (C–H Ar, Str), 2918, 2850 (C–H Aliph, Str) 1738 (C–H Ar, Bend), 1679 (C=N, Str), 1587 (N–H, Bend), 1532 (C=C Ar, Str), 1508, 1451 (−CH_2_, Bend), 1365, 1346 (S=O, −SO_2_, Str), 1316 (C–N Ar, Str), 1217, 1200 (C–N amine, Str) 1158, 1130 (S=O, sulfonamide, Str), 1083, 1026 (C–N, Str), 981 (C=C Ar, Bend).

#### 4.1.4 Synthesis of 2-{2-[4-(Naphthalene-1-sulfonyl)piperazin-1-yl]ethyl}-2,3-dihydro-1*H*-isoindole-1,3-dione **7a**

In a round-bottom flask, 1.38 g (5 mmol) of 1-(naphthalene-1-sulfonyl)piperazine **3h** and 1.27 g (5 mmol) of 2-(2-bromoethyl)-1*H*-isoindole-1,3(2*H*)-dione **6a** were mixed. Next, 2.07 g (15 mmol) of K_2_CO_3_ and 25 mL of MeCN were added. The mixture was heated at reflux for 24 h. The progress of the reaction was monitored by TLC. After this time, the solvent was evaporated, water was added to the residue, and the resulting product was filtered. The crude product was then crystallized from methanol to obtain a yield of 74%.

#### 4.1.5 Synthesis of 2-[4-(Naphthalene-1-sulfonyl)piperazin-1-yl]ethan-1-amine **2i**

In a round-bottom flask, 1.66 g (3.7 mmol) of 2-{2-[4-(naphthalene-1-sulfonyl)piperazin-1-yl]ethyl}-2,3-dihydro-1*H*-isoindole-1,3-dione **7a** and 10 mL of 40% aqueous solution of methylamine were mixed. The mixture was stirred for 5 days at room temperature. After this time, 10 mL of 20% NaOH solution was added to the flask, and stirring was continued for 2 h. The solution was extracted with 3 portions of CH_2_Cl_2_. The mixed organic phases were dried over anhydrous MgSO_4_ and then evaporated. The crude product was used directly in the next step.

#### 4.1.6 Synthesis of *N*-{2-[4-(Naphthalene-1-sulfonyl)piperazin-1-yl]ethyl}-3,4-dihydroquinazolin-2-amine **PP 6**

In a round-bottom flask, 0.32 g (1 mmol) of 2-[4-(naphthalene-1-sulfonyl)piperazin-1-yl]ethan-1-amine **2i**, 0.178 g (1 mmol) of 2-(methylsulfanyl)-3, 4-dihydroquinazoline **4a**, and 0.42 mL (3 mmol) of TEA were mixed. The resulting mixture was heated without stirring at 180 °C for 1 h. The progress of the reaction was monitored by TLC. After cooling, 5 mL of H_2_O was added to the mixture, followed by extraction three times with CH_2_Cl_2_. The mixed organic phases were dried over anhydrous MgSO_4_ and then evaporated. The crude product was separated on a column (CHCl_3_/MeOH 9:1).

##### 4.1.6.1 *N*-{2-[4-(Naphthalene-1-sulfonyl)piperazin-1-yl]ethyl}-1,2-dihydroquinazolin-2-amine **PP 6**

Formula weight for C_24_H_27_N_5_O_2_S: 449.6 g/mol, UPLC-MS: [M + H]^+^ = 450.3, purity = 95%, *R*_t_ = 5.19 min, *Y* = 29%, mp = 108–111 °C. Composition: C(64.12%) H(6.05%) N(15.58%) O(7.12%) S(7.13%), found: C(64.25%) H(6.01%) N(15.52%) S(7.08%). ^1^H NMR (400 MHz, DMSO) δ 10.42 (bp, 1H, NH), 8.70 (d, *J* = 8.6 Hz, 1H, ArH), 8.35–8.31 (m, 1H, ArH), 8.16 (dd, *J* = 9.8, 3.6 Hz, 2H, ArH), 7.77–7.67 (m, 3H, ArH), 7.52 (bp, 1H, NH), 7.24–7.13 (m, 2H, ArH), 7.07 (t, *J* = 7.0 Hz, 1H, ArH), 6.93 (d, *J* = 7.7 Hz, 1H, ArH), 4.39 (s, 2H, AlH), 3.34 (s, 6H, AlH), 3.09 (s, 4H, AlH), 2.49–2.46 (m, 2H, AlH). ^13^C NMR (101 MHz, DMSO) δ 152.17 (C_Gua_), 135.20 (ArC), 134.44 (ArC), 133.70 (ArC), 132.07 (ArC), 130.92 (ArC), 129.60 (ArC), 128.83 (ArC), 128.75 (ArC), 128.72 (ArC), 127.50 (ArC), 126.60 (ArC), 125.15 (2ArC), 124.52 (ArC), 118.78 (ArC), 115.68 (ArC), 55.69 (AlC), 55.39 (AlC), 51.98 (AlC), 45.95 (AlC), 41.21 (AlC). FT-IR: 3191(N–H, Str), 2920, 2851 (C–H Aliph, Str) 1738 (C–H Ar, Bend), 1667, 1627 (C=N, Str), 1584 (N–H, Bend), 1495, 1452 (−CH_2_, Bend), 1346, 1324 (S=O, −SO_2_, Str), 1303, 1261 (C–N Ar, Str), 1159, 1129 (S=O, sulfonamide, Str), 1064, 1028 (C–N, Str), 949 (C=C Ar, Bend).

#### 4.1.7 Synthesis of *N*-(Phenylalkyl)-1*H*-1,3-benzimidazol-2-amines **PP 17**–**18** and 2-[4-(Naphthalen-1-yl)piperazin-1-yl]-1*H*-1,3-benzimidazole **PP 20**

In a round-bottom flask, 0.152 g (1 mmol) of 2-chloro-1*H*-1,3-benzimidazole **4e**, 1 mmol of amine **8a**–**b** or piperazine **9a**, 0.414 g of K_2_CO_3_ (3 mmol), and 5 mL of EtOH were mixed. The mixture was heated under reflux for 4 h. The progress of the reaction was monitored by TLC. After the reaction was completed, the solvent was evaporated. Next, 5 mL of H_2_O was added to the residue, and the product was filtered. The crude product was purified by crystallization from ethanol.

##### 4.1.7.1 *N*-Benzyl-1*H*-1,3-benzimidazol-2-amine **PP 17**

Described in ref (38).

##### 4.1.7.2 *N*-(2-Phenylethyl)-1*H*-1,3-benzimidazol-2-amine **PP18**

Described in ref (38).

##### 4.1.7.3 2-[4-(Naphthalen-1-yl)piperazin-1-yl]-1*H*-1,3-benzodiazole **PP 20**

Described in ref (38).

#### 4.1.8 Synthesis of *N*-[2-(Naphthalen-1-yl)ethyl]-3,4-dihydroquinazolin-2-amine **PP 19**

In a round-bottom flask, 0.17 g (1 mmol) of 2-(naphthalen-1-yl)ethan-1-amine **8c** with *z* 0.178 g (1 mmol) of 2-(methylsulfanyl)-3,4-dihydroquinazoline **4a** were taken. Then, 0.42 mL (3 mmol) of TEA was added to the flask. The resulting mixture was heated without stirring at 180 °C for 1 h. The progress of the reaction was monitored by TLC. After cooling, 5 mL of H_2_O was added to the mixture, followed by extraction three times with CH_2_Cl_2_. The mixed organic phases were dried over anhydrous MgSO_4_ and then evaporated. The crude product was separated on a column (CHCl_3_/MeOH 9:1).

##### 4.1.8.1 *N*-[2-(Naphthalen-1-yl)ethyl]-1,4-dihydroquinazolin-2-amine **PP 19**

Described in ref (38).

#### 4.1.9 Synthesis of *N*-[2-(4-Arylpiperazin-1-yl)ethyl]-3,4-dihydroquinazolin-2-amines **PP 21–22**

2-(4-Arylpiperazin-1-yl)ethan-1-amines **10a**–**b** were prepared according to a known procedure. In a round-bottom flask, 1 mmol of 2-(4-arylpiperazin-1-yl)ethan-1-amines **10a**–**b** was mixed with 0.178 g (1 mmol) 2-(methylsulfanyl)-3,4-dihydroquinazoline **4a**. Then, 0.42 mL (3 mmol) of TEA was added to the flask. The resulting mixture was heated without stirring at 180 °C for 1 h. The progress of the reaction was monitored by TLC. After cooling, 5 mL of H_2_O was added to the mixture, followed by extraction three times with CH_2_Cl_2_. The mixed organic phases were dried over anhydrous MgSO_4_ and then evaporated. The crude product was separated on a column (CHCl_3_/MeOH 9:1).

##### 4.1.9.1 *N*-{2-[4-(1,2-Benzothiazol-3-yl)piperazin-1-yl]ethyl}-1,2-dihydroquinazolin-2-amine **PP 21**

Formula weight for C_21_H_24_N_6_S: 392.5 g/mol, UPLC-MS: [M + H]^+^ = 393.2, purity = 95%, *R*_t_ = 4.09 min, *Y* = 65%, mp = 209–213 °C. ^1^H NMR (400 MHz, DMSO) δ 10.63 (bp, 1H, NH), 8.99 (bp, 1H, NH), 8.07 (d, *J* = 8.9 Hz, 2H, ArH), 7.77 (bp, 1H, NH), 7.58 (dt, *J* = 8.2, 5.7 Hz, 1H, ArH), 7.48–7.42 (m, 1H, ArH), 7.28 (t, *J* = 7.7 Hz, 1H, ArH), 7.23 (d, *J* = 7.4 Hz, 1H, ArH), 7.11 (dd, *J* = 7.5, 0.8 Hz, 1H, ArH), 7.07 (dd, *J* = 9.4, 4.1 Hz, 1H, ArH), 4.53 (s, 2H, AlH), 3.50 (d, *J* = 4.4 Hz, 6H, AlH), 3.36 (s, 2H, AlH), 2.69 (d, *J* = 33.0 Hz, 4H, AlH).^13^C NMR (101 MHz, DMSO) δ 163.87 (C_Gua_), 152.44 (ArC), 133.87 (ArC), 128.91 (ArC), 128.37 (ArC), 127.76 (ArC), 126.70 (ArC), 124.92 (ArC), 124.59 (ArC), 124.55 (ArC), 121.56 (ArC), 118.95 (ArC), 115.79, (ArC) 56.64 (AlC), 52.69 (AlC), 50.04 (AlC), 41.31 (AlC). FT-IR: 3204 (N–H, Str), 3089 (C–H Ar, Str), 2997, 2945, 2849, 2779 (C–H Aliph, Str), 1738 (C–H Ar, Bend), 1675 (C=N, Str), 1616, 1576 (N–H, Bend), 1493, 1449, 1423 (−CH_2_, Bend), 1382, 1365, 1352 (S=O, −SO_2_, Str), 1273, 1263 (C–N Ar, Str), 1208 (C–N amine, Str) 1167, 1145, 1120 (S=O, sulfonamide, Str), 1094, 1066, 1023 (C–N, Str), 959 (C=C Ar, Bend).

##### 4.1.9.2 *N*-{2-[4-(1-Benzothiophen-4-yl)piperazin-1-yl]ethyl}-1,2-dihydroquinazolin-2-amine **PP 22**

Formula weight for C_22_H_25_N_5_S: 391.5 g/mol, UPLC-MS: [M + H]^+^ = 392.2, purity = 100%, *R*_t_ = 4.44 min, *Y* = 62%, mp = 201–204 °C. Composition: C(67.49%) H(6.44%) N(17.89%) S(8.19%), found: C(67.44%) H(6.49%) N(17.88%) S(8.12%). ^1^H NMR (400 MHz, DMSO) δ 10.62 (bp, 1H, NH), 8.99 (bp, 1H, NH), 7.78 (bp, 1H, NH), 7.72 (d, *J* = 5.5 Hz, 1H), 7.64 (d, *J* = 8.1 Hz, 1H, ArH), 7.42 (d, *J* = 5.5 Hz, 1H, ArH), 7.29 (td, *J* = 7.9, 4.2 Hz, 2H, ArH), 7.23 (d, *J* = 7.2 Hz, 1H, ArH), 7.13–7.02 (m, 2H, ArH), 6.91 (d, *J* = 7.6 Hz, 1H, ArH), 4.53 (s, 2H, AlH), 3.48 (s, 2H, AlH), 3.12 (s, 4H, AlH), 2.76 (s, 4H, AlH), 2.67 (s, 2H, AlH). ^13^C NMR (101 MHz, DMSO) δ 152.49(C_Gua_), 148.48 (ArC), 140.92 (ArC), 133.89 (ArC), 133.84 (ArC), 128.91 (ArC), 126.70 (ArC), 126.46 (ArC), 125.56 (ArC), 124.56 (ArC), 122.31 (ArC), 118.98 (ArC), 117.29 (ArC), 115.83 (ArC), 112.58 (ArC), 70.25 (AlC), 56.68 (AlC), 53.18 (2AlC), 52.06 (2AlC), 41.32 (AlC). FT-IR: 3185 (N–H, Str), 3048 (C–H Ar, Str), 2996, 2942, 2826 (C–H Aliph, Str), 1667, 1624 (C=N, Str), 1580, 1564 (N–H, Bend), 1495, 1450, 1414 (−CH_2_, Bend), 1371, 1344 (S=O, −SO_2_, Str), 1284, 1264 (C–N Ar, Str), 1236, 1208 (C–N amine, Str) 1136 (S=O, sulfonamide, Str), 1024 (C–N, Str), 970 (C=C Ar, Bend).

#### 4.1.10 Synthesis of N1-(1,2-Benzothiazol-3-yl)hexane-1,6-diamine **10c**

In a round-bottom flask, 2.16 g (10 mmol) of *tert*-butyl (6-aminohexyl)carbamate **1b**, 2.14 g (10 mmol) of 3-bromo-1,2-benzothiazole **11**, 4.14 g (30 mmol) of K_2_CO_3_, and 40 mL of MeCN were mixed. The mixture was heated at reflux for 24 h. The progress of the reaction was monitored by TLC. After this time, the solvent was evaporated, water was added to the residue and extracted three times with CH_2_Cl_2_. The mixed organic phases were dried over anhydrous MgSO_4_ and then evaporated. The crude product was separated on a column (CHCl_3_/MeOH 99:1). Next, 5 mL of 17% HCl was added to the residue. The resulting mixture was heated at 80 °C for 1 h. After this time, the contents of the flask were neutralized with a saturated sodium bicarbonate solution and then extracted with CH_2_Cl_2_. After evaporation, amine **10c** was used directly for the next step.

#### 4.1.11 Synthesis of N6-(1,2-Benzothiazol-3-yl)-N1-(3,4-dihydroquinazolin-2-yl)hexane-1,6-diamine **PP 23**

In a round-bottom flask, 0.25 g (1 mmol) of N1-(1,2-benzothiazol-3-yl)hexane-1,6-diamine **10c**, 0.178 g (1 mmol) of 2-(methylsulfanyl)-3,4-dihydroquinazoline **4a**, and 0.42 mL (3 mmol) of TEA were taken. The resulting mixture was heated without stirring at 180 °C for 1 h. The progress of the reaction was monitored by TLC. After cooling, 5 mL of H_2_O was added to the mixture, followed by extraction three times with CH_2_Cl_2_. The mixed organic phases were dried over anhydrous MgSO_4_ and then evaporated. The crude product was separated on a column (CHCl_3_/MeOH 9:1).

##### 4.1.11.1 *N*6-(1,2-Benzothiazol-3-yl)-*N*1-(3,4-dihydroquinazolin-2-yl)hexane-1,6-diamine **PP 23**

Formula weight for C_21_H_25_N_5_S: 379.5 g/mol, UPLC-MS: [M + H]^+^ = 380.2, purity = 99%, *R*_t_ = 6.06 min, *Y* = % mp = 67–73 °C. ^1^H NMR (400 MHz, DMSO) δ 10.39 (bp, 1H, NH), 8.41 (bp, *J* = 64.0 Hz, 1H, NH), 8.14 (d, *J* = 8.1 Hz, 1H, ArH), 8.02 (bp, 1H, NH), 7.91 (d, *J* = 8.1 Hz, 1H, ArH), 7.54–7.47 (m, 1H, ArH), 7.45–7.32 (m, 2H, ArH), 7.30–7.23 (m, 1H, ArH), 7.21 (d, *J* = 7.2 Hz, 1H, ArH), 7.09 (bp, *J* = 7.5 Hz, 2H,, ArH, NH), 4.49 (s, 2H, AlH), 3.42 (dd, *J* = 12.9, 6.7 Hz, 2H, AlH), 3.28 (dd, *J* = 12.6, 6.5 Hz, 2H, AlH), 1.72–1.63 (m, 2H, AlH), 1.61–1.52 (m, 2H, AlH), 1.45–1.36 (m, 4H, AlH). ^13^C NMR (101 MHz, DMSO) δ 159.97 (C_Gua_), 151.98 (ArC), 150.84 (ArC), 133.85 (ArC), 128.87 (ArC), 128.47 (ArC), 127.08 (ArC), 126.63 (ArC), 124.50 (ArC), 124.24 (ArC), 122.97 (ArC), 120.81 (ArC), 118.95 (ArC), 115.83 (ArC), 42.68 (AlC), 41.51 (AlC), 41.34 (AlC), 29.23 (AlC), 28.95 (AlC), 26.73 (AlC), 26.35 (AlC). FT-IR: 3195 (N–H, Str), 3056 (C–H Ar, Str), 2927, 2854 (C–H Aliph, Str), 1738 (C–H Ar, Bend), 1664, 1628 (C=N, Str), 1589 (N–H, Bend), 1494, 1452, 1428 (−CH_2_, Bend), 1366, 1346 (S=O, −SO_2_, Str), 1316, 1301 (C–N Ar, Str), 1259, 1208 (C–N amine, Str) 1173, 1157 (S=O, sulfonamide, Str), 1073, 1033 (C–N, Str), 942 (C=C Ar, Bend).

#### 4.1.12 Synthesis of 3-[6-(1,3-Dioxo-2,3-dihydro-1*H*-isoindol-2-yl)hexyl]-2λ^6^-thia-3-azatricyclo[6.3.1.0^4^,^12^]dodeca-1(11),4(12),5,7,9-pentaene-2,2-dione **7d**

To a round-bottom flask, 3.1 g (10 mmol) of 2-(6-bromohexyl)-1*H*-isoindole-1,3(2*H*)-dione **6b**, 2.05 g (10 mmol) of 1λ6-naphtho[1,8-cd][1, 2]thiazole-1,1(2*H*)-dione **12**, 4.14 g (30 mmol) of K_2_CO_3_, and 50 mL of MeCN were added. The mixture was heated at reflux for 24 h. The progress of the reaction was monitored by TLC. After this time, the solvent was evaporated, H_2_O was added to the residue, and the resulting product was filtered off, which was then crystallized in a round-bottom flask by adding methanol to obtain a yield of 68%.

#### 4.1.13 Synthesis of 2-(6-Aminohexyl)-1λ6-naphtho[1,8-cd][1,2]thiazole-1,1(2*H*)-dione **10d**

To a round-bottom flask, 2.924 g (6.7 mmol) of 3-[6-(1,3-dioxo-2,3-dihydro-1*H*-isoindol-2-yl)hexyl]-2λ^6^-thia-3-azatricyclo[6.3.1.0^4^,^12^]dodeca-1(11),4(12),5,7,9-pentaene-2,2-dione **7d**, and 20 mL of 40% aqueous solution of methylamine were added. The mixture was stirred for 5 days at room temperature. After this time, 20 mL of 20% NaOH solution was added to the flask, and stirring was continued for 2 h. The solution was extracted with 3 portions of CH_2_Cl_2_. The mixed organic phases were dried over anhydrous MgSO_4_ and then evaporated. The crude product was used directly in the next step.

#### 4.1.14 Synthesis of 3-{6-[(3,4-Dihydroquinazolin-2-yl)amino]hexyl}-2λ^6^-thia-3-azatricyclo[6.3.1.0^4^,^12^]dodeca-1(11),4(12),5,7,9-pentaene-2,2-dione **PP 24**

In a round-bottom flask, 0.31 g (1 mmol) of 2-(6-aminohexyl)-1λ6-naphtho[1,8-cd][1,2]thiazole-1,1(2*H*)-dione **10d**, 0.178 g (1 mmol) of 2-(methylsulfanyl)-3,4-dihydroquinazoline **4a**, and 0.42 mL (3 mmol) of TEA were taken. The resulting mixture was heated without stirring at 180 °C for 1 h. The progress of the reaction was monitored by TLC. After cooling, 5 mL of H_2_O was added to the mixture, followed by extraction three times with CH_2_Cl_2_. The mixed organic phases were dried over anhydrous MgSO_4_ and then evaporated. The crude product was separated on a column (CHCl_3_/MeOH 9:1).

##### 4.1.14.1 3-{6-[(1,2-Dihydroquinazolin-2-yl)amino]hexyl}-2λ^6^-thia-3-azatricyclo[6.3.1.0^4^,^12^]dodeca-1(11),4(12),5,7,9-pentaene-2,2-dione **PP 24**

Formula weight for C_24_H_26_N_4_O_2_S: 434.6 g/mol, UPLC-MS: [M + H]^+^ = 435.3, purity = 99%, *Y* = 68%, *R*_t_ = 6.30 min, mp = 192–195 °C. Composition: C(66.33%) H(6.03%) N(12.89%) O(7.36%) S(7.38%), found: C(66.31%) H(6.07%) N(12.85%) S(7.33%). ^1^H NMR (400 MHz, DMSO) δ 10.29 (bp, 1H, NH), 8.43 (bp, 1H, NH), 8.30 (d, *J* = 8.1 Hz, 1H, ArH), 8.23 (d, *J* = 7.0 Hz, 1H, ArH), 7.95 (bp, 1H, NH), 7.89 (dd, *J* = 8.1, 7.3 Hz, 1H, ArH), 7.68–7.59 (m, 2H, ArH), 7.26 (t, *J* = 7.7 Hz, 1H, ArH), 7.21 (d, *J* = 6.9 Hz, 1H, ArH), 7.14–6.98 (m, 3H, ArH), 4.48 (s, 2H, AlH), 3.85 (t, *J* = 7.3 Hz, 2H, AlH), 3.27 (dd, *J* = 12.5, 6.4 Hz, 2H, AlH), 1.89–1.80 (m, 2H, AlH), 1.56 (dt, *J* = 14.7, 7.5 Hz, 2H, AlH), 1.51–1.34 (m, 4H, AlH). ^13^C NMR (101 MHz, DMSO) δ 151.91(C_Gua_), 136.23 (ArC), 133.82 (ArC), 132.03 (ArC), 130.58 (ArC), 130.22 (ArC), 130.19 (ArC), 129.17 (ArC), 128.87 (ArC), 126.62 (ArC), 124.52 (ArC), 120.71 (ArC), 118.97 (ArC), 118.64 (ArC), 118.49 (ArC), 115.86 (ArC), 104.34 (ArC), 41.88 (AlC), 41.45 (AlC), 41.35 (AlC), 28.81 (AlC), 28.00 (AlC), 26.29 (AlC), 26.08 (AlC). FT-IR:3196 (N–H, Str), 3087, 3015 (C–H Ar, Str), 2936, 2868 (C–H Aliph, Str), 1739 (C–H Ar, Bend), 1670, 1629 (C=N, Str), 1590 (N–H, Bend), 1493, 1461, 1421 (−CH_2_, Bend), 1372, 1349 (S=O, −SO_2_, Str), 1307 (C–N Ar, Str), 1260, 1224 (C–N amine, Str) 1188, 1169, 1127 (S=O, sulfonamide, Str), 1048 (C–N, Str), 982, 927 (C=C Ar, Bend).

### 4.2 Radioligand Binding Assays

The detailed procedure is presented in previous publications.^53^ Assays were performed by incubating for 1 h at 37 °C (room temperature for 5-HT_1A_) in 200 μL volumes on 96-well plates. The reaction was terminated by rapid filtration using Unifilter plates, and radioactivity on the filters was measured with a PerkinElmer Microbeta plate reader. Radioligands used were:

5-HT_6_: 2 nM [^3^H]-LSD

5-HT_1A_: 2.5 nM [^3^H]-8-OHDPAT

5-HT_5A_: 3.5 nM [^3^H]-LSD

5-HT_7_: 0.8 nM [^3^H]-5-CT

D_2_: 2.5 nM [^3^H]-raclopride

Eight concentrations (10^–4^ to 10^–11^ M) were tested in triplicate for each compound. Inhibition constants (*K*_i_) were calculated using the Cheng–Prusoff equation.^54^ The results are expressed as the means of at least two independent experiments.

#### 4.2.1 Functional Evaluation

The detailed procedure is presented in previous publications.^53^ The functional activity of the compounds was assessed using the LANCE Ultra cAMP detection kit (PerkinElmer). G_i_-coupled receptors (D_2_ and 5-HT_1A_) were activated with 1 μM forskolin (EC_90_), and antagonist activity at D_2_R was tested with 10 nM dopamine. For other receptors (5-HT_7_R, 5-HT_1A_R, and 5-HT_6_R), 5-CT was used at 10, 100, and 1000 nM, respectively. Each compound was tested in triplicate across eight concentrations. The inhibitory constant (Kb) for each antagonist was calculated using the Cheng–Prusoff equation.

### 4.3 Cell Culture Conditions and Cytotoxicity Assessment

The detailed procedure is presented in previous publications.^53^ Briefly, the study utilized various human cancer and normal cell lines, including LN-229 (glioblastoma), MCF7 (breast carcinoma), HepG2 (hepatoma), U-251 (glioblastoma), U87MG (glioblastoma), AsPC-1 (pancreatic adenocarcinoma), and NHA (normal human astrocytes). Cells were cultured in appropriate media, supplemented with FBS, antibiotics, and other supplements, and incubated at 37 °C with 5% CO_2_. Compounds were dissolved in DMSO, prepared at various concentrations (1–25 μM), and applied to the cells for 72 h. Cell viability was assessed using the MTS assay, and the inhibitory concentration (IC_50_) was calculated using GraphPad Prism 8.0. Each compound was tested in triplicate across multiple experiments.

### 4.4 Lipophilicity and Affinity to Phospholipids^55^

The detailed procedure is presented in previous publications.^53^ High-performance liquid chromatography (HPLC) analyses were conducted using the Prominence-1 LC-2030C 3D system (Shimadzu) with a DAD detector, employing three different chromatographic columns with specific stationary phases and mobile phases. The analysis was performed at 30 °C with a flow rate of 1.5 mL/min for IAM and C18 columns, and 0.9 mL/min for the Chiralpak column. Calibration standards for the columns were sourced from various suppliers and dissolved in dimethyl sulfoxide. Detection occurred in the UV range (190–300 nm) with a 5 μL injection volume. Each compound was analyzed in triplicate. Retention times for the tested compounds and calibration standards are provided in the Supporting Information (Tables S1 and S2).

### 4.5 ADMET Tests

The ADMET properties of compounds were evaluated using a series of tests, described in refs (53,56). Passive permeability was assessed using the PAMPA Plate System, measuring the concentration of test substances in the donor and acceptor wells via LC/MS to calculate Pe values.^57^ The impact on CYP3A4 and CYP2D6 activity was tested using bioluminescent P450-Glo assays, with reference compounds KE and QD, and results were measured using a microplate reader. The stability of compounds in mouse liver microsomes was tested by incubating them with the reaction buffer for 120 min, followed by analysis using LC/MS. Hepatotoxicity was evaluated using an MTS assay in HepG2 human hepatoma cells, comparing the compounds to doxorubicin as a control.^58,59^ Plasma protein binding was assessed using the TRANSILXL PPB assay, with compounds incubated with α_1_-acid glycoprotein and human serum albumin, followed by LC/MS analysis.

#### 4.5.1 Statistical Analysis

Data were analyzed using GraphPad Prism using ANOVA and Bonferroni’s test, with significance set at *p* −drug interactions and hepatotoxicity, and *p* < 0.05 for other tests.

#### 4.5.2 *In Vivo* Cardiotoxicity and Neurotoxicity

The toxicity of compounds was evaluated using two tests: the fish embryo toxicity (FET) test and neurotoxicity assessment.^53^ Zebrafish embryos were exposed to different concentrations of the compounds in the E3 medium for 96 h. Acute toxicity was assessed by monitoring signs such as coagulation of fertilized eggs, somite formation absence, tailbud separation, and lack of heartbeat. The LD_50_ value was calculated, and cardiovascular effects were monitored through heartbeat observations.

A locomotor activity test was conducted on zebrafish larvae (4 days postfertilization). After determining the maximum tolerated concentration (MTC), larvae were exposed to selected concentrations of **PP 10** and **PP 15** for 24 h. The distance traveled by the larvae was measured in both light and dark conditions using EthoVision XT15 software, and the mean distance per minute was calculated for each condition to assess locomotor activity. These tests provided data on both acute toxicity and potential neurotoxic effects of the compounds.

### 4.6 Molecular Modeling

The molecular modeling studies focused on several computational techniques to investigate protein–ligand interactions and predict the behavior of compounds. The detailed procedure is presented in previous publications.^53^ The docking process involved the use of the Schrödinger software suite, including the Epik method for ionization state prediction and the Protein Preparation Wizard for optimizing protein structures.^60,61^ The induced fit docking (IFD) method was employed, where receptor conformations were refined, and up to 80 positions per ligand were generated. The Glide algorithm was used for flexible ligand docking, and only coherent binding modes were retained for final validation. FMO-EDA calculations were performed on selected protein–ligand complexes to analyze interaction energies and the contributions of various forces such as electrostatics, dispersion, charge transfer, exchange repulsion, and Gibbs solvation energy.^62−64^ The calculations were done using the GAMESS software and PCM model for solvation.^65^ Molecular dynamics (MD) simulations were conducted using the NAMD program, using the CHARMM force field. The system included a membrane environment and was built using the QwikMD tool in VMD. Simulations incorporated a 15 Å buffer, 0.15 mol/L NaCl salt concentration, explicit solvent, and POPC lipids.^66,67^ A 3D-QSAR model was developed using the Maestro Schrodinger Suite’s QSAR tool. This method linked known activities to molecular components, using Gaussian equations to calculate interaction energies. The model was optimized using partial least-squares (PLS) analysis and validated via cross-validation. Additionally, a pharmacophore model for the 5-HT_6_ receptor was created, assessing predictive power with regression and cross-validation coefficients.^68−70^

## Supporting Information Available

All obtained UPLC-MS, ^1^H NMR, ^13^C NMR, FT-IR data; additional information from biological activity tests; and biochromatographic data (PDF)
