# Supplementary material for: Long-Chain Cyclic Arylguanidines as Multifunctional Serotonin Receptor Ligands with Antiproliferative Activity
Source: ACS Omega. 2025 Feb 11;10(7):6446–69. doi: 10.1021/acsomega.4c06456 (PMC11866022; doi:10.1021/acsomega.4c06456)
Supplement: Supplementary file 1 — ao4c06456_si_001.pdf [file ao4c06456_si_001.pdf]

Long-chain cyclic arylguanidines as multifunctional serotonin receptors ligands with antiproliferative activity

Przemysław Zaręba*[a], Anna K. Drabczyk[b], Artur Wnorowski[c], Maciej Maj[c], Patryk Rurka[d], Katarzyna Malarz[d,e], Gniewomir Latacz[f], Krystyna Nędza[g], Krzesimir Ciura[h,i], Katarzyna Ewa Greber[h], Anna Boguszewska-Czubara[j], Paweł Śliwa[b], Julia Kuliś[a]

[a] *Faculty of Chemical Engineering and Technology, Department of Chemical Technology and Environmental Analytics, Cracow University of Technology, 24 Warszawska Street, 31-155 Cracow, Poland*
[b] *Faculty of Chemical Engineering and Technology, Department of Organic Chemistry and Technology, Cracow University of Technology, 24 Warszawska Street, 31-155 Cracow, Poland*
[c] *Department of Biopharmacy, Medical University of Lublin, 4a Chodźki Street, 20-093 Lublin, Poland*
[d] *Institute of Physics, University of Silesia in Katowice, 1A 75 Pułku Piechoty Street, 41-500 Chorzow, Poland*
[e] *Department of Systems Biology and Engineering, Silesian University of Technology, 11 Akademicka Street, 44-100 Gliwice, Poland*
[f] *Department of Technology and Biotechnology of Drugs, Jagiellonian University Medical College, 9 Medyczna Street, 30-688 Cracow, Poland*
[g] *Department of Medicinal Chemistry, Maj Institute of Pharmacology – Polish Academy of Sciences, 12 Smętna Street, 31-343 Cracow, Poland*
[h] *Department of Physical Chemistry, Faculty of Pharmacy, Medical University of Gdansk, 80-416 Gdansk, Poland*
[I] *Laboratory of Environmental Chemoinformatics, Faculty of Chemistry, University of Gdansk, 63 Wita Stwosza Street, 80-308 Gdansk, Poland*
[i] *Department of Medical Chemistry, Medical University of Lublin, 4a Chodźki Street, 20-093 Lublin, Poland*
**przemyslaw.zareba@pk.edu.pl, phone +48 126282790*

Supplementary materials - Structural analyses

Table of contents

2-[4-(naphthalene-2-sulfonyl)piperazin-1-yl]-3,4-dihydroquinazoline PP 1 2

N-{2-[(3,4-dihydroquinazolin-2-yl)amino]ethyl}naphthalene-1-sulfonamide PP 4 4

2-[4-(naphthalene-1-sulfonyl)piperazin-1-yl]-3,4-dihydroquinazoline PP 5 6

N-{2-[4-(naphthalene-1-sulfonyl)piperazin-1-yl]ethyl}-3,4-dihydroquinazolin-2-amine PP 6 8

N-{4-[(3,4-dihydroquinazolin-2-yl)amino]butyl}naphthalene-1-sulfonamide PP 7 10

N-{6-[(3,4-dihydroquinazolin-2-yl)amino]hexyl}naphthalene-1-sulfonamide PP 8 12

N-{2-[(4-methyl-3,4-dihydroquinazolin-2-yl)amino]ethyl}naphthalene-1-sulfonamide PP 9 13

N-{6-[(4-methyl-3,4-dihydroquinazolin-2-yl)amino]hexyl}naphthalene-1-sulfonamide PP 10 15

N-{6-[(5-fluoro-4-methyl-3,4-dihydroquinazolin-2-yl)amino]hexyl}naphthalene-1-sulfonamide PP 11 18

N-{6-[(6,8-dichloro-4-methyl-3,4-dihydroquinazolin-2-yl)amino]hexyl}naphthalene-1-sulfonamide PP 12 20

2-chloro-*N*-{6-[(4-methyl-3,4-dihydroquinazolin-2-yl)amino]hexyl}naphthalene-1-sulfonamide PP 13 22

N-{2-[(quinazolin-2-yl)amino]ethyl}naphthalene-1-sulfonamide PP 14 24

N-{6-[(quinazolin-2-yl)amino]hexyl}naphthalene-1-sulfonamide PP 15 26

N-{6-[(pyrimidin-2-yl)amino]hexyl}naphthalene-1-sulfonamide PP 16 29

N-{2-[4-(1,2-benzothiazol-3-yl)piperazin-1-yl]ethyl}-3,4-dihydroquinazolin-2-amine PP 21 31

N-{2-[4-(1-benzothiophen-4-yl)piperazin-1-yl]ethyl}-3,4-dihydroquinazolin-2-amine PP 22 33

N^6-(1,2-benzothiazol-3-yl)-N^1-(3,4-dihydroquinazolin-2-yl)hexane-1,6-diamine PP 23 35

3-{6-[(3,4-dihydroquinazolin-2-yl)amino]hexyl}-2λ^6-thia-3-azatricyclo[6.3.1.$0^{4,12}$]dodeca-1(11),4(12),5,7,9-pentaene-2,2-dione PP 24 37

2-[4-(naphthalene-2-sulfonyl)piperazin-1-yl]-3,4-dihydroquinazoline PP 1

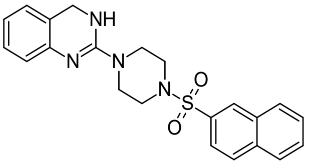

Figure S1. Structure of **PP 1**

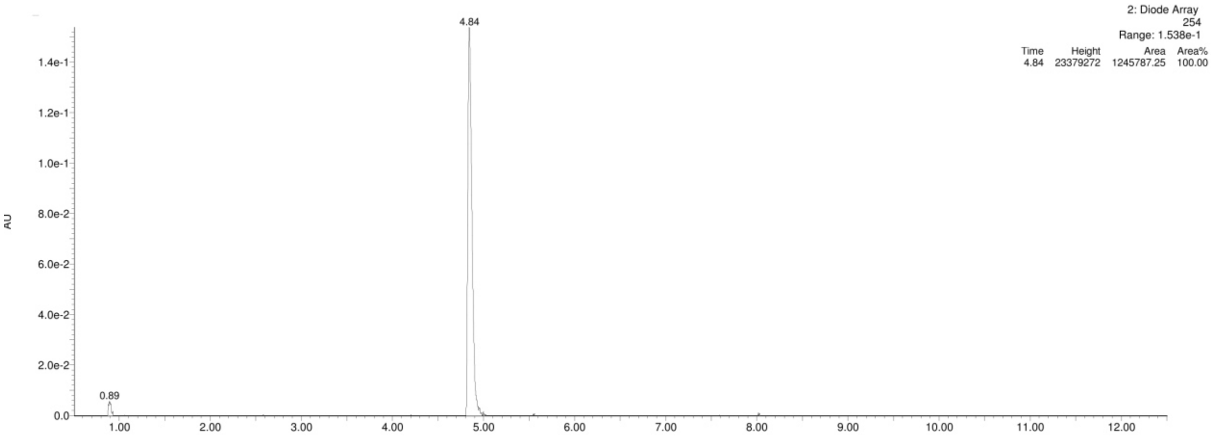

Figure S2. HPLC of **PP 1**

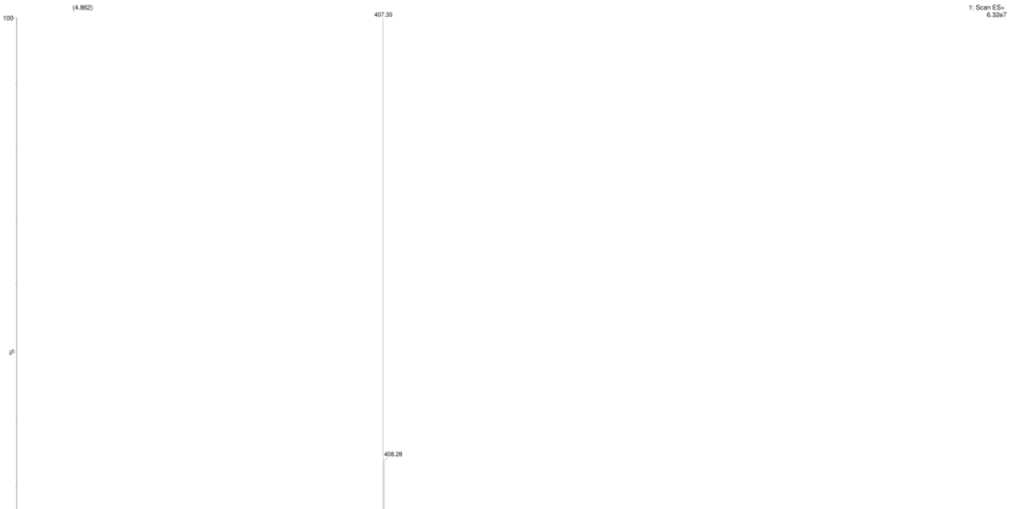

Figure S3. MS of **PP 1**

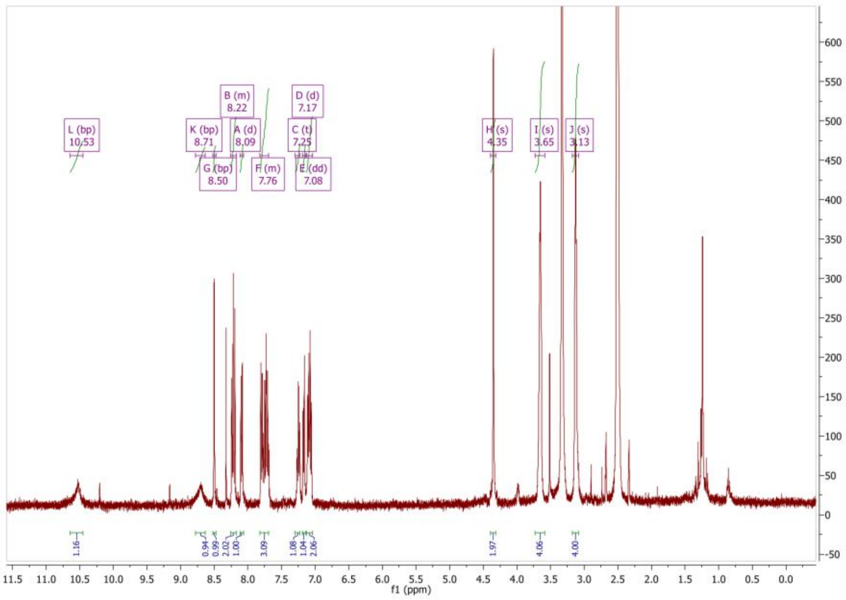

Figure S4. ^{1}H NMR of **PP 1**

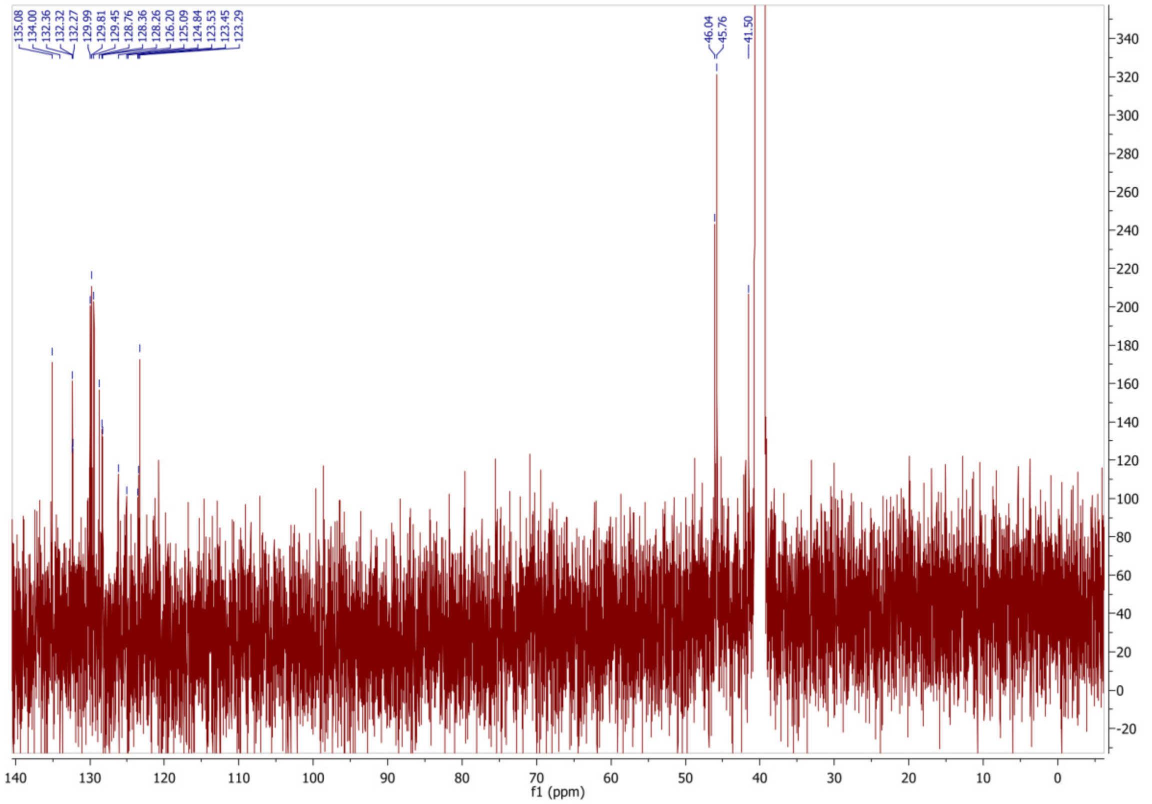

Figure S5. ^{13}C NMR of **PP 1**

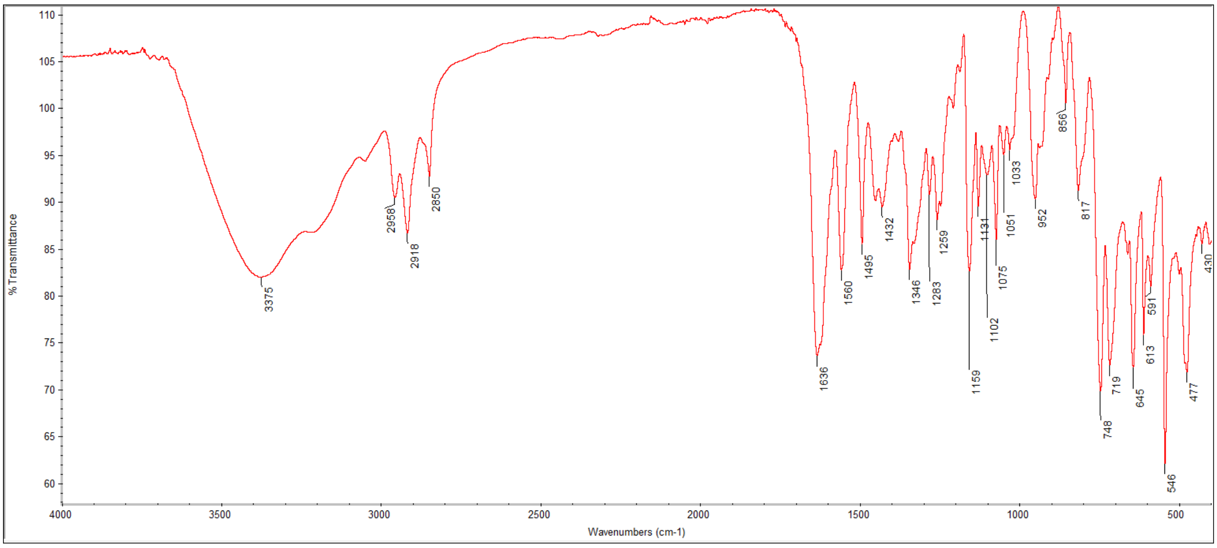

Figure S6. FT IR of **PP 1**

N-{2-[(3,4-dihydroquinazolin-2-yl)amino]ethyl}naphthalene-1-sulfonamide PP 4

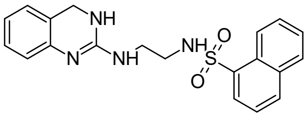

Figure S7. Structure of **PP 4**

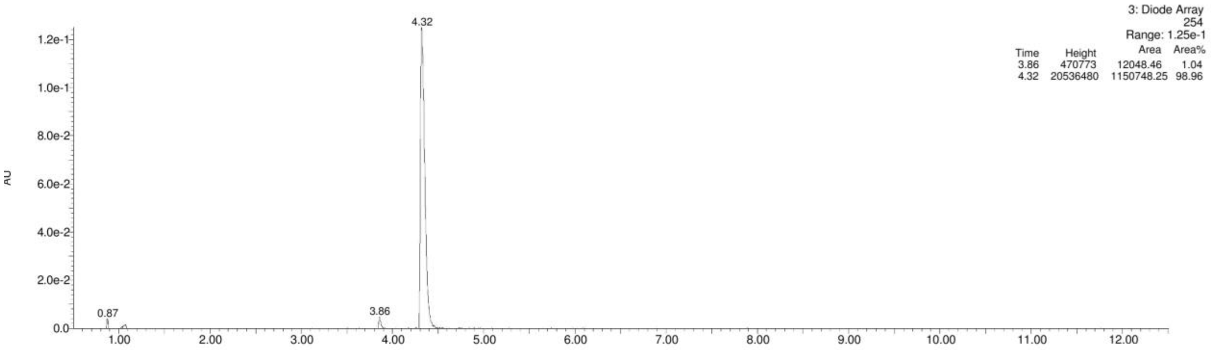

Figure S8. HPLC of **PP 4**

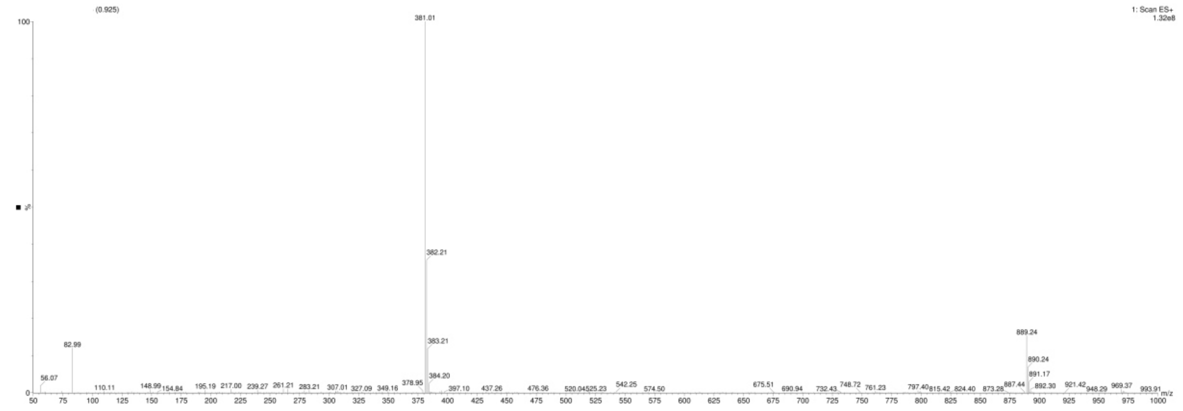

Figure S9. MS of **PP 4**

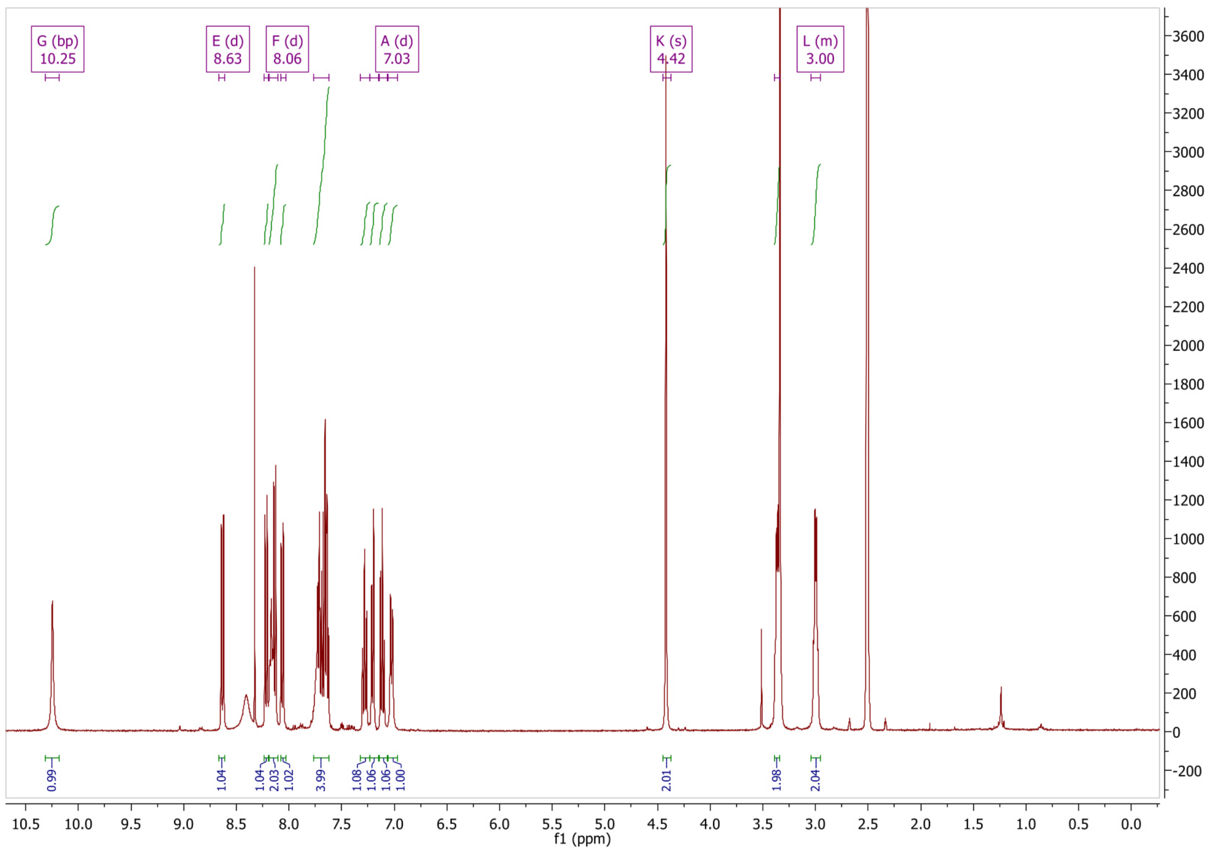

Figure S10. ^{1}H NMR of **PP 4**

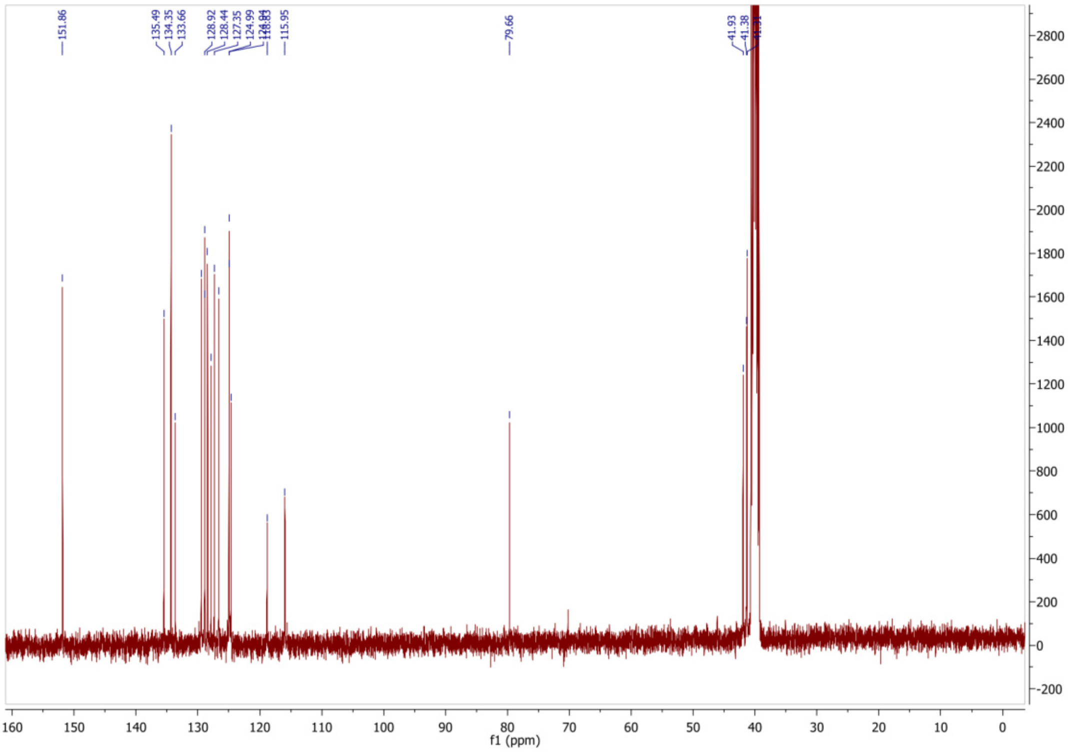

Figure S11. ^{13}C NMR of **PP 4**

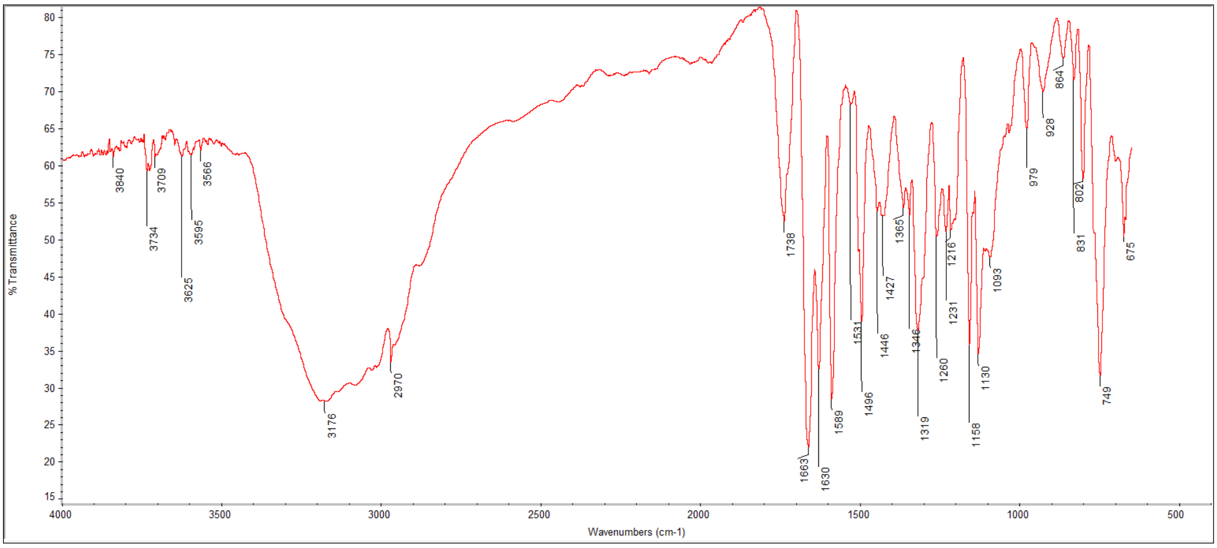

Figure S12. FT IR of **PP 4**

2-[4-(naphthalene-1-sulfonyl)piperazin-1-yl]-3,4-dihydroquinazoline PP 5

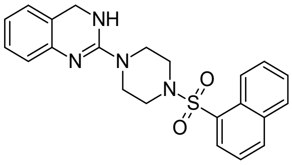

Figure S13. Structure of **PP 5**

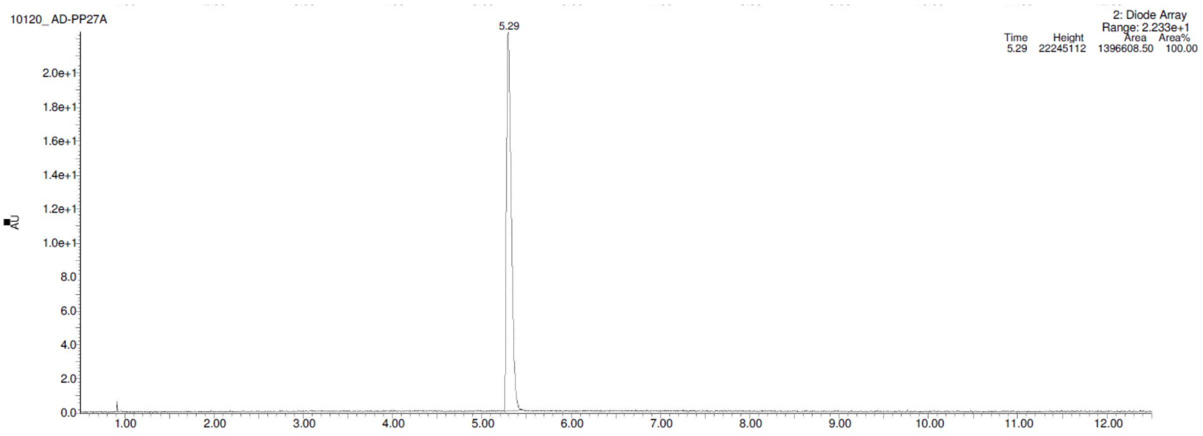

Figure S14. HPLC of **PP 5**

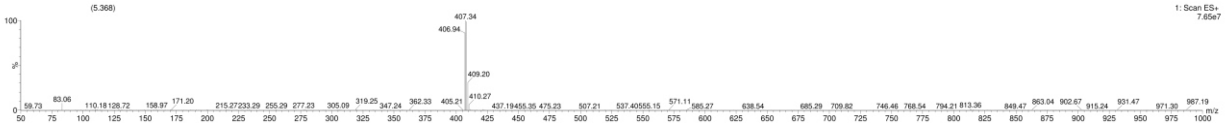

Figure S15. MS of **PP 5**

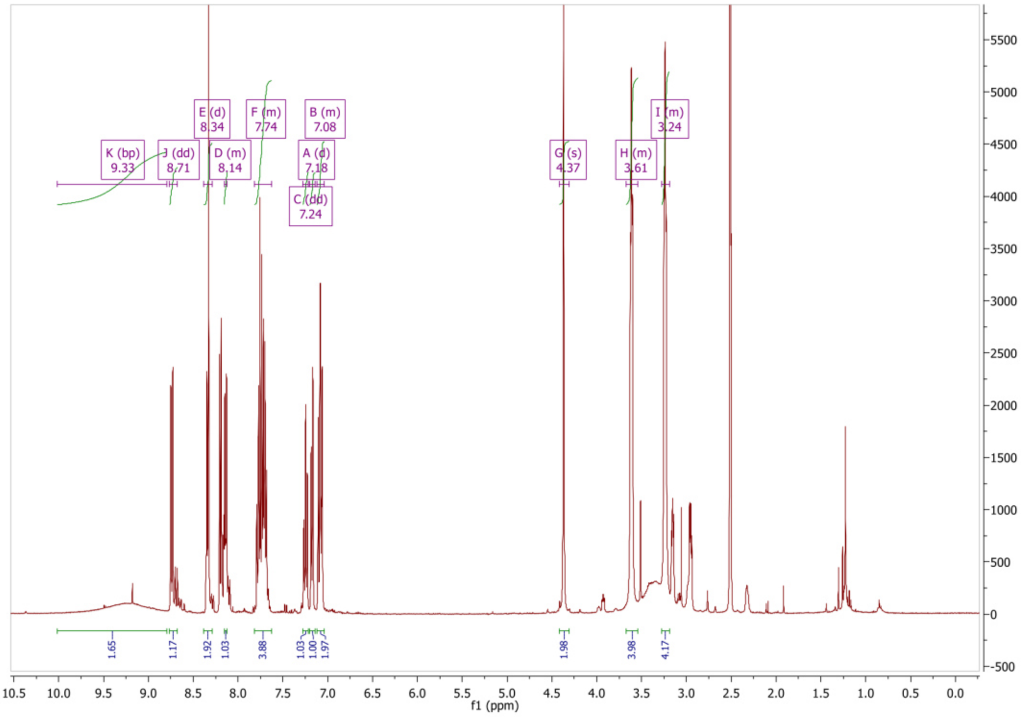

Figure S16. ^{1}H NMR of **PP 5**

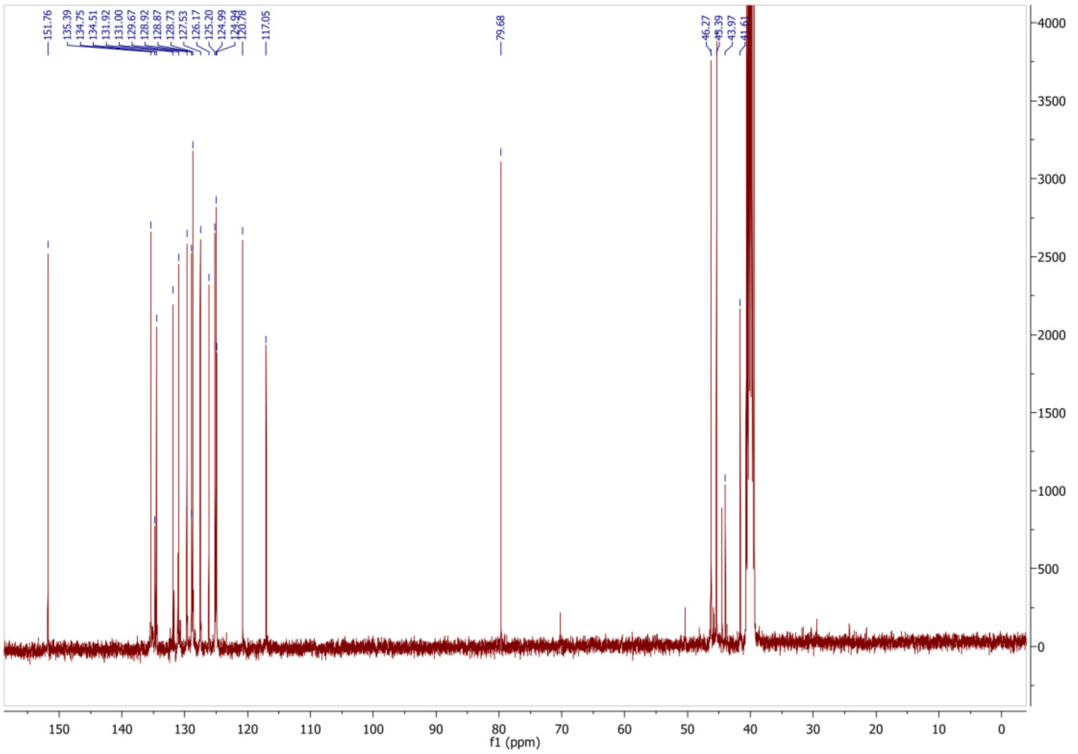

Figure S17. ^{13}C NMR of **PP 5**

***N*-{2-[4-(naphthalene-1-sulfonyl)piperazin-1-yl]ethyl}-3,4-dihydroquinazolin-2-amine PP 6**

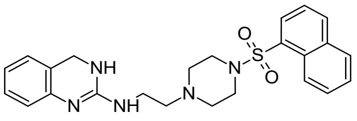

Figure S18. Structure of **PP 6**

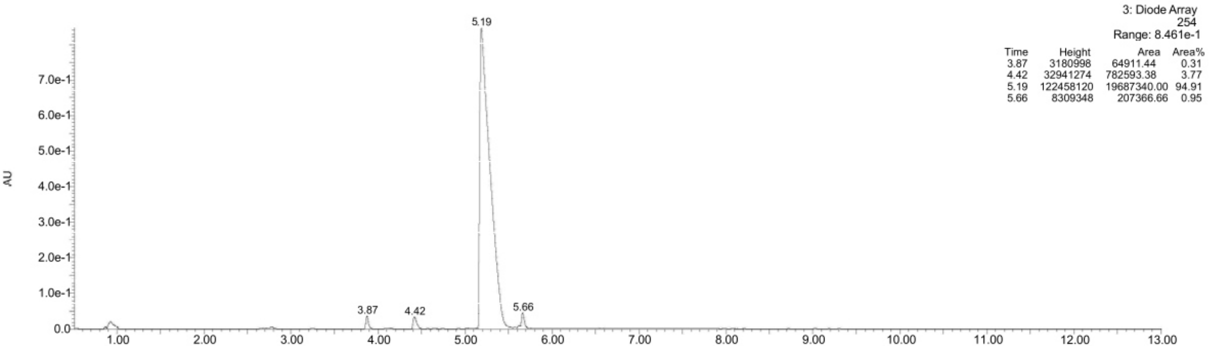

Figure S19. HPLC of **PP 6**

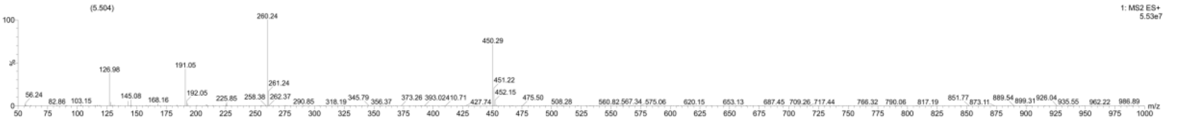

Figure S20. MS of **PP 6**

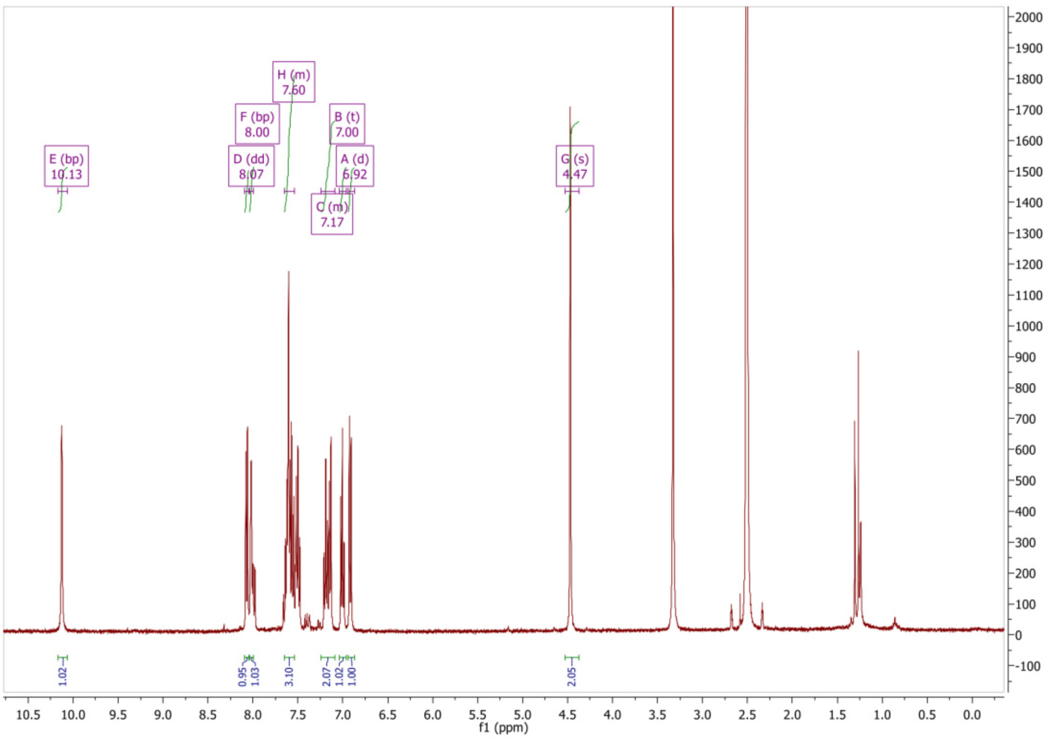

Figure S21. ^{1}H NMR of **PP 6**

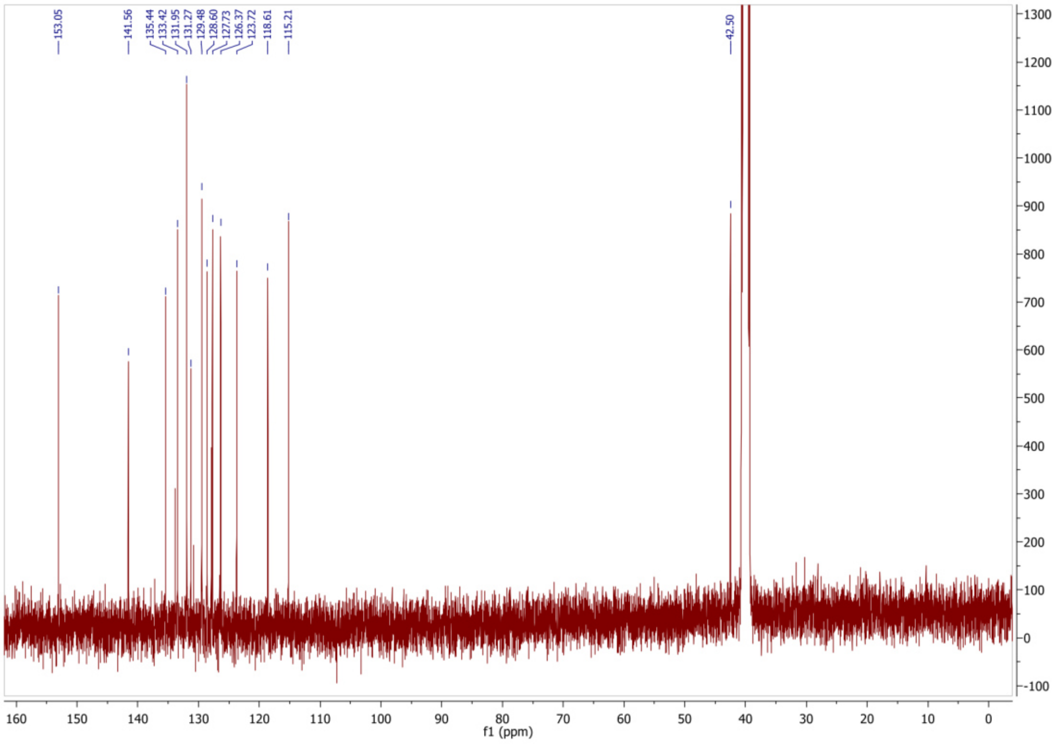

Figure S22. ^{13}C NMR of **PP 6**

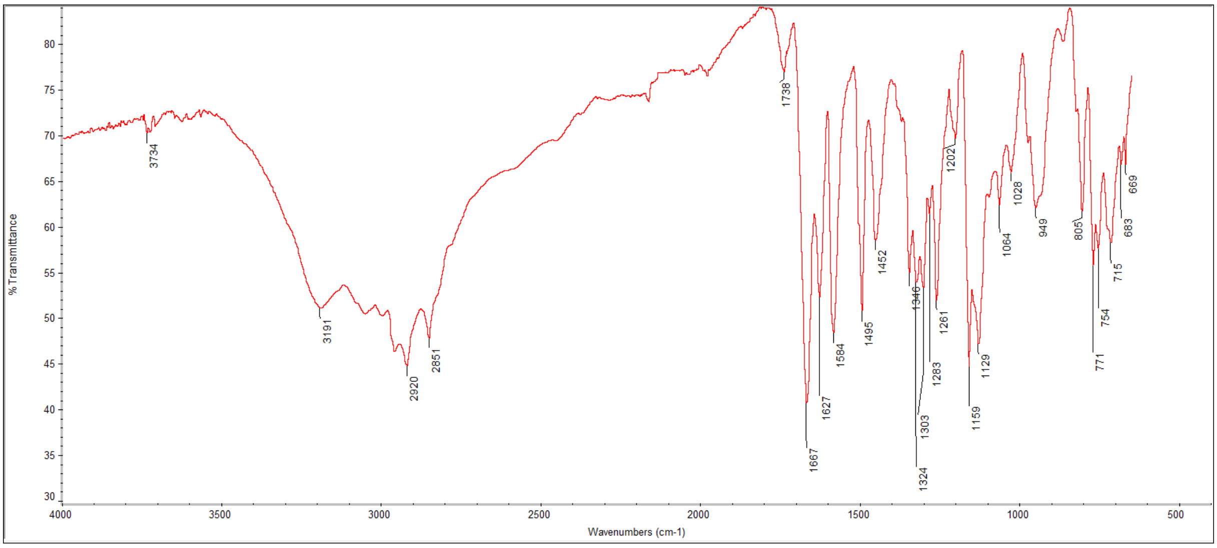

Figure S23. FT IR of **PP6**

***N*-{4-[(3,4-dihydroquinazolin-2-yl)amino]butyl}naphthalene-1-sulfonamide PP 7**

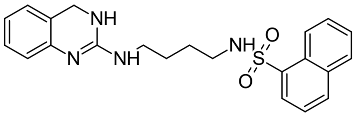

Figure S24. Structure of **PP 7**

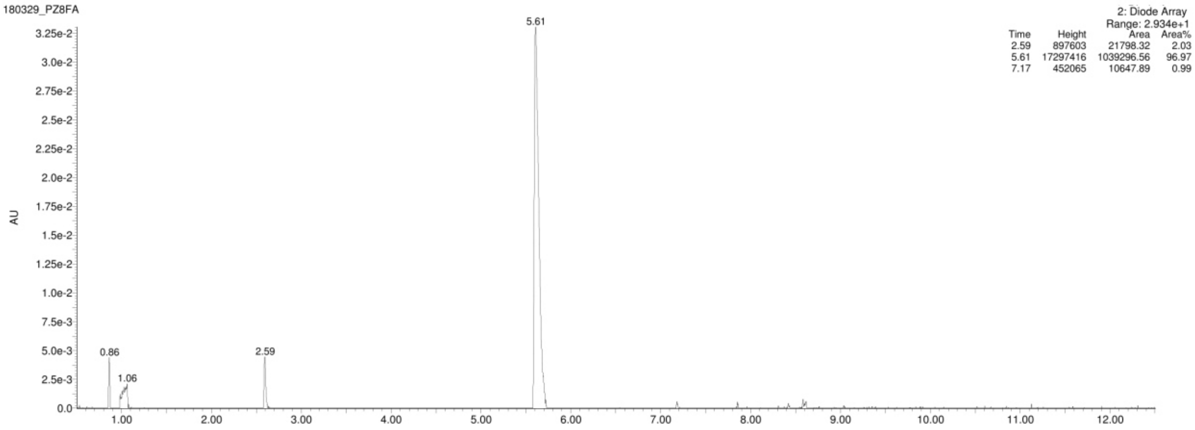

Figure S25. HPLC of **PP 7**

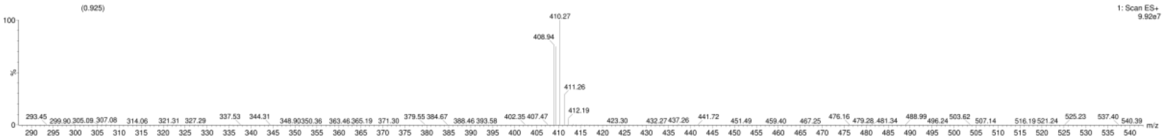

Figure S26. MS of **PP 7**

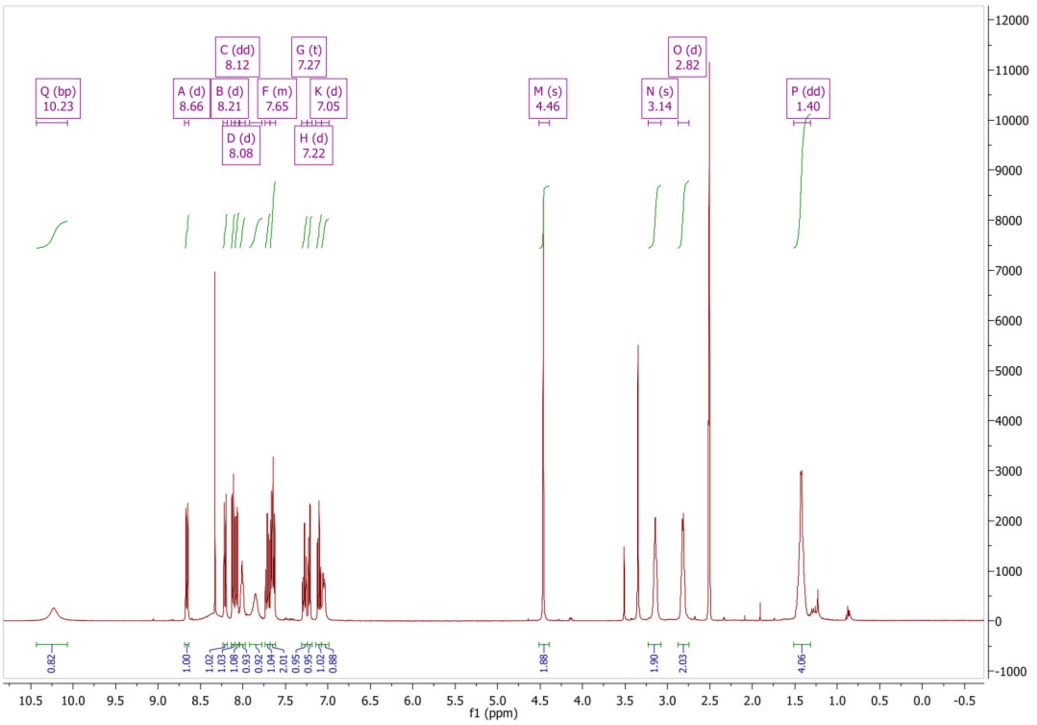

Figure S27. ^{1}H NMR of **PP 7**

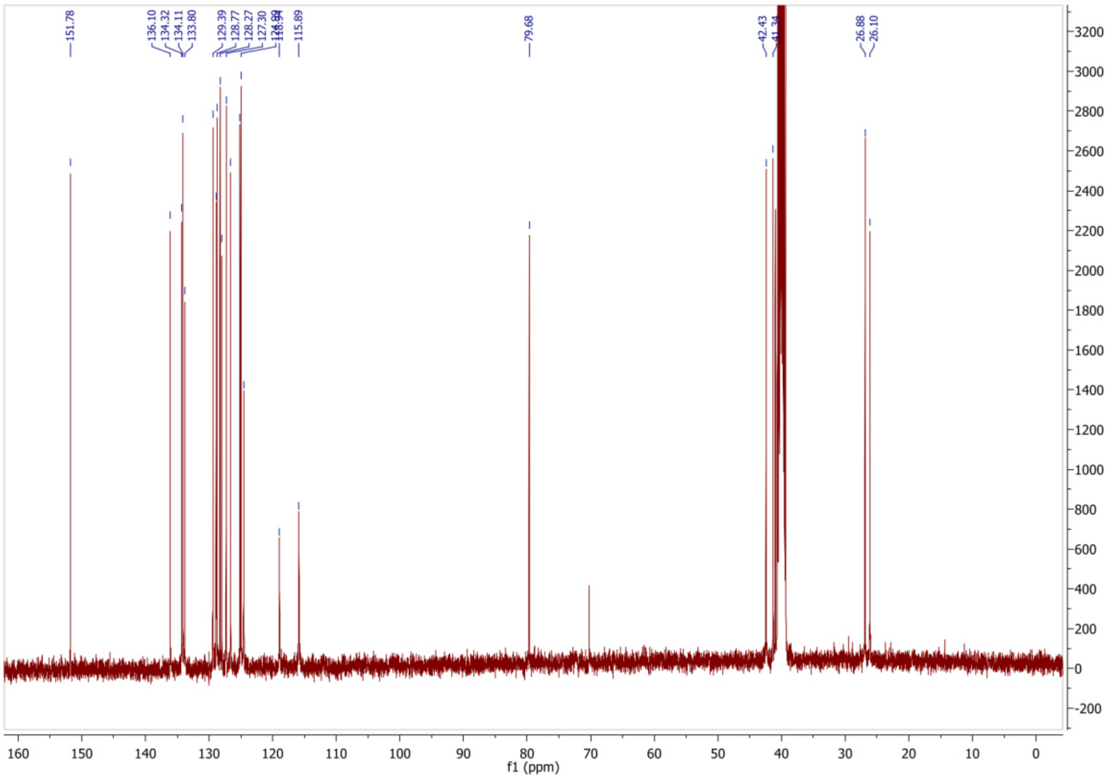

Figure S28. ^{13}C NMR of **PP 7**

N-{6-[(3,4-dihydroquinazolin-2-yl)amino]hexyl}naphthalene-1-sulfonamide PP 8

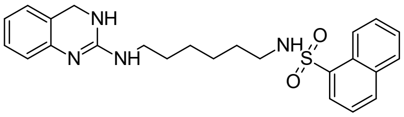

Figure S29. Structure of **PP 8**

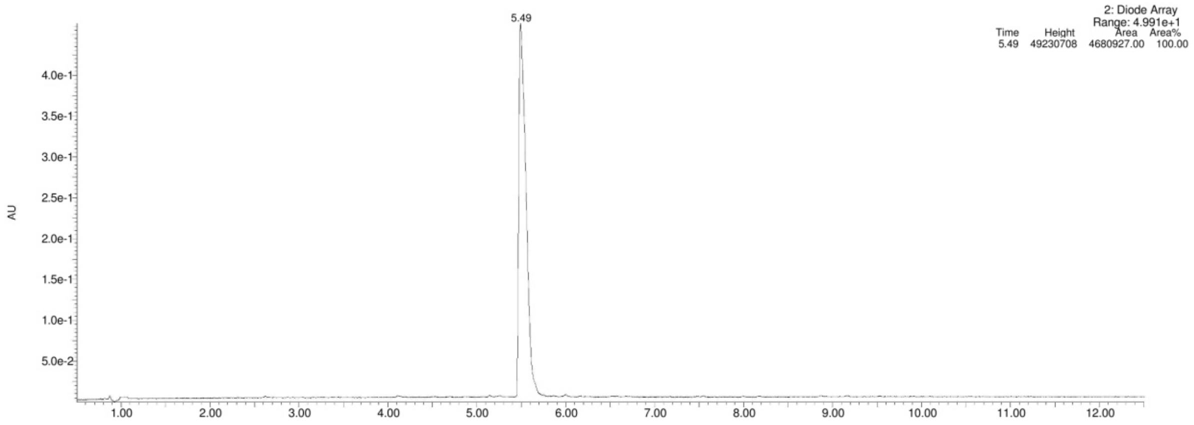

Figure S30. HPLC of **PP 8**

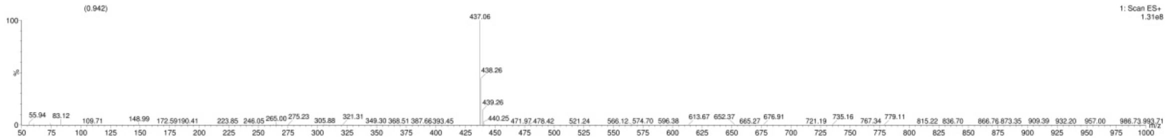

Figure S31. MS of **PP 8**

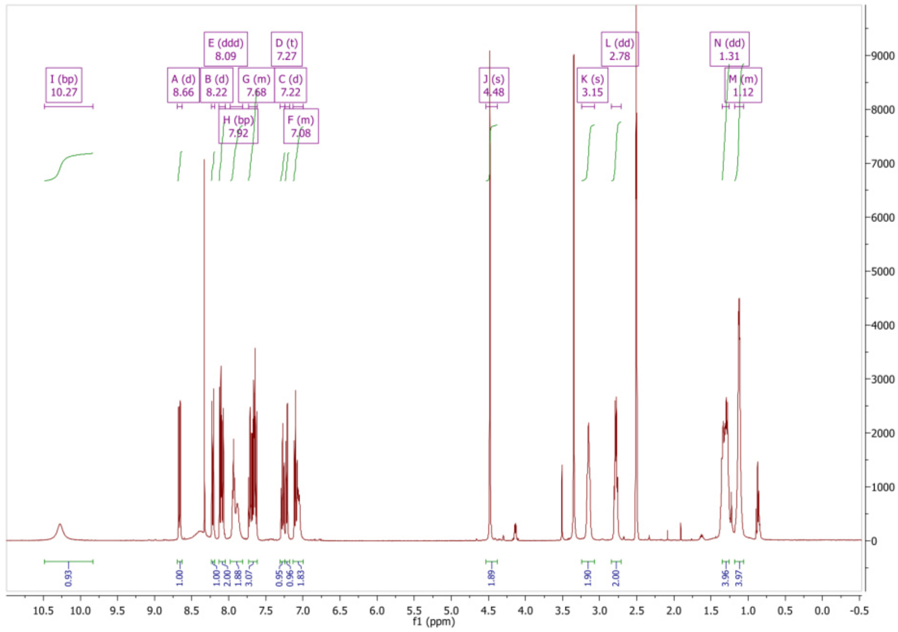

Figure S32. ^{1}H NMR of **PP 8**

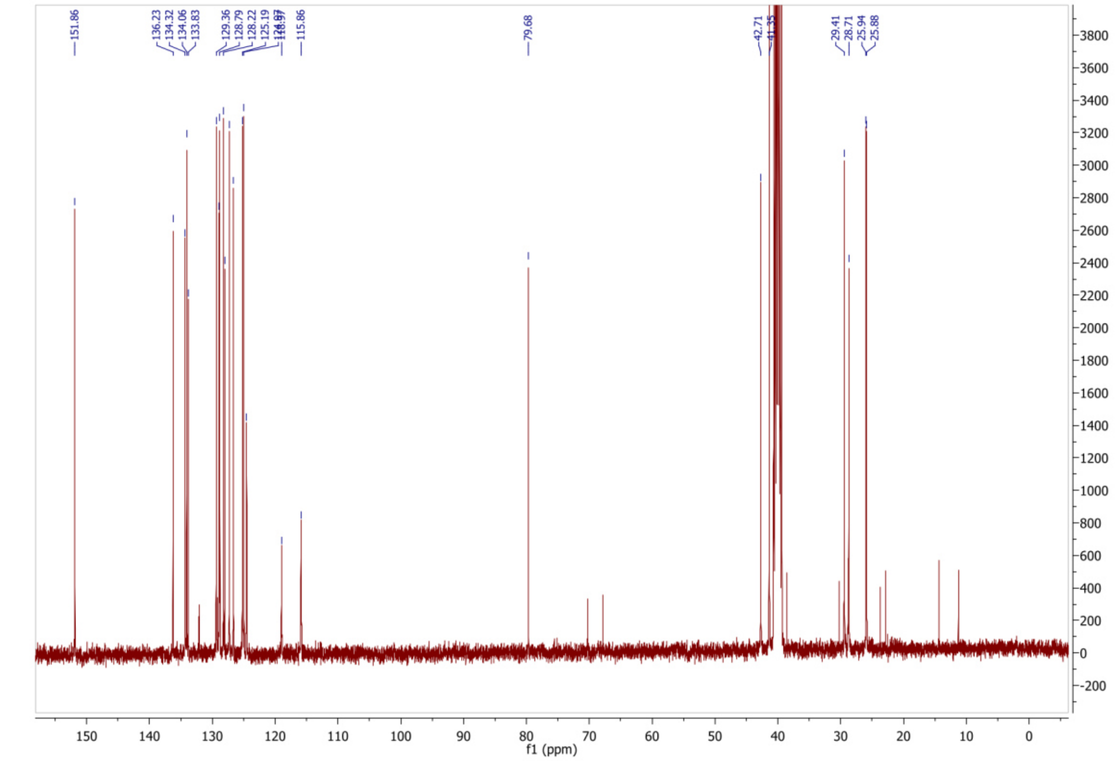

Figure S33. ^{13}C NMR of **PP 8**

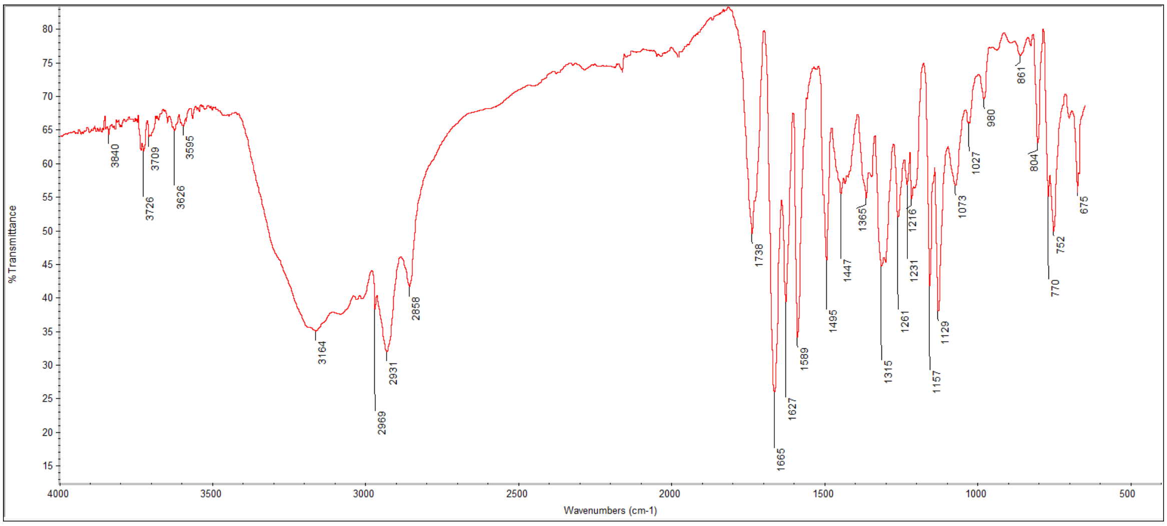

Figure S34. FT IR of **PP 8**

N-{2-[(4-methyl-3,4-dihydroquinazolin-2-yl)amino]ethyl}naphthalene-1-sulfonamide PP 9

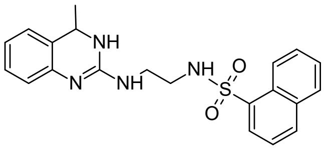

Figure S35. Structure of **PP9**

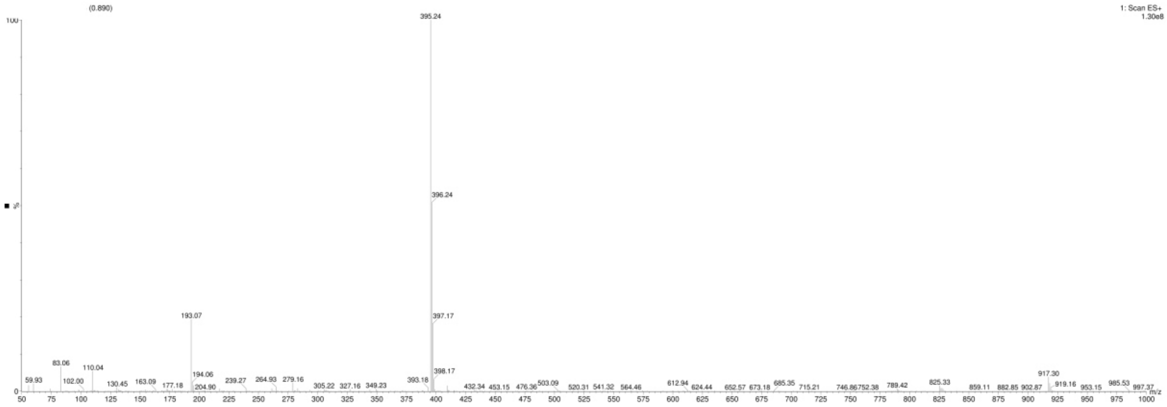

Figure S36. MS of **PP 9**

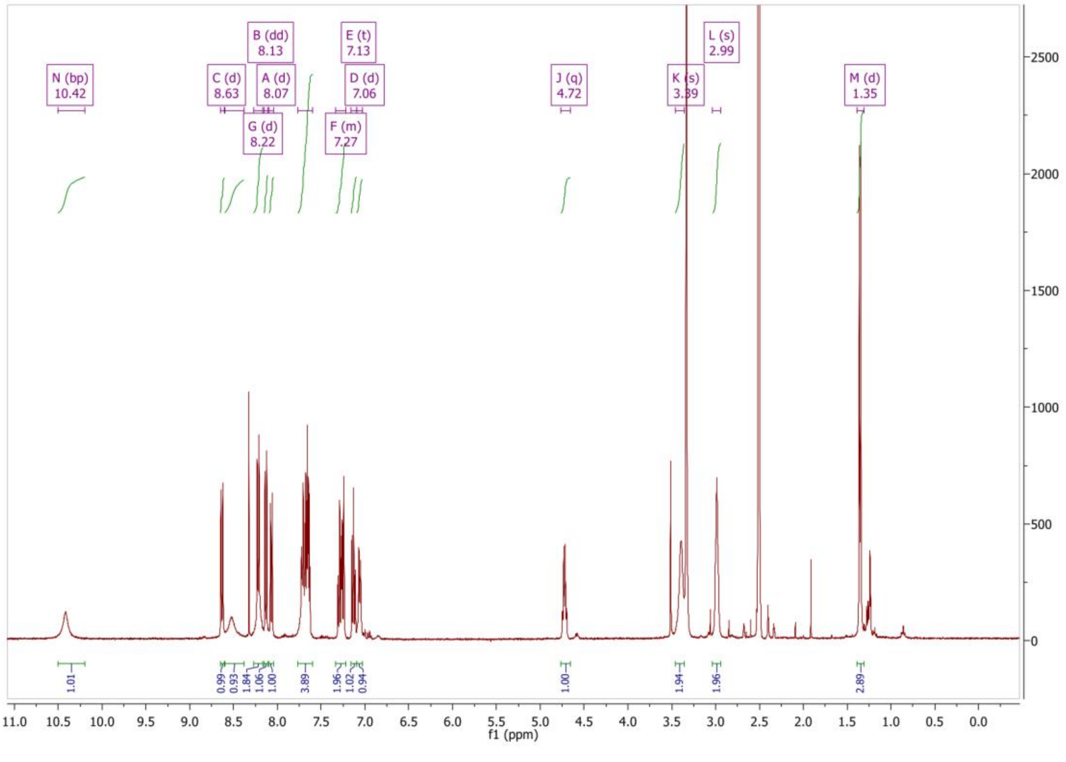

Figure S37. ^{1}H NMR of **PP 9**

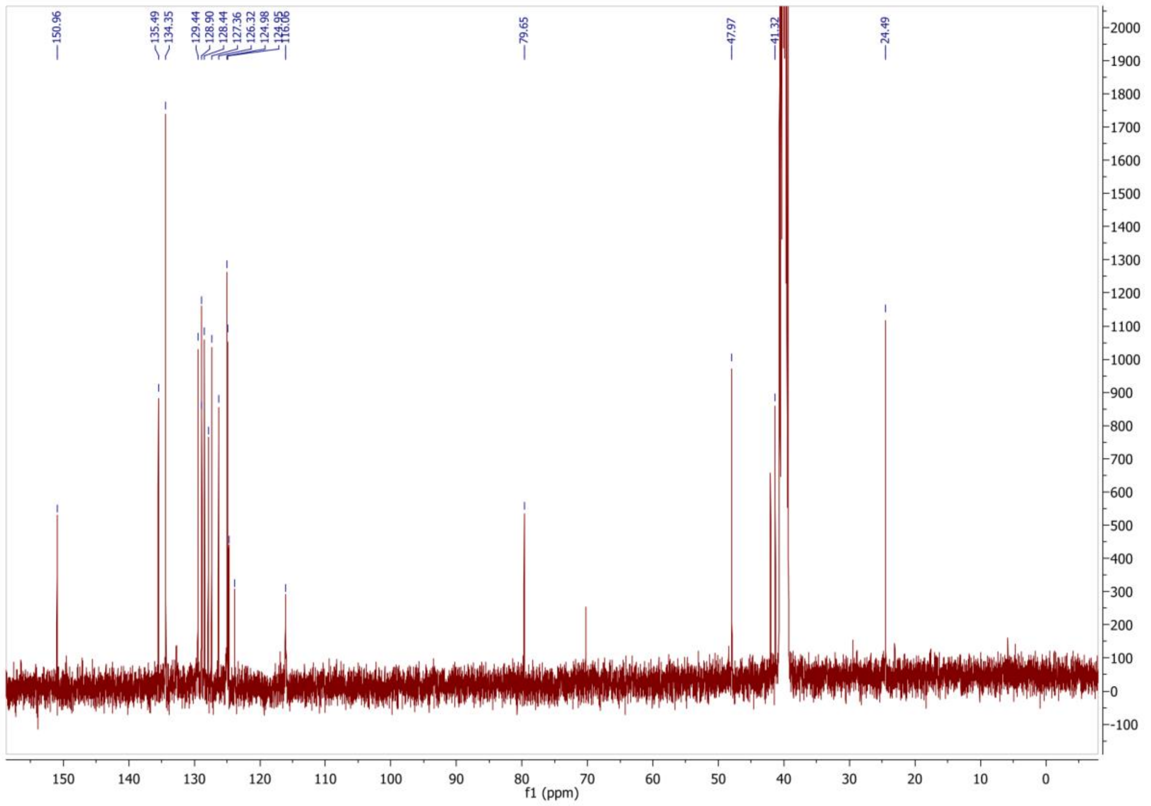

Figure S38. ^{13}C NMR of **PP 9**

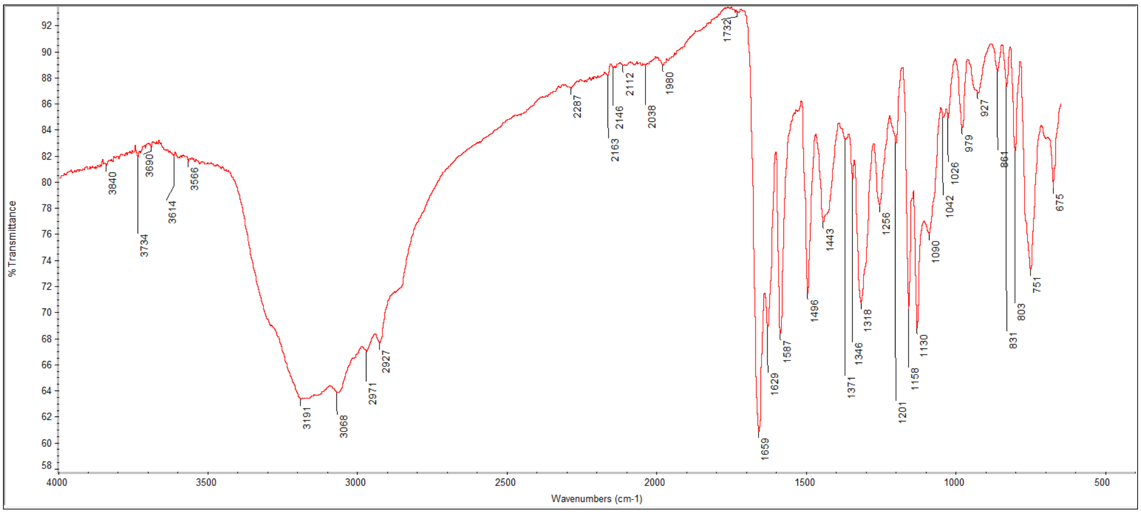

Figure S39. FT IR of **PP 9**

***N*-{6-[(4-methyl-3,4-dihydroquinazolin-2-yl)amino]hexyl}naphthalene-1-sulfonamide PP 10**

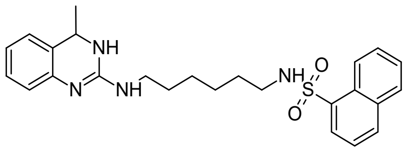

Figure S40. Structure of **PP10**

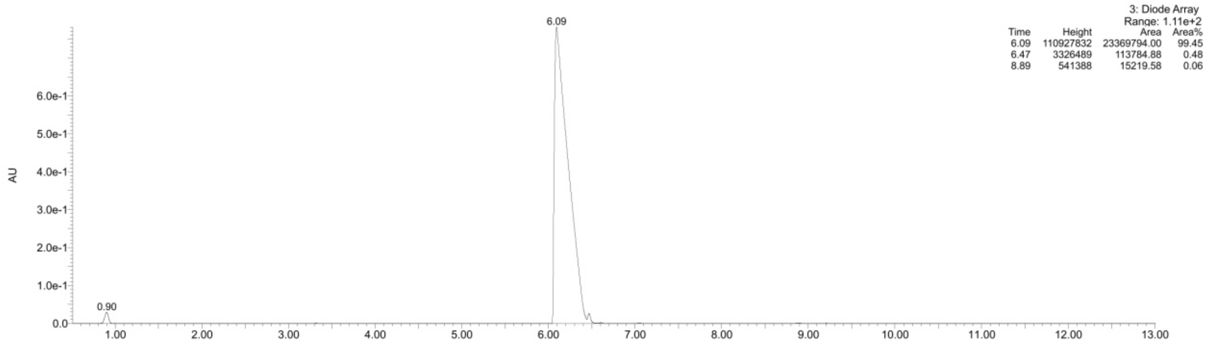

Figure S41. HPLC of **PP 10**

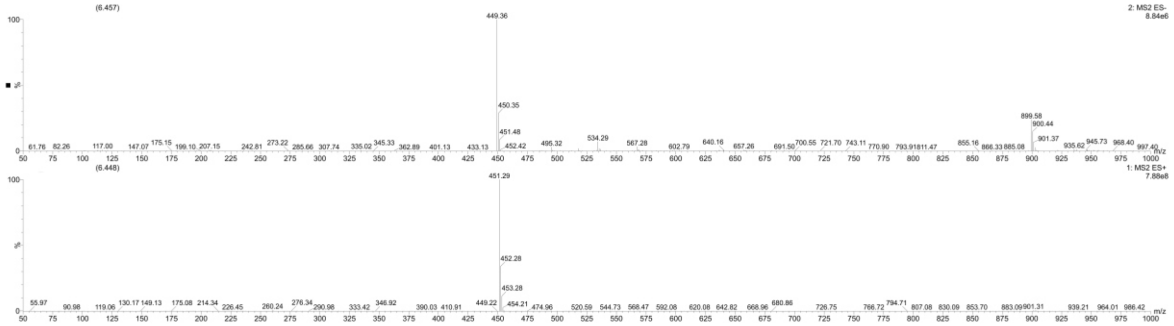

Figure S42. MS of **PP 10**

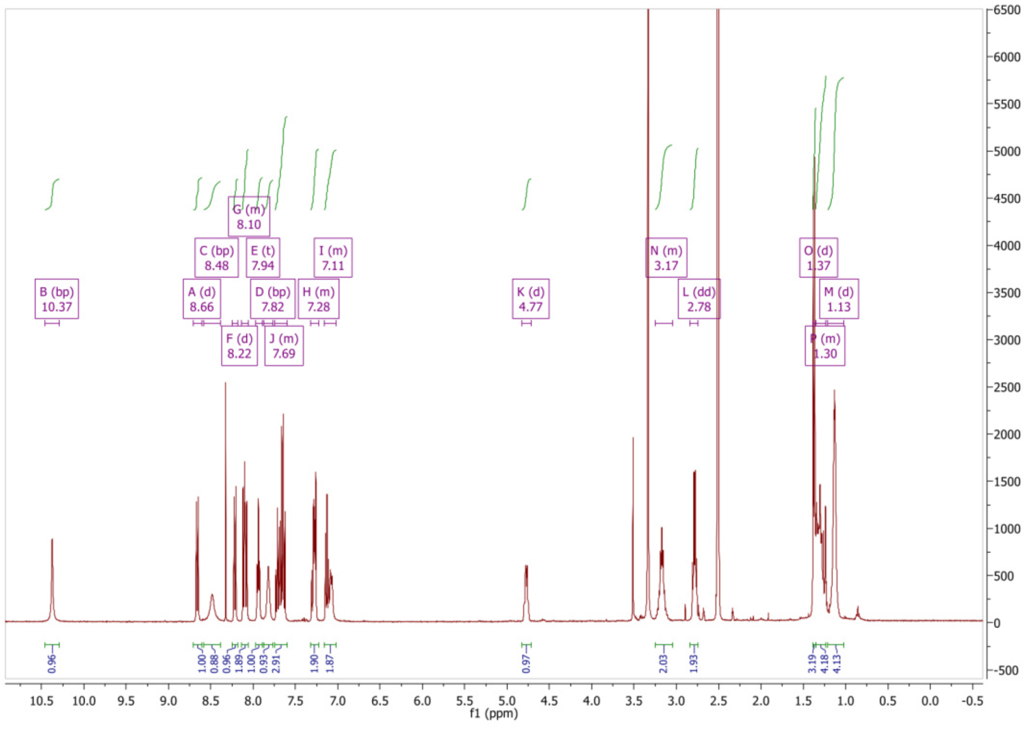

Figure S43. ^{1}H NMR of **PP 10**

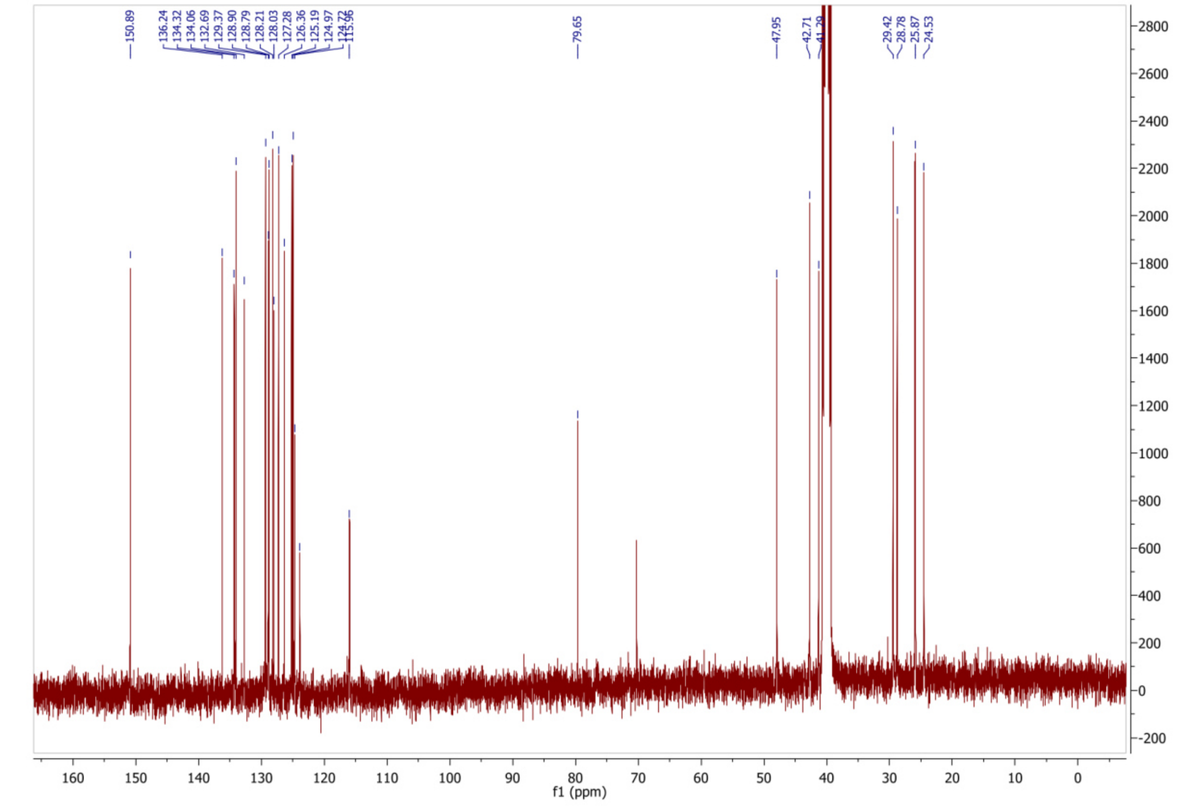

Figure S44. ^{13}C NMR of **PP 10**

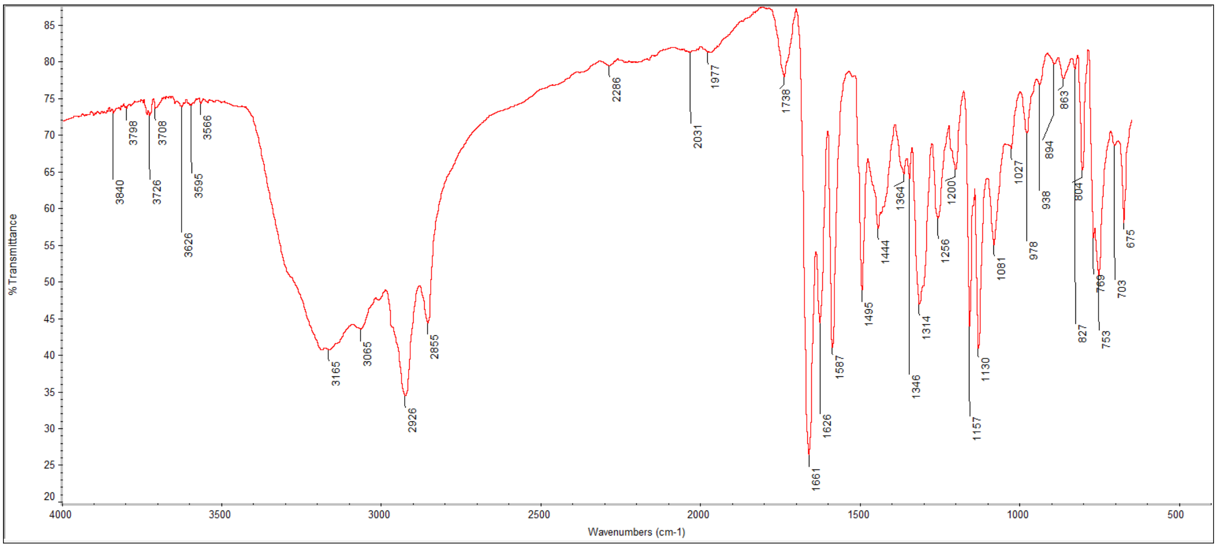

Figure S45. FT IR of **PP 10**

_N_-{6-[(5-fluoro-4-methyl-3,4-dihydroquinazolin-2-yl)amino]hexyl}naphthalene-1-sulfonamide PP 11

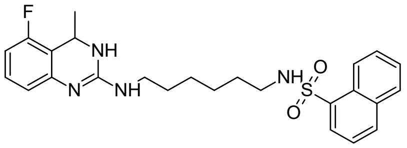

Figure S46. Structure of **PP 11**

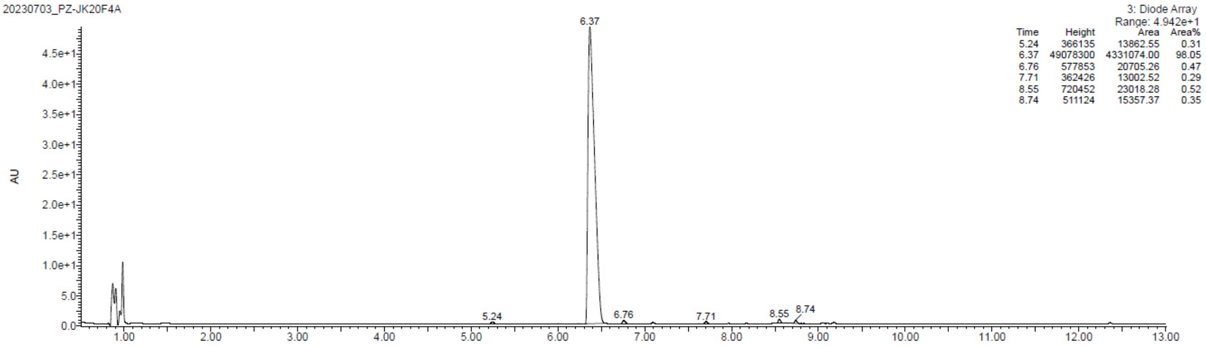

Figure S47. HPLC of **PP 11**

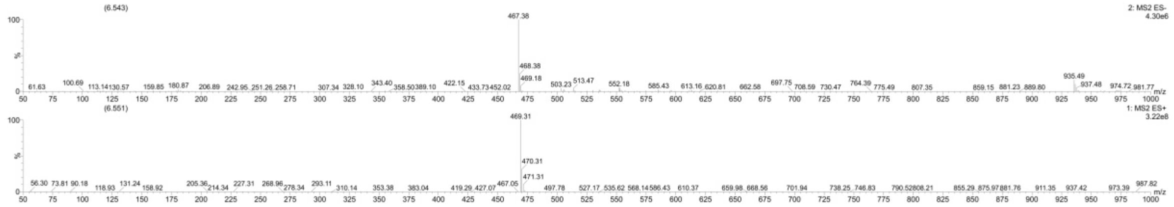

Figure S48. MS of **PP 11**

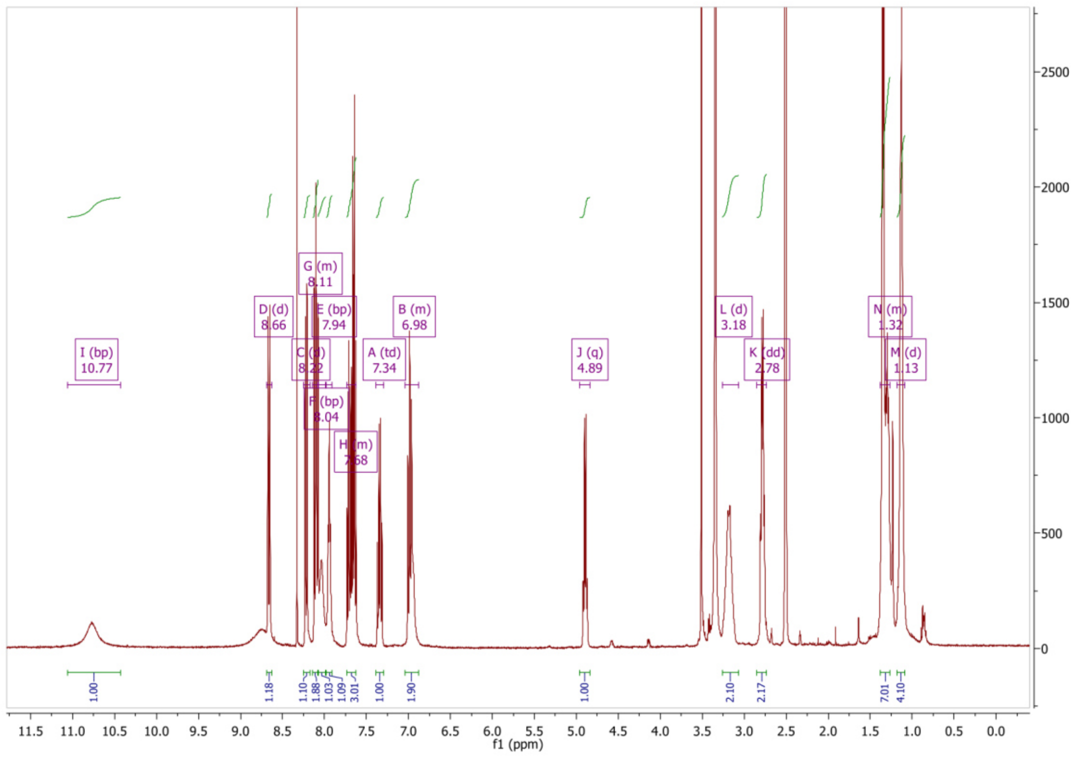

Figure S49. ^{1}H NMR of **PP 11**

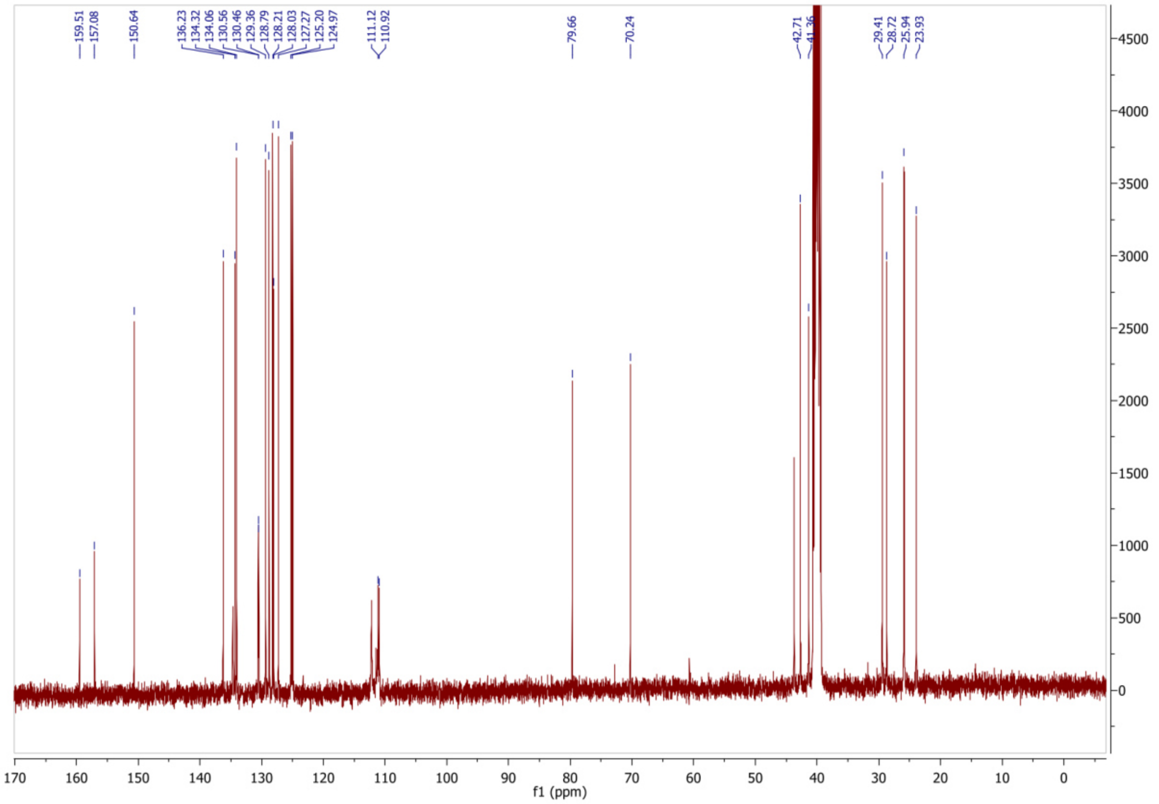

Figure S50. ^{13}C NMR of **PP 11**

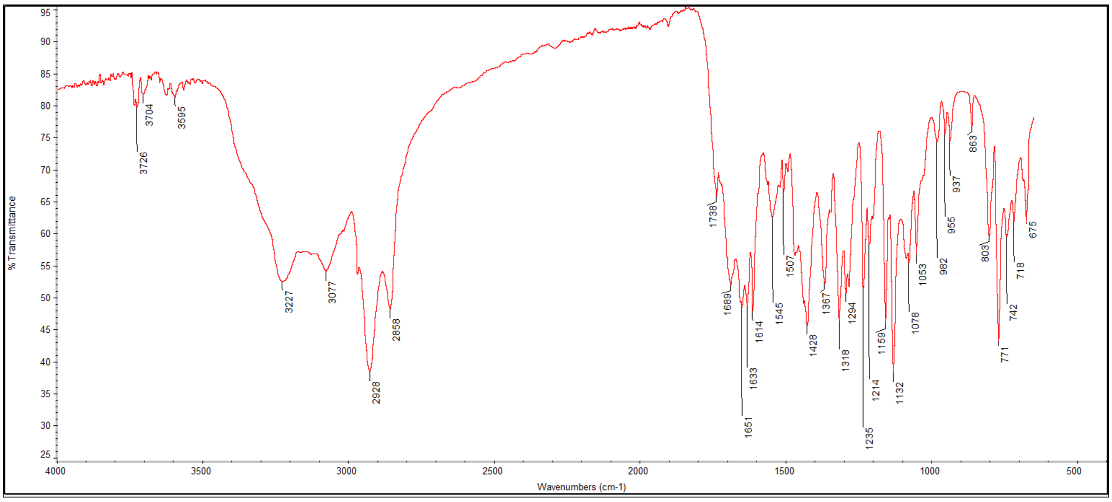

Figure S51. FT IR of **PP 11**

***N*-{6-[(6,8-dichloro-4-methyl-3,4-dihydroquinazolin-2-yl)amino]hexyl}naphthalene-1-sulfonamide PP 12**

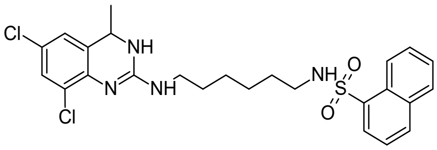

Figure S52. Structure of **PP 12**

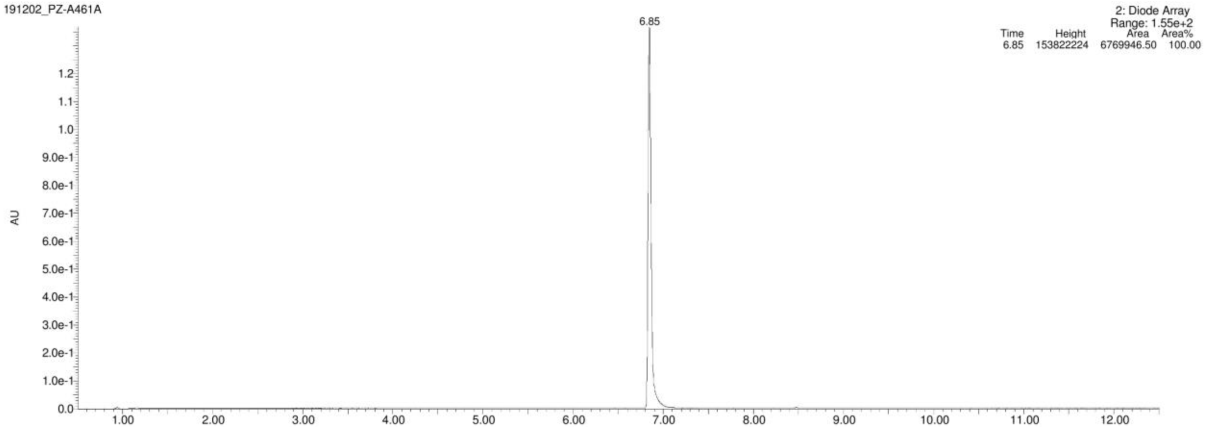

Figure S53. HPLC of **PP 12**

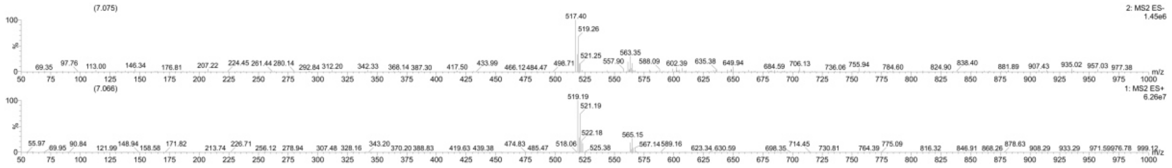

Figure S54. MS of **PP**

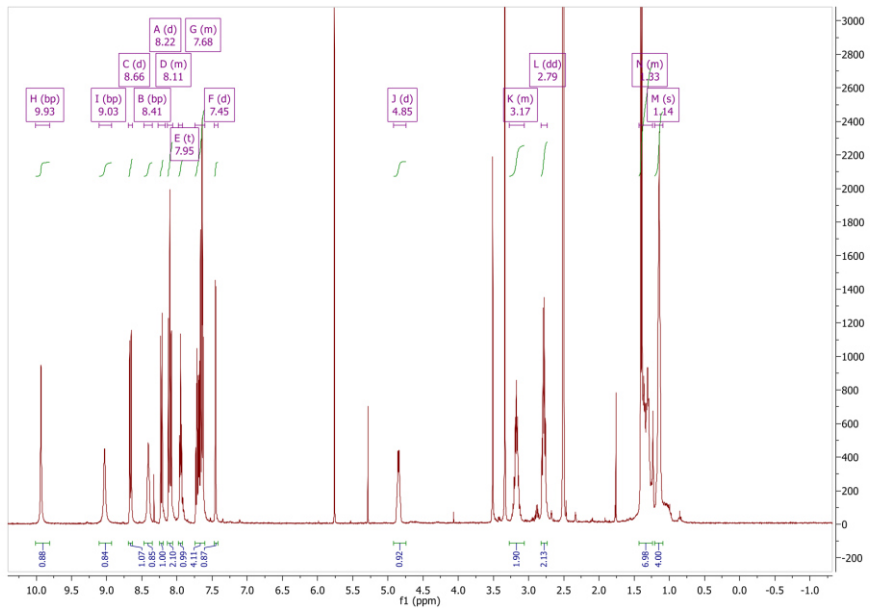

Figure S55. ^{1}H NMR of **PP 12**

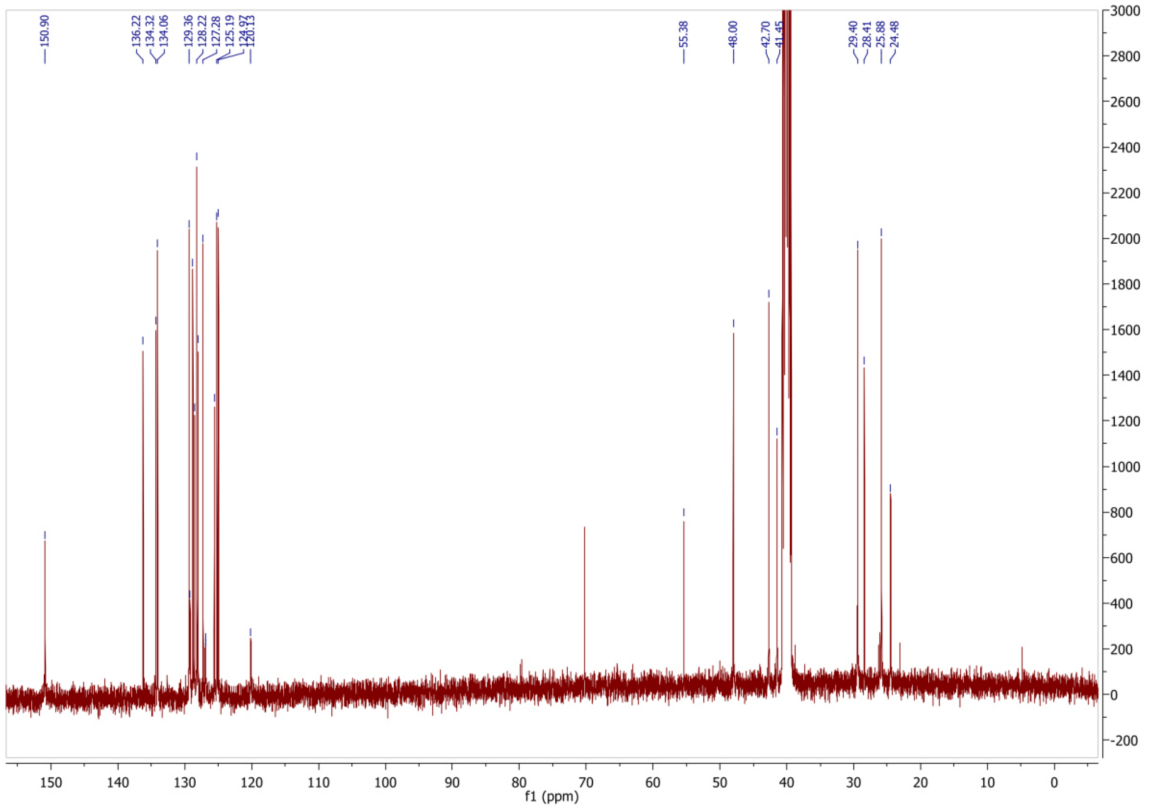

Figure S56. ^{13}C NMR of **PP 12**

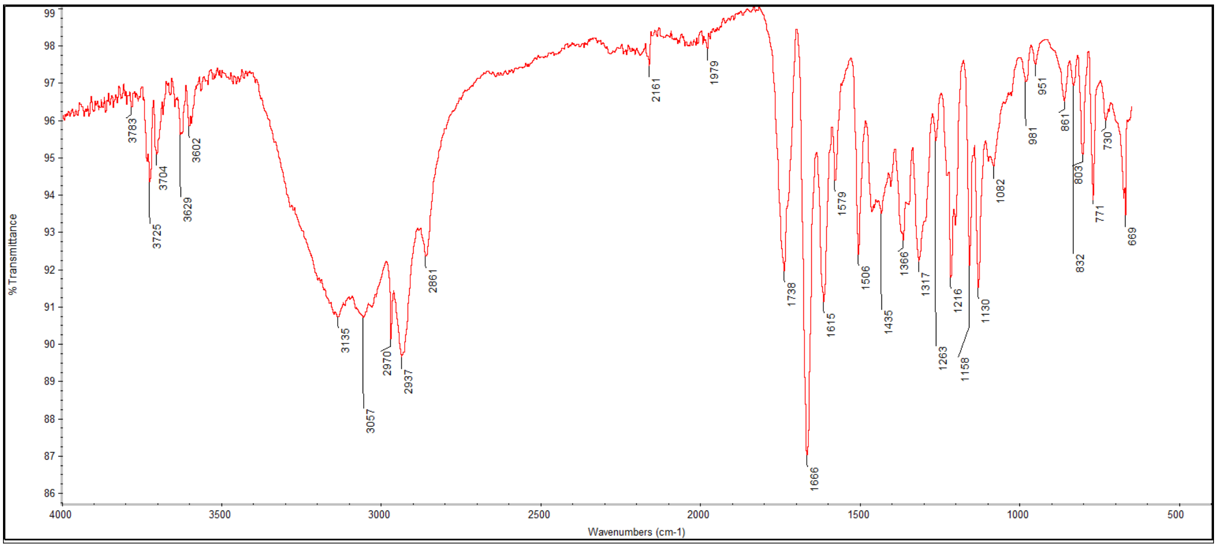

Figure S57. FT IR of **PP 12**

2-chloro-*N*-{6-[(4-methyl-3,4-dihydroquinazolin-2-yl)amino]hexyl}naphthalene-1-sulfonamide PP 13

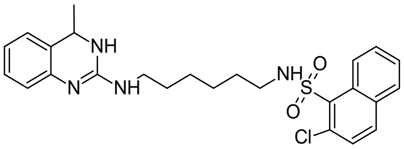

Figure S58. Structure of **PP 13**

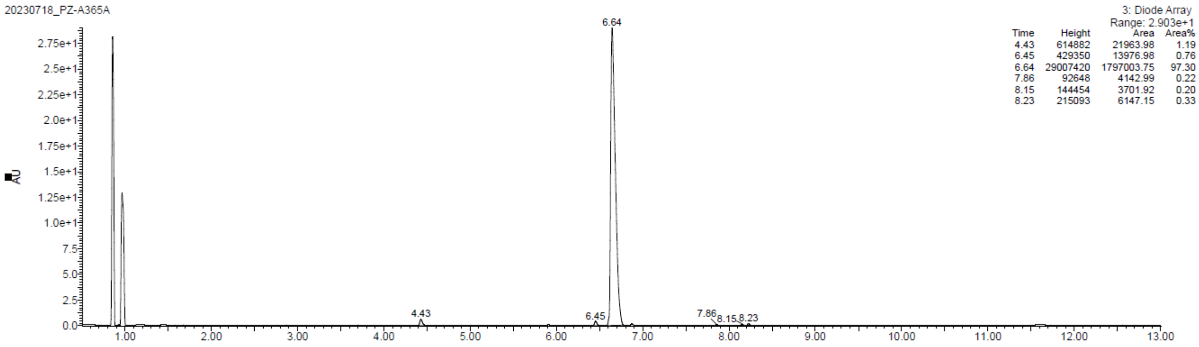

Figure S59. HPLC of **PP 13**

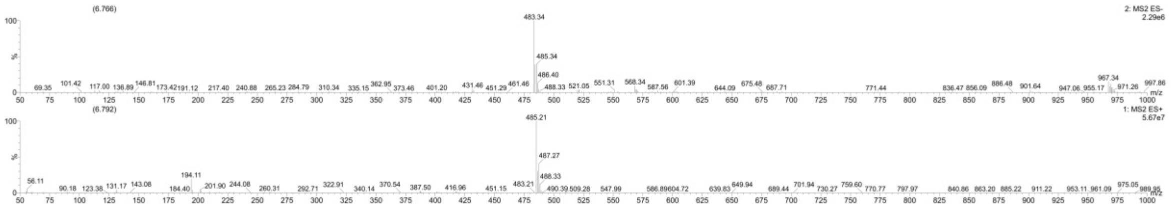

Figure S60. MS of **PP 13**

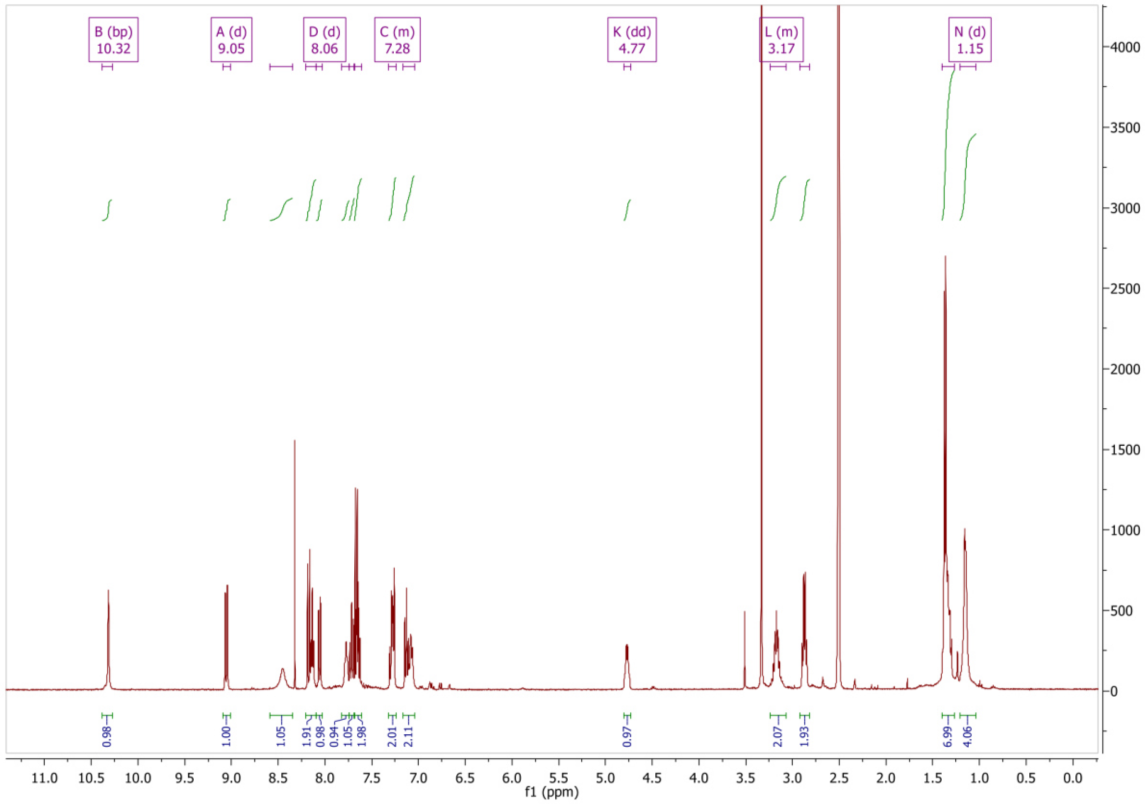

Figure S61. ^{1}H NMR of **PP 13**

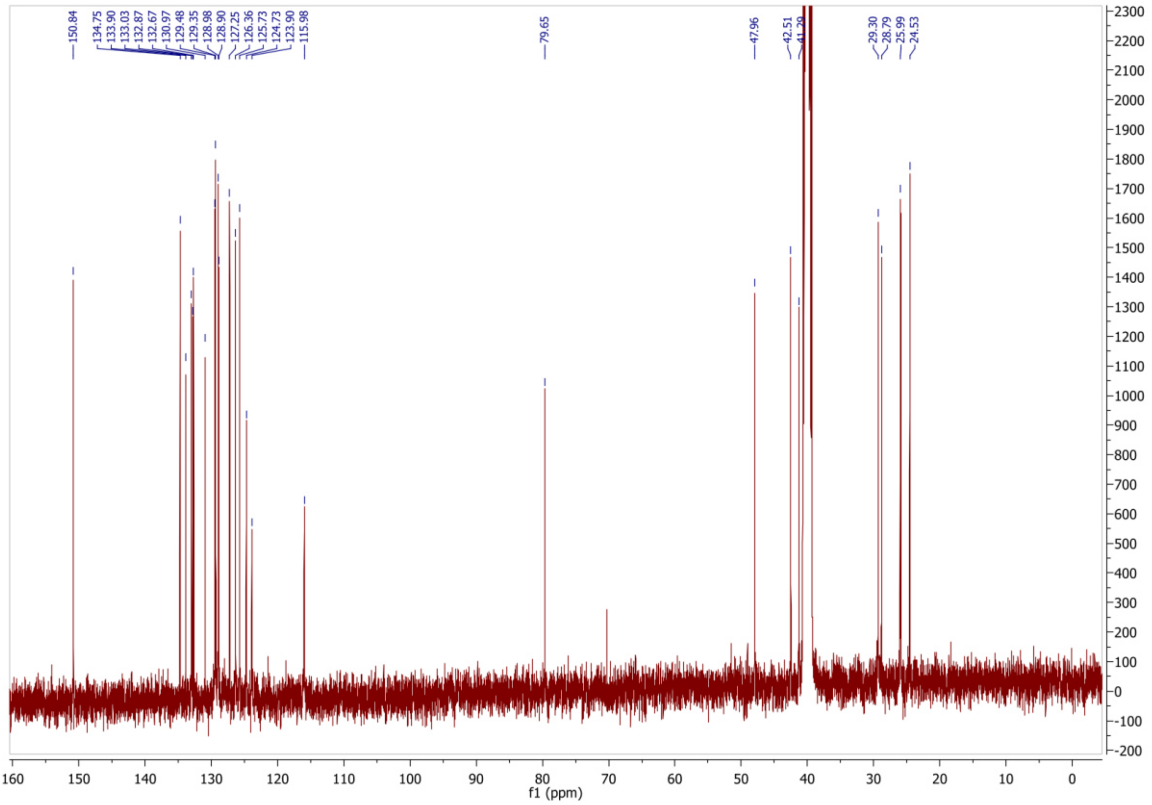

Figure S62. ^{13}C NMR of **PP 13**

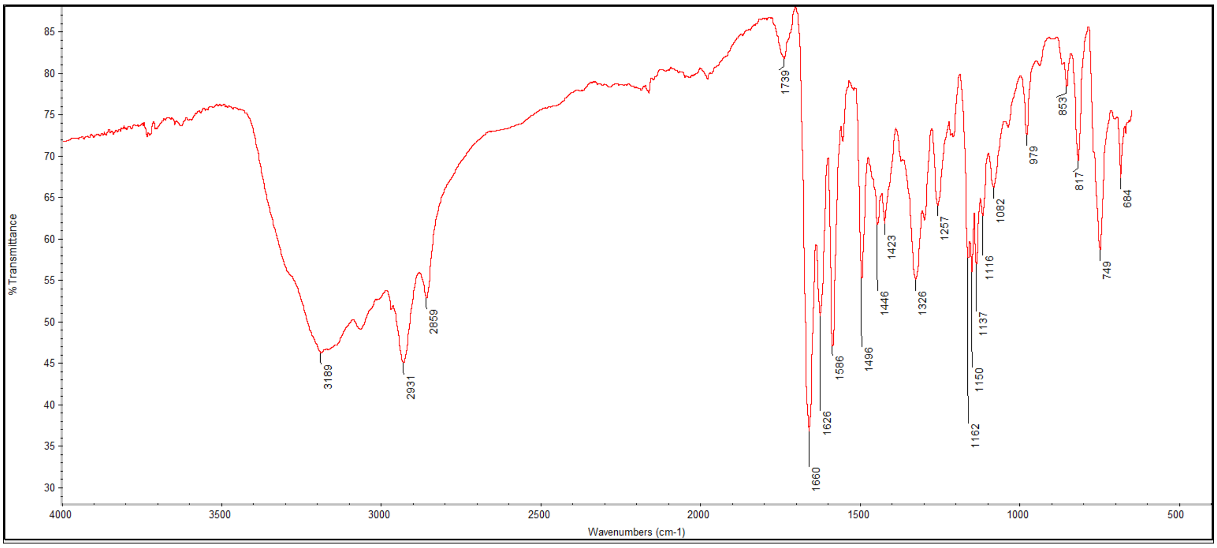

Figure S63. FT IR of **PP 13**

N-{2-[(quinazolin-2-yl)amino]ethyl}naphthalene-1-sulfonamide PP 14

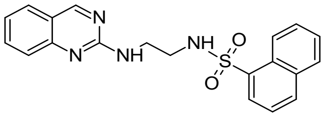

Figure S64. Structure of **PP 14**

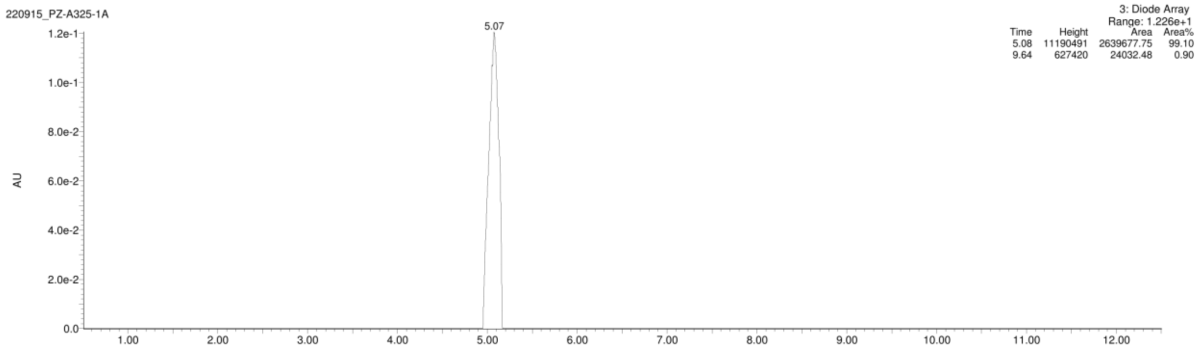

Figure S65. HPLC of **PP 14**

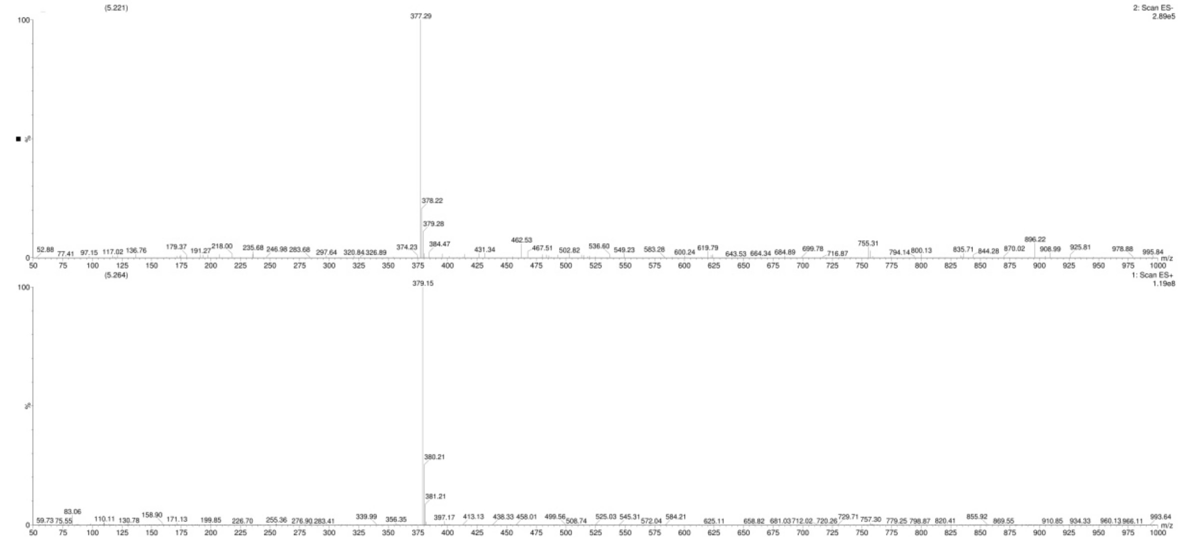

Figure S66. MS of **PP 14**

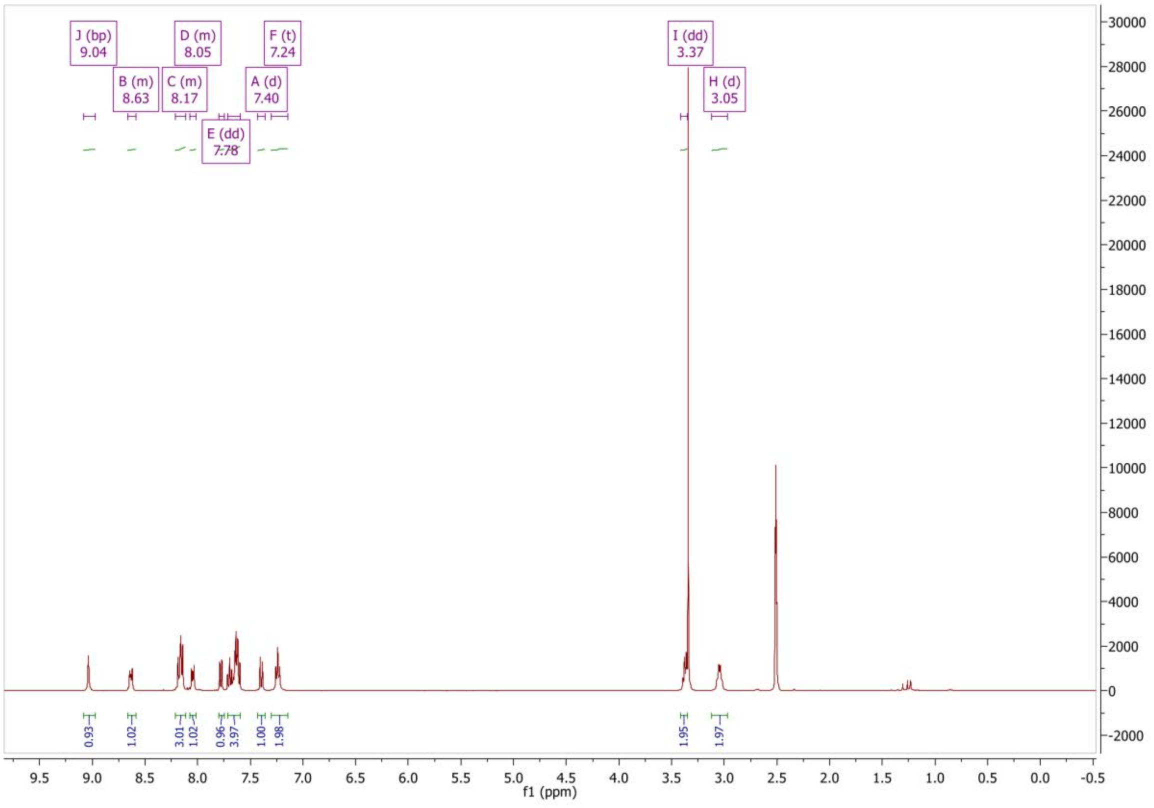

Figure S67. ^{1}H NMR of **PP 14**

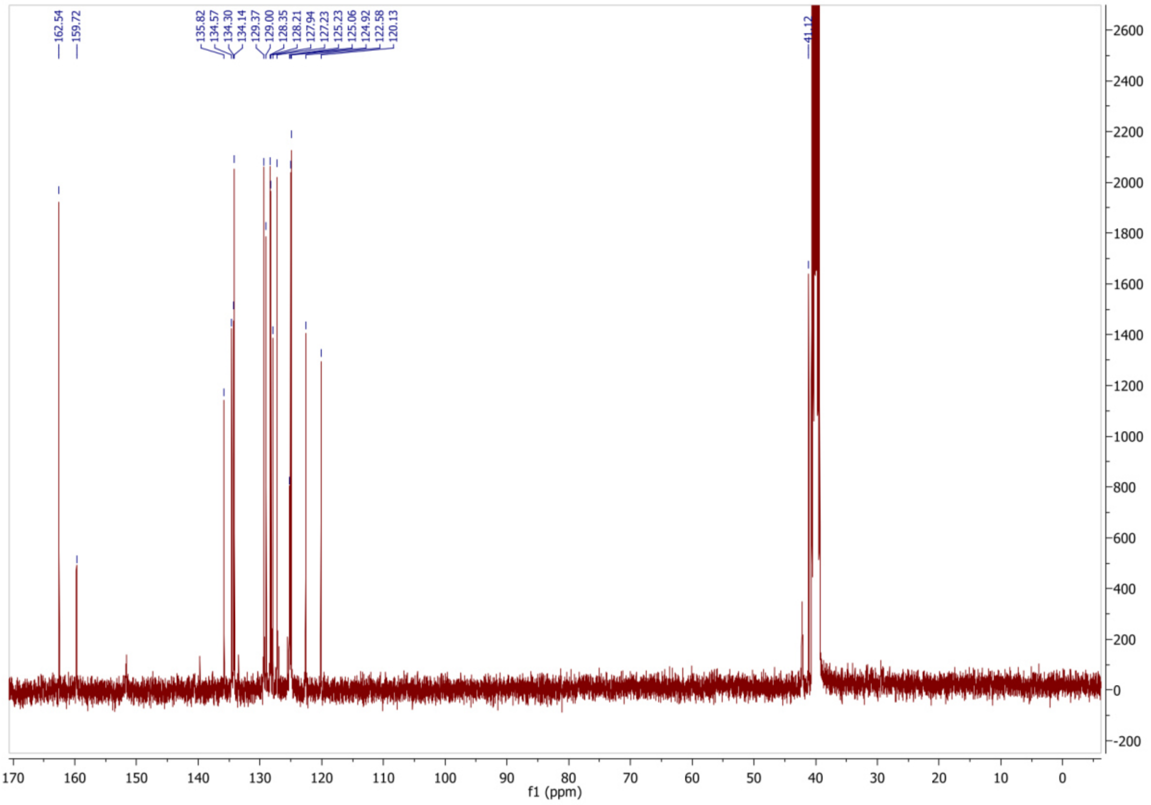

Figure S68. ^{13}C NMR of **PP 14**

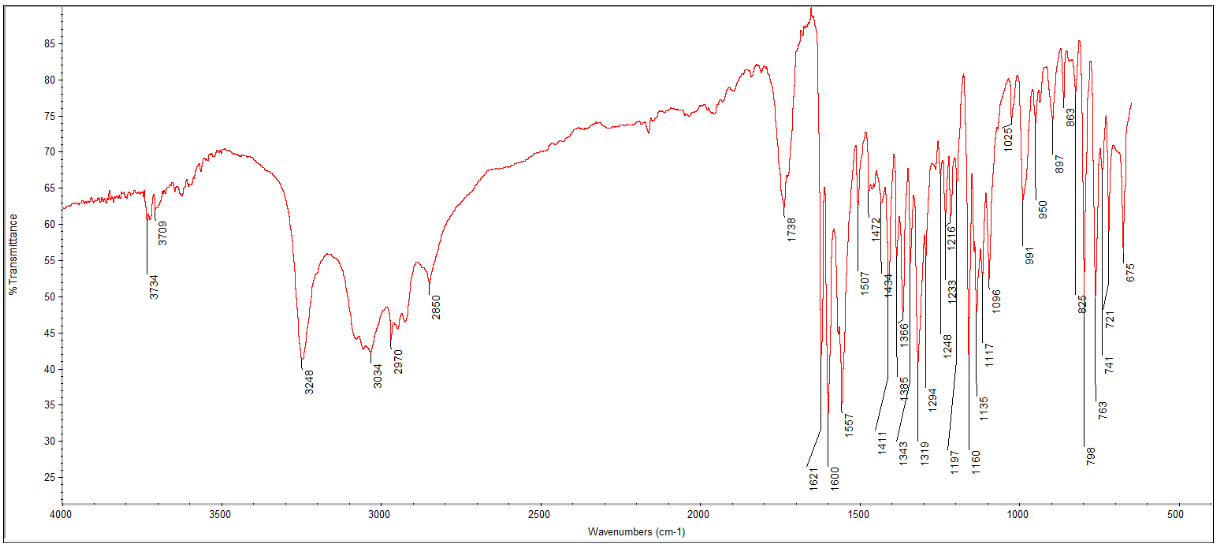

Figure S69. FT IR of **PP 14**

***N*-{6-[(quinazolin-2-yl)amino]hexyl}naphthalene-1-sulfonamide PP 15**

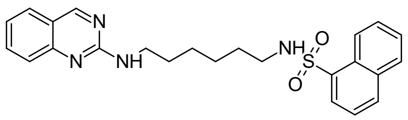

Figure S70. Structure of **PP 15**

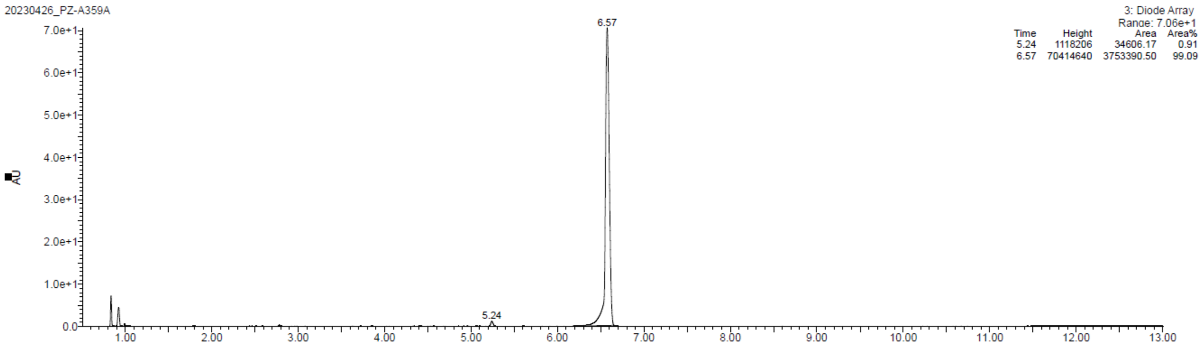

Figure S71. HPLC of **PP 15**

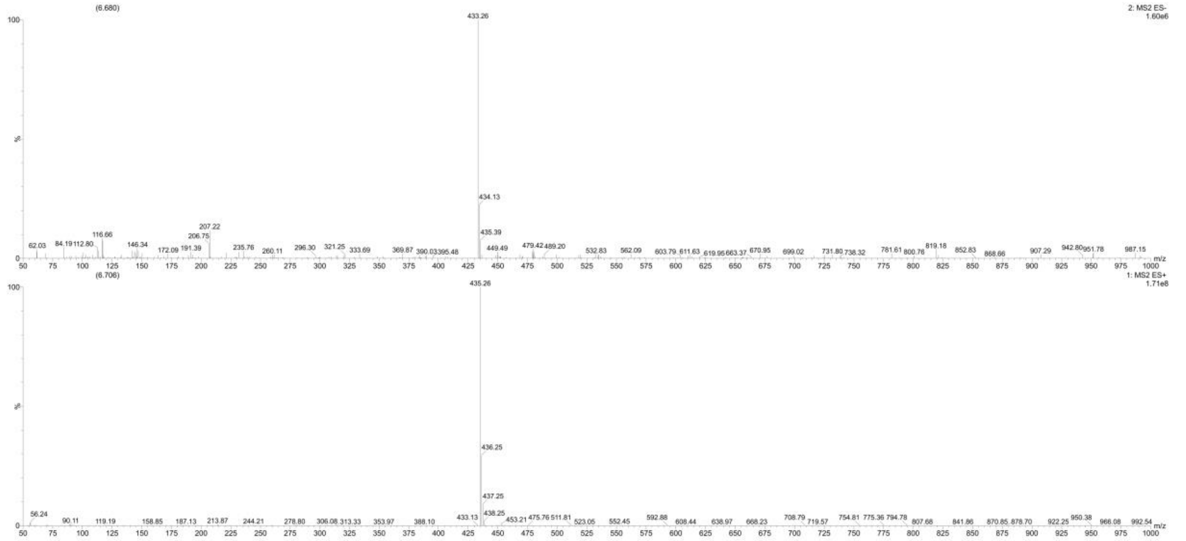

Figure S72. MS of **PP 15**

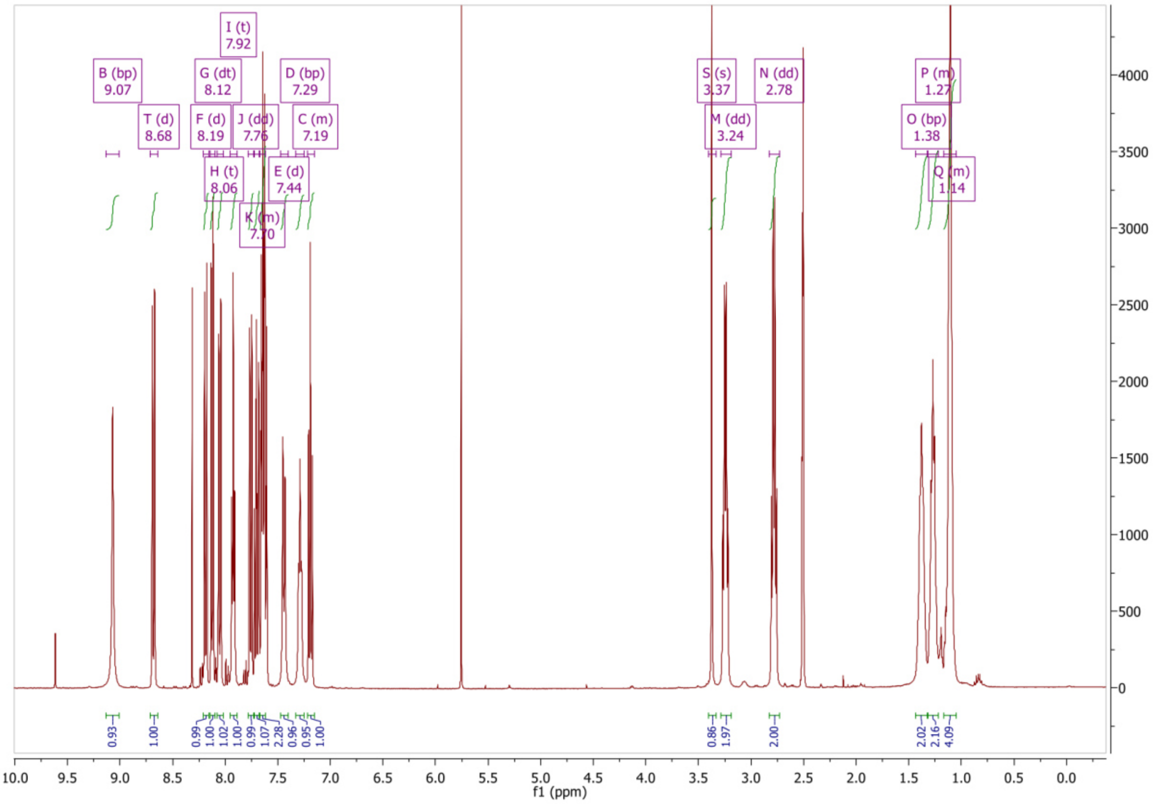

Figure S73. ^{1}H NMR of **PP 15**

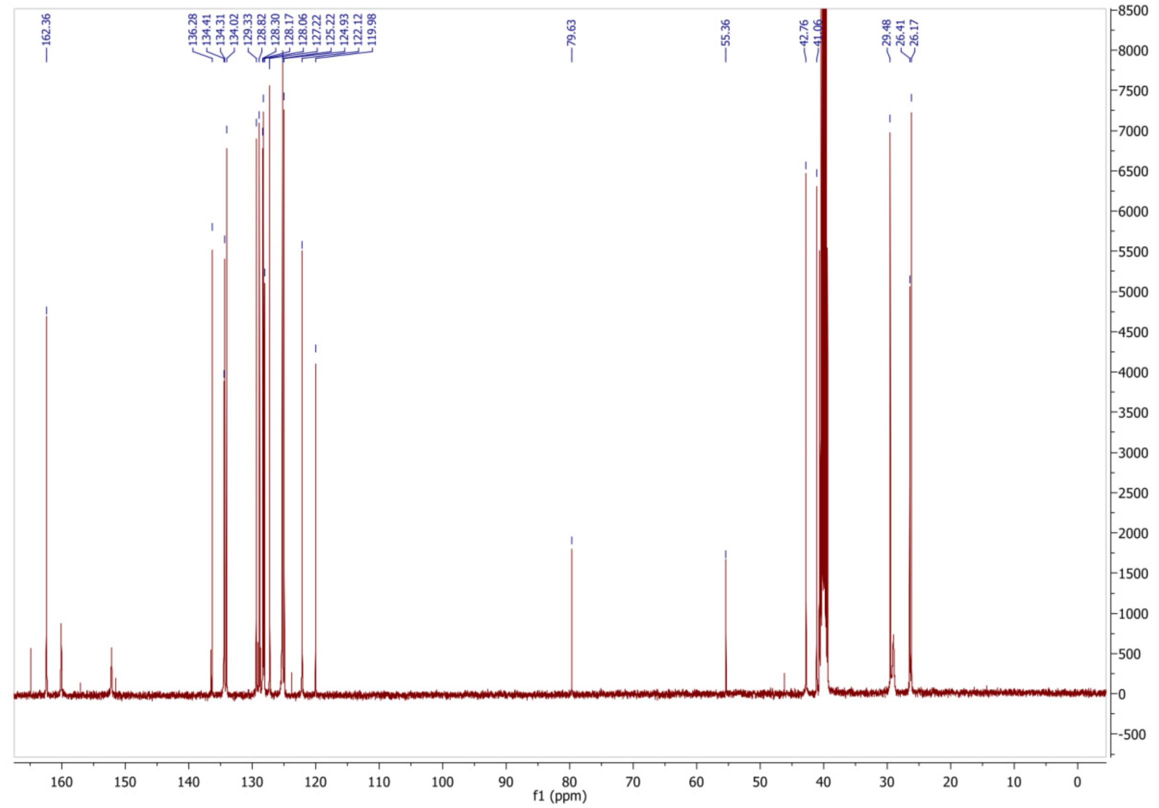

Figure S74. ^{13}C NMR of **PP 15**

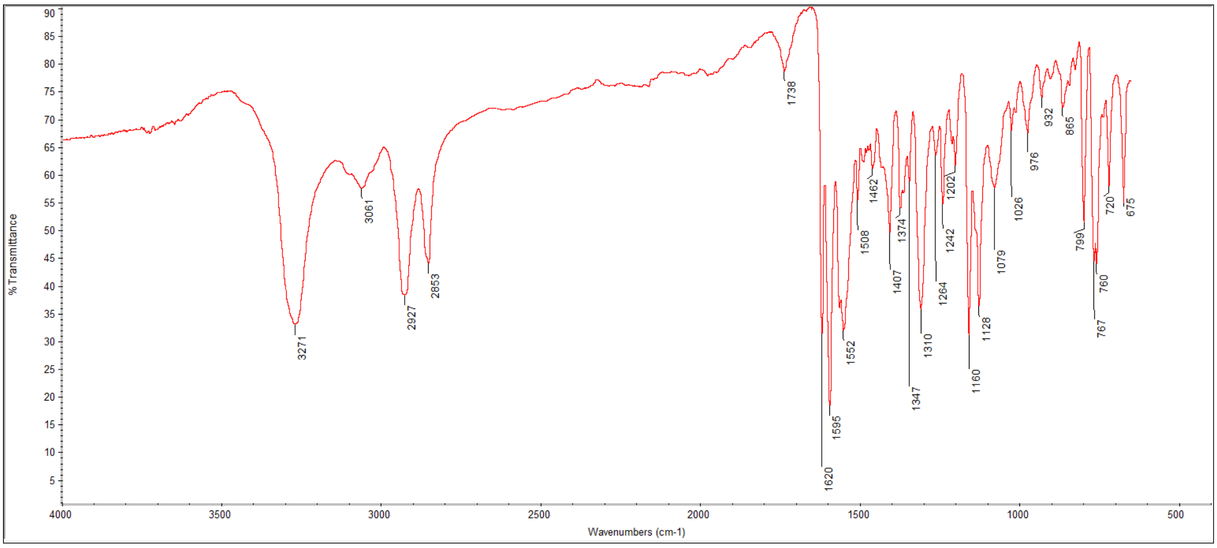

Figure S75. FT IR of **PP 15**

N-{6-[(pyrimidin-2-yl)amino]hexyl}naphthalene-1-sulfonamide PP 16

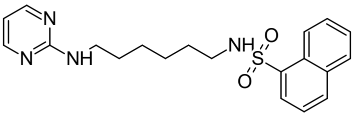

Figure S76. Structure of **PP 16**

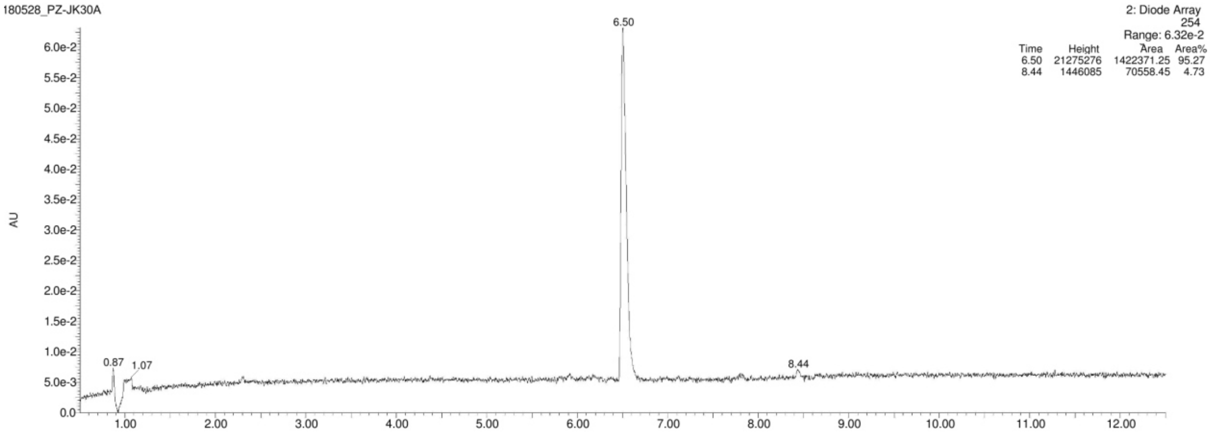

Figure S77. HPLC of **PP 16**

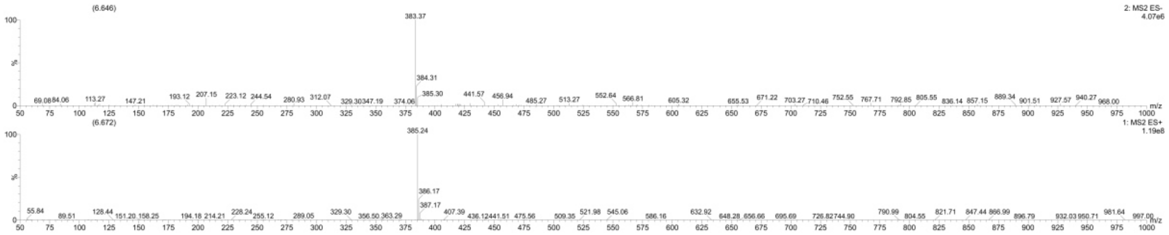

Figure S78. MS of **PP 16**

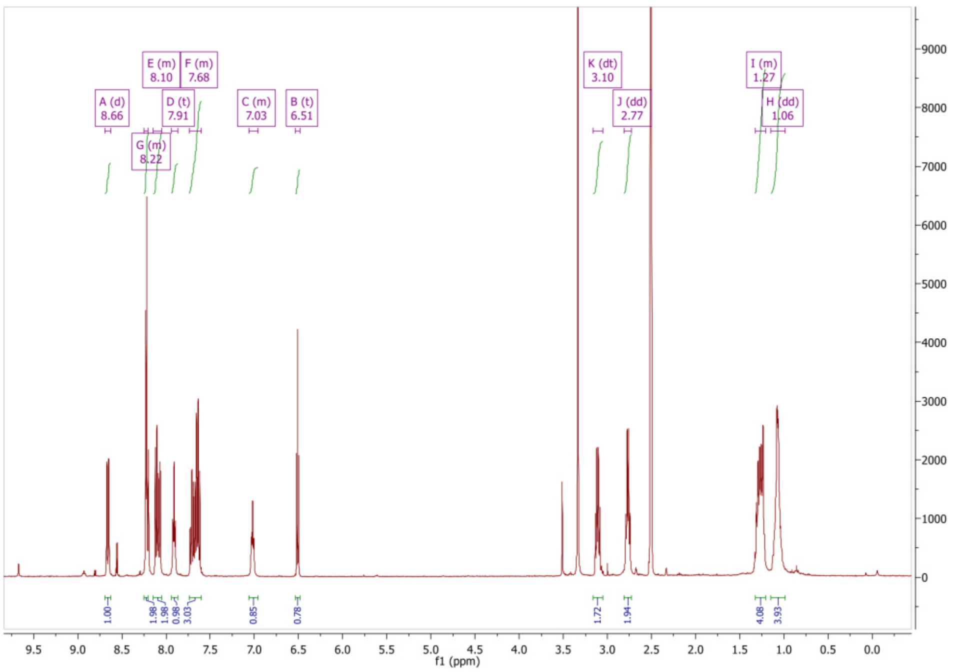

Figure S79. ^{1}H NMR of **PP 16**

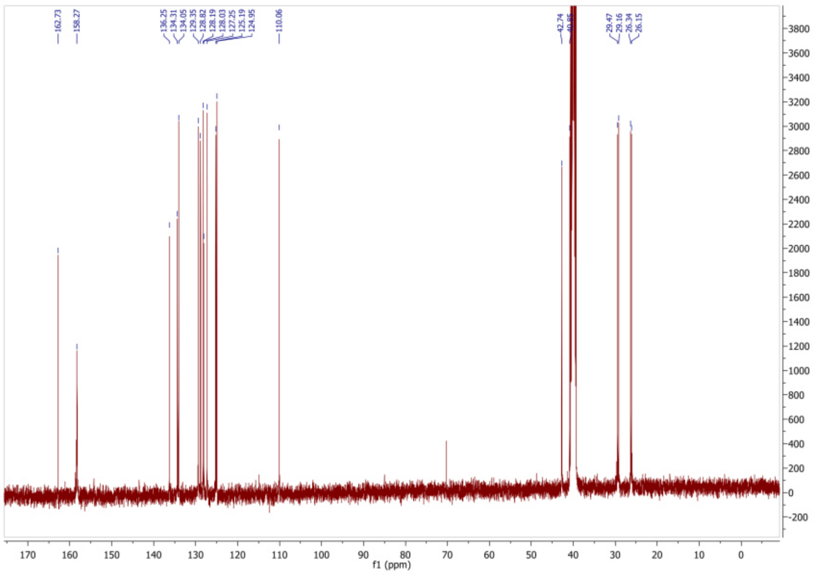

Figure S80. ^{13}C NMR of **PP 16**

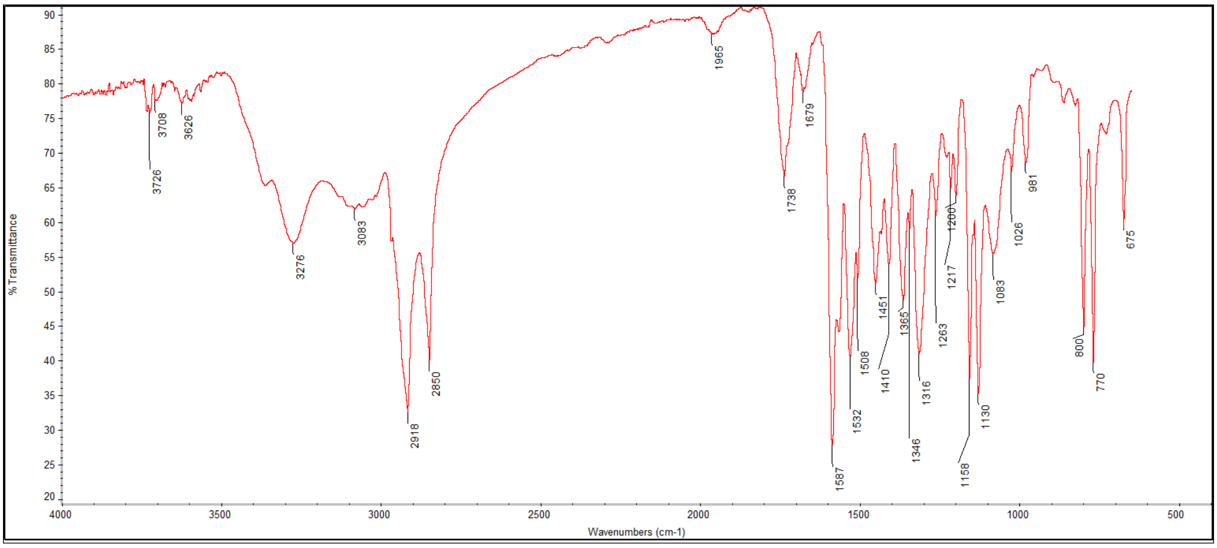

Figure S81. FT IR of **PP 16**

N-{2-[4-(1,2-benzothiazol-3-yl)piperazin-1-yl]ethyl}-3,4-dihydroquinazolin-2-amine PP 21

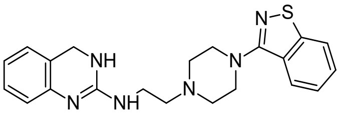

Figure S82. Structure of **PP 21**

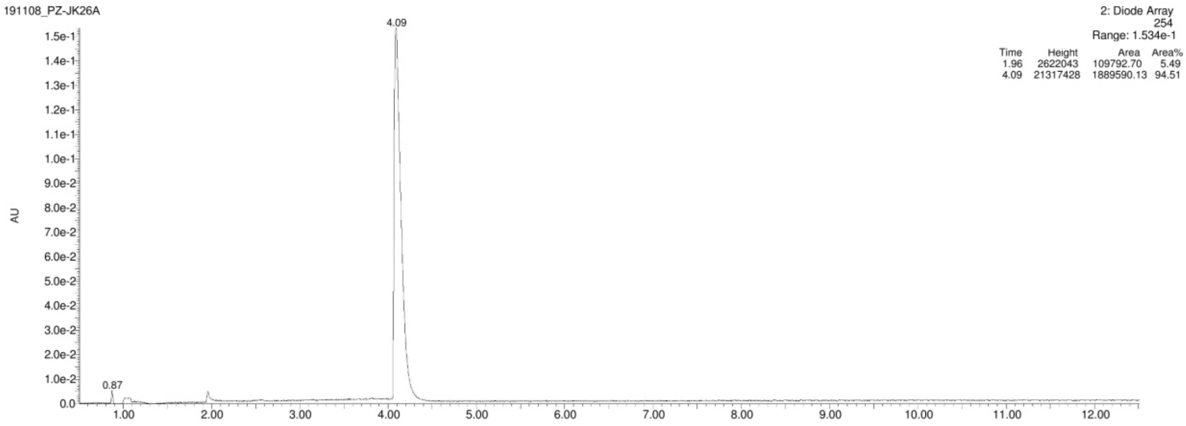

Figure S83. HPLC of **PP 21**

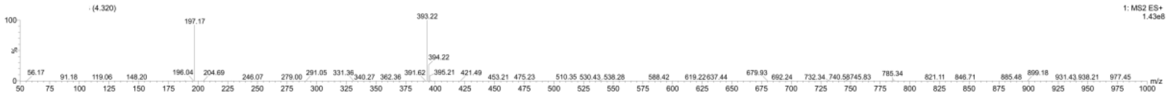

Figure S84. MS of **PP 21**

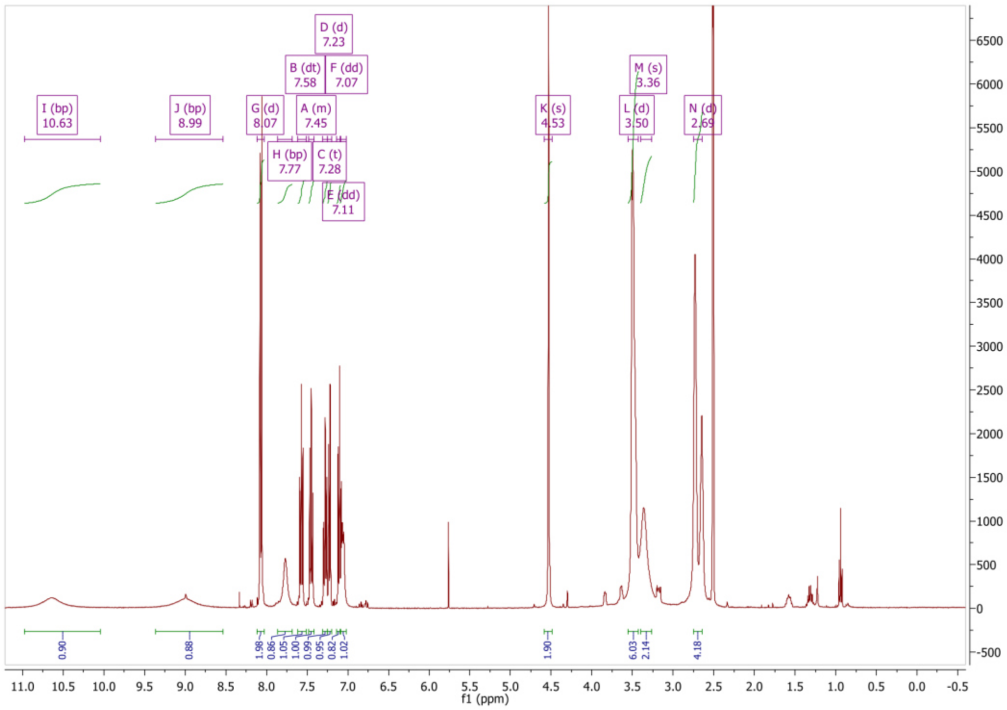

Figure S85. ^{1}H NMR of **PP 21**

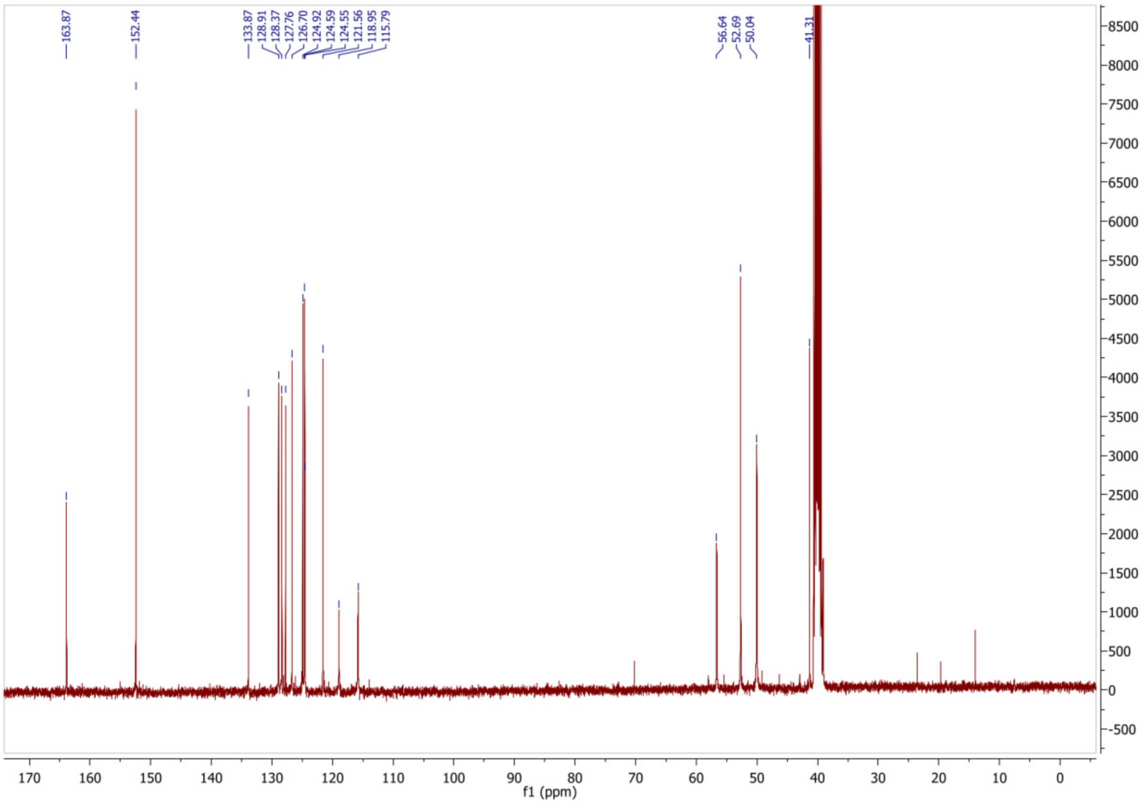

Figure S86. ^{13}C NMR of **PP 21**

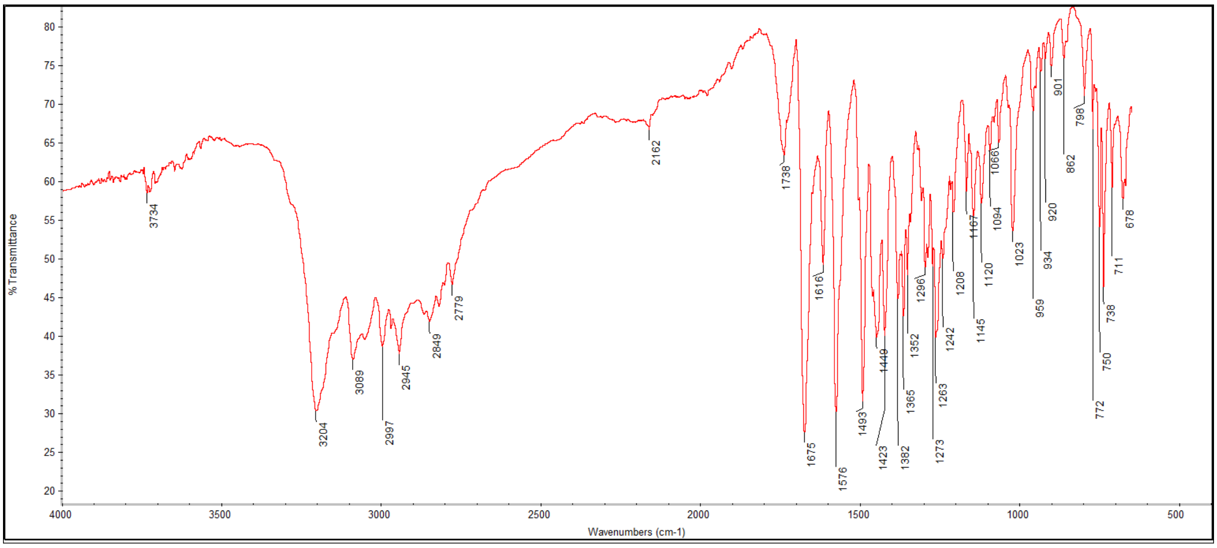

Figure S87. FT IR of **PP 21**

N-{2-[4-(1-benzothiophen-4-yl)piperazin-1-yl]ethyl}-3,4-dihydroquinazolin-2-amine PP 22

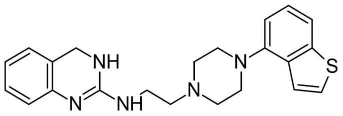

Figure S88. Structure of **PP 22**

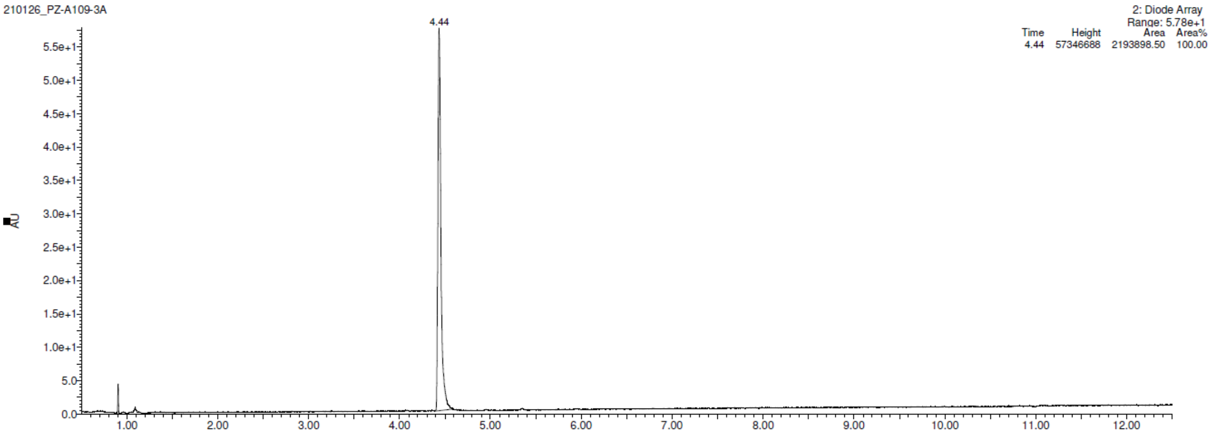

Figure S89. HPLC of **PP 22**

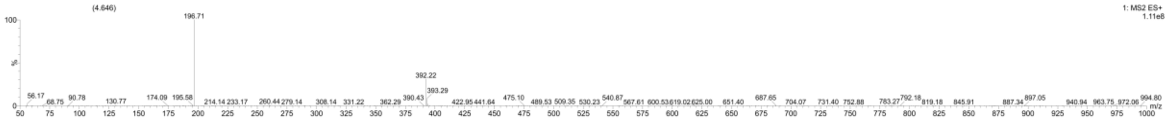

Figure S90. MS of **PP 22**

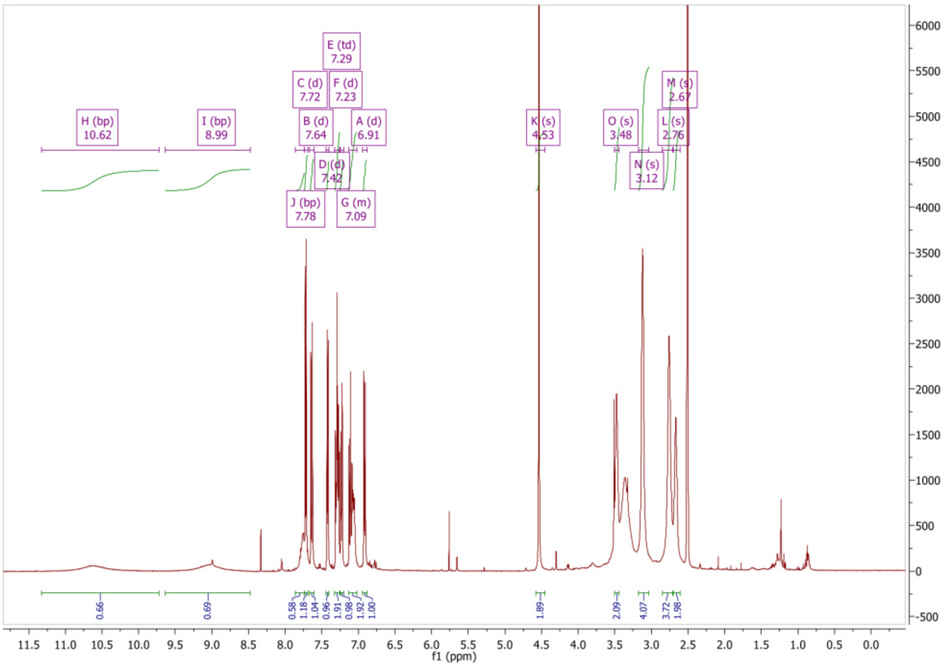

Figure S91. ^{1}H NMR of **PP 22**

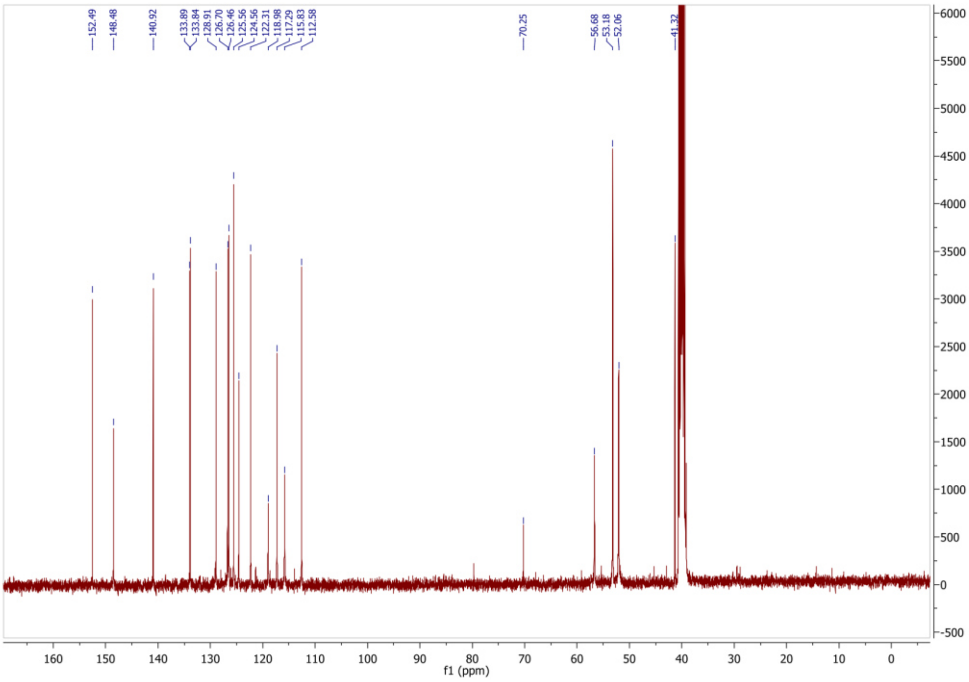

Figure S92. ^{13}C NMR of **PP 22**

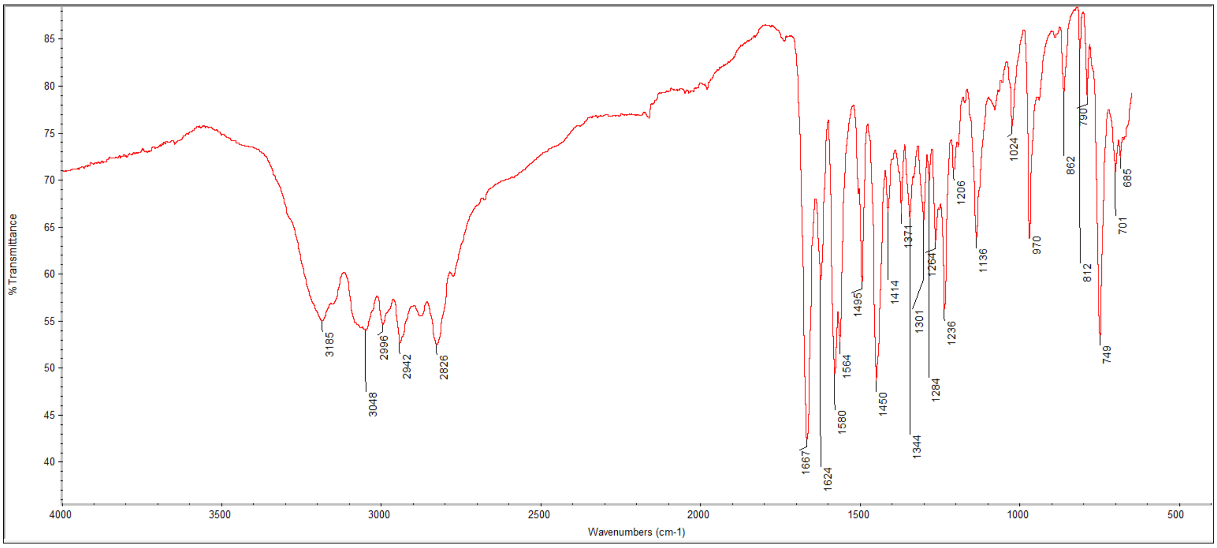

Figure S93. FT IR of **PP 22**

N[6]-(1,2-benzothiazol-3-yl)-*N*[1]-(3,4-dihydroquinazolin-2-yl)hexane-1,6-diamine PP 23

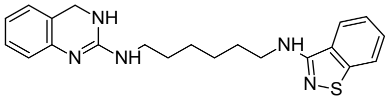

Figure S94. Structure of **PP 23**

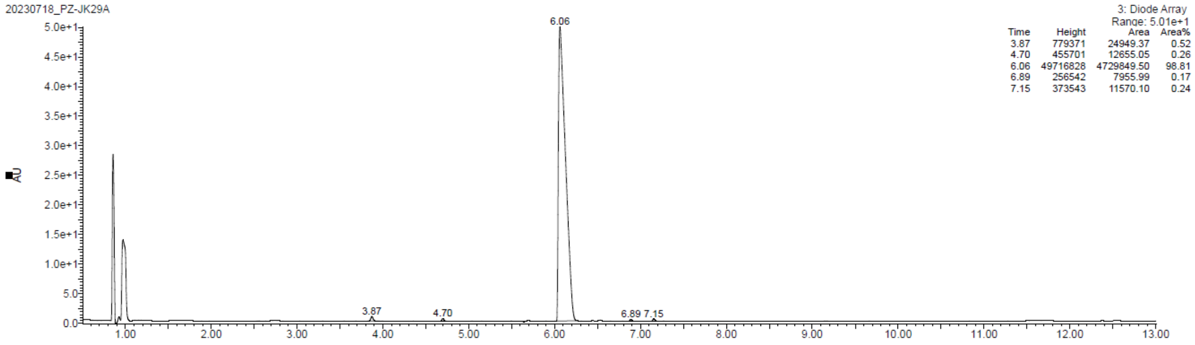

Figure S95. HPLC of **PP 23**

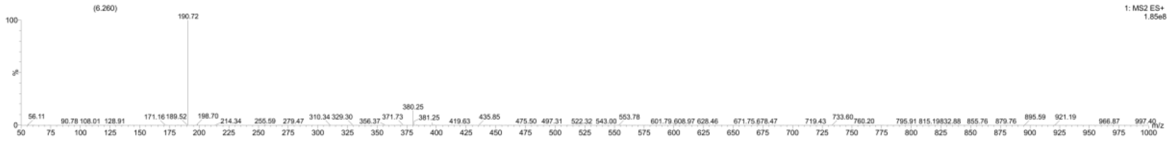

Figure S96. MS of **PP 23**

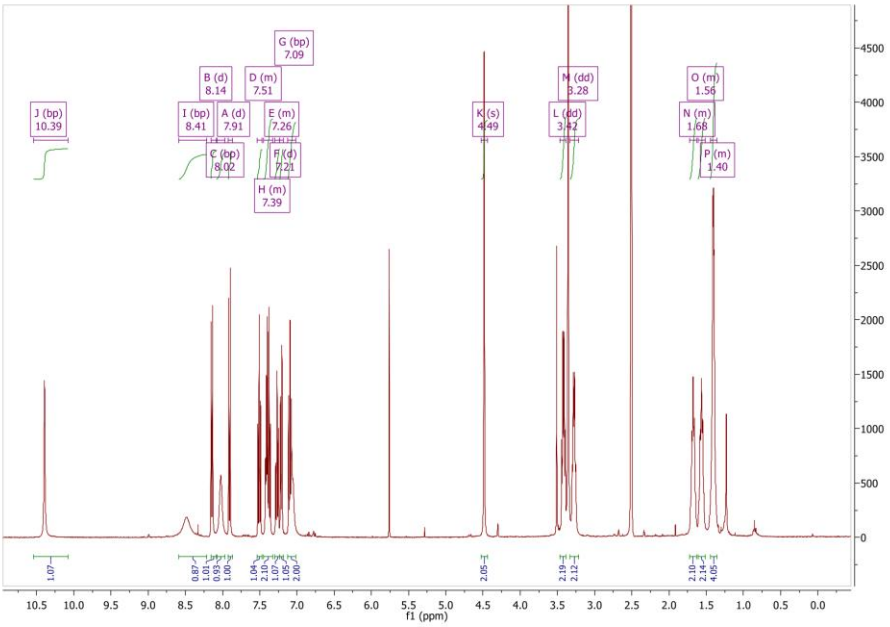

Figure S97. ^{1}H NMR of **PP 23**

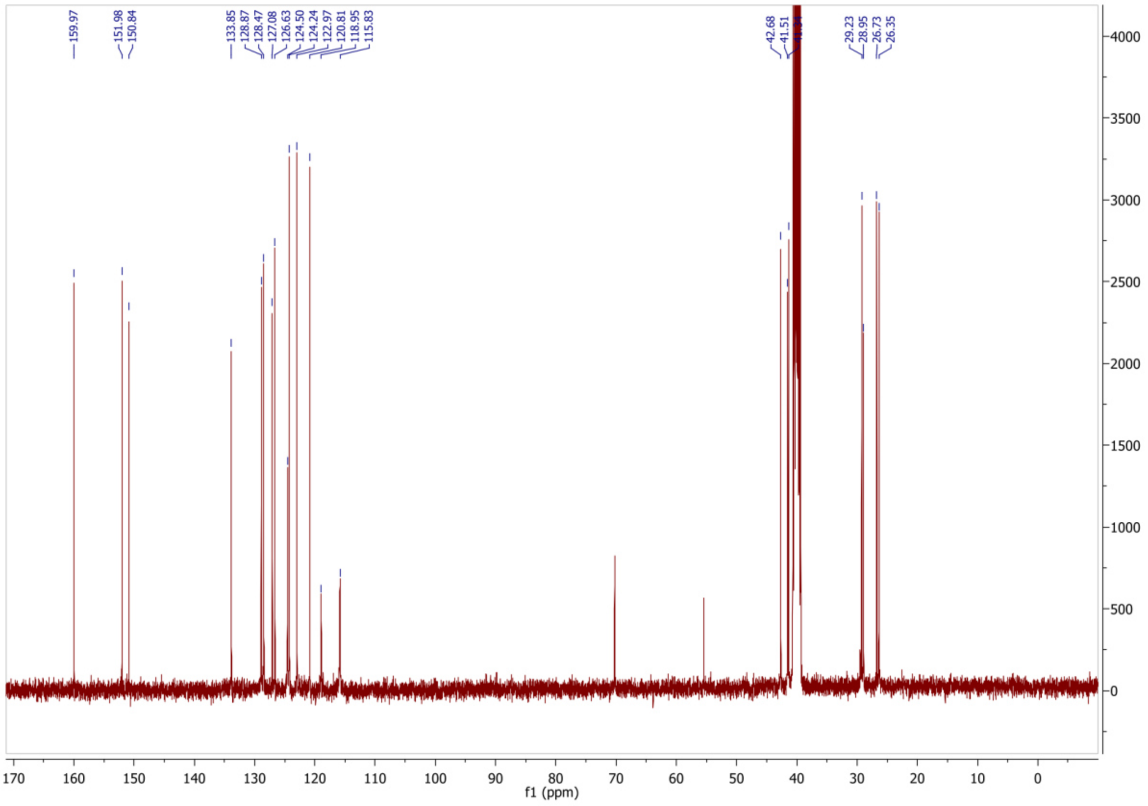

Figure S98. ^{13}C NMR of **PP 23**

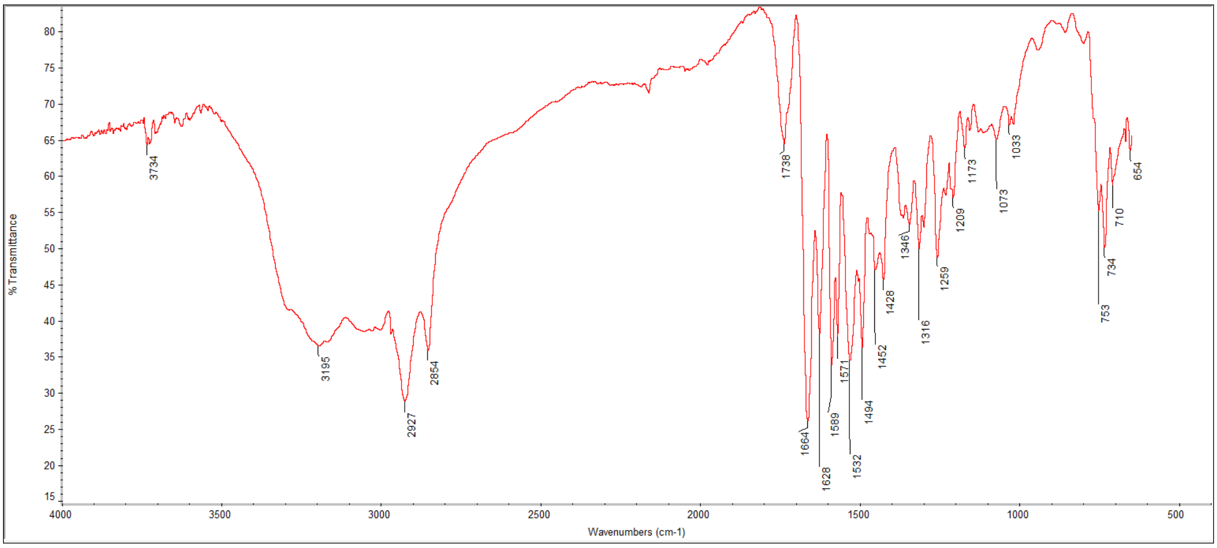

Figure S99. FT IR of **PP 23**

3-{6-[(3,4-dihydroquinazolin-2-yl)amino]hexyl}-2λ^6-thia-3-azatricyclo[6.3.1.0^{4,12}]dodeca-1(11),4(12),5,7,9-pentaene-2,2-dione PP 24

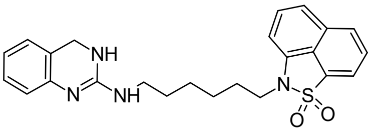

Figure S100. Structure of **PP 24**

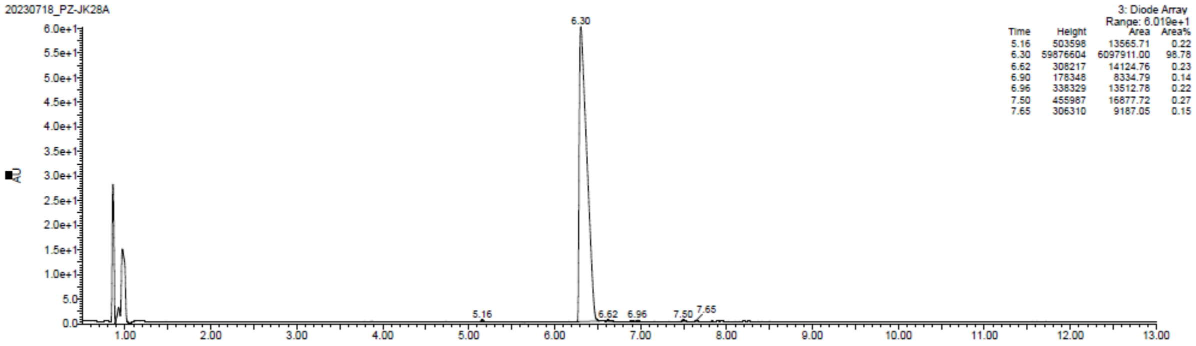

Figure S101. HPLC of **PP 24**

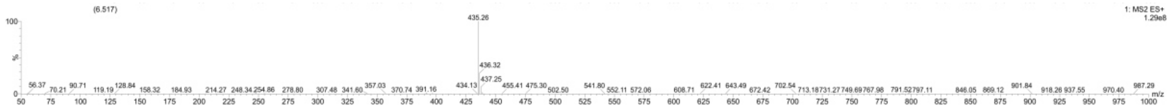

Figure S102. MS of **PP 24**

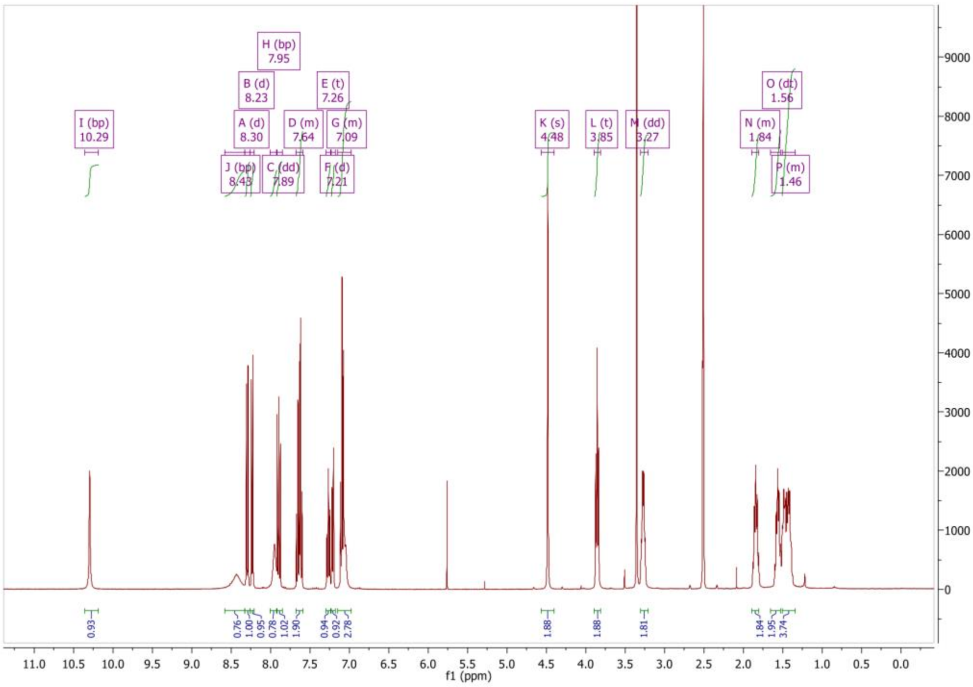

Figure S103. ^{1}H NMR of **PP 24**

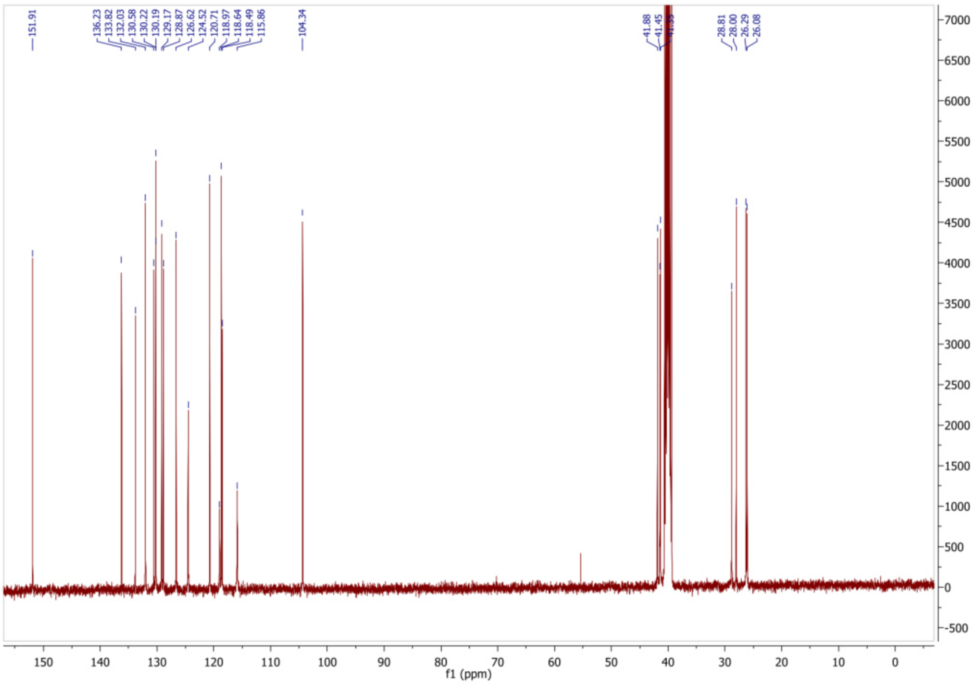

Figure S104. ^{13}C NMR of **PP 24**

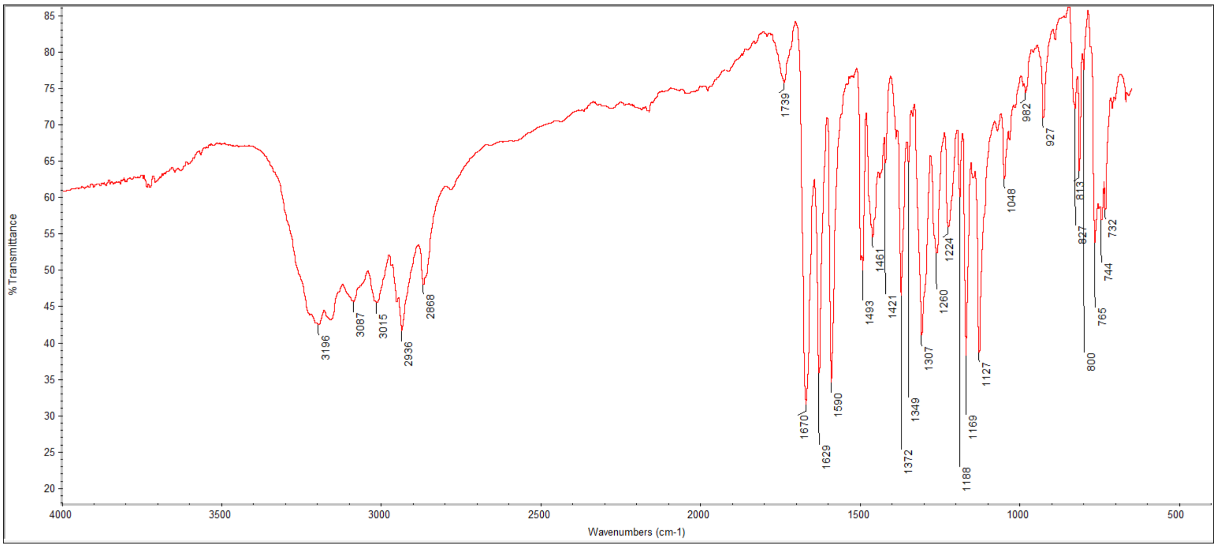

Figure S105. FT IR of **PP 24**

Supplementary materials - Biological research

1. Cytotoxicity

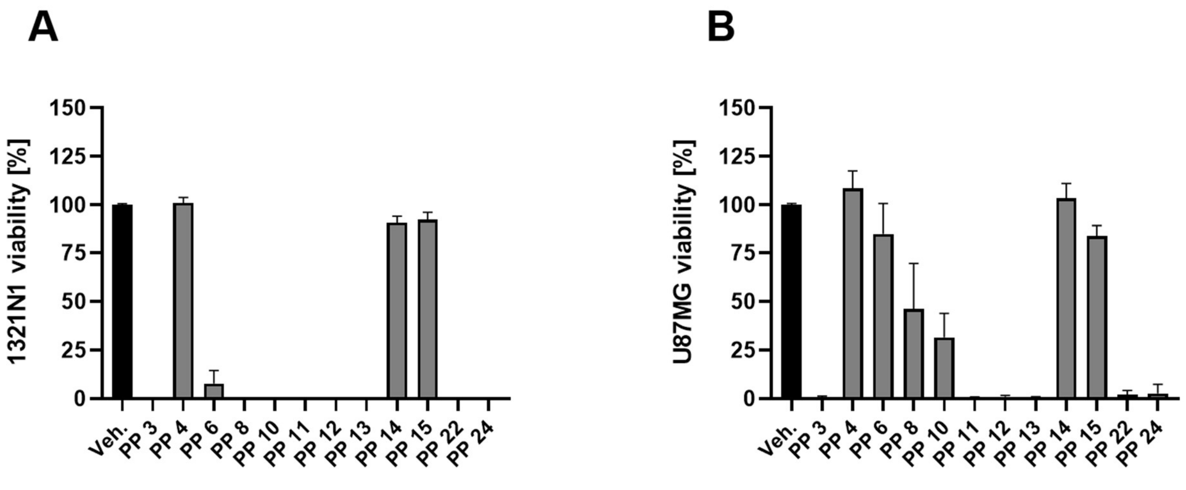

Figure S106. Effect of tested ligands on the viability on the **A**) 1321 N1 human astrocytoma cell line **B**) U87MG glioblastoma multiforme line. The cells were exposed to either vehicle (0.1 % DMSO), or inhibitor in 25 µM for 72 h. Then, cell viability was assessed by the means of MTS assay.

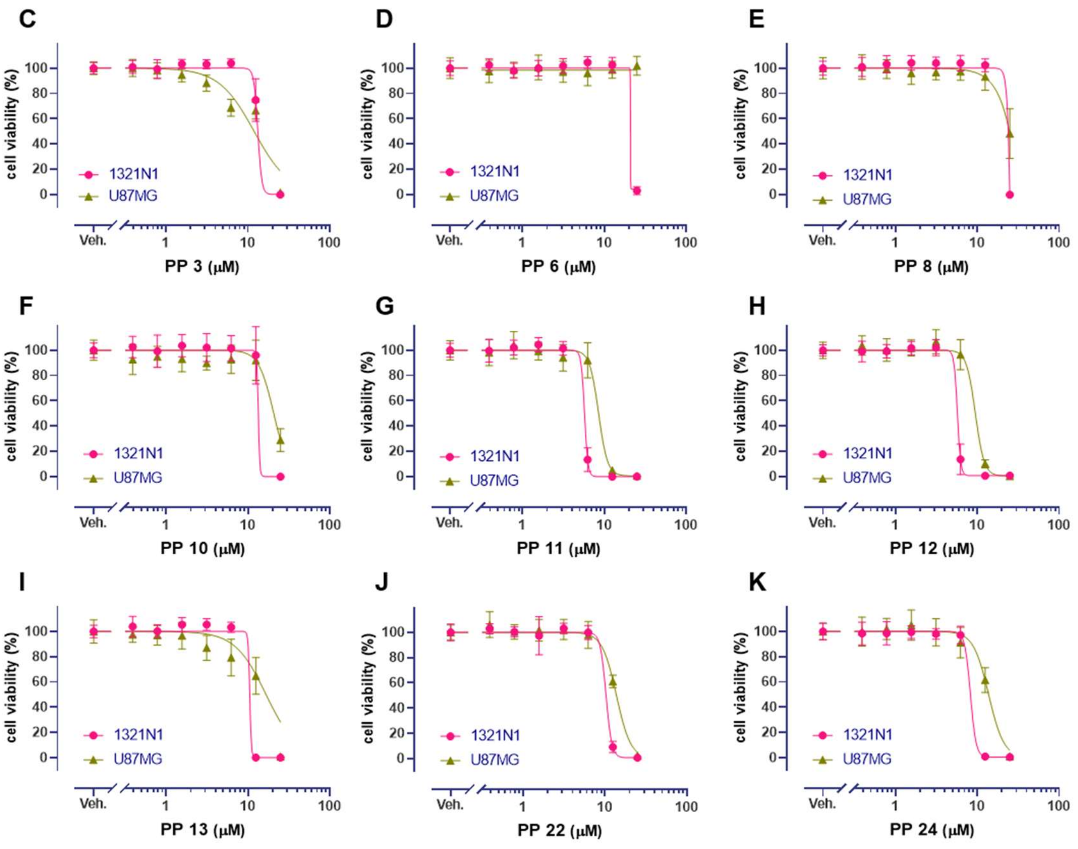

Figure S107. Effect of tested ligands on the viability on the 1321 N1 human astrocytoma cell line and U87MG glioblastoma multiforme line for **C**) **PP 3**; D) **PP 6; E) PP 8; F**) **PP 10; G**) **PP 11; H**) **PP**

12; I) **PP 13; J**) **PP 22; K**) **PP 24**. The cells were exposed to either vehicle (0.1 % DMSO), or inhibitor for 24 h. Then, cell viability was assessed by the means of MTS assay. The IC_50 were calculated as a dose that causes a 50 % decrease in cell viability relative to the maximum inhibition observed.

2. Solubility

Table S1. Solubility in DMSO and 10% DMSO in PBS buffer

	100% DMSO, 10 mM	10% DMSO, 1 mM
**PP 10**	**+ + +**	**+ + +**
**PP 15**	**+ + -**	**+ + -**
**PP 13**	**+ + +**	**+ + +**
**PP 24**	**+ + +**	**+ + +**

3. Absorption – PAMPA permeability test

Table S2. PAMPA results

**Comp.**	***Pe**** (10^{-6} cm/s)
CFN*	8.23
**PP 10**	**0.03**
**PP 15**	**0.13**
**PP 13**	**0.19**
**PP 24**	**0.73**

*Permeability coefficient. Compound is permeable if **Pe≥1.5×10^{-6} cm/s**
**CFN = caffeine, well-permeable control

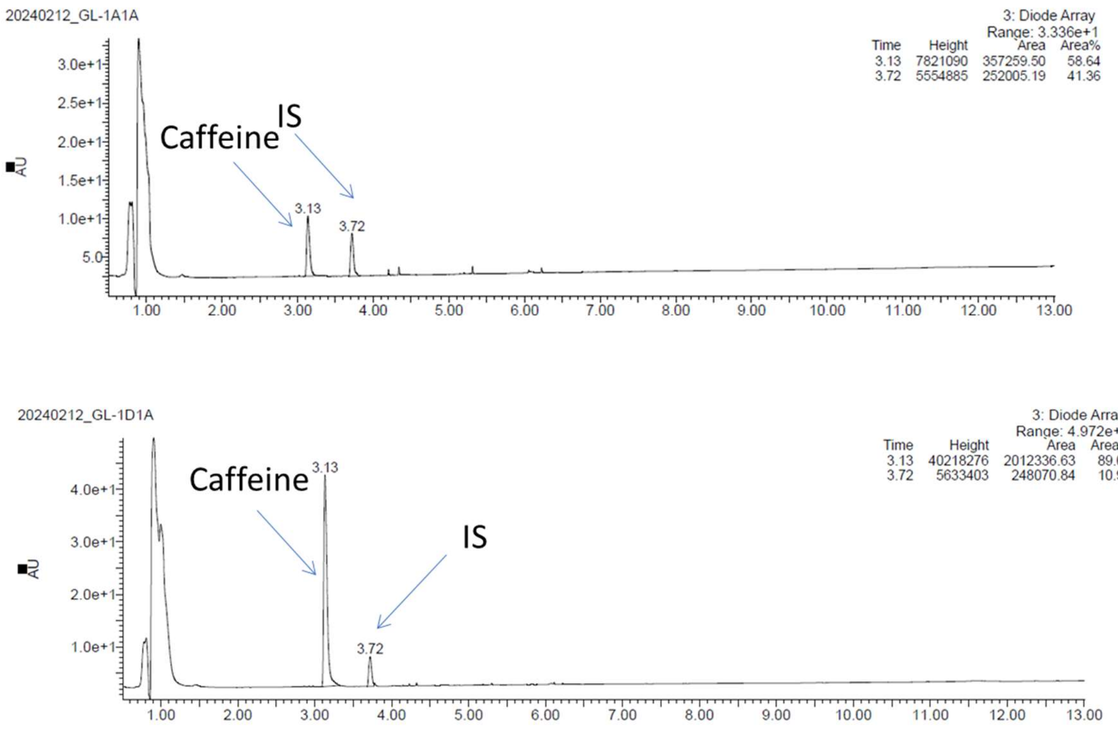

Figure S108. UPLC of **Caffeine** solution in PBS (pH=7.4) after 5h of incubation: acceptor well (above), donor well (below). IS – internal standard.

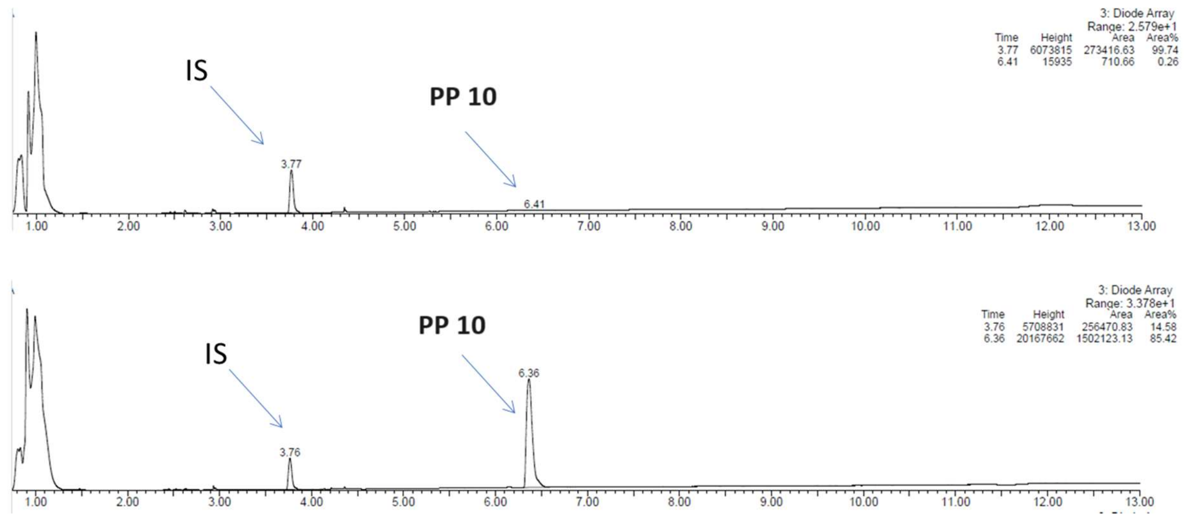

Figure S109. UPLC of **PP 10** solution in PBS (pH=7.4) after 5h of incubation: acceptor well (above), donor well (below). IS – internal standard.

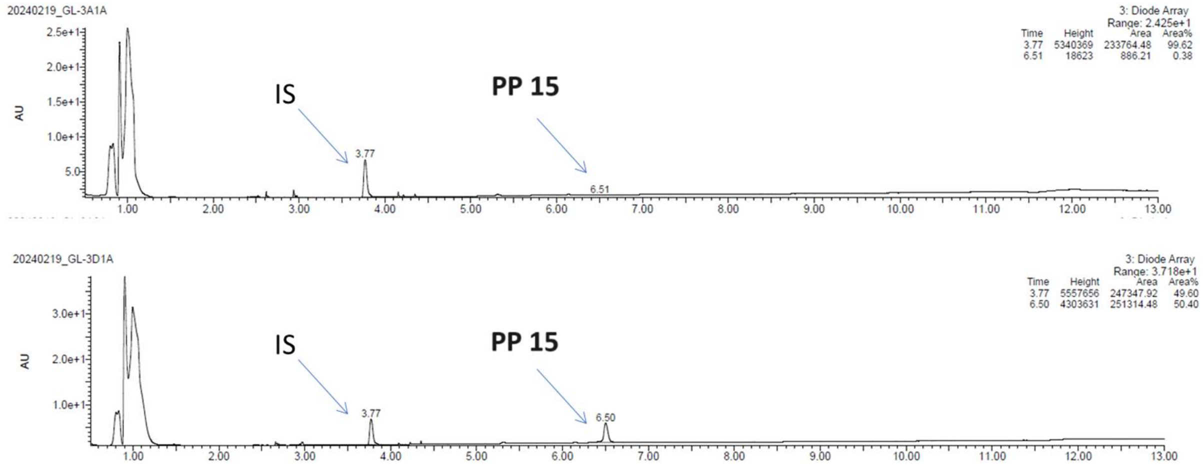

Figure S110. UPLC of **PP 15** solution in PBS (pH=7.4) after 5h of incubation: acceptor well (above), donor well (below). IS – internal standard.

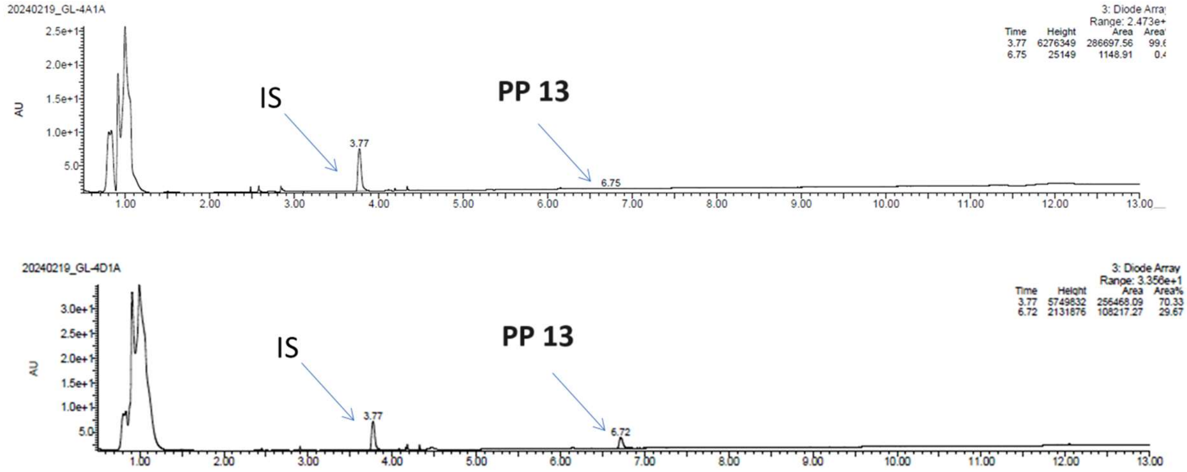

Figure S111. UPLC of **PP 13** solution in PBS (pH=7.4) after 5h of incubation: acceptor well (above), donor well (below). IS – internal standard.

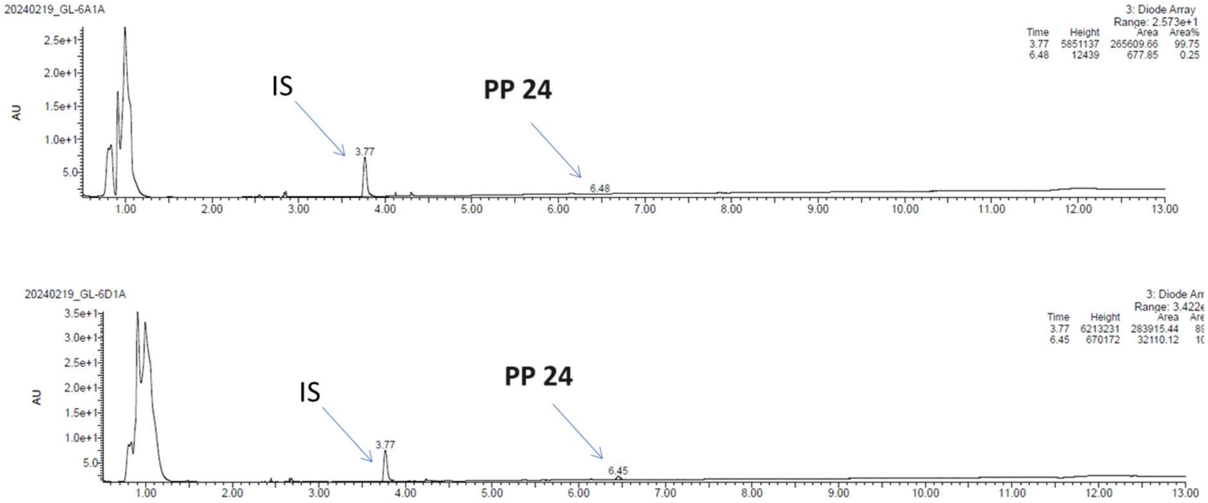

Figure S112. UPLC of **PP 24** solution in PBS (pH=7.4) after 5h of incubation: acceptor well (above), donor well (below). IS – internal standard.

4. Metabolic stability

Table S3. Metabolic stability summary: the % remaining, molecular masses, and metabolic pathways of tested compounds and **Verapamil** (reference unstable drug) after incubation with mouse liver microsomes (MLMs). Main metabolic pathways are marked in red.

Substrate	Molecular mass (*m/z*)	% remaining	Molecular mass of the metabolite (*m/z*)	Metabolic pathway
**PP 6**	450.15	**72.76**	466.12 (**M1**)	***hydroxylation***
			484.07 (**M2**)	*double hydroxylation and double bound reduction*
			464.06 (**M3**)	*oxidation*
			484.54 (**M4**)	*double hydroxylation and double bound reduction*
			465.98 (**M5**)	*hydroxylation*
			450.09 (**M6**)	*dehydrogenation/double bound reduction*
			464.32 (**M7**)	*oxidation*
**PP 10**	451.15	**66.60**	485.21 (**M1**)	***double hydroxylation and double bound reduction or double hydroxylation and ring opening***
			500.90 (**M2**)	*M1+hydroxylation*
			467.25 (**M3**)	*hydroxylation*
			467.25 (**M4**)	*decomposition and hydroxylation*
			502.10 (**M5**)	*M1+hydroxylation*
			484.81 (**M6**)	*double hydroxylation and double bound reduction or double hydroxylation and ring opening*
			467.18 (**M7**)	*hydroxylation*
			276.21 (**M8**)	*decomposition*
			501.37 (**M9**)	*M1+hydrozylation*
			499.17 (**M10**)	*triple hydroxylation*
			278.34 (**M11**)	*decomposition*
			500.90 (**M12**)	*M1+hydrozylation*
			465.12 (**M13**)	*oxidation*
			479.35 (**M14**)	*double oxidation*
**PP 11**	469.24	**74.22**	485.14 (**M1**)	***hydroxylation***
			503.03 (**M2**)	*double hydroxylation and double bound reduction*
			503.16 (**M3**)	*double hydroxylation and double bound reduction*
			518.99 (**M4**)	*triple hydroxylation and double bound reduction*
			467.25 (**M5**)	*dehydrogenation*
			471.64 (**M6**)	*double bound reduction*
**PP 12**	519.19	**76.87**	344.06 (**M1**)	***decomposition/oxidation***
			504.43 (**M2**)	*decomposition/ring opening*
			229.97 (**M3**)	*decomposition*
			230.24 (**M4**)	*decomposition/hydroxylation*
			552.18 (**M5**)	*double hydroxylation*
			553.18 (**M6**)	*double hydroxylation*
			554.97 (**M7**)	*double hydroxylation and double bound reduction*

**PP 13**	485.27	**52.60**	407.25 (**M1**)	***ring opening and triple hydroxylation***
			501.23 (**M2**)	*hydroxylation*
			519.19 (**M3**)	*double hydroxylation and double bound reduction*
			535.42 (**M4**)	*triple hydroxylation and double bound reduction*
			501.10 (**M5**)	*hydroxylation*
			274.08 (**M6**)	*decomposition*
			276.21 (**M7**)	*decomposition*
			535.22 (**M8**)	*triple hydroxylation and double bound reduction*
			535.35 (**M9**)	*triple hydroxylation and double bound reduction*
			276.48 (**M10**)	*decomposition*
			498.97 (**M11**)	*oxidation*
**PP 15**	435.12	**48.07**	451.15 (**M1**)	***hydroxylation***
			195.04 (**M2**)	*decomposition*
			467.11 (**M3**)	*double hydroxylation*
			467.31 (**M4**)	*double hydroxylation*
			453.35 (**M5**)	*hydroxylation and double bound reduction*
			260.11 (**M6**)	*decomposition*
			469.04 (**M7**)	*double hydroxylation and double bound reduction*
			262.17 (**M8**)	*decomposition*
			467.31 (**M9**)	*double hydroxylation*
			467.11 (**M10**)	*double hydroxylation*
			469.24 (**M11**)	*double hydroxylation and double bound reduction*
			450.29 (**M12**)	*oxidation*
			450.89 (**M13**)	*oxidation*
			467.31 (**M14**)	*double hydroxylation*
			451.09 (**M15**)	*hydroxylation*
			485.21 (**M16**)	*triple hydroxylation and double bound reduction*
			485.27 (**M17**)	*triple hydroxylation and double bound reduction*
**PP 24**	435.19	**93.13**	451.15 (**M1**)	***hydroxylation***
			451.15 (**M2**)	*hydroxylation*
			449.36 (**M3**)	*oxidation*
			449.16 (**M4**)	*oxidation*
			449.22 (**M5**)	*oxidation*
			449.02 (**M6**)	*oxidation*
			448.82 (**M7**)	*oxidation*
			465.39 (**M8**)	*oxidation and hydroxylation*
**Verapamil***	455.54	**23.93**	441.42 (**M1**)	***demethylation***
			441.42 (**M2**)	*demethylation*
			291.35 (**M3**)	*defragmentation*
			293.34 (**M4**)	*decomposition/hydroxylation*
			277.33 (**M5**)	*defragmentation*

* Reference unstable drug

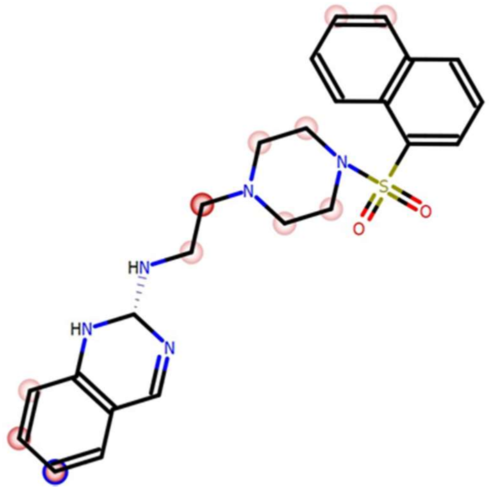

Figure S113. The MetaSite 6.0.1. software prediction of the most probable sites of compound **PP 6** metabolism. The darker red color - the higher probability to be involved in the metabolism pathway. The blue circle marked the site of compound with the highest probability of metabolic bioconversion.

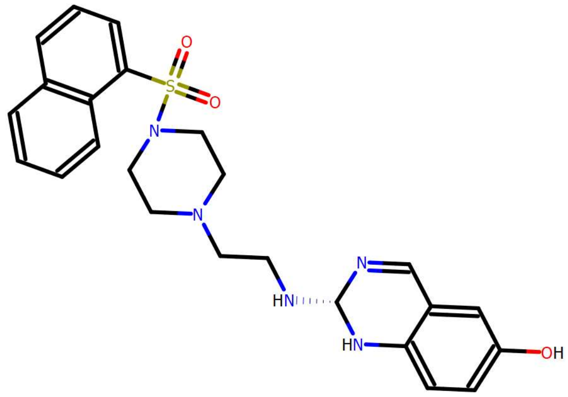

Figure S114. The MetaSite 6.0.1. software prediction of the most probable hydroxylation site of **PP 6**

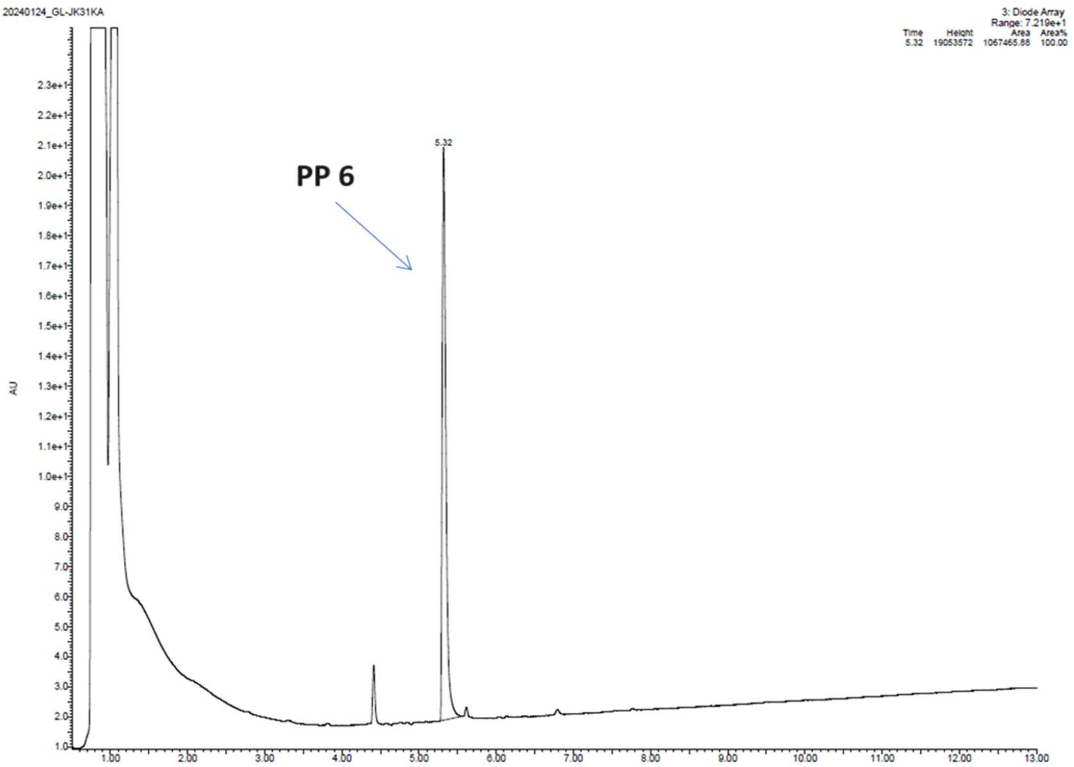

Figure S115. UPLC spectra after 120 min incubation of compound **PP 6** in buffer. One contamination found.

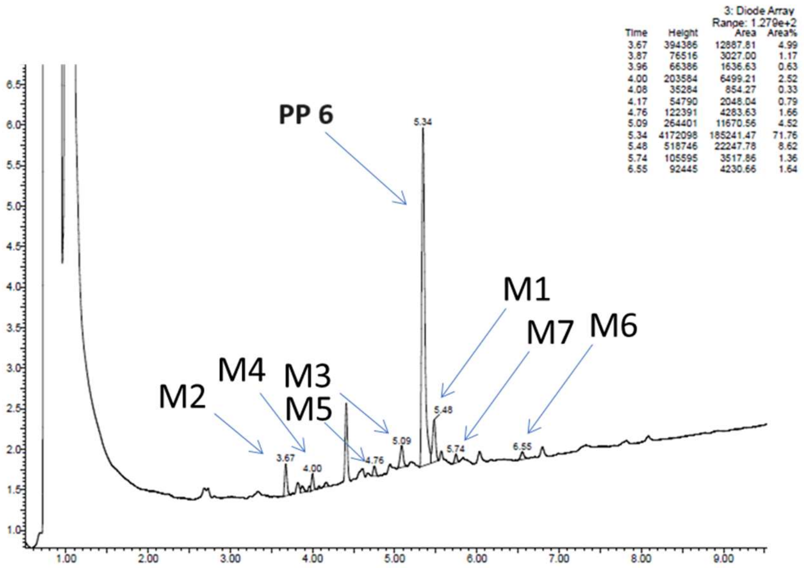

Figure S116. UPLC spectra after 120 min incubation of compound **PP 6** with MLMs.

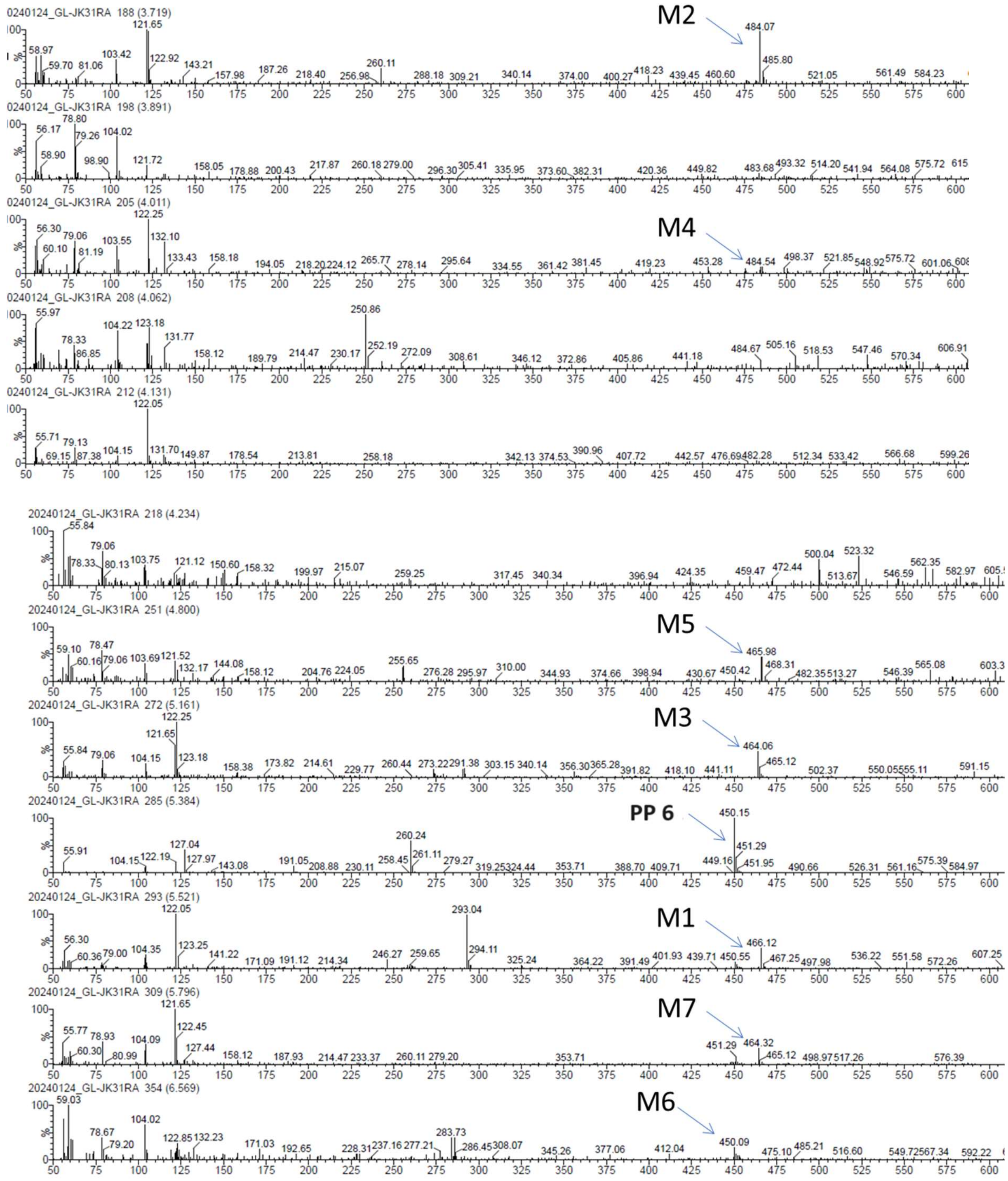

Figure S117. MS analyses of **PP 6** and metabolites.

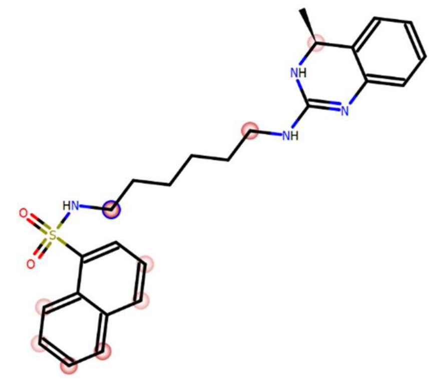

Figure S118. The MetaSite 6.0.1. software prediction of the most probable sites of compound **PP 10** metabolism. The darker red color - the higher probability to be involved in the metabolism pathway. The blue circle marked the site of compound with the highest probability of metabolic bioconversion.

Figure S119. UPLC spectra after 120 min incubation of compound **PP 10** in buffer.

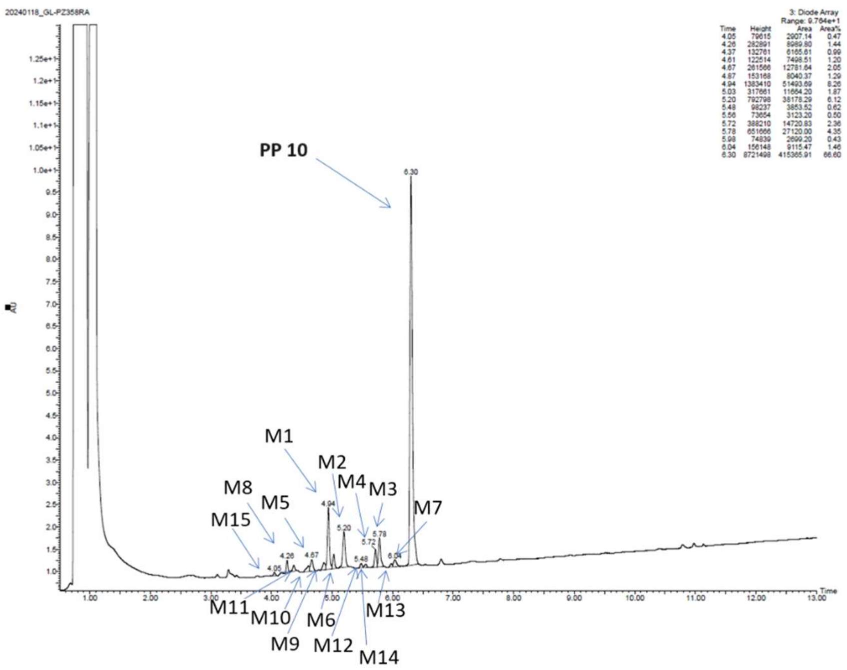

Figure 120. UPLC spectra after 120 min incubation of compound **PP 10** with MLMs.

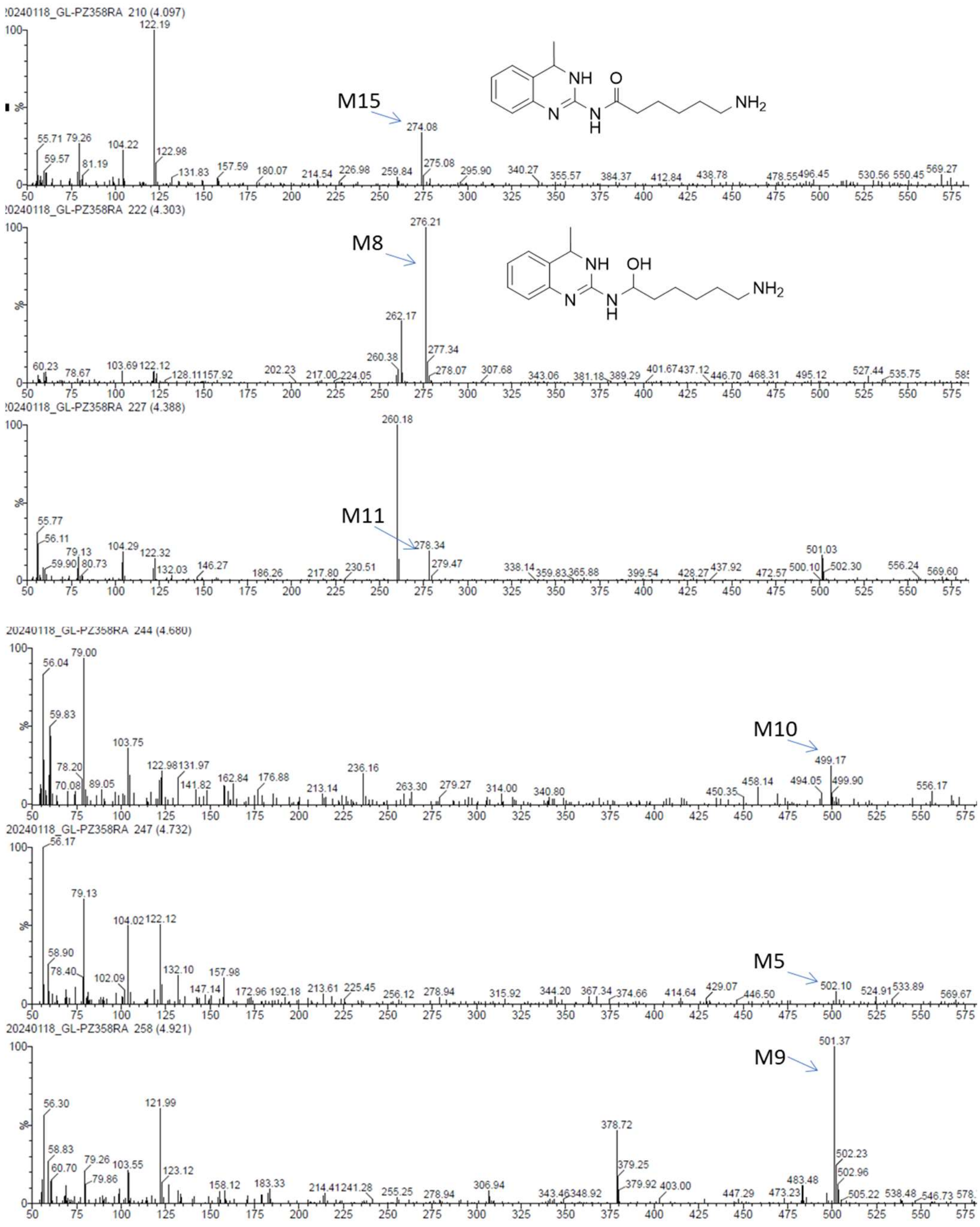
20240118_GL-PZ358RA 210 (4.097)
M15
274.08
NH
O
N
N
H
NH2
20240118_GL-PZ358RA 222 (4.303)
M8
276.21
NH
OH
N
N
H
NH2
20240118_GL-PZ358RA 227 (4.388)
M11
278.34
20240118_GL-PZ358RA 244 (4.680)
M10
499.17
20240118_GL-PZ358RA 247 (4.732)
M5
502.10
20240118_GL-PZ358RA 258 (4.921)
M9
501.37

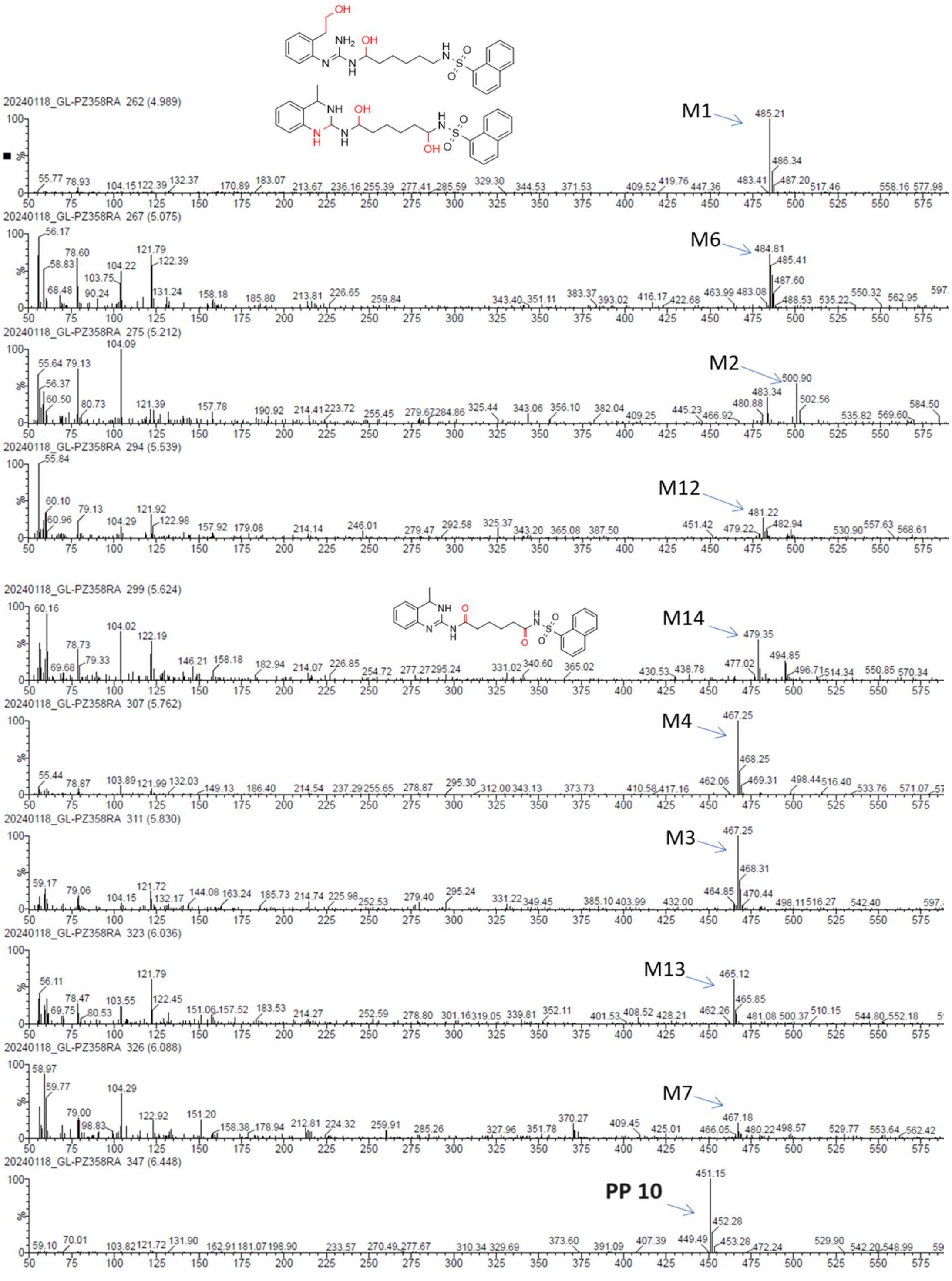

Figure S121. MS analyses of **PP 10** and metabolites.

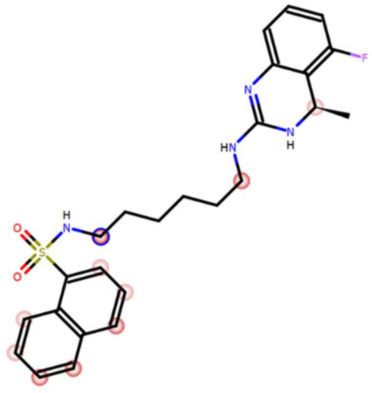

Figure S122. The MetaSite 6.0.1. software prediction of the most probable sites of compound **PP 11** metabolism. The darker red color - the higher probability to be involved in the metabolism pathway. The blue circle marked the site of compound with the highest probability of metabolic bioconversion.

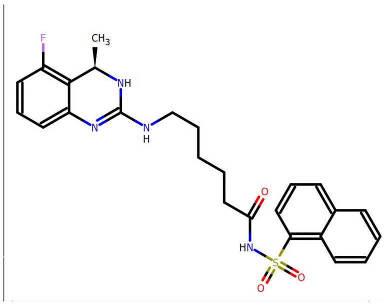

Figure S123. The MetaSite 6.0.1. software prediction of the most probable oxidation of **PP 11**

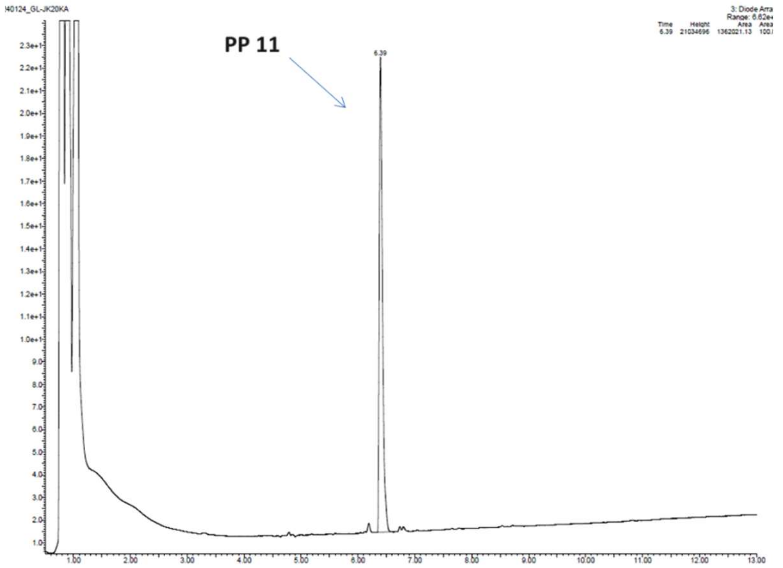

Figure S124. UPLC spectra after 120 min incubation of compound **PP 11** in buffer.

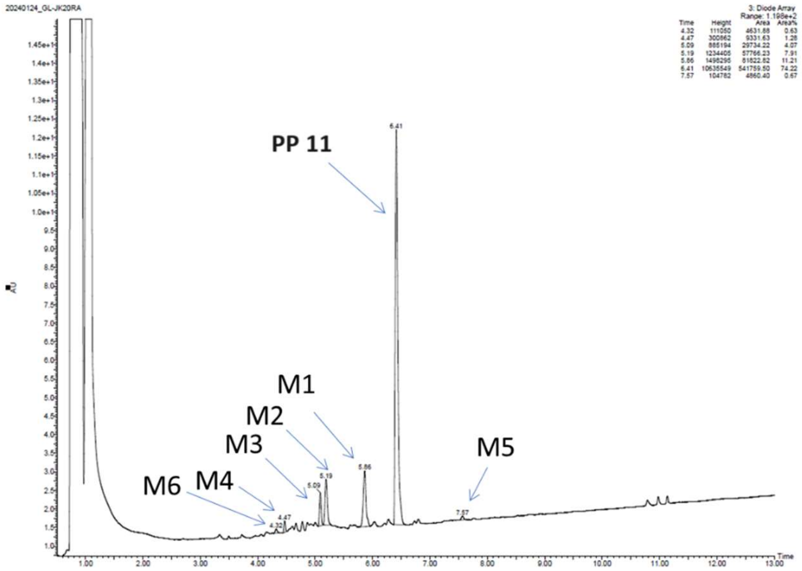

Figure S125. UPLC spectra after 120 min incubation of **PP 11** with MLMs.

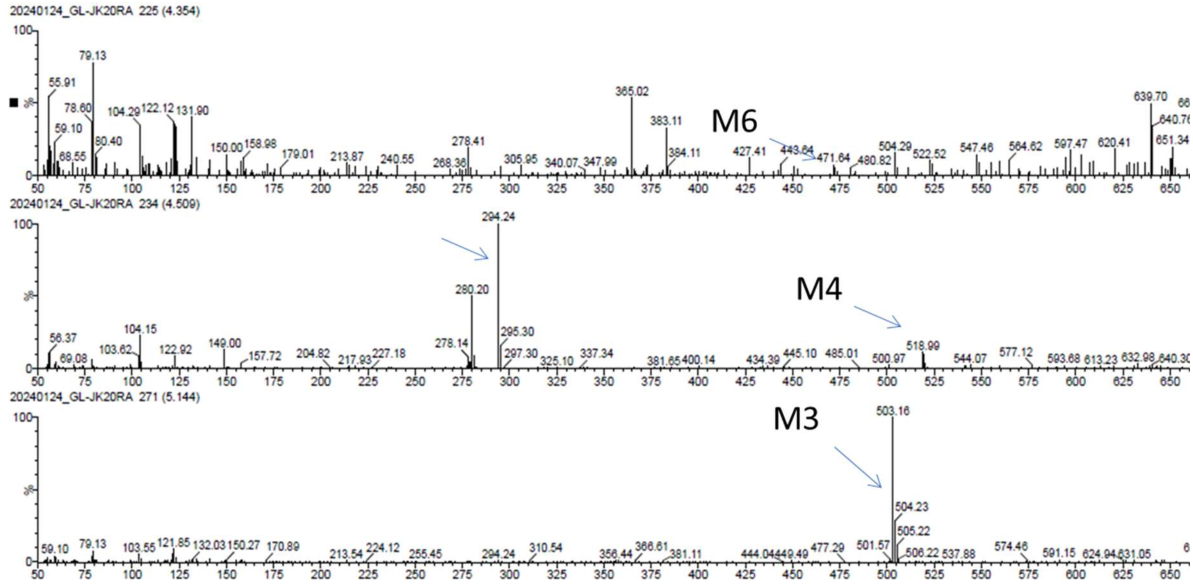

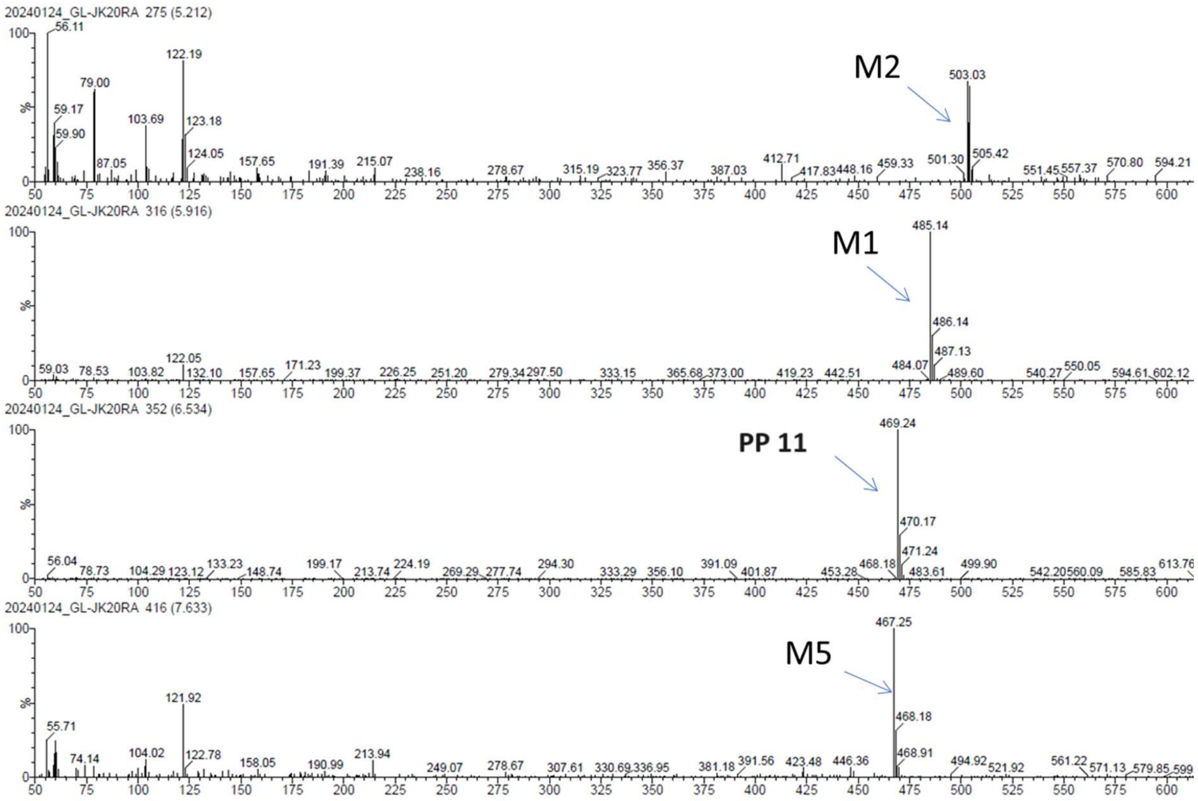

Figure S126. MS analyses of **PP 11** and metabolites.

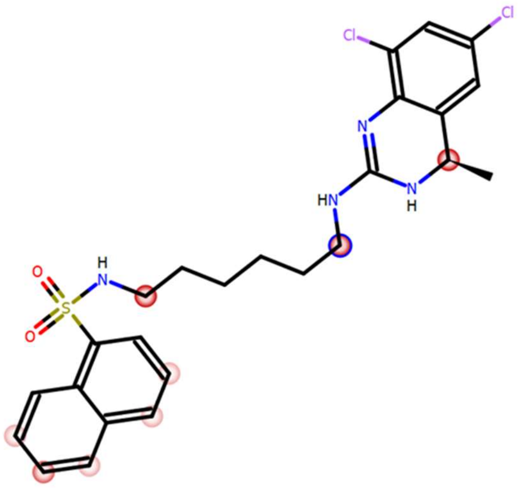

Figure S127. The MetaSite 6.0.1. software prediction of the most probable sites of compound **PP 12** metabolism. The darker red color - the higher probability to be involved in the metabolism pathway. The blue circle marked the site of compound with the highest probability of metabolic bioconversion.

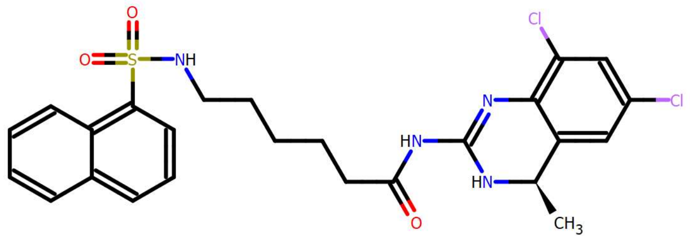

Figure S128. The MetaSite 6.0.1. software prediction of the most probable oxidation site of **PP 12.**

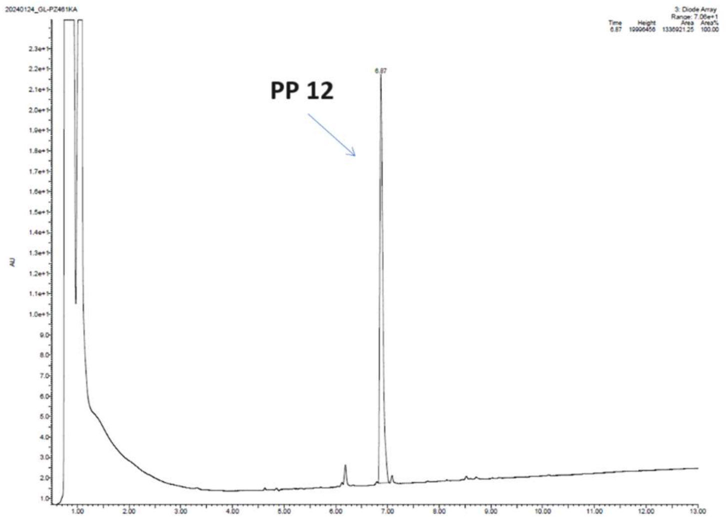

Figure S129. UPLC spectra after 120 min incubation of compound **PP 12** in buffer.

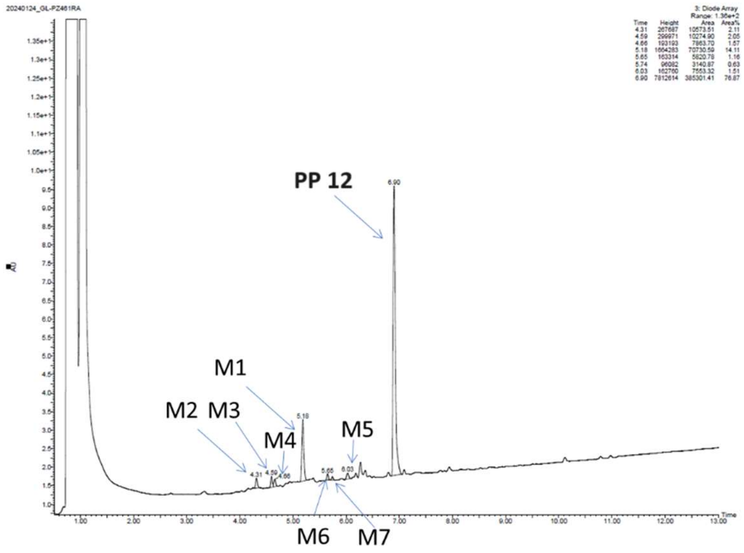

Figure S130. UPLC spectra after 120 min incubation of compound **PP 12** with MLMs.

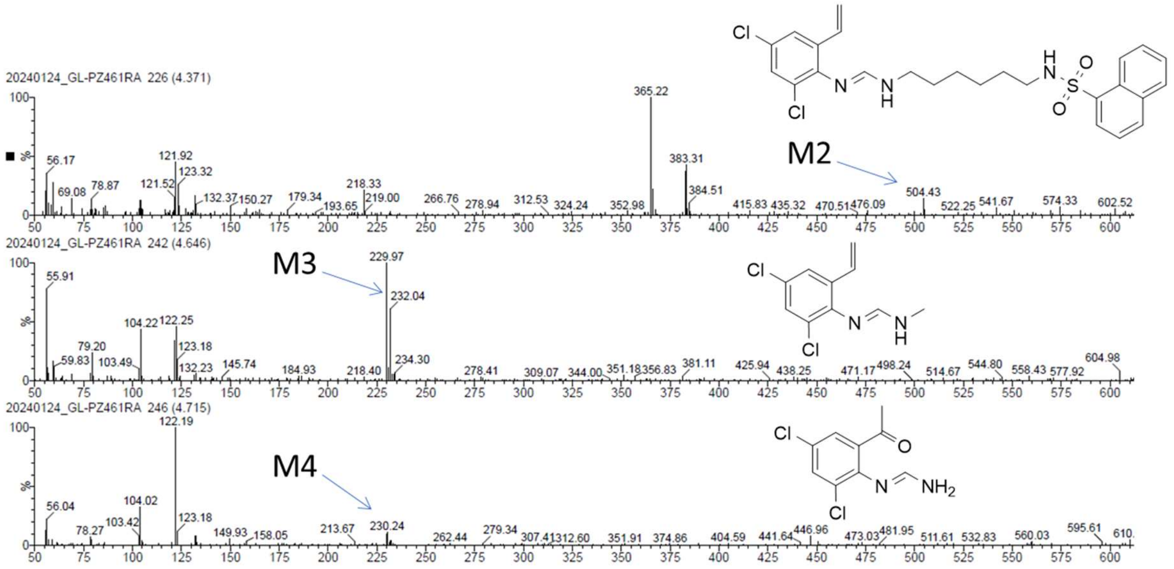

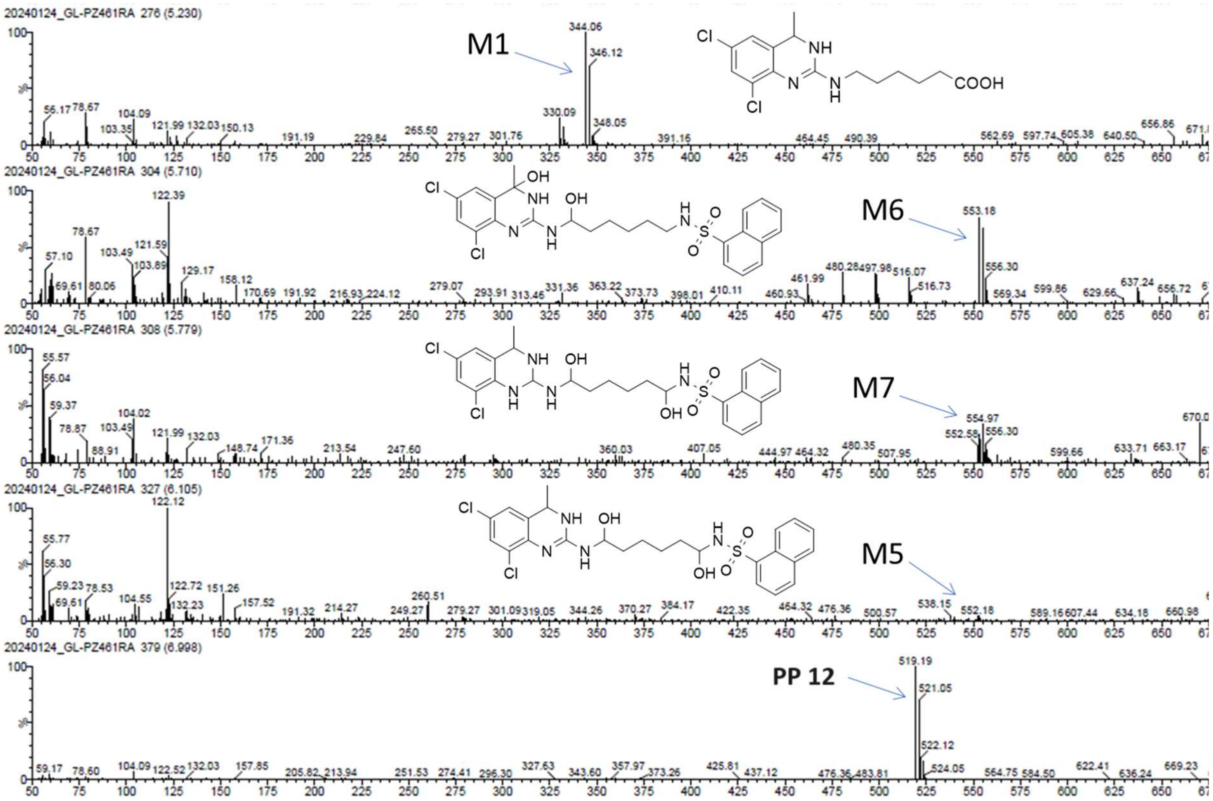

Figure S131. MS analyses of **PP 12** and metabolites.

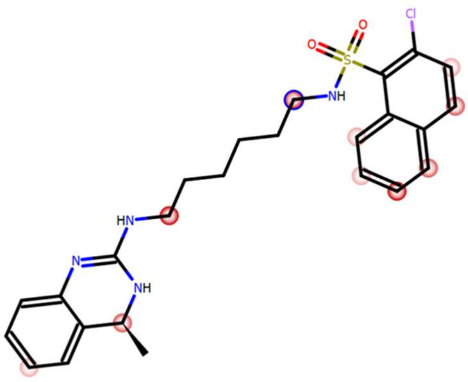

Figure S132. The MetaSite 6.0.1. software prediction of the most probable sites of compound **PP 13** metabolism. The darker red color - the higher probability to be involved in the metabolism pathway. The blue circle marked the site of compound with the highest probability of metabolic bioconversion.

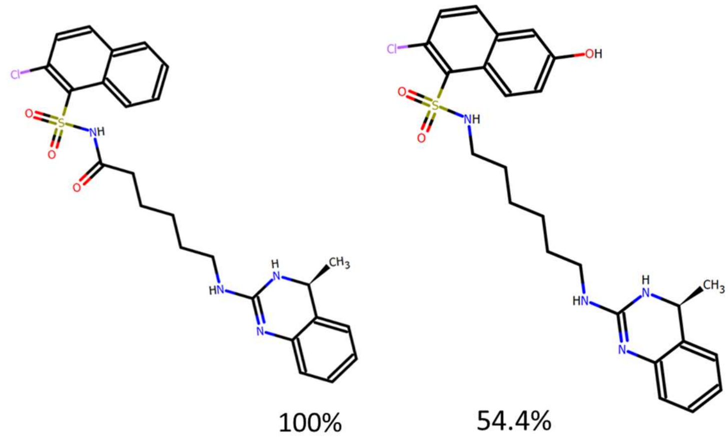

100% 54.4%

Figure S133. The MetaSite 6.0.1. software prediction of the most probable hydroxylation/oxidation site of **PP 13**

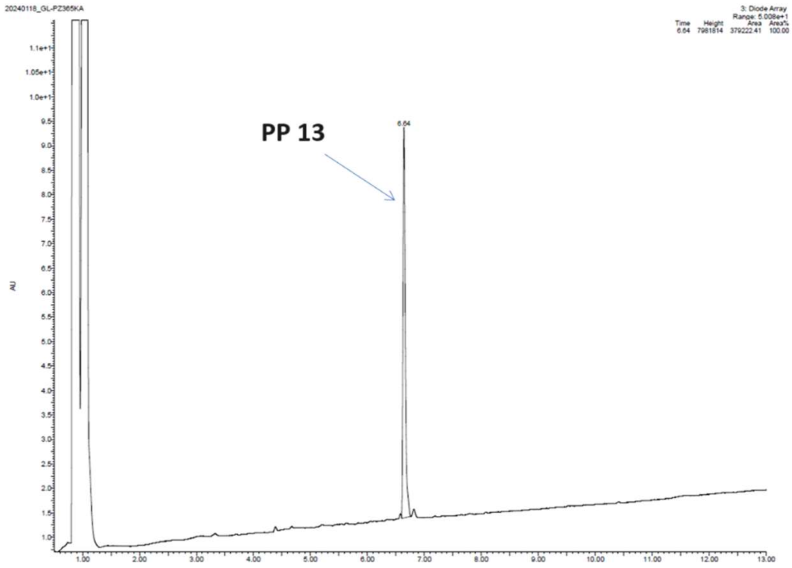

Figure S134. UPLC spectra after 120 min incubation of compound **PP 13** in buffer

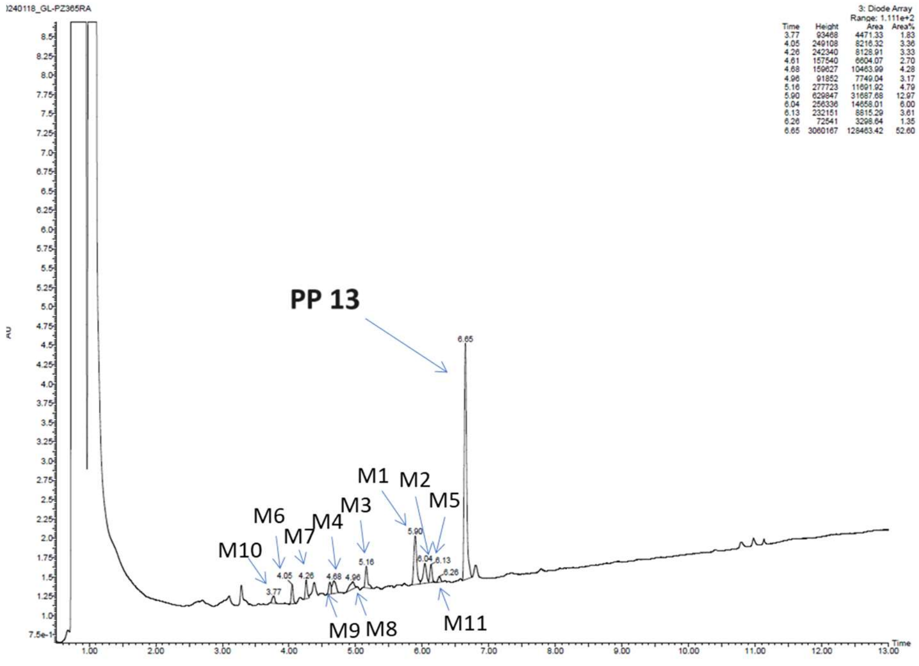

Figure S135. UPLC spectra after 120 min incubation of compound **PP 13** with MLMs.

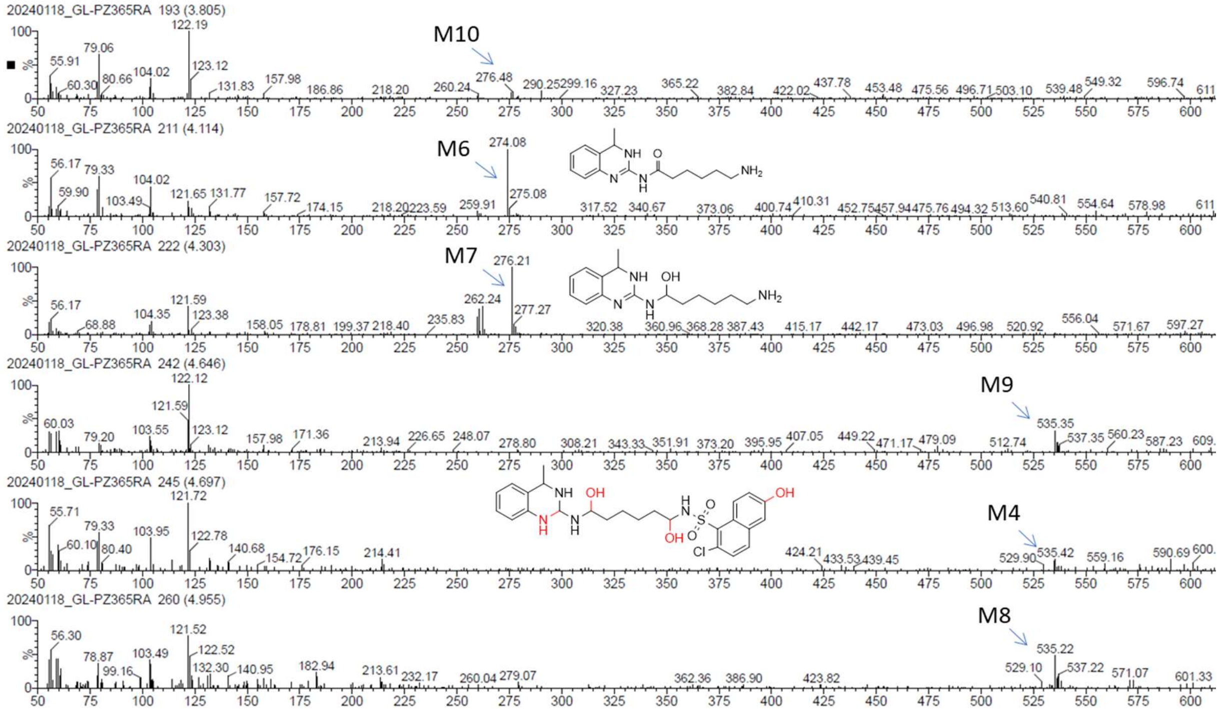

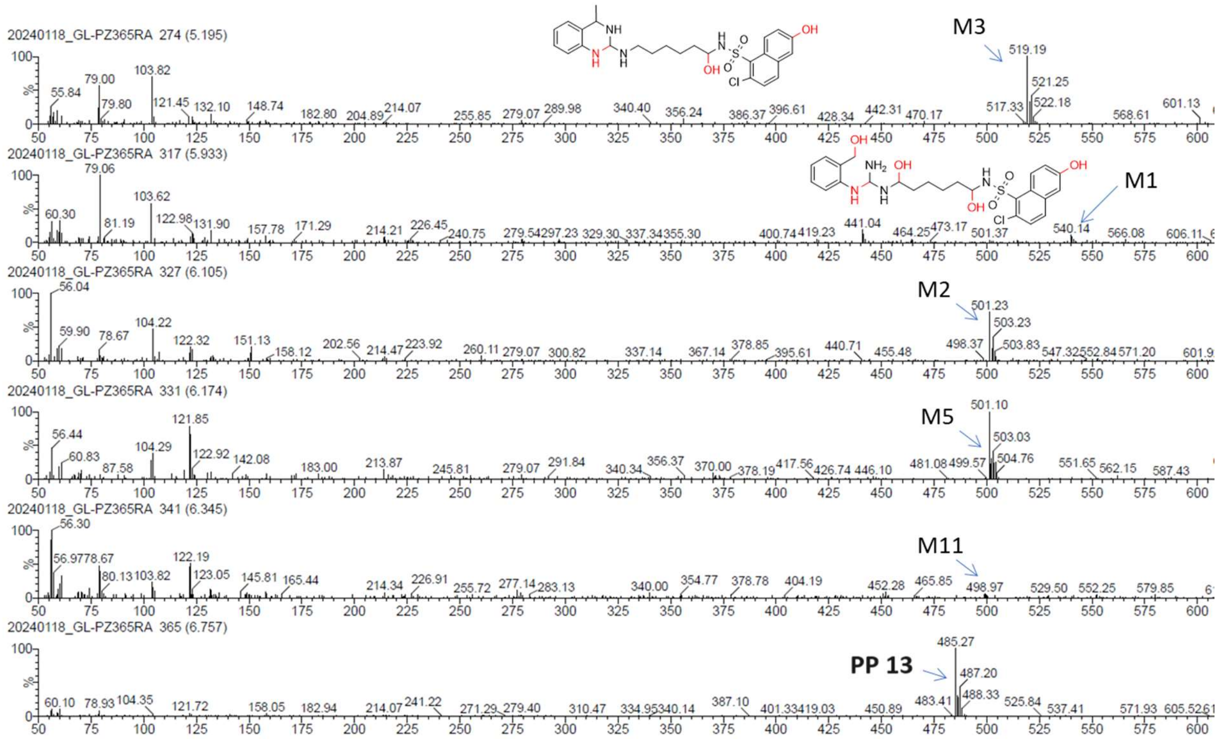

Figure S136. MS analyses of **PP 13** and metabolites.

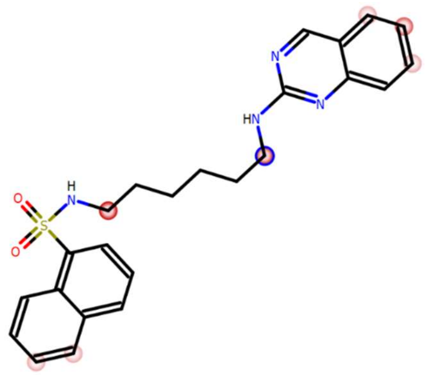

Figure S137. The MetaSite 6.0.1. software prediction of the most probable sites of compound **PP 15** metabolism. The darker red color - the higher probability to be involved in the metabolism pathway. The blue circle marked the site of compound with the highest probability of metabolic bioconversion.

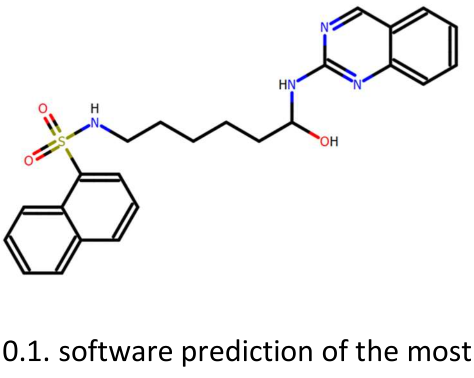

Figure S138. The MetaSite 6.0.1. software prediction of the most probable hydroxylation site of **PP 15**

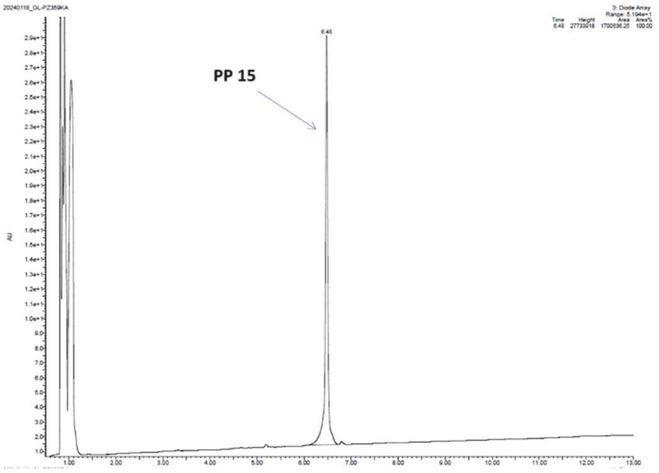

Figure S139. UPLC spectra after 120 min incubation of compound **PP 15** in buffer.

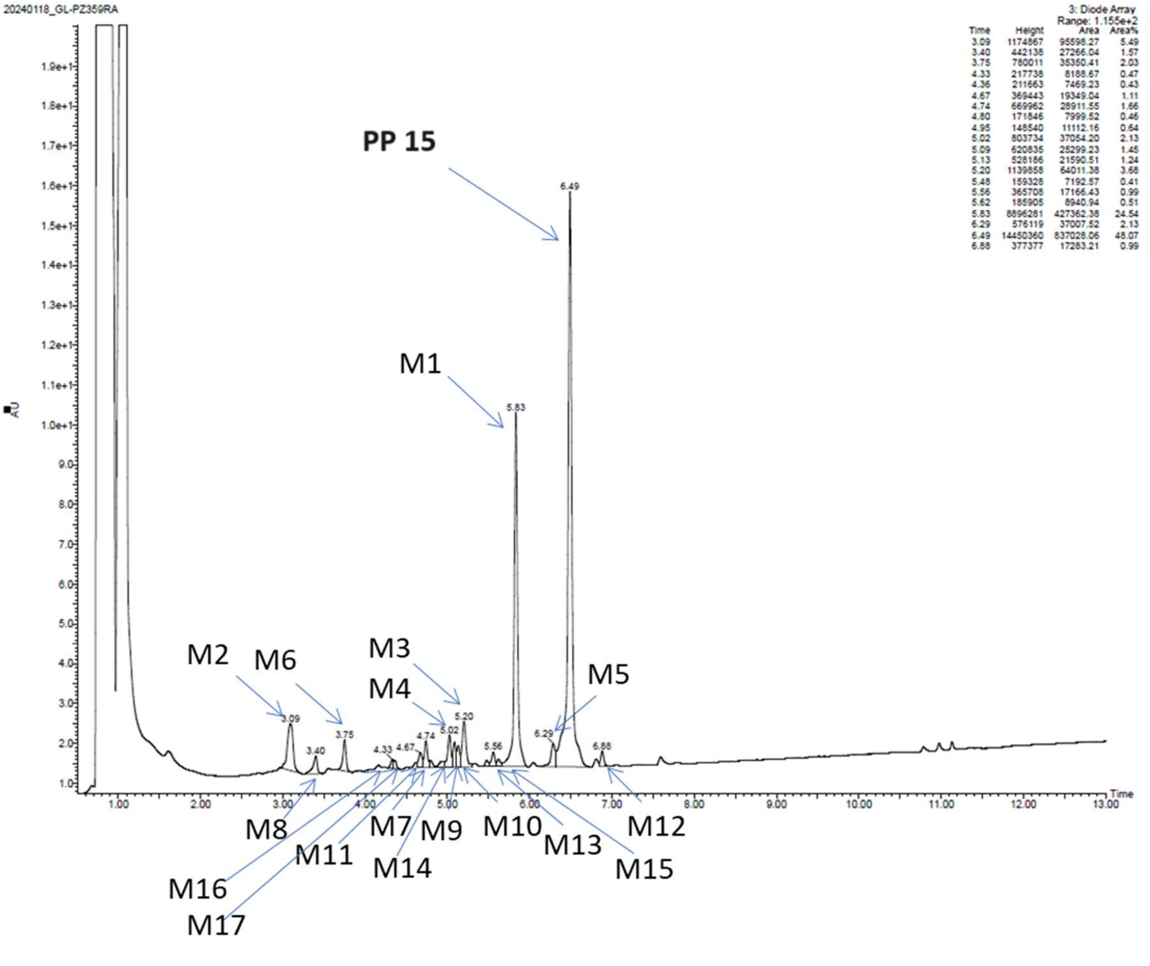

Figure S140. UPLC spectra after 120 min incubation of compound **PP 15** with MLMs.

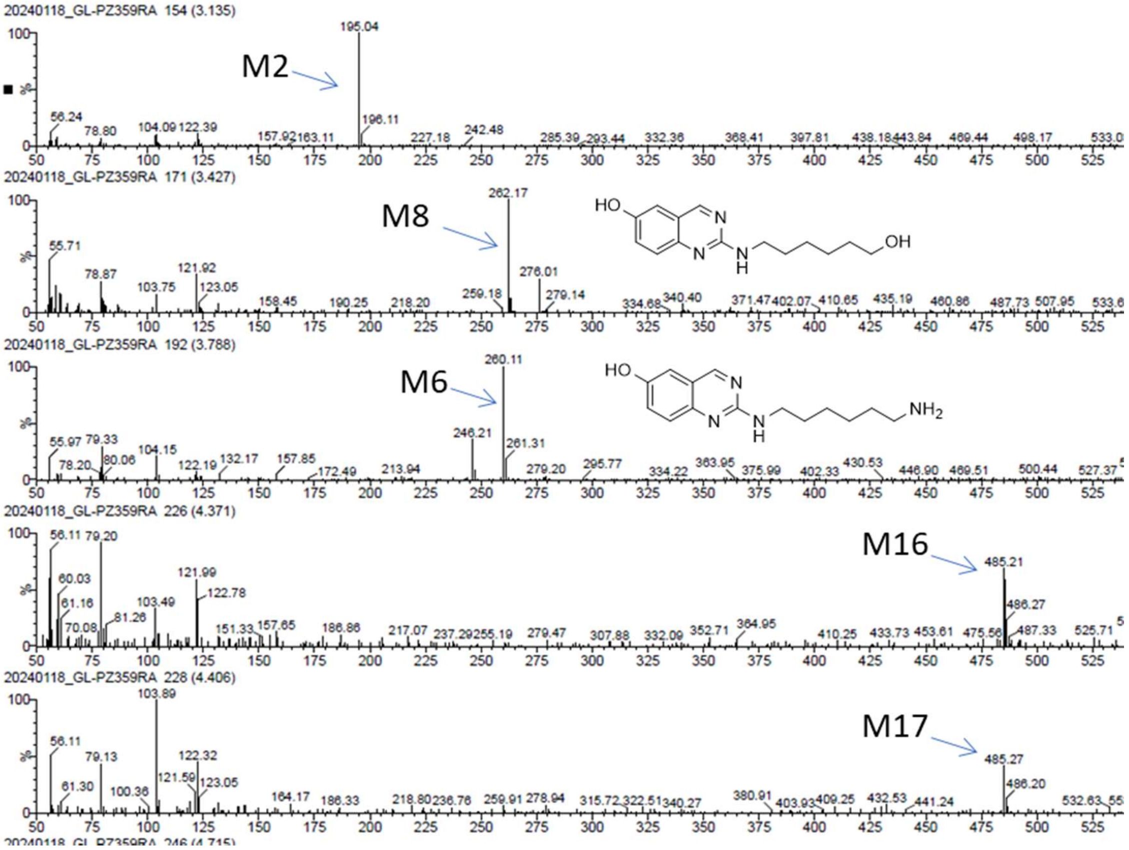
20240118_GL-PZ359RA 154 (3.135)
M2
195.04
20240118_GL-PZ359RA 171 (3.427)
M8
262.17
HO
N
N
N
H
OH
20240118_GL-PZ359RA 192 (3.788)
M6
260.11
HO
N
N
N
NH_2
20240118_GL-PZ359RA 226 (4.371)
M16
485.21
20240118_GL-PZ359RA 228 (4.406)
M17
485.27

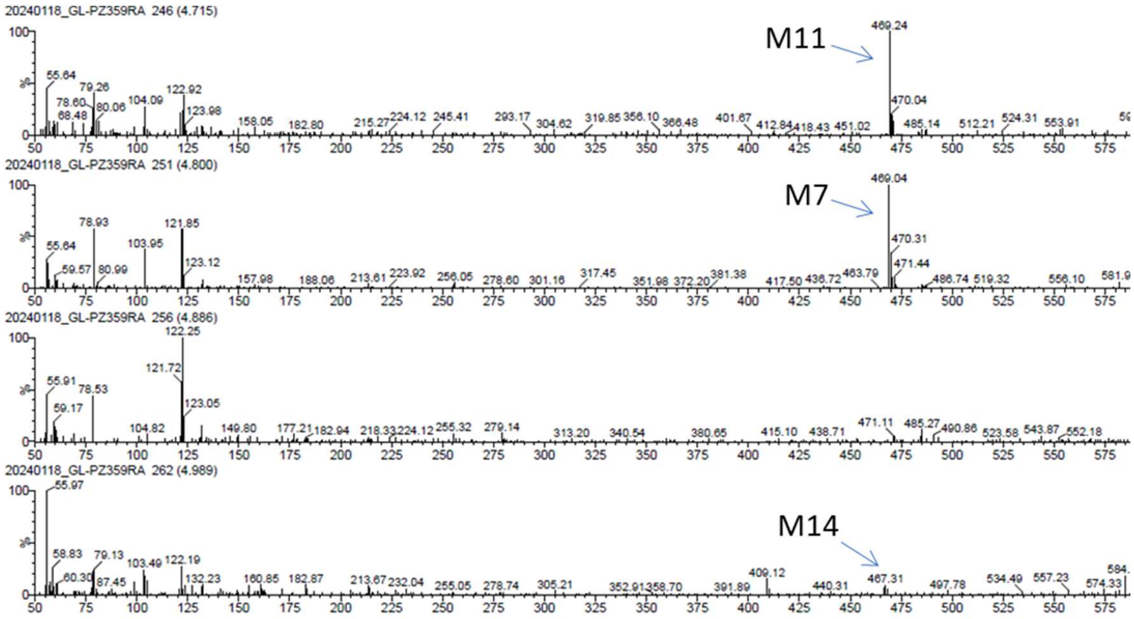
20240118_GL-PZ359RA 246 (4.715)
M11
469.24
20240118_GL-PZ359RA 251 (4.800)
M7
469.04
20240118_GL-PZ359RA 256 (4.886)
122.25
20240118_GL-PZ359RA 262 (4.989)
M14
467.31

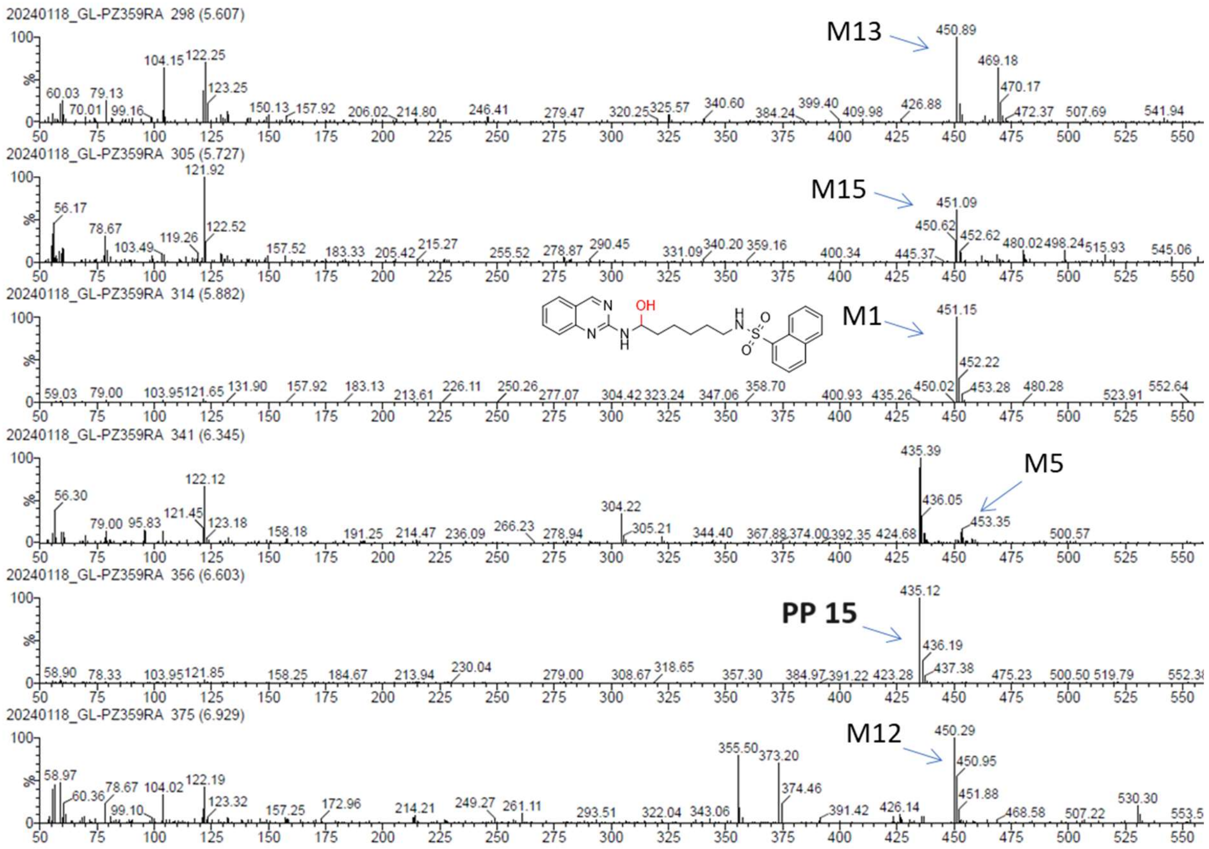

Figure S141. MS analyses of **PP 15** and metabolites.

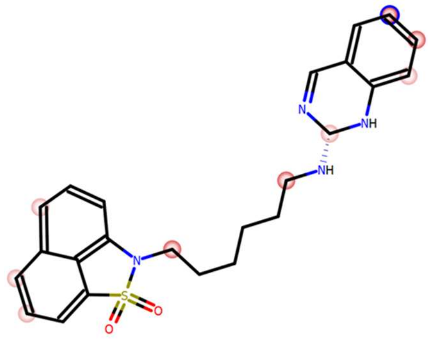

Figure S142. The MetaSite 6.0.1. software prediction of the most probable sites of compound **PP 24** metabolism. The darker red color - the higher probability to be involved in the metabolism pathway. The blue circle marked the site of compound with the highest probability of metabolic bioconversion.

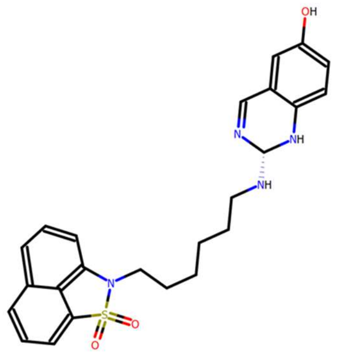

100%

Figure S143. The MetaSite 6.0.1. software prediction of the most probable hydroxylation site of **PP 24**

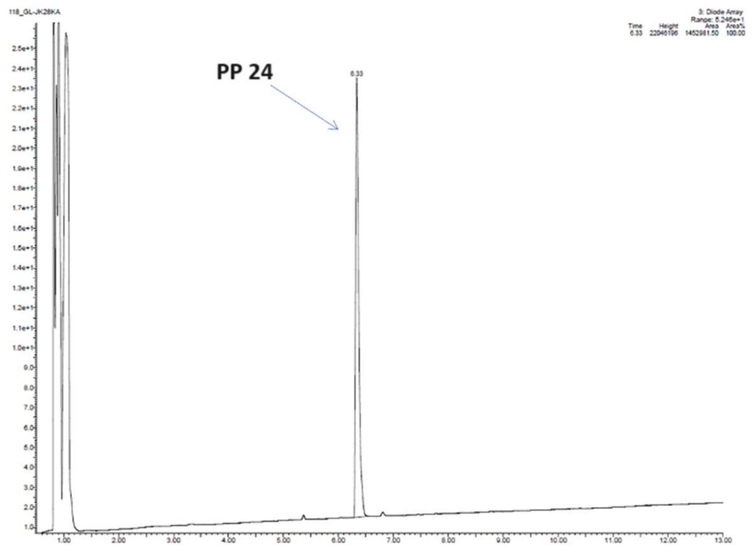

Figure S144. UPLC spectra after 120 min incubation of compound **PP 24** in buffer.

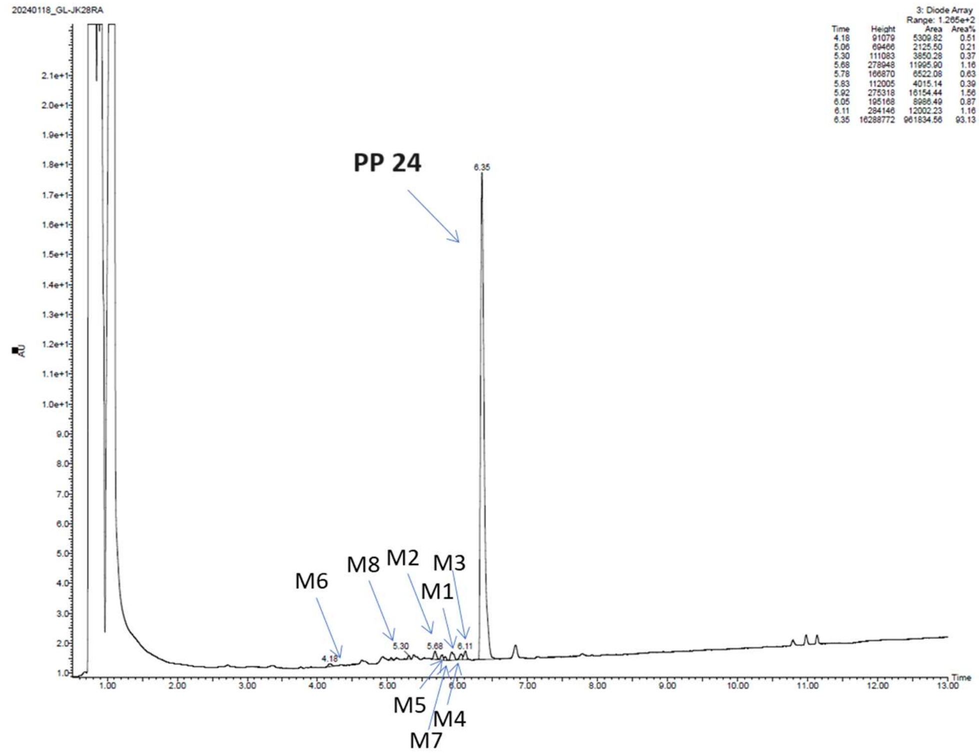

Figure S145. UPLC spectra after 120 min incubation of compound **PP 24** with MLMs.

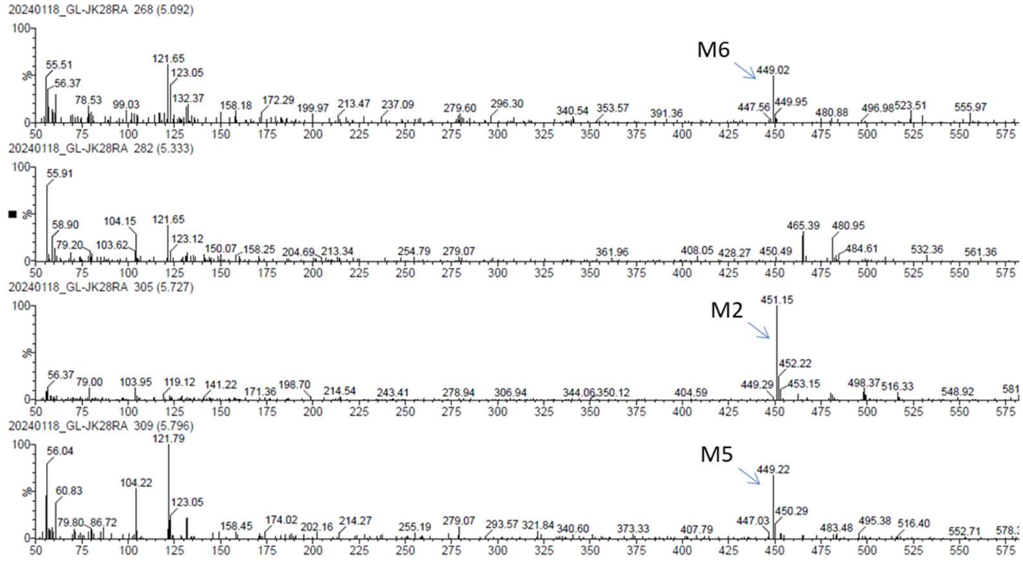

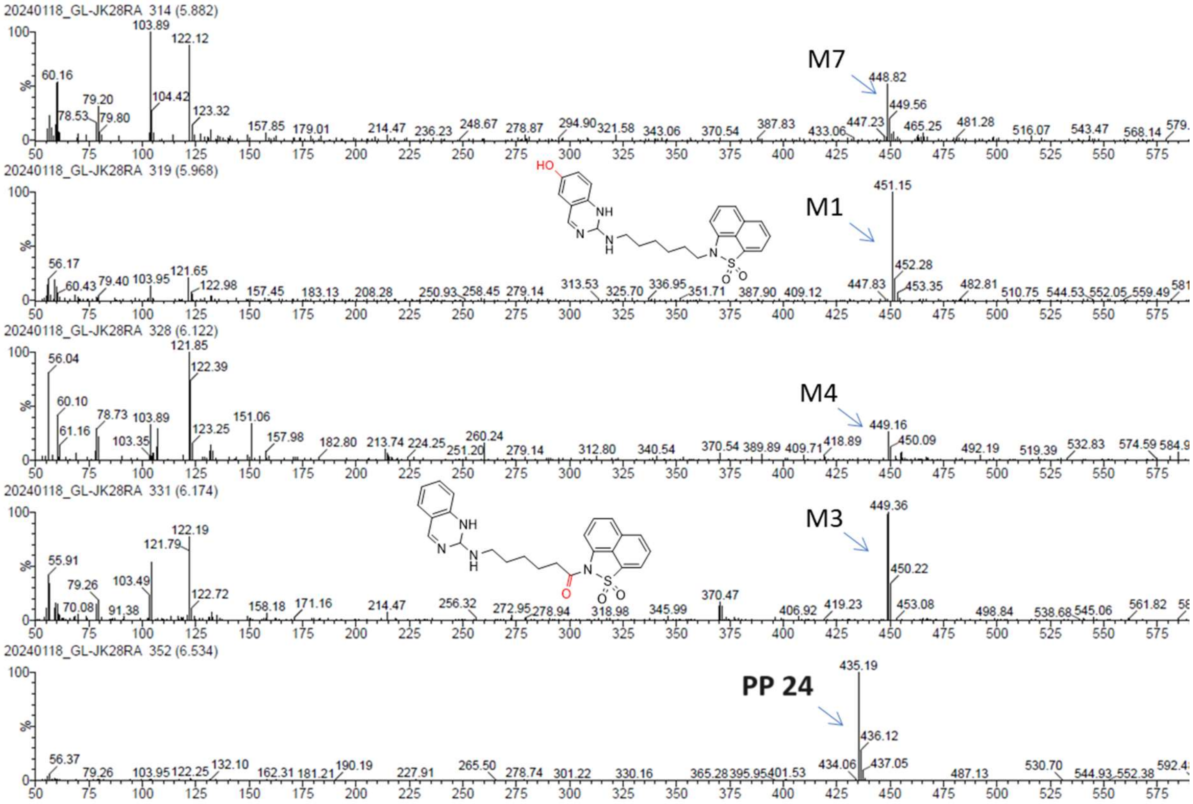

Figure S146. MS analyses of **PP 24** and metabolites.

5. Distribution - plasma protein binding (PPB)

Table S4. Plasma protein binding.

**Comp.**	**k_D** µM	**f_b** % ± SD
Warfarin	9.5	98.5 ± 2.30
**PP 10**	**2.1**	**99.7** ± 0.16
**PP 15**	**0.2**	**100.0** ± 0.06
**PP 13**	**0.4**	**99.9** ± 0.03
**PP 24**	**2.7**	**99.6** ± 0.13

k_D = dissociation constant, **f_b =** fraction bound

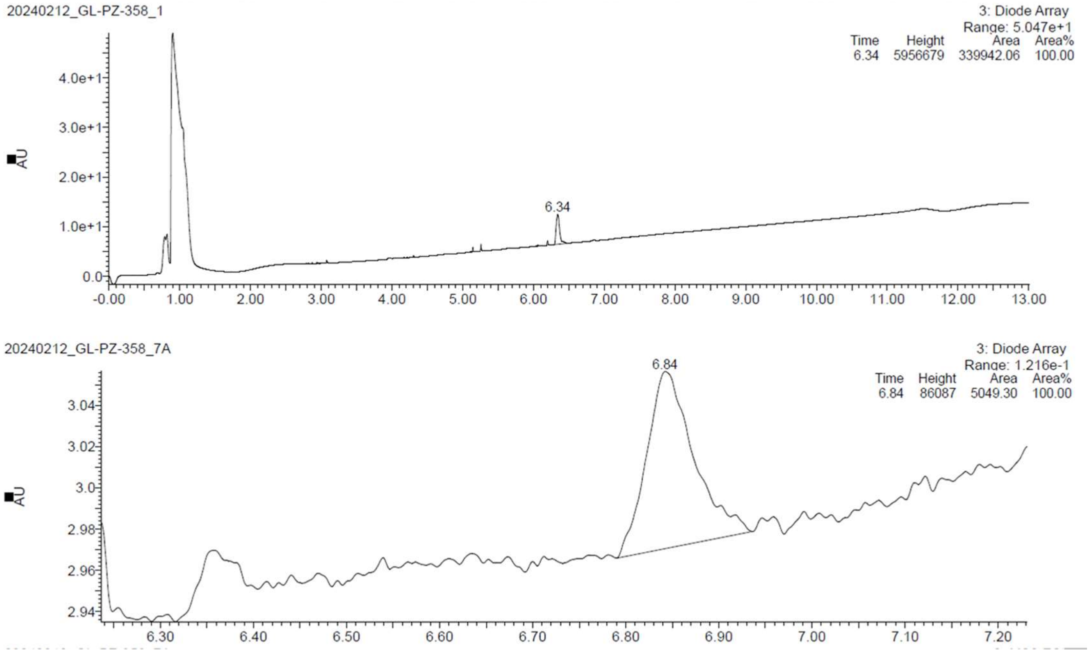

Figure S147. UPLC of **PP 10** solution in PBS - control (above). The amount of **PP 10** remaining after incubation with human plasma proteins (below).

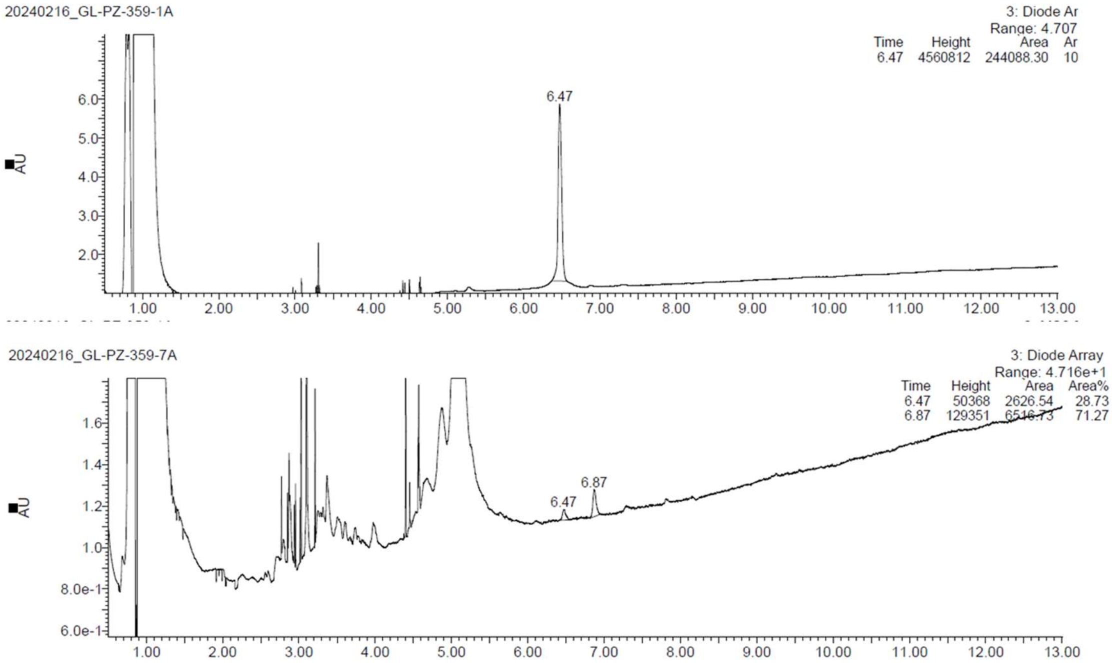

Figure S148. UPLC of **PP 15** solution in PBS - control (above). The amount of **PP 15** remaining after incubation with human plasma proteins (below).

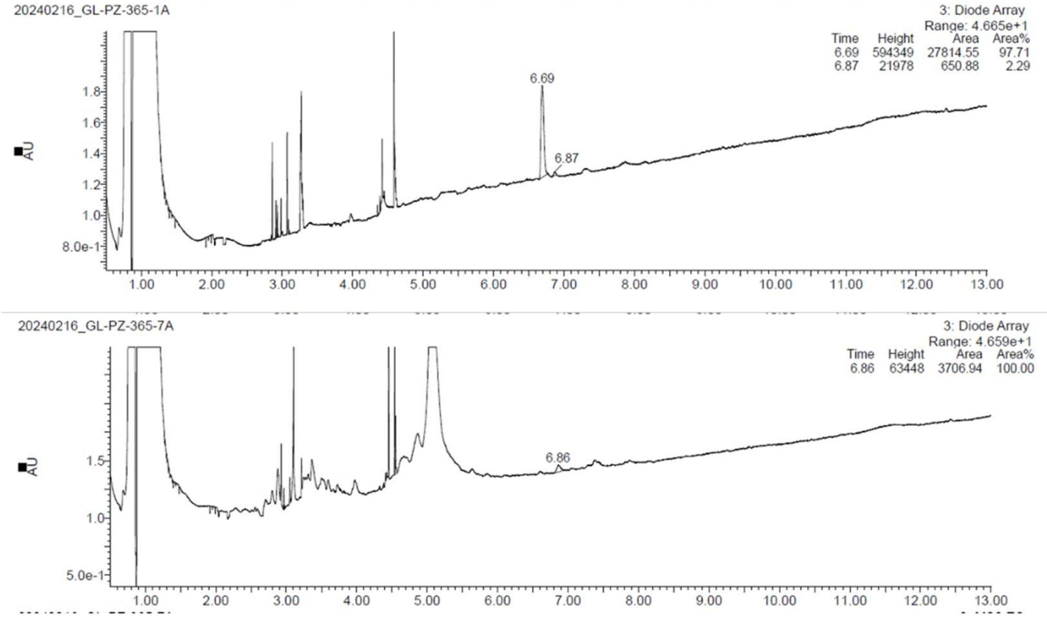

Figure S149. UPLC of **PP 13** solution in PBS - control (above). The amount of **PP 13** remaining after incubation with human plasma proteins – COMPOUND NOT DETECTED (below)

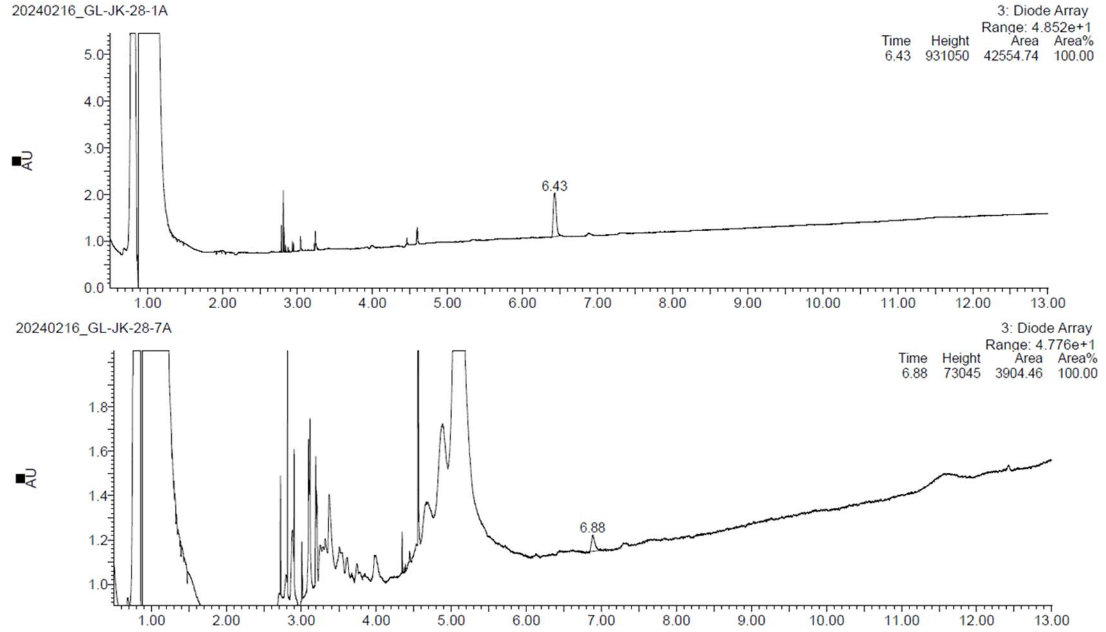

Figure S150. UPLC of **PP 24** solution in PBS - control (above). The amount of **PP 24** remaining after incubation with human plasma proteins – COMPOUND NOT DETECTED (below)

6. Comparison of known serotonin receptor ligands

Table S5. Comparison of known serotonin receptor ligands.

Receptor	5-HT_6	D_2	5-HT_{1A}	5-HT_{5A}	5-HT_7
Structure	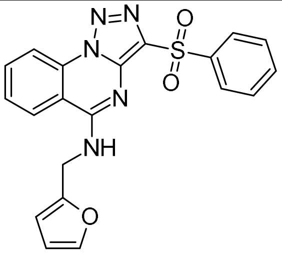	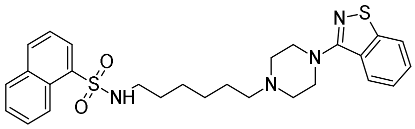	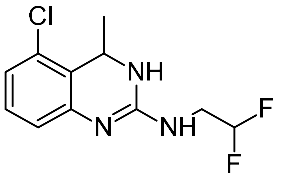		
K_i [nM]	74	31	85	10	33
Ref	Ivachtchenko AV, et al. J Comb Chem. 2010 Jul 12;12(4):445-52. doi: 10.1021/cc1000049.	Zaręba P, et al. Bioorg Med Chem. 2020 May 15;28(10):115459.	Peters JU, et al. Bioorg Med Chem Lett. 2008 Jan 1;18(1):256-61. doi: 10.1016/j.bmcl.2007.10.080.		

7. Biochromatographic data

Table S6 Biochromatographic dat

Sample		C18 at pH 2.6						C18 at pH 7.4						C18 at pH 10.5						IAM						HSA							
L.p.	Name	t1	t2	t3	tm	SD	CHI C18	t1	t2	t3	tm	SD	CHI C18	t1	t2	t3	tm	SD	CHI C18	t1	t2	t3	tm	SD	CHI IAM	t1	t2	t3	tm	SD	log(t)	logKHSA	%HSA
1	PP 15	3.83	3.86	3.87	3.85	0.025	99.0	3.99	4.02	4.03	4.01	0.0189	104.4	3.96	3.96	3.96	3.96	0.0023	107.2	4.59	4.58	4.59	4.58	0.0065	45.8	17.69	17.65	17.62	17.65	0.036	1.25	1.48	97.8
2	PP 10	3.58	3.60	3.60	3.59	0.011	89.8	3.85	3.88	3.90	3.88	0.0239	99.7	4.35	4.31	4.29	4.32	0.0301	120.4	5.28	5.28	5.27	5.28	0.0055	56.4	17.40	17.45	17.48	17.44	0.039	1.24	1.47	97.7
3	PP 13	4.44	4.38	4.15	4.32	0.156	115.8	4.05	4.06	4.04	4.05	0.0096	105.7	4.58	4.55	4.50	4.54	0.0391	128.8	5.52	5.52	5.51	5.51	0.0070	59.9	>30	>30	>30					> 99,8
4	PP 6	3.81	3.70	3.53	3.68	0.139	92.9	3.91	3.95	3.94	3.93	0.0196	101.5	4.88	4.81	4.70	4.80	0.0876	138.2	4.92	4.93	4.92	4.92	0.0055	50.9	16.14	16.12	16.12	16.13	0.009	1.21	1.41	97.2
5	PP 4	2.89	2.90	2.87	2.89	0.014	64.9	3.30	3.31	3.32	3.31	0.0127	79.9	3.85	3.83	3.76	3.82	0.0480	101.9	4.88	4.88	4.87	4.88	0.0084	50.2	12.03	12.09	12.13	12.08	0.051	1.08	1.18	94.8
6	PP 14	3.24	3.28	3.28	3.27	0.023	78.3	3.52	3.46	3.49	3.49	0.0300	86.3	3.44	3.42	3.39	3.42	0.0269	87.1	4.02	4.02	4.02	4.02	0.0035	37.2	12.36	12.32	12.27	12.32	0.044	1.09	1.20	95.0
7	PP 11	3.99	3.97	3.96	3.97	0.013	103.4	4.02	3.95	3.96	3.97	0.0412	103.1	3.58	3.57	3.58	3.58	0.0075	93.1	5.53	5.53	5.53	5.53	0.0032	60.1	23.28	22.56	23.02	22.95	0.363	1.36	1.75	99.2
8	PP 12	3.78	3.82	3.79	3.80	0.022	97.1	4.22	4.22	4.19	4.21	0.0206	111.2	4.36	4.40	4.30	4.35	0.0512	121.8	5.48	5.48	5.48	5.48	0.0029	59.4	27.93	27.73	26.04	27.23	1.037	1.44	1.90	99.7
9	PP 24	3.43	3.43	3.48	3.45	0.032	84.7	4.02	3.95	3.97	3.98	0.0343	103.2	4.54	4.49	4.46	4.49	0.0412	127.0	5.29	5.30	5.29	5.29	0.0053	56.5	20.96	20.90	19.27	20.37	0.960	1.31	1.65	98.8
10	PP 22	2.64	2.66	2.65	2.65	0.008	56.4	4.32	4.37	4.33	4.34	0.0244	115.8	5.68	5.63	5.56	5.62	0.0562	168.7	5.28	5.29	5.28	5.28	0.0050	56.4	18.91	18.82	19.12	18.95	0.154	1.28	1.59	98.4
	**Calibration set**																																
	Theophylline	1.20	1.22	1.21	1.21	0.007	17.9	1.59	1.58	1.59	1.59	0.005		1.21	1.26	1.27	1.25	1.26															
	Benzimidazole	1.66	1.61	1.60	1.62	0.033	6.3	1.92	1.91	1.93	1.92	0.008		1.79	1.83	1.85	1.82	1.83															
	Colichicine	2.60	2.58	2.58	2.58	0.012	43.9	2.59	2.59	2.59	2.59	0.000		2.49	2.53	2.60	2.54	2.56															
	Acetophenone	2.67	2.66	2.66	2.66	0.006	64.1	2.66	2.66	2.66	2.66	0.003		2.54	2.61	2.61	2.59	2.60															
	Indole	2.98	2.98	2.99	2.98	0.006	72.1	3.01	3.00	3.01	3.01	0.006		2.89	2.88	2.87	2.88	2.88															
	Propiophenone	3.18	3.08	3.08	3.11	0.058	77.4	3.08	3.09	3.08	3.08	0.009		2.97	3.02	3.02	3.00	3.01															
	Butyrophenone	3.59	3.51	3.51	3.53	0.046	87.3	3.54	3.51	3.53	3.53	0.017		3.40	3.45	3.42	3.42	3.43															
	Valerophenone	3.94	3.85	3.86	3.88	0.047	96.4	3.88	3.85	3.87	3.87	0.016		3.75	3.79	3.79	3.78	3.79															
	Paracetamol																		Paracetamol	1.88	1.88	1.88	1.88	0.004	2.9								
	Acetanilidine																		Acetanilidine	2.31	2.31	2.31	2.31	0.003	11.5								
	Acetophenone																		Acetophenone	2.61	2.61	2.60	2.61	0.003	17.2								
	Propiohenone																		Propiohenone	3.20	3.20	3.19	3.19	0.005	25.9								
	Butyrophenone																		Butyrophenone	3.66	3.66	3.65	3.65	0.006	32								
	Valerophenone																		Valerophenone	4.04	4.05	4.03	4.04	0.007	37.3								
	Hexanophenone																		Hexanophenone	4.37	4.37	4.36	4.36	0.007	41.8								
	Heptanophenone																		Heptanophenone	4.63	4.63	4.62	4.63	0.009	45.7								
	Octanophenone																		Octanophenone	4.86	4.87	4.83	4.85	0.021	49.4								
	Paracetamol																		paracetamol							1.16	1.15	1.15	1.15	0.008	0.06		
	Nizatidine																		nizatidine							1.38	1.36	1.40	1.38	0.018	0.14		
	Trimetoprim																		trimetoprim							2.18	2.18	2.18	2.18	0.003	0.34		
	Carbamazepine																		carbamazepine							4.06	4.07	4.09	4.07	0.017	0.61		
	Propranolol																		propranolol							10.62	10.66	10.62	10.63	0.020	1.03		
	Nicardipine																		nicardipine							11.74	11.78	11.87	11.80	0.067	1.07		
	Warfarin																		warfarin							16.35	16.41	16.46	16.41	0.057	1.22		
	Diclofenac																		diclofenac							23.07	23.03	23.21	23.10	0.096	1.36		
	Indometacin																		indometacin							23.65	23.79	24.22	23.89	0.300	1.38		
